# Spectroscopy of C_120_^−^ and larger fulleride cluster monoanions in the mid-infrared

**DOI:** 10.1039/d5cp03392f

**Published:** 2025-09-17

**Authors:** Miriam Kappe, Gabriel Schöpfer, Arne Schiller, Elisabeth Gruber, Milan Ončák, Andrew M. Ellis, Paul Scheier

**Affiliations:** a Institut für Ionenphysik und Angewandte Physik, Universität Innsbruck Technikerstr. 25 A-6020 Innsbruck Austria milan.oncak@uibk.ac.at andrew.ellis@le.ac.uk; b Institute for Breath Research, Universität Innsbruck Innrain 66 A-6020 Innsbruck Austria; c School of Chemistry, University of Leicester University Road Leicester LE1 7RH UK

## Abstract

An optical spectrum of the singly charged anionic dimer of C_60_, the C_120_^−^ anion, is reported for the first time. This spectrum, recorded in the mid-infrared and extending from 800 cm^−1^ through to 2200 cm^−1^, shows a mixture of discrete peaks and broader features. An assignment of this spectrum poses a major challenge for theory. A broad feature observed above 1600 cm^−1^ can be unambiguously assigned to one or many electronic transitions. However, it is not clear which isomer is responsible for it, as many isomers show electronic transitions in this spectral range. The origin of peaks below 1600 cm^−1^ in the experimental spectrum remains uncertain, as calculations predict both electronic and vibrational transitions in this spectral range for various isomers. The proximity of several electronic and vibrational transitions suggests a breakdown of the Born–Oppenheimer approximation. This suspicion is supported by the fact that the vibrational spectrum is very dependent on the computational method, much more than expected for small molecules in the gas phase. All in all, C_120_^−^ seems to be at the brink of what is computationally feasible with current methods of quantum chemistry. We also report mid-IR spectra for the larger cluster anions, C_180_^−^ and C_240_^−^, whose spectra show significant similarities to that of C_120_^−^.

## Abstract 2

An optical spectrum of the singly charged anionic dimer of C_60_, the C_120_^−^ anion, is reported for the first time.

## Graphical abstract

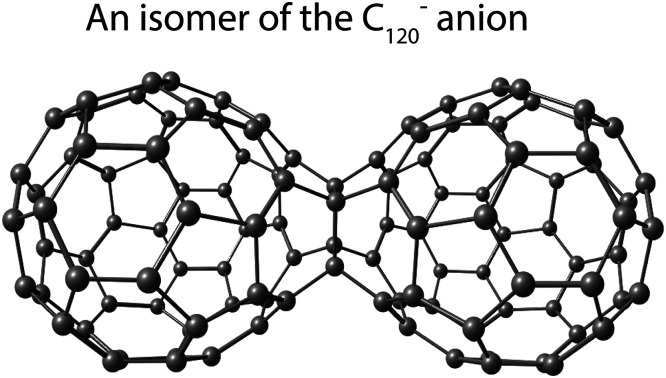

## I. Introduction

Ever since the discovery of the bulk synthesis of fullerenes, there has been much interest in the anions of fullerenes, the fullerides. That interest derives from the fact that many chemical compounds containing fullerenes come in the form of metal-containing salts, in which the fullerene is anionic.^1,2^ Good examples are the salts provided by the combination of alkali atoms (M) with C_60_. These can have a variety of stoichiometries, M_*x*_C_60_, where *x* can be as low as 1 (RbC_60_) and as large as 12 (Li_12_C_60_), although the latter are better viewed as intercalated compounds rather than conventional salts. Nevertheless, C_60_ is clearly a strong electron acceptor and the charge on the C_60_^*n*−^ anion can vary depending on the identity of the metal counterion. Perhaps the most intriguing of all of the alkali fullerides are the M_3_C_60_ salts, which have been found to be (relatively) high temperature superconductors.^3–5^ Critical to the superconductivity in these salts is the role of the half-filled conduction band arising from the periodic array of C_60_^3−^ ions and the resulting electron–phonon coupling.^4^

C_60_ is a highly symmetric molecule (*I*_h_ point group symmetry) with closed electronic shells in its ground electronic state. Its lowest unoccupied molecular orbital (LUMO) has *t*_1u_ symmetry, so when C_60_ acquires a single additional electron, it enters this triply-degenerate orbital in the electronic ground state of the anion. The electron affinity of C_60_ is high, 2.6835 ± 0.0006 eV,^6^ and so the electron in the *t*_1u_ LUMO is more tightly bound to the C_60_ core than the LUMO in many other anions. Unlike most anions, C_60_^−^ has several bound electronic excited states and electron correlation is critical for a realistic description of these states, *i.e.* they are unbound at the Hartree–Fock level of theory.^7–10^ Furthermore, the nominal high symmetry of C_60_^−^ delivers multiple electronic and vibrational degeneracies, which are removed by the Jahn–Teller effect.^11–14^ Understanding the behaviour of C_60_^−^ therefore requires a detailed grasp of both electron behaviour and the impact of Jahn–Teller dynamics.

There have been several experimental studies of isolated C_60_^−^. Early work using photoelectron spectroscopy was limited in its resolution, largely because of the residual temperature resulting from the need to evaporate C_60_, which causes the population of many low-lying vibrational states.^15–17^ However, with the introduction of cryogenic cooling, the resolution can be improved significantly and, along with providing new information on the vibrational states in the anion and neutral molecule, such studies have also provided more detailed probes of the vibronic coupling in the electronic ground state of C_60_^−^.^6,18^ Studies of the laser-induced electron detachment from C_60_^−^ ions following tuneable near-infrared excitation have provided some experimental information on the excited electronic states of C_60_^−^.^19,20^ Subsequently, using two-colour time-resolved photoelectron spectroscopy, the lifetime of one of those states, identified as the B̃^2^E_g_ state, was found to be 2.2 ps.^21^ These investigations in the gas phase complement earlier work on C_60_^−^ trapped in rare gas matrices, where near-infrared absorption features have been assigned to electronic excitation.^22,23^

Very recently, the same electronic absorption features have been recorded at higher resolution in the gas phase using helium-tagged C_60_^−^ ions.^24^ This study employed electron attachment to create negatively charged helium droplets, which were then used to capture C_60_ molecules. By allowing the helium droplets to collide with a metal surface, anions tagged with one or more loosely bound helium atoms could be produced in the gas phase. Optical spectra were then recorded by vibrational predissociation spectroscopy. The benefit of this approach is that helium is a weak perturber of the spectrum of C_60_^−^ and so these measurements could potentially form the basis for astronomical searches for this anion.

C_60_^−^ has also been studied in the mid-infrared. Early work exploiting a neon matrix to isolate the ions revealed a number of peaks between 1100–1450 cm^−1^, which were assigned to vibrational transitions.^23^ Subsequently, Kern *et al.* were able to obtain cleaner spectra by externally generating ions and then, using a mass filter, co-depositing these ions with the desired matrix material.^25^ The advantage of this approach, as opposed to the more usual matrix deposition without a mass filter, is that it removes contributions from neutral C_60_. Good agreement was obtained between the IR spectrum and density functional theory predictions and a Jahn–Teller distorted ground electronic state with *D*_3d_ point group symmetry was inferred from the spectrum. Two of the bands seen in the matrix work have also been recorded in a gas phase IR spectrum of C_60_^−^.^26^

Although much is now known about C_60_^−^, almost nothing is known about the corresponding singly charged anionic clusters. The anionic dimer, (C_60_)_2_^−^, was once postulated^27^ as a possible source of a sharp line persistently seen in the EPR spectra of solutions containing the C_60_^−^ anion.^28,29^ However, subsequent work has shown that the impurity species, C_120_O^−^, is responsible for that sharp feature,^30^ and so the singly charged dimer anion remains an unknown entity. If formed, it could be expressed as (C_60_)_2_^−^ or C_120_^−^, depending on whether it consists of two recognisable C_60_ units, as implied in the case of (C_60_)_2_^−^, or is very heavily coalesced into a single fullerene unit, in which C_120_^−^ would be a more appropriate label. We will tend to avoid this distinction and will just refer to the ion as C_120_^−^.

In recent work we reported the first IR spectrum of the cationic equivalent, C_120_^+^, presenting evidence for a peanut-shaped structure in our experiments.^31^ Here we address the corresponding anion formed under similar experimental conditions, alongside the trimer and tetramer, recording the first optical spectra of these ions. As will be seen, the spectra of these cluster anions are challenging to assign because of the complexity of the potential energy landscape and the likelihood of a serious breakdown of the Born–Oppenheimer approximation.

## II. Experimental and computational details

Full details of the experimental setup can be found elsewhere,^32^ so only a brief account is provided here. Central to the experiment is the production of negatively charged helium nanodroplets, which was achieved using electron impact at *ca.* 22 eV. This leads to the formation of He*^−^ anions within helium droplets,^33,34^ which can act as powerful reducing agents.^35^ The negatively charged helium droplets were then doped with C_60_ molecules by passage of the droplets through the vapor emanating from a resistively heated oven containing solid C_60_. The average number of C_60_ molecules acquired by a helium droplet is dictated by the size of the droplet (collision cross section), the partial pressure of C_60_ vapor in the oven, and the length of the pickup zone (which is fixed). Collision of these charged droplets with a stainless-steel surface leads to splashing and extraction of lower-mass ions.^32^ In addition to bare anions such as C_60_^−^ and its dimer C_120_^−^, some of the fulleride anions are ejected into the gas phase with one or more helium atoms attached.

Optical spectra were then recorded by monitoring the production of excess bare anions induced by photodissociation (loss of all helium atoms) from the helium-tagged anions as a function of laser wavelength. The laser system used was the Ekspla model NT273 XIR (bandwidth <10 cm^−1^, 833–2230 cm^−1^). The recorded absorption features were corrected for changes in laser power as a function of wavelength. Note that, because we are potentially seeing signal from anions with different numbers of attached helium atoms, an inhomogeneous broadening effect will result as each of these ions will absorb at slightly different frequencies.

Supporting the experimental findings about C_120_^−^ with theoretical calculations is complex, not least because there are multiple potential isomers that must be considered, which we distinguish here into four classes, namely as fully reconstructed buckyball (B), peanut-shaped (P), covalently bound dimers (C) and van der Waals (V) structures (see SI for all calculated stable isomers, *i.e.* isomers whose energy lies below the energy of C_60_^−^ + C_60_); the structures were taken from our C_120_^+^ study,^31^ and based also on previous work by Onoe and co-workers.^36,37^

We have performed density functional theory (DFT) calculations on 29 isomers of C_120_^−^ with the BP86/def2-SVP method to obtain geometries and vibrational frequencies, and TD-BMK/6-31+G* to calculate electronic excitations. For benchmarking purposes, we also calculated structures and vibrational frequencies of C_60_, C_60_^−^ and the four isomers B1, P1, C1 and V1 of C_120_^−^ using eight further methods of quantum chemistry, namely ωB97XD/def2-SVP, BHandHLYP/def2-SVP, BMK/def2-SVP, M06L/def2-SVP, B3LYP/def2-SVP, BLYP/def2-SVP, BP86/def2-TZVP and PBE/def2-SVP. Where possible, we applied density fitting and Grimme's empirical dispersion correction D3.^38^ To calculate electronic excitations of C_60_^−^ and the four isomers B1, P1, C1 and V1 of C_120_^−^, we used five different DFT methods, namely TD-BHandHLYP/6-31+G*, TD-BHandHLYP/def2-TZVP, TD-BMK/6-31+G*, TD-BMK/def2-TZVP and TD-CAM-B3LYP/6-31+G*, which were applied to geometries obtained from BP86/def2-SVP, BP86/def2-TZVP and B3LYP/def2-SVP, making a total of 15 calculations for each of these five molecules. Wave function stabilisation^39^ was performed prior to every calculation, to make sure that the electronic wave function, which was used for geometry optimization or as a starting point for calculation of electronically excited states, is the wave function of the electronic ground state.

To compare structures in the excited state to the ground state structure, we used root mean square displacement (RMSD) as a measure of structural similarity. For example, an RMSD of 0.01 Å means that, on average, every nucleus in the excited electronic state is 0.01 Å away from its original position in the ground electronic state. All calculations were performed in Gaussian 16.^40^

## III. Results and discussion

### 1. Infrared spectroscopy of C_60_^−^

Initial work started with an attempt to record a spectrum of C_60_^−^ in the mid-IR by monitoring wavelength-dependent changes in the anion signal at *m*/*z* 720. Spectra in this region have been successfully recorded previously in a neon matrix^23,25^ and in the gas phase.^26^ A number of discrete bands have been seen in these spectra which have been attributed to vibrational transitions. However, and in contrast to the previous work, we were unable to see any absorption features between 800–2200 cm^−1^ when monitoring C_60_^−^. We assume that this is because the vibrational transitions of C_60_^−^ are too weak for us to observe in our current experiments. We will return to this in the next section.

We performed DFT calculations to predict the IR vibrational spectrum of C_60_ and C_60_^−^, as can be seen in Fig. S1. In the case of the vibrational spectra of C_60_, all methods provide very similar results. It is, however, interesting that there is such a quite strong method-dependence for the calculated vibrational spectrum of C_60_^−^. By comparing our results to an experimental spectrum of C_60_^−^ in the mid-IR from Kern *et al.*,^25^ we identify BLYP/def2-SVP, BP86/def2-SVP, BP86/def2-TZVP and PBE/def2-SVP, as the most reliable methods among those investigated, because the vibrational spectra from these methods match very well with the experimental one. In the case of the calculated spectrum, only two electronic transitions are predicted for C_60_^−^ in the measured energy range covered in the present work (see Fig. S2), both of which are forbidden by symmetry.

### 2. Infrared spectrum of C_120_^−^

**Fig. 1:**
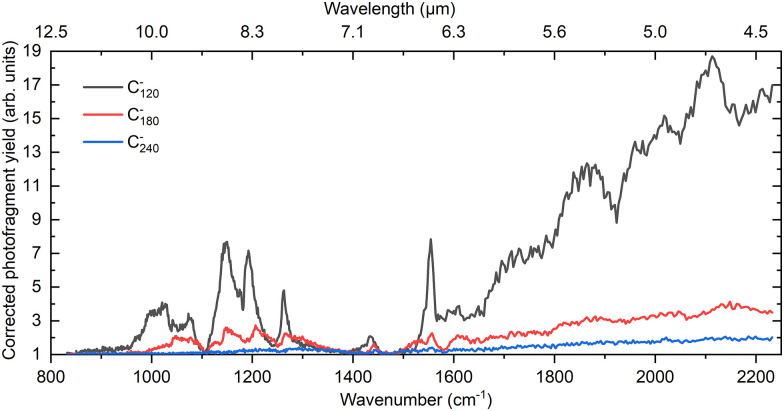
IR spectrum of helium-tagged C_120_^−^ ions alongside those of C_180_^−^ and C_240_^−^. The photofragment yield has been corrected for both variations in the laser power and the background signal in the absence of the laser.

In contrast to the null spectrum for C_60_^−^, a variety of absorption features are seen in the IR spectrum detected at *m*/*z* 1440, which corresponds to the C_120_^−^ anion. The spectrum obtained is shown by the black trace in Fig. 1. Discrete structure is more evident at lower frequencies while in the higher frequency part of the spectrum there is some broad structure built upon a rising absorption background. The low-frequency structure shows a strong resemblance to the vibrational features observed in the IR spectra of C_60_^+^ and C_60_^−^,^23,25^ as well as the C_120_^+^ ion.^31^ We therefore consider whether vibrational structure might be responsible for the peaks below 1600 cm^−1^ in the spectrum for C_120_^−^.

**Fig. 2:**
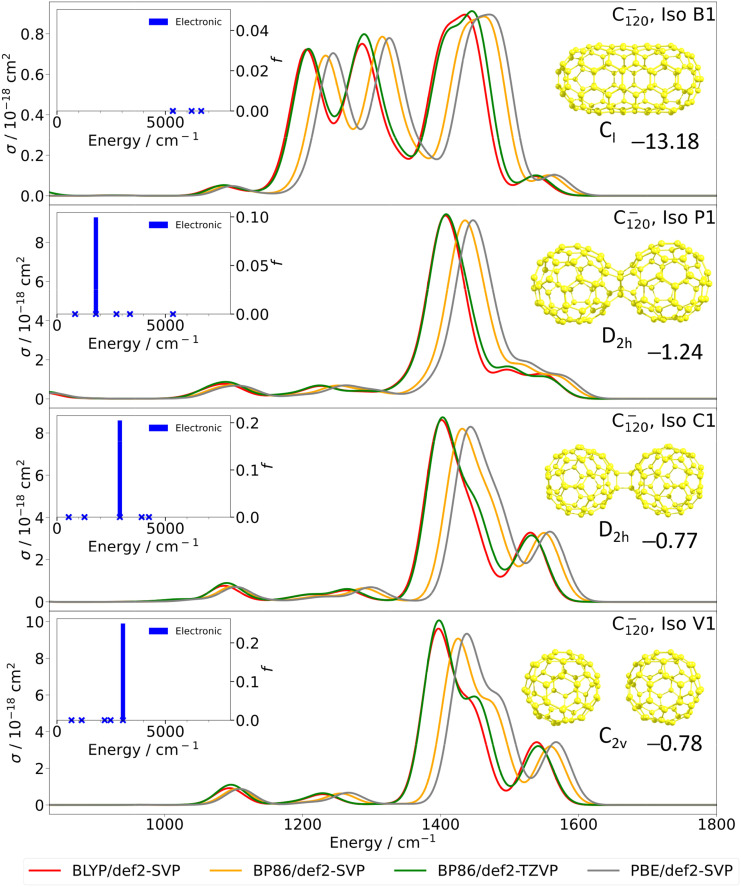
Calculated spectra of C_120_^−^ for four different isomers labelled as B_1_, P_1_, C_1_ and V_1_ from top to bottom. The structures and symmetries of the isomers are also shown in the upper right of each panel. The vibrational spectra from four different DFT-based methods are shown, as indicated by the legend at the bottom of the figure. To calculate the electronic spectra, TD-BMK/6-31+G*//BP86/def2-SVP methodology was employed and the resulting electronic transitions are identified in the insets in the upper left side of each panel (electronic excitations). The stabilisation energy in eV with respect to dissociation into C_60_^−^ + C_60_ at the BP86/def2-TZVP level of theory is also shown beneath each structure in each panel. Vibrational and electronic transitions are shown within the spectral region covered within Fig. 1. The vertical scale for the vibrational spectra is the absorption cross section, *σ*, and an empirical broadening of 50 cm^−1^ has been applied to the vibrational frequencies to generate the calculated spectra shown. For the electronic spectra, each electronic excitation is marked with a blue “x” together with its oscillator strength *f*.

To assist, we have performed DFT calculations for a variety of representative structures, ranging from a fully-fused ‘buckytube’ structure all the way through to a simple dimer structure held together by dispersion forces. One representative structure for each class of isomers is shown in Fig. 2, along with vibrational spectra obtained through the four most reliable methods, as derived from benchmarking calculations on C_60_^−^. In all, 29 distinct isomers were considered, as detailed in the SI (Fig. S3; see Fig. S4 and S5 for further benchmarking and direct comparison with the experiment).

Three things become clear from all of these calculations. First, for most isomers of C_120_^−^, the vibrational absorption cross sections are up to one order of magnitude higher than those calculated for C_60_^−^(see Fig. 2 and Fig. S1, S3–S5). This might be seen as a possible explanation for our inability to observe a spectrum of C_60_^−^ in the mid-IR. However, a second point of note is that none of the calculated vibrational spectra for C_120_^−^ produce vibrational bands that match those seen in the observed experimental spectrum, even if multiple isomers are considered to be present simultaneously. This is seen most clearly in the absence of intense absorption bands below 1300 cm^−1^ in the calculated spectra of these isomers, which is not consistent with the observed experimental spectrum. A detailed comparison can be made by accessing the full set of calculated spectra in Fig. S3. Thirdly, even more than in the case of C_60_^−^, the calculation of vibrational spectra is unexpectedly method-dependent (see Fig. S4). Even though BLYP/def2-SVP, BP86/def2-SVP, BP86/def2-TZVP and PBE/def2-SVP show good agreement with the experiment in the case of C_60_^−^, and these methods are very consistent with each other in the case of C_120_^−^, we cannot rule out that the calculated vibrational spectra are considerably flawed, as other methods predict considerably different vibrational spectra. Our conclusion is therefore that either the vibrational calculations are unexpectedly wrong, or that pure vibrational transitions are not the source of the discrete absorption bands of C_120_^−^ lying below 1600 cm^−1^.

The alternative explanation is that one or more electronic transitions, or a combination of vibrational and electronic transitions, are responsible for the experimentally observed peaks. Electronic transitions in the mid-infrared are indeed predicted for C_120_^−^, as illustrated in Fig. 2 for the four selected structures. The energies and intensities of these electronic transitions are very robust with respect to changing the computational method (see Fig. S6). Interestingly, the electronic transitions seem to be more consistent among different methods than the vibrational ones.

Furthermore, and as expected, the electronic transitions are calculated to be more intense than the pure vibrational transitions (see Fig. S3 in the SI for directly comparable numerical values). In the lower two examples of Fig. 2, we have what could reasonably be described as (C_60_)_2_^−^. Isomer C1 is a dimer of two covalently bound C_60_ units, while isomer V1 is based on two C_60_ units held together by dispersion forces. In a simulation that spans the same wavelength region as the experimental spectrum in Fig. 1, we see weak vibrational structure and, at higher frequencies, an electronic absorption band for these two isomers. The electronic absorption prediction could account for the rising background seen in Fig. 1 above 1500 cm^−1^, lending support to the idea that this arises from electronic absorption. However, as already discussed above, there is poor agreement between the calculated vibrational structure and the sharper bands seen below 1500 cm^−1^ in the experimental spectrum, both in terms of the band positions and their relative intensities.

Isomer P1 shown in Fig. 2 has more extensive covalent bonding between the two C_60_ units and has more of a ‘peanut’ shape. Electronic and vibrational features show the same qualitative behaviour as for the other two isomers, but with the electronic excitation at ∼2000 cm^−1^ being at a lower frequency than for the other two isomers. This shows that, for some structures of C_120_^−^, very low-lying electronic transitions are possible. The fourth representative, isomer B1, is of a buckytube-type, having slightly different vibrational features and no allowed electronic transitions up to 8000 cm^−1^, making it unlikely that this isomer is the main carrier of the experimental spectrum in Fig. 1. In our calculations we have not attempted to predict the vibrational structure accompanying the electronic transitions due to a very complex potential energy surface (PES), as detailed below.

As can be seen in the SI, we have pursued the same type of analysis for all 29 isomers of C_120_^−^ considered in the present work, including the comparison in intensities between vibrations and electronic transitions, and none of them convincingly account for the experimental spectrum of C_120_^−^. So what can we conclude? First, there is no reason why multiple isomers couldn’t contribute to this spectrum. For example, the electronic transitions identified for the isomers shown in Fig. 2 could account for the experimental spectrum. After all, these span almost the entire range of the absorption features seen in Fig. 2. To account for the spectral structure seen experimentally, we might have to assume that the resolved structure corresponds to excitation of particular vibrations in the electronic band system(s). In any case, the complexity of the potential energy landscape provides a serious challenge to finding one or more specific isomers to account for the observed structure.

The findings of our calculations also point to another serious problem, a possible breakdown of the Born–Oppenheimer approximation. The methodology underlying our calculations makes the standard assumption that the Born–Oppenheimer approximation is valid, so that electronic and vibrational motions are fully separated. However, the examples in Fig. 2 show that this is likely to be a poor approximation, with excited electronic and vibrational states in close proximity. This could result in strong electronic–vibrational state mixing, further complicating attempts to understand the IR spectroscopy of C_120_^−^. Note that this is markedly different from C_60_^−^, where the vibrational fundamental and electronic transitions are well separated (see ref. 22–24 and Fig. S1, S2), and where the vibrational spectrum from DFT calculations closely matches the experimental mid-IR spectrum (see ref. 25), even though this is only the case for some DFT methods (see Fig. S1).

**Fig. 3:**
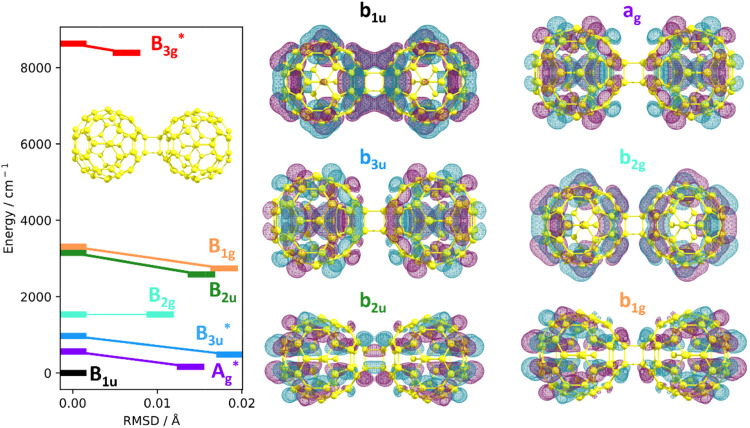
Left: Electronic states in isomer C1 of C_120_^−^ at the BP86-D3/def2-SVP level of theory. Each energy level corresponds to the lowest energy state for the stated irreducible representation, connecting vertical excitation (RMSD = 0) to structures obtained upon nuclear relaxation (RMSD > 0). Structures marked with an asterisk contain an imaginary vibrational frequency. Right: Singly-occupied orbitals of the six lowest states shown in the energy level diagram.

To further analyse the situation, Fig. 3 includes lower-lying electronic states of isomer C1 of C_120_^−^ along with the respective irreducible representations of the states within *D*_2h_ symmetry and the corresponding singly-occupied molecular orbitals. We choose C1 simply as a representative structure to show the impact of electronic excitation on the geometry, but expect similar findings for other isomers. We calculated vertical and adiabatic excitation energies and estimate the difference between two structures through a calculation of the RMSD. Upon optimization, the RMSD value reached 0.006–0.019 Å, showing that the geometrical structure barely changes upon electronic excitation, irrespective of electron localization in a considerably different orbital. The orbitals involved in these excitations are very much delocalized over the entire C_120_^−^ ion. The fact that many electronic states in C_120_^−^ lie close to each other, both energetically and geometrically, strongly influences the potential energy surface of the system and complicates the spectral assignment. Indeed, this phenomenon can also be interpreted as an onset of solid-state behavior, where many electronic states lie close to each other, forming energy bands, and the geometry of the system barely changes upon electronic excitation.

### 3. Infrared spectra of C_180_^−^ and C_240_^−^

Fig. 1 also shows a comparison of the IR spectrum of C_120_^−^ with those from C_180_^−^ and C_240_^−^. The C_180_^−^ spectrum has some significant similarities to that of C_120_^−^, with several peaks below 1600 cm^−1^ matching quite well in the two cases. The spectrum of C_180_^−^ also shows a broadly rising background absorption above 1500 cm^−1^, with some hints of coarse structure on top of that. For C_240_^−^, absorption falls into similar regions as for C_120_^−^ and C_180_^−^. However, any structure is less pronounced than for the two smaller cluster anions.

We have not attempted any calculations on C_180_^−^ and C_240_^−^. However, the similarities seen between the spectra of C_120_^−^, C_180_^−^ and C_240_^−^ indicate that all three anions have some structural features in common, *i.e.* in the way each C_60_ unit is linked to an adjacent one. If that was not the case, we would expect markedly different spectra. One can also expect many low-lying electronic states of the C_180_^−^ and C_240_^−^ anions, complicating the spectral analysis.

## IV. Conclusions

Infrared spectra of the fullerene anions C_120_^−^, C_180_^−^ and C_240_^−^, have been recorded for the first time. The spectra show considerable structure, particularly for C_120_^−^ and C_180_^−^, with several well-resolved bands alongside a structured but broad absorption feature extending to higher frequency. Supporting DFT calculations yield many possible structures for C_120_^−^. The rising background above 1600 cm^−1^ in the experimental spectrum can be attributed to one or many electronic excitations. However, the calculations do not allow for an unambiguous assignment of the experimentally observed features below 1600 cm^−1^ to either vibrational or electronic excitations of any of the investigated isomers.

The calculations do show that vibrational and the lowest-lying electronic states of C_120_^−^ lie in close proximity. Indeed, for some isomers the lowest-lying electronic transition is calculated to lie below the most prominent vibrational fundamental transitions (although possibly inaccessible by allowed electronic transitions). These findings suggest a likely breakdown of the Born–Oppenheimer approximation, which will mean that standard DFT and *ab initio* calculations on C_120_^−^ and its larger equivalents will be of limited value. These fulleride monoanion clusters pose a serious challenge to current computational methods in quantum chemistry and we hope that this study will stimulate alternative approaches to try and understand their behaviour.

## Conflicts of interest

There are no conflicts of interest to declare.

## Supplementary Material

CP-027-D5CP03392F-s001

## Data Availability

The data supporting this article have been included as part of the supplementary information (SI). Supplementary information: method benchmarking *via* DFT calculations on C_60_^−^ and C_120_^−^. Electronic and vibrational spectra of all calculated C_120_^−^ isomers, Cartesian coordinates and electronic energies of all calculated structures of C_120_^−^. See DOI: https://doi.org/10.1039/d5cp03392f.
