# Supplementary material for: Spectroscopy of C120− and larger fulleride cluster monoanions in the mid-infrared
Source: Phys Chem Chem Phys. 2025 Sep 17;27(39):21150–6. doi: 10.1039/d5cp03392f (PMC12455574; doi:10.1039/d5cp03392f)
Supplement: CP-027-D5CP03392F-s001 [file CP-027-D5CP03392F-s001.pdf]

Supplementary Information (SI) for Physical Chemistry Chemical Physics.
This journal is © the Owner Societies 2025

Supplemental Material:

Spectroscopy of C_{120}^{-} and larger fulleride cluster monoanions in the mid-infrared

Miriam Kappe,[1] Gabriel Schöpfer,[1] Arne Schiller,[1,2] Elisabeth Gruber,[1] Milan Ončák,[1,*] Andrew M. Ellis[3,*], Paul Scheier[1]

[1] Institut für Ionenphysik und Angewandte Physik, Universität Innsbruck, Technikerstr. 25, A-6020 Innsbruck, Austria

[2] Institute for Breath Research, Universität Innsbruck, Innrain 66, A-6020 Innsbruck, Austria

[3] School of Chemistry, University of Leicester, University Road, Leicester, LE1 7RH, UK

*Corresponding authors: Email: milan.oncak@uibk.ac.at; andrew.ellis@le.ac.uk

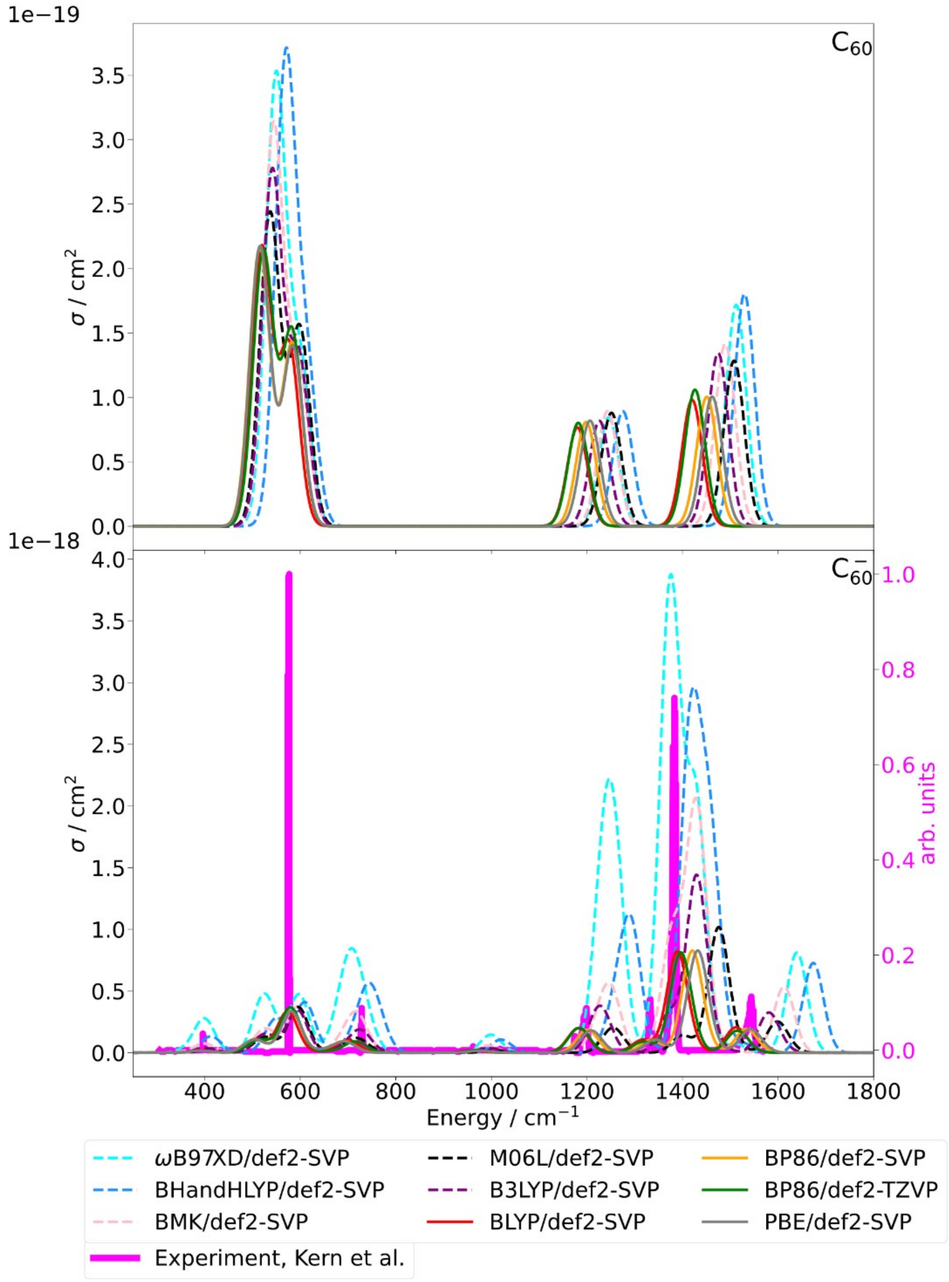

Figure S1. IR vibrational spectrum of C_{60} and C_{60}^{-} calculated with several quantum chemical methods. An empirical broadening of 50 cm^{-1} has been used. For comparison, an experimental spectrum of C_{60}^{-} from Kern et al. (*J. Phys. Chem. A* 2013, *117*, 8251) is also shown, with an arbitrarily scaled intensity, digitalized by hand with the help of Chemcraft (https://chemcraftprog.com). Theoretical methods which show good agreement with the experimental C_{60}^{-} spectrum are shown in solid lines, whereas the other theoretical data is plotted in dashed lines.

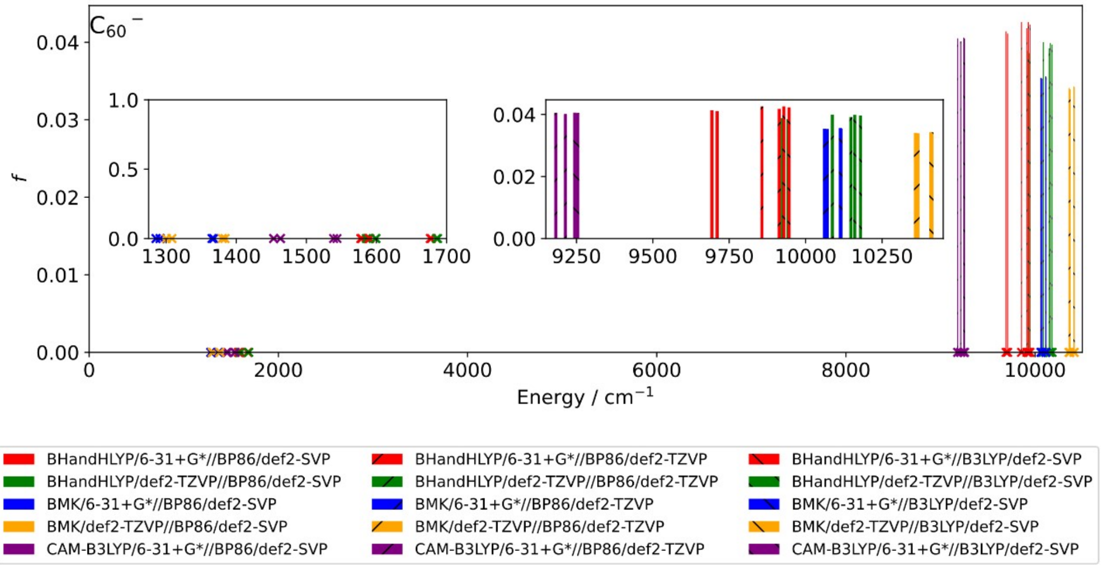

Figure S2. Electronic transitions in C_{60}^- as calculated through various quantum chemical methods. The two insets zoom into the regions in which electronic transitions appear.

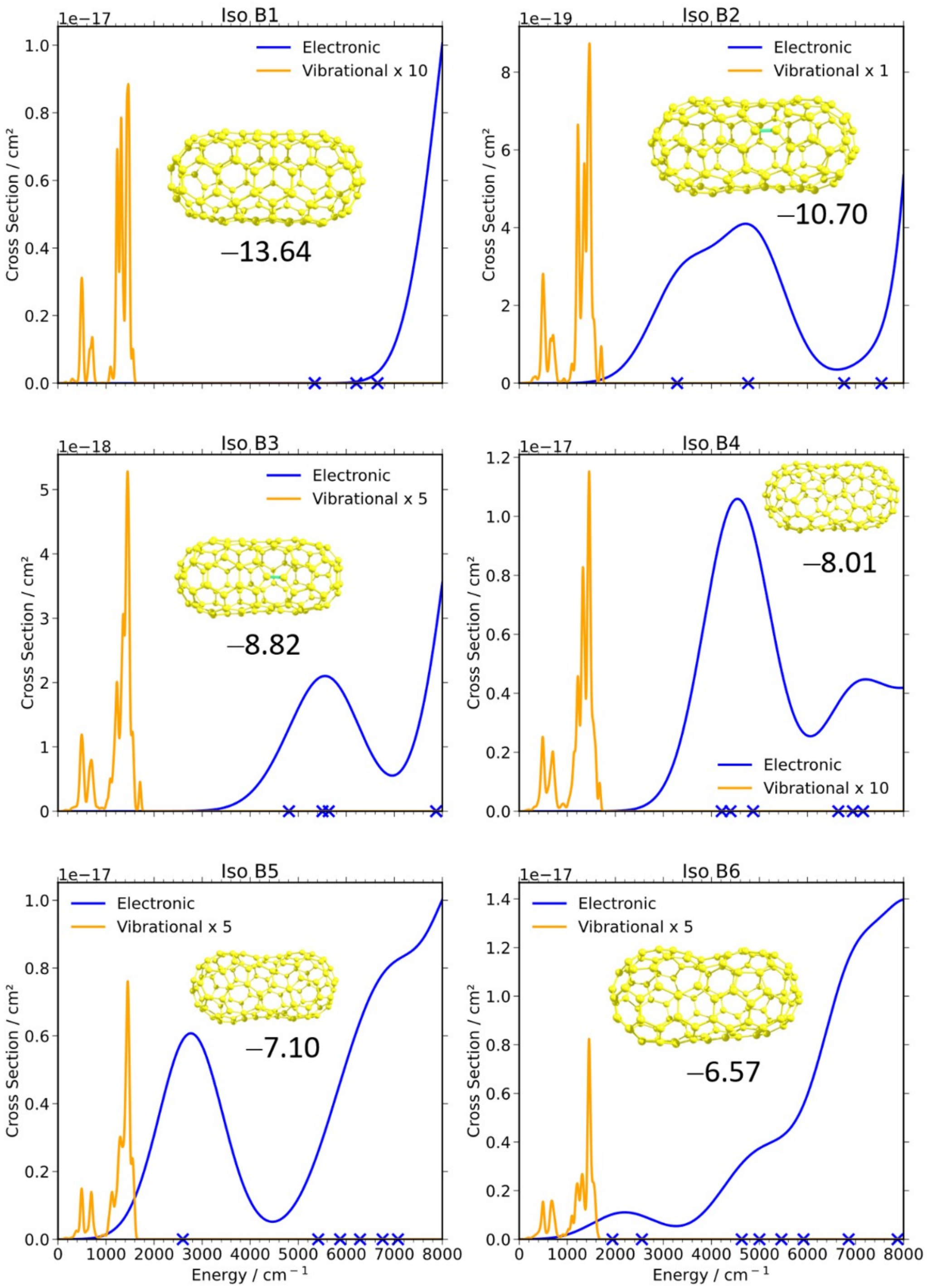
Iso B1
1e−17
Electronic
Vibrational x 10
−13.64
Cross Section / cm²
Iso B2
1e−19
Electronic
Vibrational x 1
−10.70
Cross Section / cm²
Iso B3
1e−18
Electronic
Vibrational x 5
−8.82
Cross Section / cm²
Iso B4
1e−17
Electronic
Vibrational x 10
−8.01
Cross Section / cm²
Iso B5
1e−17
Electronic
Vibrational x 5
−7.10
Cross Section / cm²
Energy / cm^{-1}
Iso B6
1e−17
Electronic
Vibrational x 5
−6.57
Cross Section / cm²
Energy / cm^{-1}

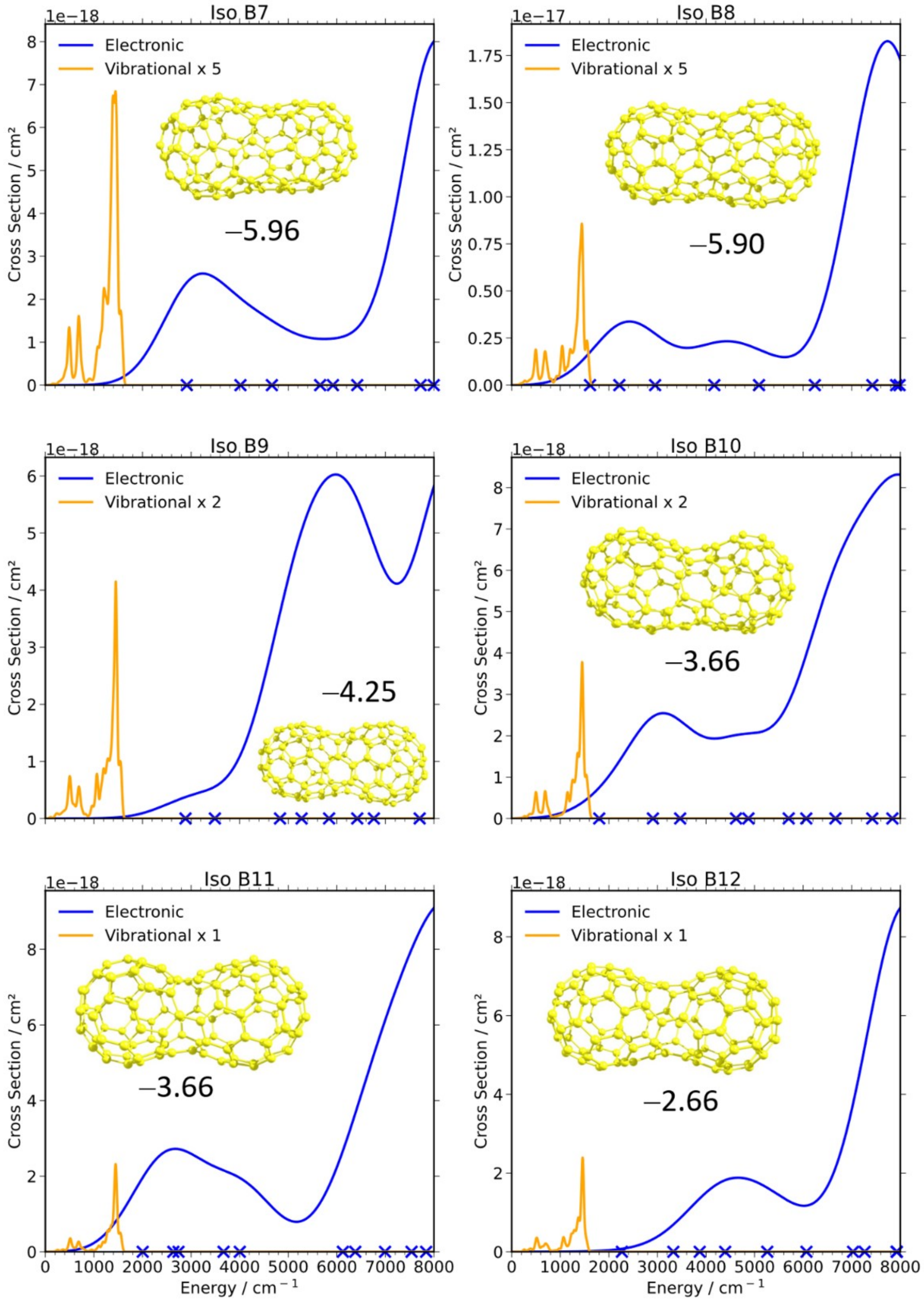
Iso B7
1e−18
Electronic
Vibrational x 5
−5.96
Iso B8
1e−17
Electronic
Vibrational x 5
−5.90
Iso B9
1e−18
Electronic
Vibrational x 2
−4.25
Iso B10
1e−18
Electronic
Vibrational x 2
−3.66
Iso B11
1e−18
Electronic
Vibrational x 1
−3.66
Iso B12
1e−18
Electronic
Vibrational x 1
−2.66
Cross Section / cm²
Energy / cm⁻¹

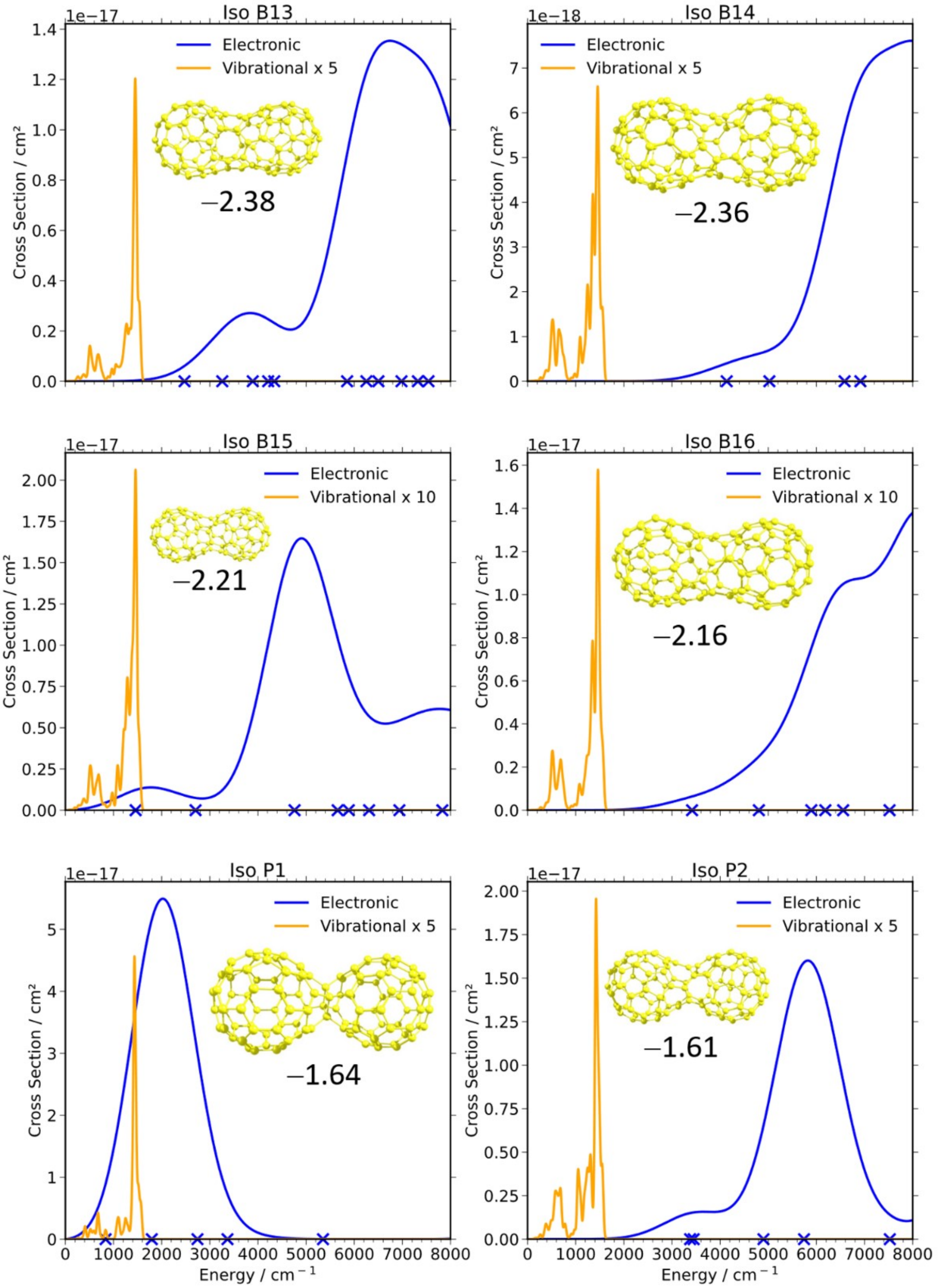
Iso B13
1e−17
Electronic
Vibrational x 5
−2.38
Cross Section / cm²
Iso B14
1e−18
Electronic
Vibrational x 5
−2.36
Cross Section / cm²
Iso B15
1e−17
Electronic
Vibrational x 10
−2.21
Cross Section / cm²
Iso B16
1e−17
Electronic
Vibrational x 10
−2.16
Cross Section / cm²
Iso P1
1e−17
Electronic
Vibrational x 5
−1.64
Cross Section / cm²
Energy / cm⁻¹
Iso P2
1e−17
Electronic
Vibrational x 5
−1.61
Cross Section / cm²
Energy / cm⁻¹

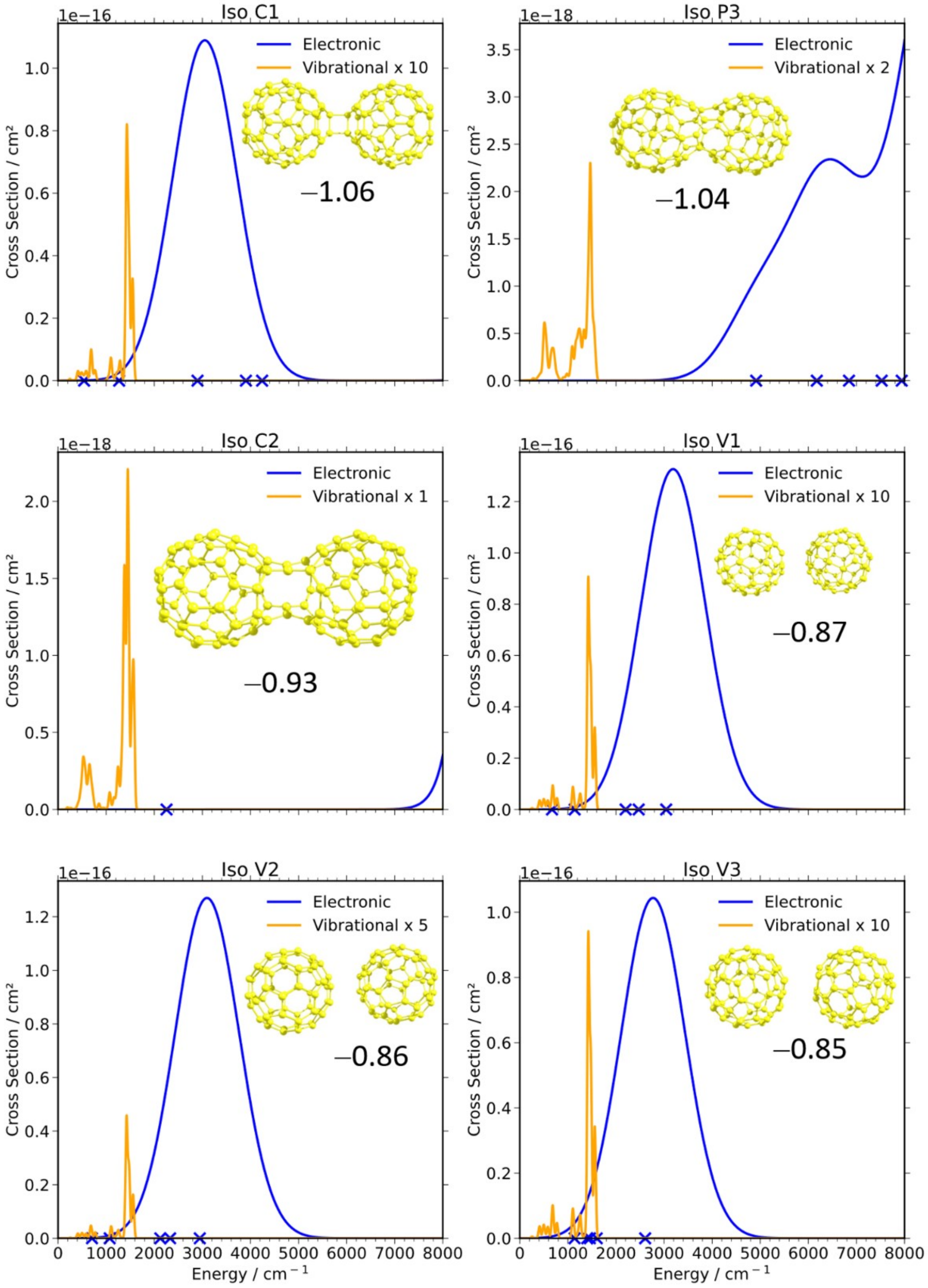

Iso C1
1e−16
Electronic
Vibrational x 10
−1.06
Cross Section / cm²
Iso P3
1e−18
Electronic
Vibrational x 2
−1.04
Iso C2
1e−18
Electronic
Vibrational x 1
−0.93
Iso V1
1e−16
Electronic
Vibrational x 10
−0.87
Iso V2
1e−16
Electronic
Vibrational x 5
−0.86
Iso V3
1e−16
Electronic
Vibrational x 10
−0.85
Energy / cm⁻¹

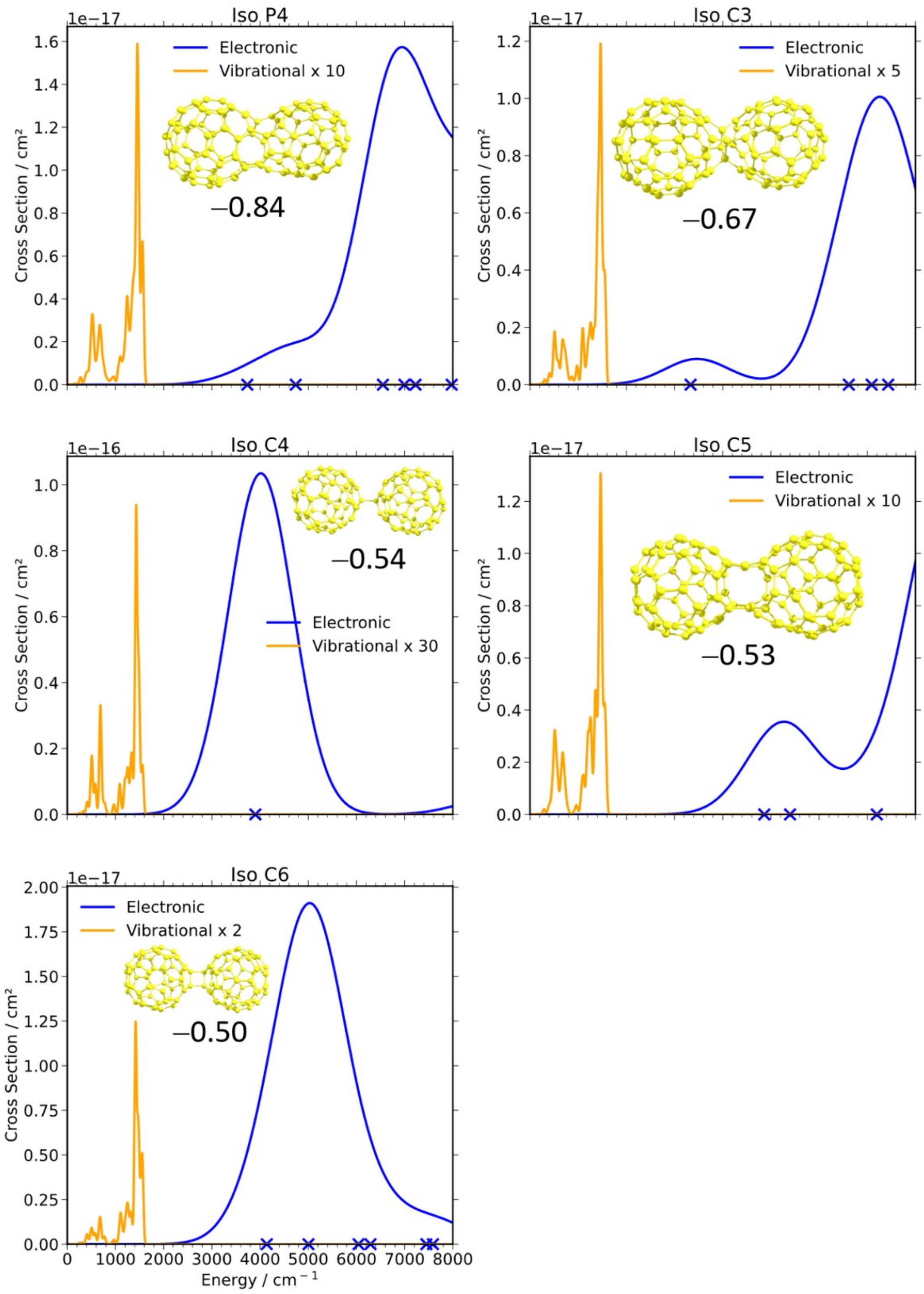

Figure S3. IR vibrational spectra of all 29 considered isomers of C_{120}^{-} calculated with BP86/def2-SVP, and electronic transitions calculated with the time-dependent DFT BMK/6-

31+G* method. An empirical broadening of 50 cm^{-1} and 1600 cm^{-1} has been used for the vibrational and the electronic spectra, respectively. In addition, every electronic excitation is marked with a blue “x”. Note the multiplication factors for the vibrational spectra, showing that the intensities of the vibrational transitions are significantly lower than the electronic transitions. Energies for the reaction $C_{60} + C_{60}^{-} \rightarrow C_{120}^{-}$ are given in eV inside the plot for each isomer, determining also the order in which the isomers are shown.

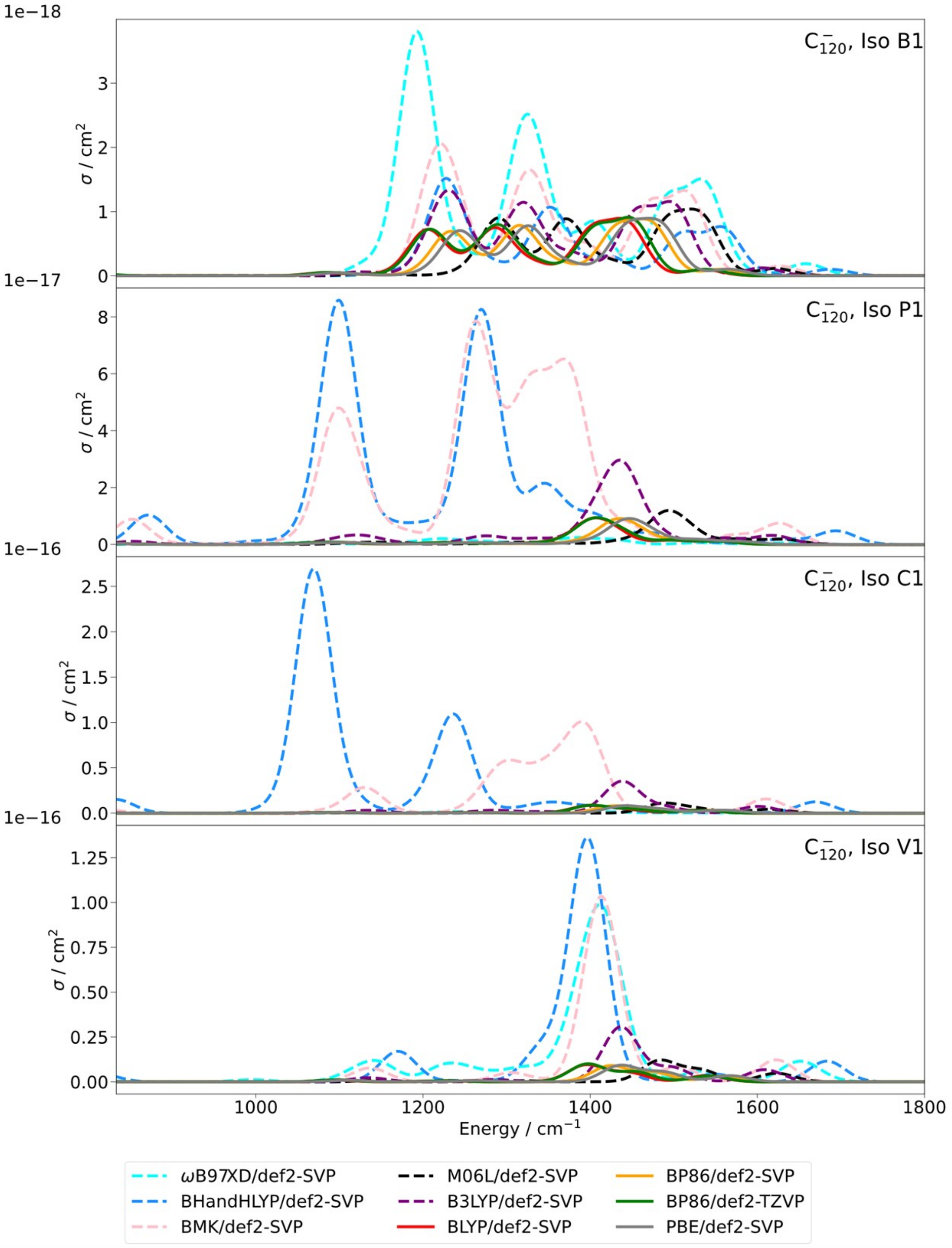

Figure S4. Vibrational spectra of isomers B1, P1, C1 and V1 of C_{120}^- as calculated through various quantum chemical methods. The intensities from BHandHLYP/def2-SVP were divided by 2 for better visibility.

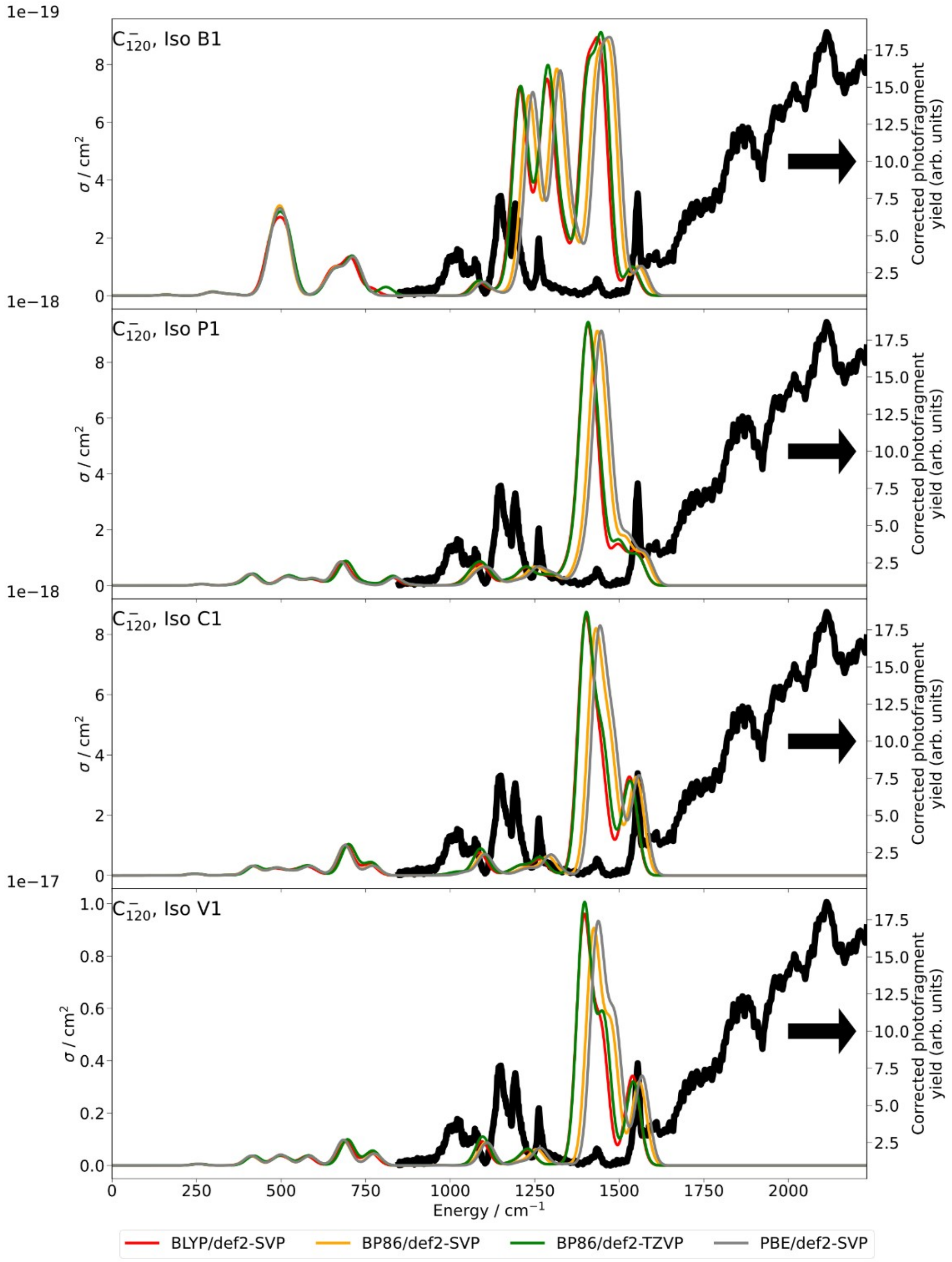

Figure S5. Vibrational spectra with cross sections σ of isomers B1, P1, C1 and V1 of C_{120}^{-} as calculated through various quantum chemical methods. Shown are only the most promising methods according to the comparison of the calculated vibrational spectra of C_{60}^{-} with literature. The experimental spectrum of C_{120}^{-} from Figure 1 with corrected photofragment yield in arbitrary units is added in black for comparison.

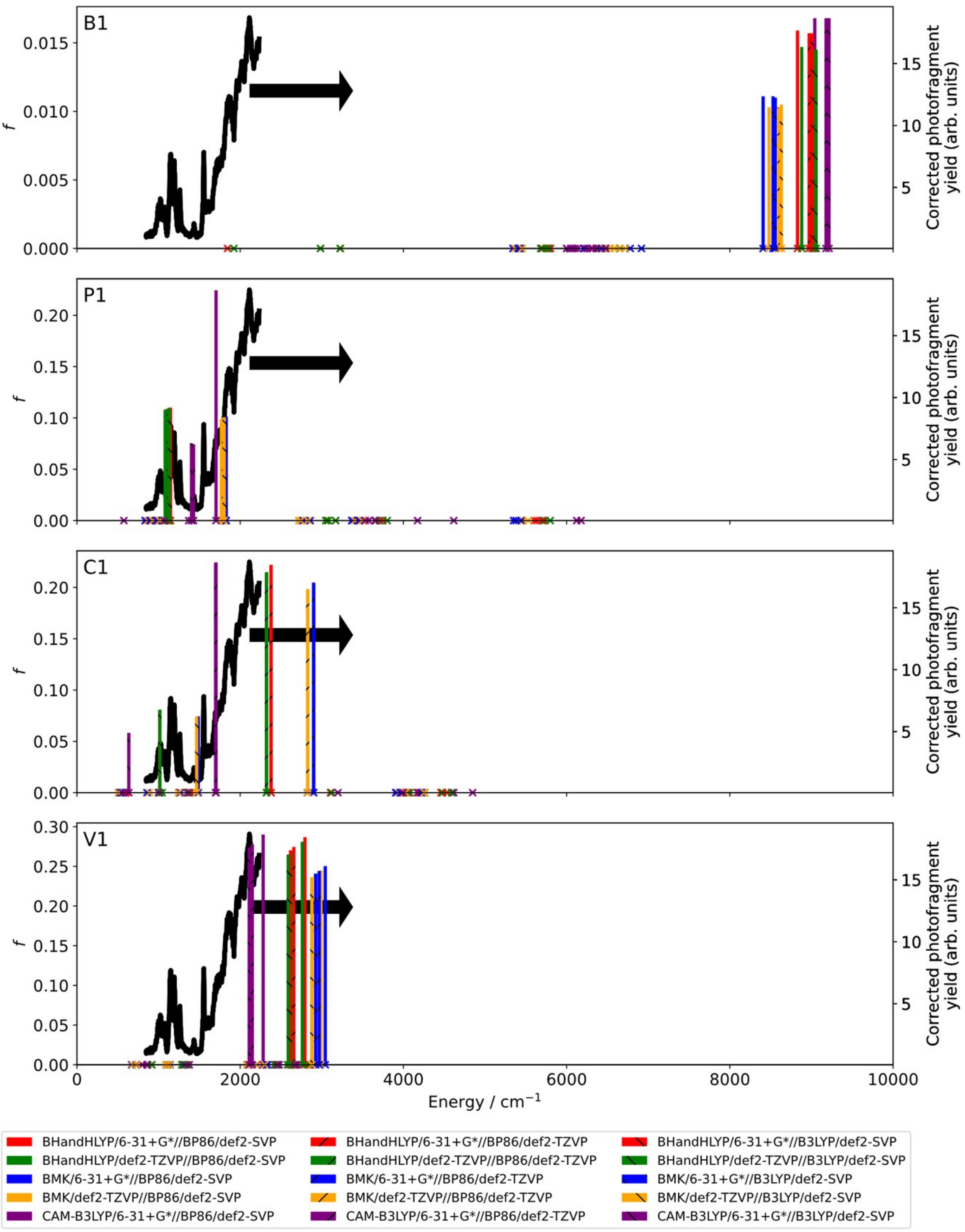

Figure S6. Electronic transitions in isomers B1, P1, C1 and V1 of C_{120}^{-} as calculated through various quantum chemical methods. Five lowest electronically excited states were considered for benchmarking purposes. The experimental spectrum of C_{120}^{-} from Figure 1 with corrected photofragment yield in arbitrary units is added in black for comparison.

Table S1. Electron attachment energies of C_{60} in eV, i.e., energies for the reaction $C_{60} + e^{-} \rightarrow C_{60}^{-}$, calculated at various levels of theory.

B3LYP/ def2-SVP	BHandHLYP/ def2-SVP	BLYP/ def2-SVP	BMK/ def2-SVP	BP86/ def2-SVP	BP86/ def2-TZVP	M06L/ def2-SVP	PBE/ def2-SVP	wB97XD/ def2-SVP
−2.64	−2.36	−2.51	−2.65	−2.92	−2.97	−2.73	−2.82	−2.48

Table S2. Binding energies (in eV) of four isomers B1, P1, C1 and V1 of C_{120}^{-}, i.e., energies for the reaction $C_{60} + C_{60}^{-} \rightarrow C_{120}^{-}$, calculated at various levels of theory. In isomer V1 bound by van der Waals forces, we were unable to obtain a true minimum, and imaginary frequencies were obtained even after several attempts to modify the structures (marked in the table).

	B3LYP/ def2-SVP	BHandHLYP/ def2-SVP	BLYP/ def2-SVP	BMK/ def2-SVP	BP86/ def2-SVP	BP86/ def2-TZVP	M06L/ def2-SVP	PBE/ def2-SVP	ωB97XD/ def2-SVP
B1	−14.32	−14.64	−13.70	−14.42	−13.64	−13.18	−13.95	−13.61	−14.13
P1	−1.26	−0.64	−1.19	−1.91	−1.64	−1.24	−1.08	−1.48	−1.68
C1	−0.74	−0.26	−0.67	−1.35	−1.06	−0.77	−0.64	−0.96	−1.19
V1	−0.70	−0.23[a]	−0.82	−0.67[b]	−0.87	−0.78	−0.78	−0.80	−0.52[c]

[a] – Imaginary frequency of 14*i* present. [b] – Imaginary frequency of 5*i* present. [c] – Imaginary frequency of 6*i* present.

Cartesian coordinates of structures (in Å) along with the zero-point corrected electronic energy at the BP86-D3/def2-SVP level of theory (in Hartree)

C60-
E = -2284.521068
C -0.277656 -3.372279 1.120287
C -0.131989 -1.621199 -3.169868
C 0.340195 -2.662034 -2.343925
C 1.692958 -2.589676 -1.784872
C 2.515982 -1.477459 -2.073817
C 2.029030 -0.405820 -2.929842
C 0.718262 -0.478185 -3.473159
C -0.535844 -3.270830 -1.351982
C 0.265793 -3.550196 -0.169426
C 1.646590 -3.137812 -0.439109
C -1.872610 -2.809300 -1.209779
C -2.433034 -2.620636 0.120466
C -1.656993 -2.897938 1.268140
C 0.525447 -2.787056 2.184115
C -0.348019 -1.930460 2.974852
C -1.700834 -2.007048 2.416296
C -3.280357 -1.429075 0.094675
C -3.322512 -0.565401 1.207084
C -2.519843 -0.855328 2.394019
C -2.029030 0.405820 2.929842
C -0.718262 0.478185 3.473159
C 0.128002 -0.707093 3.489961
C 1.508970 -0.293270 3.223982
C 2.354540 -1.121180 2.450466
C 1.863587 -2.385150 1.923111
C 2.423957 -2.563905 0.591618
C -3.233919 -0.878179 -1.256191
C -2.359523 -1.732602 -2.058549
C -1.511419 -1.146561 -3.026104
C -1.508970 0.293270 -3.223982
C -2.354540 1.121180 -2.450466
C -3.231400 0.517594 -1.447831
C -3.319902 0.880815 1.008467
C -3.275106 1.412267 -0.295543
C -2.515982 1.477459 2.073817
C -2.423957 2.563905 -0.591618
C -1.646590 3.137812 0.439109
C -1.692958 2.589676 1.784872
C 0.131989 1.621199 3.169868
C -0.340195 2.662034 2.343925
C 1.511419 1.146561 3.026104
C 0.535844 3.270830 1.351982
C 1.872610 2.809300 1.209779
C 2.359523 1.732602 2.058549
C 3.231400 -0.517594 1.447831
C 3.233919 0.878179 1.256191
C 3.275106 -1.412267 0.295543
C 3.319902 -0.880815 -1.008467
C 3.280357 1.429075 -0.094675
C 3.322512 0.565401 -1.207084
C 2.519843 0.855328 -2.394019
C 1.700834 2.007048 -2.416296
C 1.656993 2.897938 -1.268140
C 2.433034 2.620636 -0.120466
C 0.348019 1.930460 -2.974852
C -0.525447 2.787056 -2.184115
C 0.277656 3.372279 -1.120287
C -0.265793 3.550196 0.169426
C -1.863587 2.385150 -1.923111
C -0.128002 0.707093 -3.489961

Iso b1
E = -4569.436157
C -2.581946 2.323240 4.320091
C -2.595182 1.581585 5.565428
C -3.029179 0.245881 5.565428
C -3.454405 -0.361910 4.320091
C -3.454405 0.361910 -4.320091
C -3.007397 1.737656 -4.320091
C -1.431756 1.970643 6.350682
C -2.316630 -0.752719 6.350682
C -3.007397 -1.737656 4.320091
C -3.029179 -0.245881 -5.565428
C -2.306132 1.979428 -5.565428
C -1.411667 3.173497 4.320091
C -2.595182 -1.581585 -5.565428
C -2.581946 -2.323240 -4.320091
C -1.431756 -1.970643 -6.350682
C -1.411667 -3.173497 -4.320091
C -0.729705 1.004353 7.105056
C -2.306132 -1.979428 5.565428
C -0.702221 -2.956901 -5.565428
C -1.169914 -2.804939 5.565428
C -1.180688 -0.383629 7.105056
C -2.316630 0.752719 -6.350682
C -1.169914 2.804939 -5.565428
C 0.729705 1.004353 7.105056
C -0.723273 3.397170 -4.320091
C -0.702221 2.956901 5.565428
C 0.000000 2.435849 -6.350682
C 0.723273 3.397170 -4.320091
C 0.702221 2.956901 5.565428
C -0.723273 -3.397170 4.320091
C -1.180688 0.383629 -7.105056
C -0.000000 -1.241449 7.105056
C 1.169914 2.804939 -5.565428
C 1.411667 3.173497 4.320091
C 1.431756 1.970643 6.350682
C 2.595182 1.581585 5.565428
C 1.180688 -0.383629 7.105056
C -0.000000 -2.435849 6.350682
C 1.169914 -2.804939 5.565428
C 2.581946 2.323240 4.320091
C 3.007397 1.737656 -4.320091
C 3.454405 0.361910 -4.320091
C 2.306132 1.979428 -5.565428
C -0.729705 -1.004353 -7.105056
C 2.306132 -1.979428 5.565428
C 0.000000 1.241449 -7.105056
C 1.180688 0.383629 -7.105056
C 2.316630 -0.752719 6.350682
C 0.723273 -3.397170 4.320091
C 1.411667 -3.173497 -4.320091
C 3.029179 0.245881 5.565428
C 3.454405 -0.361910 4.320091
C 2.316630 0.752719 -6.350682
C 3.029179 -0.245881 -5.565428
C 3.007397 -1.737656 4.320091
C 0.729705 -1.004353 -7.105056
C 0.702221 -2.956901 -5.565428
C 2.581946 -2.323240 -4.320091
C 1.431756 -1.970643 -6.350682
C 2.595182 -1.581585 -5.565428
C 1.436085 3.216410 -3.103836
C 0.720563 3.405329 -1.861361
C -0.720563 3.405329 -1.861361
C -1.436085 3.216410 -3.103836
C 2.615213 2.359723 -3.103836
C -2.615213 2.359723 -3.103836
C 0.728741 3.446240 3.103836
C -0.728741 3.446240 3.103836
C 3.052376 1.758021 3.103836
C 2.584550 2.331432 1.861361
C 1.418654 3.178505 1.861361
C -1.418654 3.178505 1.861361
C -2.584550 2.331432 1.861361
C -3.052376 1.758021 3.103836
C 3.502763 0.371873 3.103836
C 2.615213 -2.359723 3.103836
C 3.015994 -1.737601 1.861361
C 3.461327 -0.367008 1.861361
C 3.052376 -1.758021 -3.103836
C 3.502763 -0.371873 -3.103836
C 3.461327 0.367008 -1.861361
C 3.015994 1.737601 -1.861361
C 1.436085 -3.216410 3.103836
C 0.728741 -3.446240 -3.103836
C 1.418654 -3.178505 -1.861361
C 2.584550 -2.331432 -1.861361
C -0.728741 -3.446240 -3.103836
C -3.052376 -1.758021 -3.103836
C -2.584550 -2.331432 -1.861361
C -1.418654 -3.178505 -1.861361
C -2.615213 -2.359723 3.103836
C -1.436085 -3.216410 3.103836
C -3.502763 -0.371873 -3.103836
C -3.015994 1.737601 -1.861361
C -3.461327 0.367008 -1.861361
C -3.502763 0.371873 3.103836
C -0.720563 -3.405329 1.861361
C 0.720563 -3.405329 1.861361
C -1.407866 3.165343 -0.618905
C -2.575365 2.317105 -0.618905
C 2.575365 2.317105 -0.618905
C 1.407866 3.165343 -0.618905
C 2.999529 -1.733293 -0.618905
C 3.445474 -0.360816 -0.618905
C -0.721554 -3.388339 -0.618905
C 0.721554 -3.388339 -0.618905
C -3.445474 -0.360816 -0.618905
C -2.999529 -1.733293 -0.618905
C -3.461327 -0.367008 1.861361
C -3.015994 -1.737601 1.861361
C 0.721554 3.388339 0.618905
C -0.721554 3.388339 0.618905
C 3.445474 0.360816 0.618905
C 2.999529 1.733293 0.618905
C 1.407866 -3.165343 0.618905
C 2.575365 -2.317105 0.618905
C -2.575365 -2.317105 0.618905
C -1.407866 -3.165343 0.618905
C -2.999529 1.733293 0.618905
C -3.445474 0.360816 0.618905

Iso b2
E = -4569.328087
C -6.407215 0.682107 -2.308332
C -5.579713 -0.292616 -3.004285
C -5.675271 1.939120 -2.299178
C -4.397369 1.731980 -2.963801
C -4.338928 0.352656 -3.389711
C -3.213275 2.412011 -2.562202
C -1.906897 1.813367 -2.857629
C -1.870449 0.435163 -3.291053
C -3.099335 -0.336539 -3.400892
C -5.561313 -1.630496 -2.582101
C -4.304269 -2.344150 -2.563200
C -3.091375 -1.737551 -2.992749
C -6.353251 -2.048055 -1.431665
C -7.128565 -1.100221 -0.731058
C -7.168026 0.286324 -1.182166
C -4.308138 -3.222918 -1.410641
C -5.557825 -3.026764 -0.703068
C -3.097189 -3.503822 -0.729376
C -1.855983 -3.218091 -1.412125

C -1.853191 -2.313628 -2.533064
C 5.595495 0.203609 -2.986159
C 4.312421 -0.348634 -3.368204
C 6.369681 -0.825687 -2.304293
C 5.554245 -2.029300 -2.294686
C 4.299530 -1.737578 -2.961700
C 3.114064 0.424198 -3.336408
C 1.857599 -0.287412 -3.260360
C 1.851032 -1.683698 -2.912498
C 3.086214 -2.353766 -2.572459
C 5.555605 -2.874065 -1.170752
C 4.309045 -3.460038 -0.724648
C 3.092843 -3.260384 -1.431163
C 5.664295 1.535911 -2.565965
C 4.455818 2.341726 -2.534266
C 3.165368 1.815677 -2.896009
C -7.209685 1.140563 0.000009
C -6.522091 2.369877 0.000019
C -5.756733 2.781757 -1.171850
C -5.557825 -3.026776 0.703019
C -6.353252 -2.048079 1.431632
C -7.128566 -1.100232 0.731040
C -4.308139 -3.222941 1.410589
C -3.097189 -3.503834 0.729320
C 6.350623 -2.523654 -0.000020
C 7.133532 -1.349590 -0.000011
C 7.156073 -0.490400 -1.179639
C 7.219490 0.895686 -0.730349
C 6.515176 1.899251 -1.432940
C -4.574106 3.474019 -0.732770
C -3.350414 3.352727 -1.454785
C 4.309045 -3.460050 0.724592
C 5.555605 -2.874084 1.170705
C 3.092843 -3.260409 1.431111
C 1.854362 -3.467721 0.719255
C 1.854362 -3.467708 -0.719309
C 5.554246 -2.029338 2.294653
C 6.369682 -0.825725 2.304280
C 7.156074 -0.490419 1.179632
C 4.299531 -1.737627 2.961673
C 3.086215 -2.353809 2.572422
C -7.168026 0.286304 1.182171
C -4.304270 -2.344192 2.563162
C -5.561314 -1.630538 2.582075
C 4.611739 3.262865 -1.432933
C 5.837792 2.961972 -0.708867
C -5.756732 2.781739 1.171895
C -5.675271 1.939082 2.299209
C -6.407216 0.682070 2.308344
C 3.455951 3.617333 -0.729041
C 2.153464 3.253123 -1.193790
C 1.937564 2.398376 -2.325426
C 5.837793 2.961960 0.708915
C 6.515176 1.899228 1.432971
C 7.219489 0.895674 0.730364
C 4.611737 3.262837 1.432984
C 3.455951 3.617317 0.729097
C -4.574108 3.474009 0.732826
C -3.091376 -1.737601 2.992723
C -1.853191 -2.313672 2.533029
C -1.855984 -3.218115 1.412074
C -5.579714 -0.292665 3.004281
C -3.350412 3.352698 1.454835
C -2.164613 3.619381 0.728138
C -2.164611 3.619405 -0.728087
C -3.213273 2.411966 2.562240
C -4.397369 1.731930 2.963828
C -4.338928 0.352599 3.389718
C -3.099336 -0.336596 3.400889
C 5.664295 1.535868 2.565990
C 4.455817 2.341682 2.534304
C 3.165368 1.815627 2.896038
C 1.937564 2.398333 2.325461
C 2.153464 3.253093 1.193837
C 5.595496 0.203560 2.986163
C 4.312421 -0.348690 3.368199
C 3.114064 0.424142 3.336417
C 1.857599 -0.287468 3.260358
C 1.851033 -1.683748 2.912473
C -1.870449 0.435107 3.291064
C -1.906897 1.813317 2.857660
C 1.315741 3.258061 0.000023
C 0.625922 1.812477 -2.666753
C -0.668457 2.393306 -2.319528
C -0.875835 3.266517 -1.191253
C -0.039859 3.255387 0.000024
C 0.620002 0.455152 -3.162963
C -0.875836 3.266474 1.191294
C -0.668457 2.393261 2.319565
C -0.618615 -0.272792 -3.245607
C -0.616501 -1.673946 -2.894280
C -0.618616 -0.272848 3.245607
C -0.616502 -1.673997 2.894257
C 0.625922 1.812426 2.666780
C 0.620002 0.455097 3.162974
C 0.612868 -2.288411 2.509229
C 0.614210 -3.208289 1.399078
C 0.614210 -3.208265 -1.399128
C 0.612869 -2.288368 -2.509263
C -0.619146 -3.455240 0.721593
C -0.619146 -3.455229 -0.721648

Iso b3
E = -4569.258948
C -6.311584 -0.739039 2.342872
C -5.557696 0.333973 2.962885
C -5.481594 -1.931729 2.404750
C -4.208535 -1.586079 3.034842
C -4.254735 -0.190458 3.365670
C -2.968232 -2.186221 2.664134
C -1.682352 -1.499967 2.920316
C -1.742223 -0.089754 3.315268
C -3.068029 0.585198 3.350232
C -5.668568 1.638238 2.462918
C -4.481226 2.448472 2.408896
C -3.236711 1.967696 2.885607
C -6.480844 1.919168 1.285262
C -7.179845 0.872286 0.654431
C -7.116118 -0.478121 1.203214
C -4.527360 3.242316 1.191802
C -5.746155 2.897006 0.491616
C -3.330484 3.563977 0.495218
C -2.091175 3.455230 1.219602
C -2.078391 2.661479 2.421448
C 5.424214 -0.184029 2.964625
C 4.058329 0.257349 3.291799
C 6.131804 0.900102 2.312692
C 5.229084 2.031953 2.272174
C 3.964983 1.661796 2.870583
C 3.017028 -0.713412 3.359768
C 0.845980 0.661823 3.333061
C 1.450465 1.941606 2.900365
C 2.765453 2.336613 2.491782
C 5.298376 2.939223 1.197770
C 4.068815 3.502233 0.707856
C 2.838476 3.294495 1.367216
C 5.709129 -1.509113 2.589112
C 4.638438 -2.471001 2.576854
C 3.334496 -2.074631 2.970067
C -7.110887 -1.412331 0.084320
C -6.346598 -2.592654 0.166139
C -5.538324 -2.859167 1.351084
C -5.724692 2.793240 -0.911099
C -6.449526 1.713319 -1.567679
C -7.171953 0.771727 -0.804287
C -4.477357 3.005878 -1.620001
C -3.293262 3.414962 -0.954834
C 6.165535 2.664174 0.063661
C 7.007076 1.533928 0.074113
C 7.019836 0.655249 1.240607
C 7.221143 -0.711958 0.778198
C 6.601610 -1.778394 1.457032
C -4.311549 -3.491101 0.945072
C -3.089043 -3.218749 1.632634
C 4.131770 3.554260 -0.740390
C 5.409464 3.010538 -1.138276
C 2.944615 3.328582 -1.491526
C 1.685071 3.545345 -0.832160
C 1.646321 3.610812 0.604063
C 5.474659 2.172208 -2.265880
C 6.343264 1.007304 -2.260849
C 7.118335 0.699582 -1.115461
C 4.243833 1.826940 -2.958166
C 2.997190 2.400893 -2.610353
C -7.123155 -0.643108 -1.156287
C -4.407206 2.044743 -2.700636
C -5.624736 1.264379 -2.682185
C 4.837488 -3.329400 1.430600
C 6.020646 -2.884776 0.706909
C -5.569919 -3.040401 -0.984099
C -5.542342 -2.278823 -2.169994
C -6.344149 -1.067319 -2.258813
C 3.689286 -3.716404 0.722751
C 2.371037 -3.413664 1.210848
C 2.135241 -2.643841 2.381032
C 6.008604 -2.838482 -0.708746
C 6.618677 -1.719263 -1.408408
C 7.252695 -0.685868 -0.683138
C 4.791766 -3.182981 -1.433858
C 3.661853 -3.626715 -0.737332
C -4.340445 -3.622915 -0.510365
C -3.156143 1.480861 -3.076805
C -1.952456 2.145573 -2.635966
C -2.018968 3.132232 -1.585867
C -5.570742 -0.096015 -3.018143
C -3.142438 -3.499239 -1.268321
C -1.944501 -3.633848 -0.535768
C -1.918735 -3.476552 0.899916
C -3.061741 -2.641952 -2.444691
C -4.281988 -2.046242 -2.864569
C -4.295037 -0.692420 -3.366095
C -3.091245 0.062063 -3.412300
C 5.755641 -1.384209 -2.538049
C 4.586876 -2.244842 -2.512835
C 3.273318 -1.772427 -2.863084
C 2.079846 -2.409425 -2.286566
C 2.338662 -3.288067 -1.174204
C 5.627581 -0.054222 -2.952389
C 4.322803 0.437552 -3.347120
C 3.157585 -0.385480 -3.312090
C 1.870564 0.270305 -3.255039
C 1.791793 1.674733 -2.945801
C -1.823283 -0.636805 -3.271877
C -1.790271 -1.997453 -2.789885
C 1.531042 -3.326686 0.035634
C 0.969246 -1.801234 2.743965
C -0.415343 -2.128436 2.401988
C -0.632202 -3.052788 1.317640
C 0.185722 -3.184030 0.113599
C 1.545474 -0.572997 3.274935
C -0.678932 -3.272152 -1.049796
C -0.520863 -2.488928 -2.237528
C -0.607096 0.818141 3.323588
C -0.847802 2.163988 2.883907
C -0.606557 0.136369 -3.242687
C -0.678318 1.547727 -2.945122
C 0.747560 -1.869059 -2.616857
C 0.669763 -0.528015 -3.143917
C 0.520433 2.237772 -2.574785
C 0.460257 3.227852 -1.526006
C 0.386002 3.513248 1.260116
C 0.363958 2.759443 2.471712
C -0.802434 3.487857 -0.898051
C -0.842118 3.669377 0.534264

Iso b4
E = -4569.229209
C 6.119226 0.671435 2.373708
C 5.497078 -0.507176 2.917420
C 5.159636 1.759898 2.477578
C 3.891697 1.271599 3.004619
C 4.127649 -0.153750 3.325648
C 2.633747 1.907085 2.668325
C 1.222474 1.416664 2.892949
C 1.683721 -1.069022 3.337224
C 3.164746 -1.159847 3.394741
C 5.819215 -1.772097 2.369833
C 4.774787 -2.752274 2.286809
C 3.539744 -2.477807 2.889092
C 6.625803 -1.858157 1.158827
C 7.214636 -0.701343 0.619620
C 7.007458 0.586553 1.270125
C 4.803205 -3.358756 0.966037
C 5.965182 -2.821598 0.280101
C 3.583874 -3.632073 0.277554
C 2.350255 -3.628860 1.047066
C 2.365197 -3.078054 2.358189
C -5.433498 0.010651 2.973847
C -4.031915 -0.303147 3.288716
C -6.019234 -1.128764 2.309645
C -4.998439 -2.161383 2.248943
C -3.753382 -1.671017 2.814469
C -3.102399 0.743863 3.378092
C -0.686832 -0.370054 3.184686
C -1.125945 -1.717511 2.755496
C -2.483200 -2.203225 2.396069
C -5.030504 -3.078964 1.184620
C -3.775249 -3.541851 0.683982
C -2.550681 -3.195448 1.307763
C -5.842687 1.310344 2.606657
C -4.866308 2.365905 2.608219
C -3.541730 2.086566 3.025275
C 6.981012 1.612289 0.234252
C 6.143134 2.728413 0.393561
C 5.260323 2.815321 1.548755
C 5.897189 -2.567105 -1.098077
C 6.548587 -1.393224 -1.665860
C 7.210830 -0.474366 -0.824882
C 4.641465 -2.774481 -1.797244
C 3.494056 -3.294545 -1.144461
C -5.946691 -2.899287 0.067324
C -6.869228 -1.835754 0.081030
C -6.940560 -0.962076 1.246924
C -7.260082 0.383319 0.784281
C -6.738885 1.499887 1.462684
C 4.044725 3.433553 1.153720
C 2.806951 3.072424 1.764032
C -3.874197 -3.651304 -0.761254
C -5.190033 -3.202018 -1.144000
C -2.727311 -3.368986 -1.547445
C -1.455044 -3.512047 -0.909115
C -1.377715 -3.503202 0.523703
C -5.322723 -2.372427 -2.276486
C -6.262549 -1.266307 -2.259228
C -7.052733 -1.016296 -1.109411
C -4.125072 -1.942464 -2.981994
C -2.844474 -2.445736 -2.661361
C 7.078066 0.958583 -1.063687
C 4.509458 -1.734126 -2.794393
C 5.692037 -0.901766 -2.733811
C -5.103348 3.189746 1.440790
C -6.240402 2.645863 0.709723
C 5.395915 3.256435 -0.745567
C 5.433665 2.590542 -1.991287
C 6.289776 1.427493 -2.147258
C -3.985004 3.667529 0.736939
C -2.649802 3.489870 1.248081
C -2.405508 2.760434 2.435275
C -6.203795 2.593129 -0.702557
C -6.727044 1.431221 -1.402776
C -7.287379 0.355243 -0.677738
C -5.005418 3.021392 -1.417519
C -3.920200 3.552901 -0.714971
C 4.135981 3.752539 -0.275313
C 3.233468 -1.200833 -3.121369
C 2.070780 -1.942394 -2.705590
C 2.200596 -2.991636 -1.721816
C 5.569789 0.472712 -2.976532
C 2.969457 3.671981 -1.086401
C 1.753602 3.782400 -0.395380
C 1.691185 3.546543 1.035398
C 2.940111 2.878587 -2.303324
C 4.192023 2.351473 -2.723342
C 4.267464 1.030434 -3.295667
C 3.096796 0.231140 -3.380195
C -5.831863 1.159238 -2.522785
C -4.721148 2.095515 -2.485366
C -3.371885 1.711549 -2.814664
C -2.222109 2.422425 -2.222376
C -2.560177 3.301303 -1.124006
C -5.618510 -0.155244 -2.945785
C -4.285602 -0.553776 -3.346261
C -3.172204 0.341531 -3.284866
C -1.846185 -0.230839 -3.239010
C -1.687327 -1.639896 -2.986298
C 1.796448 0.865867 -3.207956
C 1.691926 2.206150 -2.683068
C -1.772576 3.445597 0.090123
C -1.209813 1.978638 2.770994
C 0.141208 2.274437 2.435391
C 0.381071 3.242852 1.398029
C -0.412554 3.379570 0.188600
C -1.629142 0.709103 3.241401
C 0.494513 3.451050 -0.953532
C 0.390654 2.642352 -2.136277
C 0.772908 0.029144 3.237986
C 1.233679 -2.328773 2.836166
C 0.618843 0.036013 -3.211749
C 0.769469 -1.381142 -2.978994
C -0.848693 1.962228 -2.537390
C -0.692603 0.632113 -3.089657
C -0.388059 -2.146307 -2.647522
C -0.265284 -3.156428 -1.627411
C -0.120133 -3.393667 1.151172
C -0.055582 -2.582361 2.346586
C 1.005354 -3.409470 -1.030296
C 1.074804 -3.648172 0.387713

Iso b5
E = -4569.195544
C 1.456573 1.226152 -3.125110
C 1.620861 -0.184793 -3.354041
C 0.151424 1.726637 -2.660967
C -1.097093 0.946297 -2.946292
C -0.863954 -0.473576 -3.093880
C 0.481272 -1.023856 -3.087505
C 2.673742 2.007844 -2.913879
C 3.939216 1.477468 -3.312390
C 4.075717 0.067174 -3.567397
C 2.946776 -0.783881 -3.402931
C -1.963716 -1.410010 -2.934707
C -1.708669 -2.636517 -2.228374
C -0.374502 -2.930097 -1.808291
C 0.725318 -2.285115 -2.447215
C 0.238221 2.674472 -1.594035
C 1.521687 3.013474 -1.014542
C 2.699744 2.955661 -1.817379
C -2.500889 1.421883 -2.856944
C -3.599258 0.467871 -3.138948
C -3.321312 -0.967662 -3.106558
C -0.864828 3.098350 -0.759752
C -2.188538 3.357487 -1.183988
C -2.921199 2.677867 -2.236648
C -0.642047 3.023795 0.632320
C 0.574822 2.581238 1.195627
C 1.688345 3.005614 0.413136
C 3.924124 3.342641 -1.203471
C 4.039258 3.469069 0.229815
C 2.940875 3.101839 1.056938
C 5.201370 1.943948 -2.747235
C 5.202691 2.888398 -1.707894
C -3.133261 3.866891 -0.211540
C -2.900740 3.684558 1.173402
C -1.670290 3.102559 1.567087
C -4.328351 2.971269 -1.979080
C -4.466841 3.683586 -0.715991
C -4.980435 0.845657 -2.973221
C -5.345792 2.093829 -2.358298
C 0.626307 1.765412 2.346889
C 1.992057 1.556262 2.847099
C 3.101223 2.402694 2.327367
C -1.807133 2.116210 2.592931
C -0.717518 1.168776 2.796965
C 5.422247 -0.336505 -3.185518
C 6.136011 0.827336 -2.685940
C 3.170884 -2.069633 -2.750623
C 4.481663 -2.435036 -2.330749
C 5.626637 -1.584574 -2.578406
C 2.062432 -2.676162 -2.067648
C 6.126416 2.769294 -0.587065
C 5.390403 3.146866 0.613620
C 7.035005 0.701304 -1.597118
C 7.035278 1.694390 -0.528777
C 5.588575 2.482763 1.830356
C 4.459685 2.142818 2.700362
C -3.905991 3.187758 2.060273
C -3.272062 2.108986 2.848160
C -5.517569 3.396499 0.179772
C -5.235343 3.135168 1.591450
C -0.922738 -0.268587 3.000148
C 0.270383 -1.166410 2.890685
C 1.614229 -0.900753 3.297137
C 2.354414 0.361222 3.531190
C -4.064080 1.009487 3.176320
C -3.600288 -0.407048 3.201270
C -2.272474 -0.951547 2.957392
C 3.753411 0.043230 3.731144
C 4.796450 0.932785 3.392793
C 7.262778 1.009431 0.741743
C 6.576656 1.417638 1.911468
C 6.067337 0.437953 2.862703
C -2.787883 -3.315465 -1.550316
C -4.107255 -3.040347 -1.957609
C -4.370851 -1.895958 -2.799402
C -0.157537 -3.425036 -0.484033
C -1.279412 -3.460385 0.400378
C -2.568573 -3.688486 -0.163607
C 2.277222 -3.293461 -0.781574
C 1.136911 -3.394640 0.084893
C -5.724008 -1.469443 -2.527220
C -6.022816 -0.108388 -2.627478
C 6.564950 -1.728044 -1.472492
C 7.263452 -0.601667 -0.984230
C 7.412526 -0.412867 0.455899
C 3.595440 -3.362027 -0.200517
C 4.693241 -3.090972 -1.059647
C 5.981866 -2.676750 -0.538810
C -5.246307 -3.234341 -1.072889
C -5.012808 -3.526512 0.288247
C -3.651011 -3.683870 0.738109
C -6.268032 -2.270611 -1.440256
C -6.938037 0.507135 -1.676022
C -7.404099 -0.239908 -0.567710
C -7.054292 -1.651444 -0.438507
C -6.517729 1.887660 -1.504790
C -6.775490 -1.917797 0.964823
C -5.753983 -2.821537 1.324946
C -3.506982 -2.973813 1.999291
C -2.306490 -2.295562 2.341942
C -1.142877 -2.795517 1.659580

C -4.789832 -2.458905 2.350866
C 0.139942 -2.375692 2.093381
C 1.274970 -2.995949 1.455009
C 3.755451 -3.186147 1.243044
C 2.572918 -2.830240 2.009247
C 2.656561 -1.889315 3.072091
C -4.805002 -1.186637 2.947844
C 6.137882 -2.507659 0.847174
C 5.030627 -2.783402 1.742637
C 5.112570 -1.841247 2.842036
C 3.937888 -1.362989 3.448175
C 6.884730 -1.362502 1.359945
C -6.580606 2.489777 -0.229986
C 6.228312 -0.935006 2.588011
C -5.493838 1.040330 2.835387
C -6.072022 2.053835 2.026052
C -6.966738 1.691527 0.930234
C -5.940012 -0.317668 2.674339
C -7.375007 0.356530 0.762742
C -6.903535 -0.664636 1.693559

Iso b6
E = -4569.176187
C 6.344604 1.108658 2.118281
C 5.705380 0.057024 2.887331
C 5.461085 2.256377 2.103437
C 4.223356 1.898669 2.815813
C 4.380577 0.518800 3.309667
C 2.995135 2.471882 2.453808
C 1.549567 2.029002 2.569555
C 1.822840 -0.276174 3.449418
C 3.325401 -0.394736 3.499376
C 5.954325 -1.289434 2.535394
C 4.866046 -2.215880 2.651284
C 3.649763 -1.791901 3.206744
C 6.708156 -1.615473 1.333995
C 7.293500 -0.590623 0.565373
C 7.144971 0.800018 0.986631
C 4.859991 -3.087414 1.480114
C 6.004941 -2.713411 0.672655
C 3.622483 -3.470258 0.892461
C 2.409171 -3.280792 1.670143
C 2.452016 -2.455838 2.828299
C -5.427708 0.643820 2.929541
C -4.003837 0.464307 3.274699
C -5.974730 -0.635386 2.542054
C -4.915764 -1.615425 2.679132
C -3.671931 -0.959922 3.071490
C -3.104695 1.539836 3.134110
C -0.593959 0.456723 3.057068
C -1.021585 -0.938928 2.934641
C -2.393508 -1.530446 2.742202
C -4.946568 -2.756853 1.862202
C -3.689738 -3.288720 1.450796
C -2.467640 -2.756974 1.930027
C -5.930524 1.808006 2.302609
C -5.009760 2.883277 2.059626
C -3.681393 2.759304 2.519953
C 7.057261 1.622185 -0.213752
C 6.238314 2.763694 -0.218104
C 5.482452 3.116399 0.977048
C 5.902275 -2.727901 -0.729358
C 6.532041 -1.686130 -1.527760
C 7.230451 -0.632745 -0.890355
C 4.627976 -3.060830 -1.338708
C 3.501280 -3.445581 -0.566764
C -5.896804 -2.877030 0.764706
C -6.848335 -1.860108 0.558443
C -6.927548 -0.746908 1.497718
C -7.321981 0.443512 0.754631
C -6.834553 1.700281 1.159367
C 4.231686 3.665352 0.557631
C 3.084959 3.418053 1.328134
C -3.823252 -3.745913 0.075773
C -5.160227 -3.429380 -0.364040
C -2.707732 -3.640412 -0.787133
C -1.422600 -3.608339 -0.162789
C -1.312050 -3.231764 1.210268
C -5.332570 -2.879376 -1.653259
C -6.296274 -1.813713 -1.861304
C -7.077511 -1.337058 -0.781214
C -4.158365 -2.587508 -2.459782
C -2.864057 -2.990302 -2.074867
C 7.071485 0.732688 -1.373854
C 4.462113 -2.232696 -2.514344
C 5.638247 -1.393667 -2.633934
C -5.285228 3.422247 0.728385
C -6.393378 2.668715 0.160956
C 5.381326 3.047366 -1.369326
C 5.349455 2.170615 -2.464765
C 6.222688 1.001738 -2.472373
C -4.220644 3.816165 -0.104043
C -2.887252 3.815148 0.420322
C -2.645954 3.332726 1.726791
C -6.349218 2.301776 -1.204017
C -6.831442 0.993277 -1.618066
C -7.352297 0.091682 -0.661065
C -5.168598 2.603260 -2.011750
C -4.118345 3.353870 -1.486346
C 4.129551 3.594217 -0.891457
C 3.173775 -1.774039 -2.893019
C 2.031498 -2.439427 -2.326033
C 2.196264 -3.274173 -1.162730
C 5.483583 -0.078484 -3.099697
C 2.886896 3.294853 -1.532509
C 1.684998 3.390783 -0.749286
C 1.848882 3.420940 0.668945
C 2.831393 2.356975 -2.645279
C 4.076179 1.803901 -3.069303
C 4.160456 0.413303 -3.446770
C 3.003223 -0.403994 -3.378728
C -5.929484 0.505865 -2.653059
C -4.844730 1.455285 -2.827499
C -3.480545 1.044951 -3.045821
C -2.346963 1.901548 -2.623753
C -2.728839 3.047961 -1.802003
C -5.690266 -0.866365 -2.788381
C -4.352669 -1.304320 -3.100250
C -3.256027 -0.388248 -3.226051
C -1.920146 -0.910582 -3.086587
C -1.729516 -2.250459 -2.585811
C 1.703464 0.233576 -3.269316
C 1.590326 1.624491 -2.910782
C -1.979690 3.459424 -0.642058
C -1.440006 2.625303 2.003440
C 0.927214 2.717674 1.506193
C -0.396855 2.805239 1.065563
C -0.663889 3.116627 -0.290696
C -1.628034 1.527072 2.886410
C 0.411053 2.975173 -1.265550
C 0.296161 2.108005 -2.397780
C 0.918733 0.793779 3.127258
C 1.344715 -1.606241 3.171849
C 0.533786 -0.597794 -3.124460
C 0.716139 -1.950403 -2.668144
C -0.966980 1.397604 -2.766297
C -0.785743 -0.007556 -3.088942
C -0.420871 -2.664274 -2.185679
C -0.256561 -3.434987 -0.978704
C -0.044055 -2.969370 1.764282
C 0.050403 -1.907104 2.741145
C 1.021330 -3.542576 -0.371237
C 1.124091 -3.428656 1.061558

Iso b7
E = -4569.153683
C 0.630504 -0.523240 -3.268346
C 1.081071 0.871028 -3.231453
C -0.849484 -0.530039 -3.136137
C -1.298724 0.823615 -3.072543
C -0.091317 1.647581 -2.891737
C 1.335518 2.956883 -1.412515
C 0.020302 2.677411 -1.923451
C 2.485150 2.622596 -2.203350
C 2.411466 1.439992 -3.069003
C -1.070621 3.174232 -1.119511
C -2.424314 3.305197 -1.532956
C -3.146243 2.492706 -2.494162
C -2.686288 1.210466 -3.004570
C 1.534204 3.173689 -0.012011
C 0.433353 2.871221 0.848952
C -0.810831 3.223276 0.278933
C 3.672026 0.812015 -3.366664
C 3.919281 -0.636238 -3.427643
C 2.971767 -1.664085 -3.191985
C 1.491404 -1.634661 -3.075781
C 3.715976 3.121260 -1.717734
C 4.972153 2.592703 -2.169078
C 4.927965 1.435343 -2.968358
C 2.811287 3.334555 0.567175
C 3.878019 3.551416 -0.344600
C -1.841456 3.294918 1.217809
C -3.118480 3.765739 0.803033
C -3.390126 3.826661 -0.582859
C 0.544116 2.155471 2.074750
C -0.757908 1.546846 2.614054
C -1.905785 2.398053 2.327699
C 3.015757 2.832769 1.914735
C 1.932623 2.034509 2.557927
C 5.244448 3.323097 0.039365
C 5.946967 2.737481 -1.098645
C 4.389033 2.665410 2.287896
C 5.492961 2.890055 1.348982
C -1.809091 -1.584397 -2.812442
C -1.467312 -2.704287 -1.959168
C -0.107940 -2.983445 -1.490997
C 1.109384 -2.727284 -2.178710
C -3.671434 0.140152 -3.171809
C -3.229906 -1.252642 -2.993584
C 5.946865 0.419135 -2.768571
C 5.327371 -0.857908 -3.046988
C 6.906104 1.733192 -0.876495
C 6.916841 0.559145 -1.742076
C 3.431881 -2.793792 -2.405997
C 2.328272 -3.263239 -1.630126
C 5.739161 -1.981167 -2.293313
C 4.732486 -2.932348 -1.902876
C -2.527181 -3.367379 -1.220932
C -3.869781 -3.233212 -1.638483
C -4.215978 -2.203221 -2.577529
C -5.610130 -1.855176 -2.338126
C -6.028931 -0.544512 -2.565841
C -5.067716 0.439864 -3.022666
C -5.000272 -3.431224 -0.734992
C -6.091912 -2.585990 -1.180441
C -2.324447 -3.622073 0.195569
C -3.402662 -3.623530 1.098424
C -4.767944 -3.593646 0.644992
C 0.066860 -3.329075 -0.104910
C -1.065947 -3.286163 0.752433
C 1.335049 -3.328760 0.551678
C 2.509117 -3.519047 -0.235135
C -0.973767 -2.527291 1.952296
C 0.282329 -2.022640 2.358939
C 1.435087 -2.714043 1.842646
C -3.310828 -2.803111 2.297492
C -2.158697 -2.025004 2.578375
C 3.824777 -3.417524 0.366634
C 4.941330 -3.285525 -0.512896
C 2.720159 -2.441650 2.393014
C 3.933178 -2.905662 1.734906
C 7.274430 -0.589108 -0.920318
C 6.678150 -1.836305 -1.189392
C 6.171237 -2.647114 -0.086176
C -4.562926 2.708807 -2.233277

C -5.506559 1.717532 -2.513777
C -4.723001 3.521551 -1.038813
C 6.522660 1.889190 1.587591
C 7.204094 1.307702 0.487453
C 7.430039 -0.132280 0.457417
C 2.356447 0.958710 3.391120
C 3.767775 0.727914 3.630053
C 4.776521 1.590565 3.153793
C -0.886684 0.128534 2.996634
C 0.345221 -0.719339 2.986384
C 1.677552 -0.351758 3.346728
C 6.064854 1.065307 2.696860
C -4.078707 3.290880 1.749972
C -3.370960 2.330470 2.617013
C -5.737211 3.238445 -0.100260
C -5.415847 3.121672 1.319993
C -2.195648 -0.624690 3.049243
C -4.097058 1.230484 3.072530
C -3.554290 -0.144635 3.246116
C -6.977838 0.095817 -1.662301
C -7.378048 -0.573679 -0.483681
C -6.928599 -1.938556 -0.231510
C -6.651426 1.511538 -1.624869
C -6.635119 -2.053958 1.187453
C -5.557344 -2.853637 1.621842
C -4.622603 -2.339618 2.606550
C -6.739802 2.228195 -0.411147
C 2.754186 -1.329361 3.283361
C 4.008193 -0.697651 3.576895
C 5.208285 -1.223201 3.069097
C 6.285067 -0.326388 2.661362
C -6.178861 2.038841 1.866700
C -5.530686 1.143177 2.756380
C -4.708353 -1.017053 3.081420
C -5.894808 -0.248859 2.734315
C -7.059933 1.516470 0.823416
C 5.180565 -2.351682 2.160279
C 6.291083 -2.196007 1.237658
C 6.963313 -0.932276 1.525472
C -6.837973 -0.749117 1.800715
C -7.379540 0.149015 0.785191

Iso b8
E = -4569.151672
C 0.099387 0.010288 -3.275333
C -0.011034 -1.333494 -2.798114
C -1.066575 0.848331 -3.248347
C -2.384949 0.257046 -3.350967
C -2.448128 -1.188054 -3.097307
C -1.307427 -1.835319 -2.497157
C 1.090836 -2.076281 -2.244230
C 0.858460 -2.707731 -0.975743
C -0.455104 -2.782483 -0.401297
C -1.519167 -2.709996 -1.365688
C -3.677489 -1.860570 -2.967820
C -3.866788 -2.905501 -1.973231
C -2.836282 -3.155811 -1.029367
C -0.626726 2.119503 -2.652155
C 0.842620 2.071822 -2.539934
C 1.296456 0.837968 -3.094906
C -3.643837 0.952727 -3.358032
C -4.890168 0.213591 -3.331408
C -4.925762 -1.189408 -3.172759
C -1.492691 2.978721 -1.936474
C -2.960544 3.047789 -2.016943
C -3.904350 2.267412 -2.735997
C 3.220778 2.633225 -2.043736
C 1.796196 2.817471 -1.728723
C 4.192726 3.245564 -1.194093
C 3.828204 3.620665 0.149422
C 2.489572 3.457759 0.566114
C 1.443765 3.294264 -0.415431
C -1.114732 3.429892 -0.582437
C 0.089186 3.248204 0.133397
C -0.089009 2.741678 1.470603
C 1.049753 2.191854 2.129093
C 2.286241 2.847881 1.869634
C -2.325466 3.589887 0.163991
C -2.512964 3.079244 1.487628
C -1.356229 2.452991 2.051322
C -3.424058 3.605158 -0.754228
C -5.307587 2.271252 -2.308954
C -5.919328 1.011142 -2.688452
C -4.724597 3.490774 -0.269494
C -5.732981 2.878743 -1.100251
C 2.678890 0.476179 -3.213187
C 3.127311 -0.892370 -3.320749
C 2.413679 -2.036758 -2.770327
C 3.667098 1.505531 -2.873962
C -5.924377 -1.827224 -2.331341
C -5.251800 -2.896356 -1.592057
C -6.905783 0.398574 -1.880003
C -6.905630 -1.051537 -1.689777
C -3.150650 -3.417762 0.361062
C -2.133860 -3.058757 1.368194
C -0.754358 -2.760510 1.015734
C -5.587895 -3.168334 -0.255998
C -4.537661 -3.451130 0.717403
C 1.961640 -3.237690 -0.251542
C 3.162544 -3.568524 -0.982010
C 3.383310 -2.947308 -2.235055
C 0.141444 -2.013934 1.977955
C 1.575428 -2.112045 2.020724
C 2.266012 -3.061491 1.144055
C 5.589524 2.840197 -1.163807
C 6.017046 1.818049 -2.014007
C 5.057992 1.172815 -2.893684
C 4.953216 3.342037 1.027281
C 6.062270 2.878708 0.212360
C 3.365560 2.437389 2.655661
C 4.726612 2.748437 2.286765
C 5.495835 -0.196858 -3.062799
C 4.541731 -1.206938 -3.247487
C 4.715501 -2.484710 -2.572707
C -2.624684 -2.593768 2.623123
C -1.936828 -1.443605 3.187313
C -0.636415 -1.067557 2.823590
C -4.990891 -2.947415 1.997302
C -4.025328 -2.465851 2.901951
C 3.676830 -3.368215 1.261579
C 4.238929 -3.715307 -0.044825
C 2.497965 -1.215059 2.824644
C 3.872604 -1.608032 2.957257
C 4.473098 -2.701698 2.186155
C -0.350642 0.354477 2.909821
C 0.967714 0.906104 2.739684
C 2.221205 0.218903 3.061035
C 5.553418 -3.329097 -0.390770
C 5.789636 -2.697333 -1.690240
C -4.948255 3.105083 1.112626
C -3.845781 2.744066 1.947732
C -1.478181 1.275843 2.873804
C -3.994773 1.634464 2.889166
C -2.805622 0.865179 3.268330
C 3.326448 1.112355 3.261232
C -7.263929 -1.334908 -0.304426
C -6.633161 -2.393098 0.395801
C -6.240925 -2.227184 1.789904
C -6.686200 2.221418 -0.227696
C -7.292159 1.007631 -0.613997
C -7.491282 -0.057338 0.361499
C -6.194079 2.372920 1.144067
C -2.955172 -0.506719 3.607614
C 4.693639 0.678989 3.361575
C 5.575987 1.676925 2.763692
C 4.959029 -0.684451 3.226859
C 6.680038 1.256684 1.991766
C 6.936174 1.875084 0.697597
C -6.359282 1.339395 2.075173
C -5.273997 0.996994 2.979990
C -5.386604 -0.422806 3.260480
C -4.232887 -1.165153 3.532521
C -7.061940 0.114784 1.699843
C 6.657808 -0.432951 -2.212032
C 6.787549 -1.651940 -1.509020
C 6.980182 0.824676 -1.553741
C 7.406902 0.831135 -0.207469
C -6.449213 -0.981874 2.426400
C 5.855507 -2.381404 1.875000
C 6.386799 -2.663882 0.587087
C 7.176703 -1.643746 -0.102083
C 6.161422 -1.118718 2.521992
C 6.988858 -0.165621 1.886372
C 7.482946 -0.424258 0.535833

Iso b9
E = -4569.090792
C -1.690092 -3.058509 0.557885
C -0.335008 -2.548766 0.728292
C -2.660303 -2.822247 1.635736
C -2.427043 -1.689752 2.530212
C -1.232856 -0.907107 2.368970
C -0.114650 -1.596950 1.750749
C 0.866926 -2.910278 -0.101870
C 2.039074 -3.078505 0.706570
C 2.077277 -2.462994 2.011519
C 1.191192 -1.399516 2.314035
C -1.235395 0.513423 2.524831
C -0.120261 1.356293 2.039576
C 1.217121 1.094729 2.437045
C 1.704481 -0.208171 2.950916
C -2.189015 -3.485375 -0.706337
C -1.672331 -2.838019 -1.926477
C -0.412648 -2.251789 -2.159003
C 0.867157 -2.646030 -1.521850
C -3.510208 -1.080047 3.208154
C -3.539491 0.352665 3.360362
C -2.484186 1.163507 2.855322
C -4.012456 -3.265311 1.489766
C -5.118854 -2.617319 2.185435
C -4.870569 -1.551351 3.057595
C -3.603981 -3.697989 -0.885197
C -4.502481 -3.688765 0.200309
C -0.487563 -0.984907 -2.845040
C 0.722741 -0.256537 -2.931888
C 1.932804 -1.020070 -3.031304
C 2.092841 -2.244259 -2.251450
C -2.838169 -2.370116 -2.642699
C -2.934722 -1.032183 -3.138326
C -1.708595 -0.282457 -3.098012
C -4.002382 -3.032396 -2.114400
C -5.864123 -3.187225 0.039877
C -6.269742 -2.563862 1.293094
C -5.265044 -2.451299 -2.246763
C -6.254215 -2.591013 -1.181039
C 3.387505 -2.531869 2.569977
C 3.833228 -1.480299 3.390670
C 3.008060 -0.299048 3.530339
C 3.447239 -3.389639 0.396455
C 4.246438 -3.175273 1.614881
C 3.461033 -2.645306 -2.055388
C 4.089584 -3.251783 -0.847938
C -5.759345 -0.400792 3.107962
C -4.925825 0.773593 3.321609
C -7.108156 -1.424513 1.297011
C -6.864883 -0.332901 2.235002
C -2.806138 2.453280 2.251458
C -1.870726 2.959750 1.242616
C -0.524104 2.443747 1.126947
C -5.241427 1.997050 2.717951
C -4.181207 2.847973 2.202724
C 2.242203 2.100316 2.221852
C 3.524137 2.017205 2.904922
C 3.908107 0.833879 3.568594
C 0.235451 2.677869 -0.123749

C 1.659364 2.776696 -0.212678
C 2.438042 2.866309 1.012323
C 3.010097 -0.277510 -3.526450
C 4.340679 -0.790514 -3.501475
C 4.539722 -1.978804 -2.760249
C 0.745944 1.132497 -2.656342
C 2.040288 1.799252 -2.554199
C 3.057655 1.166326 -3.320000
C -1.722031 1.145772 -2.897986
C -0.512205 1.759674 -2.378286
C 5.521810 -2.952546 -0.871071
C 6.272498 -2.719378 0.315549
C 5.614501 -2.827071 1.588031
C 5.789688 -2.140531 -2.035647
C 6.108455 -1.759600 2.460216
C 5.233958 -1.089137 3.340822
C 5.283154 0.362592 3.468523
C -2.434482 3.662629 0.131073
C -1.896606 3.320616 -1.166680
C -0.640863 2.737033 -1.319708
C -4.722462 3.530778 1.034937
C -3.850856 3.853685 -0.014323
C 3.825292 3.281839 0.992851
C 4.507228 2.774416 2.171909
C 2.441000 2.718403 -1.483186
C 3.822702 3.090239 -1.457750
C 4.514023 3.406233 -0.216896
C 5.851196 2.342587 2.088051
C 6.242689 1.099610 2.736828
C -5.391935 -1.127199 -2.829134
C -4.226279 -0.361067 -3.160801
C -4.261475 1.094214 -2.986031
C -3.001273 1.818696 -2.788320
C -7.171717 0.928094 1.566713
C -6.399222 2.081615 1.830164
C -6.049164 3.000559 0.756868
C -7.072340 -1.391199 -1.181270
C -7.529588 -0.830627 0.032192
C -7.573493 0.618045 0.198403
C -6.553848 -0.508989 -2.226928
C -6.595880 0.879699 -2.066200
C -7.158237 1.465614 -0.853528
C -5.470888 1.691358 -2.498996
C -3.017586 3.027720 -2.036124
C 4.451127 1.520562 -3.243679
C 5.263945 0.318268 -3.397533
C 4.825804 2.519852 -2.340951
C 6.453790 0.178829 -2.655097
C 6.738928 -1.087620 -1.994882
C -4.213796 3.486679 -1.380694
C -5.438228 2.862207 -1.640946
C 7.100548 -0.999274 1.706891
C 7.167409 0.404411 1.847000
C 7.213764 -1.608829 0.382893
C 7.438216 -0.797578 -0.747291
C -6.416557 2.684798 -0.575258
C 5.909394 3.001501 -0.309553
C 6.558366 2.442187 0.821650
C 7.373090 1.245716 0.667229
C 6.093836 2.412744 -1.622343
C 6.892643 1.253865 -1.777035
C 7.523761 0.650311 -0.604087

Iso b10
E = -4569.069348
C -1.279225 -1.912354 -2.079113
C -0.364933 -0.850256 -2.438677
C -2.445040 -1.675154 -2.856766
C -2.439996 -0.293126 -3.328413
C -1.107545 0.251787 -3.035304
C 0.951941 2.881164 -1.260066
C 0.744200 1.975117 -2.357464
C -0.242854 3.298644 -0.550832
C -1.450153 3.360626 -1.245880
C -1.717131 2.570922 -2.420298
C -0.677296 1.629262 -2.759405
C -3.734141 0.274968 -3.532238
C -4.095989 1.637942 -3.142045
C -3.200368 2.571150 -2.569050
C 1.002285 -1.098569 -2.434782
C 1.905527 -0.192572 -3.086497
C 1.906865 1.228568 -2.893257
C -3.583694 -2.444599 -2.635136
C -4.880264 -1.908300 -2.960900
C -4.929936 -0.555512 -3.373005
C -1.325890 -2.659347 -0.869377
C -2.604739 -3.217295 -0.474725
C -3.662924 -3.247995 -1.442533
C 1.563014 -2.266381 -1.741702
C 1.112149 -2.571392 -0.384780
C -0.250177 -2.487504 0.113722
C 3.336620 1.618804 -2.984218
C 4.073761 2.537577 -2.189714
C 3.536569 3.212045 -0.989609
C 2.195620 3.176836 -0.519436
C 3.203938 -0.662699 -3.472196
C 4.098994 0.452584 -3.484997
C 2.831670 -2.724038 -2.183742
C 3.638498 -1.950176 -3.101975
C -0.625489 -2.273209 1.485791
C -1.985852 -2.619051 1.871699
C -2.936533 -3.243097 0.940514
C 2.133647 -2.997343 0.540076
C 2.340089 -2.495747 1.876647
C 1.586343 -1.388922 2.494546
C 0.184780 -1.345583 2.367444
C 3.727719 -3.430410 -1.308728
C 3.380732 -3.565160 0.048701
C -2.674275 3.632878 -0.551205
C -3.737420 3.366114 -1.466417
C -0.364504 3.121472 0.865860
C -1.619600 2.933061 1.519771
C -2.822579 3.360089 0.842169
C 2.042712 3.142060 0.925927
C 0.816959 2.691619 1.515490
C -5.057897 -3.280894 -1.063292
C -5.820040 -2.441893 -1.994652
C -4.327266 -3.327931 1.298691
C -5.384986 -3.318578 0.295404
C -6.020802 0.273134 -2.881156
C -5.508883 1.621787 -2.735070
C -6.865824 -1.633466 -1.507666
C -6.967781 -0.252454 -1.966479
C -5.987754 2.412181 -1.664104
C -5.037916 3.244119 -0.970349
C 0.798449 1.644428 2.463268
C -0.496407 1.112186 2.793707
C -1.679782 1.938794 2.563576
C 3.125810 3.084455 1.801628
C 3.141443 2.075301 2.857929
C 2.083205 1.118982 2.940420
C 4.640318 3.380121 -0.058584
C 4.454596 3.304470 1.339855
C -4.136769 3.007433 1.355261
C -5.249904 3.113046 0.460296
C -2.984374 1.506963 3.023427
C -4.221792 2.090694 2.490845
C -7.408875 0.555734 -0.832796
C -6.904006 1.861852 -0.681477
C -6.442482 2.309040 0.633947
C 5.037802 -2.183401 -2.803117
C 5.944385 -1.106835 -2.874425
C 5.469662 0.230034 -3.231189
C 5.100863 -3.125428 -1.686587
C 6.198842 1.191468 -2.447028
C 5.507578 2.329627 -1.960840
C 5.842595 2.817131 -0.635808
C -6.505838 -2.525021 0.794091
C -7.209062 -1.672132 -0.089507
C -7.546489 -0.316757 0.325632
C -2.533498 -1.986746 3.028442
C -3.942561 -1.920637 3.270017
C -4.843605 -2.635475 2.458799
C -0.680950 -0.302673 3.001358
C -1.952286 -0.705255 3.402424
C -6.147660 -2.072155 2.127733
C 4.382690 -3.422826 1.066134
C 3.729248 -2.735283 2.182362
C 6.080504 -2.969751 -0.686739
C 5.723280 -3.141771 0.720849
C 2.436806 -0.297724 3.099227
C 4.468838 -1.817373 2.937814
C 3.814672 -0.578058 3.365470
C 5.358931 2.545293 2.180055
C 4.524085 1.762792 3.087736
C 6.778668 2.143829 0.188606
C 6.507761 1.969379 1.611673
C 4.853443 0.435042 3.382372
C -3.044498 0.215486 3.616899
C 7.142896 0.472927 -1.600497
C 7.418439 0.925153 -0.294372
C 6.969321 -0.957630 -1.848261
C 7.039912 -1.874928 -0.775057
C -4.265222 -0.547773 3.653735
C -5.464476 0.037969 3.240893
C -6.466169 -0.750202 2.524144
C 6.477373 -2.180866 1.501936
C 5.856660 -1.549240 2.616665
C 6.092263 -0.136940 2.864669
C 7.291173 -1.396493 0.585292
C -5.451914 1.383872 2.698490
C -6.545356 1.463266 1.743594
C -7.150918 0.138206 1.607917
C 6.906487 0.617654 1.988061
C 7.498959 -0.018758 0.813834

Iso b11
E = -4569.069176
C 0.150554 -1.506939 1.800542
C 0.536313 -2.545593 0.840199
C 1.254261 -0.713760 2.367887
C 2.486445 -1.432833 2.643921
C 2.794560 -2.674155 1.930429
C 1.859323 -3.085907 0.886943
C -0.230882 -2.649887 -0.425321
C 0.635296 -2.519026 -1.616290
C 1.878016 -3.140944 -1.548062
C 2.406188 -3.673134 -0.304863
C 4.157765 -3.092917 1.845351
C 4.691574 -3.661641 0.609630
C 3.815237 -3.848938 -0.472981
C -1.190498 -1.276159 2.199974
C -2.189696 -2.305325 1.954474
C -2.386799 -3.000958 0.711395
C -1.626712 -2.767796 -0.506457
C 3.550951 -0.716108 3.249877
C 4.927080 -1.170678 3.188652
C 5.226676 -2.326796 2.455900
C 1.254504 0.725448 2.379448
C 2.485095 1.443149 2.659533
C 3.550850 0.724406 3.265151
C -1.697977 -0.007970 2.763059
C -1.209760 1.240433 2.161479
C 0.156056 1.494320 1.764802
C -2.415440 -2.531458 -1.741175
C -2.033468 -1.430331 -2.628688
C -0.759226 -0.705324 -2.593890
C 0.509140 -1.383397 -2.490469
C -3.758685 -3.436038 0.662399
C -4.455807 -3.435394 -0.556509
C -3.779358 -2.945360 -1.758388
C -3.451186 -2.309557 2.667890
C -4.433150 -3.029194 1.887837
C -2.969071 -0.011235 3.401485
C -3.836569 -1.176047 3.403871

C 0.536622 2.530694 0.838532
C 1.864219 3.077775 0.892690
C 2.794541 2.677925 1.941407
C -2.194012 2.274158 1.930916
C -2.374357 3.030456 0.722411
C -1.614877 2.809136 -0.509500
C -0.239027 2.674802 -0.435075
C -3.833416 1.150739 3.393079
C -3.454264 2.283334 2.662068
C 2.991775 -2.742950 -2.370565
C 4.181612 -3.308197 -1.783192
C 1.717059 -0.727485 -2.954008
C 2.984753 -1.442301 -2.959407
C 4.927238 1.170430 3.192688
C 5.787756 -0.002348 3.115911
C 4.161590 3.094432 1.850671
C 5.233943 2.326677 2.464930
C 6.386605 -2.339422 1.568006
C 6.023096 -3.126229 0.395627
C 6.902938 -0.001363 2.251310
C 7.184878 -1.184208 1.441678
C 6.402630 -2.662792 -0.892217
C 5.413411 -2.681929 -1.965312
C 7.603054 -0.728392 0.117897
C 7.167180 -1.438648 -1.024674
C 5.463081 -1.414697 -2.673600
C 4.262188 -0.732776 -3.072409
C 6.609956 -0.695817 -2.156011
C -0.759160 0.710858 -2.592692
C 0.510957 1.380617 -2.493576
C 1.717540 0.719369 -2.950780
C -3.059375 -0.725839 -3.305223
C -3.059731 0.737799 -3.300544
C -2.032473 1.441075 -2.620592
C -4.789168 -2.286525 -2.560169
C -4.434376 -1.159992 -3.316425
C 2.983103 1.436868 -2.957189
C 2.986317 2.737990 -2.366261
C 1.872105 3.139221 -1.553867
C 0.636861 2.516287 -1.624035
C -2.413980 2.543865 -1.740377
C 4.260729 0.730367 -3.069963
C -5.224529 -0.741082 3.387831
C -6.186652 -1.444804 2.624393
C -5.780512 -2.618722 1.858086
C -5.225414 0.717911 3.386809
C -6.501842 -2.615996 0.594379
C -5.849290 -3.043837 -0.595913
C -6.055798 -2.307158 -1.835457
C 6.392394 2.341122 1.579844
C 7.189592 1.181673 1.447362
C 7.602366 0.728078 0.123019
C 2.406566 3.664014 -0.295376
C 3.808536 3.849257 -0.461138
C 4.690194 3.666693 0.625684
C 6.022716 3.126612 0.408485
C -4.427929 3.021294 1.900791
C -3.748665 3.447692 0.679346
C -6.178018 1.426862 2.624412
C -5.776563 2.611404 1.869036
C -4.451994 3.449621 -0.539012
C -3.776486 2.952773 -1.745468
C -5.299254 0.006994 -3.344573
C -4.430580 1.172131 -3.309465
C -6.898498 -1.174599 -1.861105
C -6.508416 0.007709 -2.621685
C -4.786660 2.297281 -2.546411
C -7.355567 -1.440466 0.570831
C -7.549590 -0.724589 -0.631923
C -7.157842 -0.709747 1.825569
C -7.160262 0.700872 1.827514
C 4.174461 3.304387 -1.772374
C 5.402734 2.678759 -1.952954
C 6.395922 2.660756 -0.877831
C -6.500576 2.617786 0.610172
C -5.840462 3.045972 -0.577489
C -6.052208 2.318742 -1.823083
C -7.352539 1.439061 0.578882
C 5.458864 1.415653 -2.669225
C 6.606702 0.700324 -2.152808
C -6.895990 1.186159 -1.853318
C 7.161875 1.441406 -1.017530
C -7.551910 0.732081 -0.629504

Iso b12
E = -4569.032497
C -0.019621 0.728998 2.190773
C -0.413195 1.961111 1.582002
C -1.108116 -0.205525 2.513415
C -2.335382 0.358233 3.016210
C -2.674549 1.731680 2.656226
C -1.789193 2.392429 1.677863
C -4.037646 2.132821 2.784681
C -4.642311 3.048502 1.835736
C -3.857390 3.508186 0.760341
C -2.455873 3.169215 0.656741
C 0.538595 2.495623 0.528172
C 0.127221 2.217268 -0.805278
C -1.207490 2.119827 -1.324975
C -2.210099 2.949486 -0.772751
C 1.349607 0.337199 2.516635
C 2.374294 1.315350 2.755596
C 2.622593 2.532012 1.968415
C 1.915364 2.829689 0.742465
C -3.380679 -0.543652 3.374877
C -4.752885 -0.120208 3.512812
C -5.079617 1.210355 3.214874
C -1.141285 -1.522574 1.955616
C -2.354637 -2.294718 1.963750
C -3.386939 -1.888278 2.848267
C 1.785685 -1.068358 2.512991
C 1.248782 -1.921504 1.442803
C -0.096317 -1.942759 1.021728
C 2.758530 3.139552 -0.428328
C 2.364801 2.545678 -1.691238
C 1.111360 1.897167 -1.785409
C 4.034396 2.828686 2.085120
C 4.787413 3.335553 0.999722
C 4.134163 3.469569 -0.283876
C 3.636995 0.923640 3.357517
C 4.665277 1.863913 2.976177
C 3.044598 -1.425862 3.056916
C 3.966377 -0.428642 3.548623
C -0.541011 -2.396992 -0.273764
C -1.864340 -2.908665 -0.388570
C -2.707075 -3.089773 0.786472
C 2.167065 -2.775604 0.739751
C 2.303724 -2.871991 -0.696610
C 1.562182 -2.079505 -1.672282
C 0.157216 -1.935116 -1.499630
C 3.839579 -2.515897 2.520248
C 3.404244 -3.174131 1.355547
C -3.418857 3.135347 -1.497635
C -4.456090 3.539266 -0.575480
C -1.501036 1.196469 -2.402123
C -2.810252 1.215966 -2.953511
C -3.708183 2.284623 -2.588134
C 0.888868 0.694254 -2.551833
C -0.443236 0.179180 -2.626743
C 3.321012 2.190657 -2.673471
C 3.201095 0.881577 -3.303802
C 2.105652 0.014367 -3.003856
C -4.762879 -2.304848 2.665486
C -5.622872 -1.208100 3.074109
C -4.061255 -3.489723 0.610831
C -5.096639 -3.094413 1.550263
C -6.306127 1.513932 2.490625
C -6.028249 2.650339 1.622006
C -6.796347 -0.923256 2.347095
C -7.142598 0.462716 2.046881
C -6.594793 2.687808 0.324436
C -5.780038 3.133852 -0.796854
C -7.713122 0.500534 0.706125
C -7.434621 1.594342 -0.143970
C -6.102034 2.298888 -1.957295
C -5.073086 1.874659 -2.826103
C -7.135946 1.359934 -1.556299
C 5.109259 3.159700 -1.317298
C 4.705533 2.509415 -2.505382
C 2.398707 -1.388257 -2.698896
C -0.774093 -1.219366 -2.426435
C 4.546445 0.407800 -3.523344
C 4.812332 -0.955326 -3.342600
C 3.743266 -1.835468 -2.905005
C 5.492047 1.413234 -3.051877
C -2.072856 -1.780883 -2.573726
C -3.310410 -1.266631 -3.178645
C -3.636013 0.088346 -3.422634
C -5.033418 0.515446 -3.347330
C -2.486124 -2.845152 -1.670357
C -4.661084 -3.496253 -0.717243
C -3.879956 -3.108648 -1.827961
C 3.654195 -3.328322 -0.945708
C 4.340102 -3.545559 0.320085
C 4.369601 -2.835897 -2.045402
C 5.247621 -2.203145 2.681921
C 5.327967 -0.892950 3.325270
C 5.708505 -3.248229 0.466210
C 6.169796 -2.555585 1.667353
C 6.327848 0.021017 2.933408
C 5.992459 1.431085 2.767824
C -6.317173 -2.816474 0.806215
C -7.144357 -1.739819 1.189210
C -7.707564 -0.856663 0.169275
C -6.037079 -3.059922 -0.607458
C -4.403663 -2.174274 -2.805362
C -5.744078 -1.751856 -2.705026
C -6.576046 -2.199215 -1.592439
C -7.426220 -1.083221 -1.195885
C 5.789871 -2.554076 -1.919057
C 6.449269 -2.735591 -0.675281
C 6.064978 -1.368343 -2.716978
C -6.075091 -0.372135 -2.994909
C -7.123924 0.046702 -2.073863
C 6.979097 -0.397111 -2.250096
C 6.682184 1.022002 -2.411522
C 7.376708 -1.724463 -0.184765
C 7.632560 -0.571217 -0.955478
C 7.200440 -1.608695 1.263486
C 7.275289 -0.341897 1.884364
C 6.153587 2.893136 0.771599
C 6.748728 1.945042 1.634511
C 6.352788 2.779542 -0.673103
C 7.125966 1.720695 -1.207636
C 7.721444 0.736306 -0.313481
C 7.537781 0.850818 1.084333

Iso b13
E = -4569.022108
C -0.785388 -1.850743 2.961017
C -0.721079 -0.513035 2.450308
C -1.895338 -2.738079 2.633260
C -2.659594 -2.410607 1.434000
C -2.360214 -1.193655 0.718664
C -1.695069 -0.120919 1.470331
C 0.567134 0.251849 2.575472
C 0.618294 1.657991 2.756539
C -0.630827 2.397938 2.996569
C -1.868607 2.203719 2.265792
C -2.105359 1.229189 1.239288
C -2.360214 -1.193655 -0.718664
C -1.695069 -0.120919 -1.470331
C -2.105359 1.229189 -1.239288
C -2.724832 1.702363 0.000000
C 0.430092 -2.454100 3.405852

C 1.653237 -1.981974 2.784313
C 1.725128 -0.668958 2.289381
C -3.305390 -3.458756 0.719815
C -3.305390 -3.458756 -0.719815
C -2.659594 -2.410607 -1.434000
C -1.843314 -4.104828 3.053504
C -2.523432 -5.154024 2.314993
C -3.270539 -4.835377 1.170136
C 0.534649 -3.855194 3.647511
C -0.607826 -4.677792 3.563756
C -0.491261 -6.042563 3.082848
C -1.696278 -6.356714 2.328119
C 1.807080 -4.313631 3.107593
C 1.921587 -5.635753 2.631840
C 0.763645 -6.522657 2.632726
C 2.423528 -3.177303 2.425304
C 2.548974 -0.315746 1.168120
C 2.716272 1.014982 0.716619
C 2.730805 2.239752 1.453173
C 1.818173 2.543253 2.546415
C 2.548974 -0.315746 -1.168120
C 2.754166 -1.196206 -0.000000
C 2.716272 1.014982 -0.716619
C 3.080912 -3.428103 1.191031
C 3.032777 -2.565624 -0.000000
C -2.625335 3.439016 2.280832
C -3.395797 3.808046 1.163845
C -3.394859 2.950097 0.000000
C -0.634437 3.778235 3.424299
C -1.877073 4.426148 3.012607
C 1.775367 3.911037 2.955202
C 0.550826 4.536944 3.437315
C -3.235622 -5.699124 -0.000000
C -3.270539 -4.835377 -1.170136
C -1.628637 -7.176771 1.180010
C -2.420679 -6.854078 -0.000000
C -1.895338 -2.738079 -2.633260
C -0.785388 -1.850743 -2.961017
C -0.721079 -0.513035 -2.450308
C -2.523432 -5.154024 -2.314993
C -1.843314 -4.104828 -3.053504
C -1.868607 2.203719 -2.265792
C -2.625335 3.439016 -2.280832
C -3.395797 3.808046 -1.163845
C 0.567134 0.251849 -2.575472
C 0.618294 1.657991 -2.756539
C -0.630827 2.397938 -2.996569
C 3.369299 3.290798 0.731524
C 3.322618 4.653295 1.167224
C 2.541397 4.959187 2.295368
C 2.730805 2.239752 -1.453173
C 3.369299 3.290798 -0.731524
C 0.535039 5.931537 3.041310
C -0.670761 6.546856 2.613171
C -1.903700 5.778744 2.611460
C 1.771203 6.192842 2.312405
C -2.682856 6.165356 1.438150
C -3.427748 5.195842 0.730886
C -3.427748 5.195842 -0.730886
C 0.430092 -2.454100 -3.405852
C 1.653237 -1.981974 -2.784313
C 1.725128 -0.668958 -2.289381
C -0.607826 -4.677792 -3.563756
C 0.534649 -3.855194 -3.647511
C -0.634437 3.778235 -3.424299
C -1.877073 4.426148 -3.012607
C 1.818173 2.543253 -2.546415
C 1.775367 3.911037 -2.955202
C 0.550826 4.536944 -3.437315
C -1.903700 5.778744 -2.611460
C -2.682856 6.165356 -1.438150
C 2.686514 -5.894622 1.432136
C 3.230420 -4.795744 0.726350
C 3.080912 -3.428103 -1.191031
C 2.423528 -3.177303 -2.425304
C 3.230420 -4.795744 -0.726350
C 3.322618 4.653295 -1.167224
C 3.324521 5.522933 0.000000
C 2.541397 4.959187 -2.295368
C -1.628637 -7.176771 -1.180010
C -1.696278 -6.356714 -2.328119
C -0.491261 -6.042563 -3.082848
C 0.838283 -7.356006 1.439703
C -0.337172 -7.690512 0.729216
C -0.337172 -7.690512 -0.729216
C 2.043970 -6.978713 0.704200
C 2.043970 -6.978713 -0.704200
C 0.838283 -7.356006 -1.439703
C 2.686514 -5.894622 -1.432136
C 2.548315 6.695030 0.000000
C 1.770037 7.046258 1.182749
C 1.771203 6.192842 -2.312405
C 1.770037 7.046258 -1.182749
C 0.535039 5.931537 -3.041310
C 1.807080 -4.313631 -3.107593
C 1.921587 -5.635753 -2.631840
C 0.763645 -6.522657 -2.632726
C -0.670761 6.546856 -2.613171
C -1.917876 7.167865 0.706316
C -1.917876 7.167865 -0.706316
C -0.676120 7.408227 -1.439807
C -0.676120 7.408227 1.439807
C 0.520652 7.650274 -0.728709
C 0.520652 7.650274 0.728709

Iso b14
E = -4569.021580
C 0.194432 0.653236 1.931820
C -0.204838 1.812612 1.225747
C -0.881868 -0.232016 2.361967
C -2.048085 0.417707 2.886900
C -2.378999 1.750437 2.395494
C -1.581546 2.248315 1.264776
C -3.688078 2.233785 2.637579
C -4.328934 3.088782 1.662844
C -3.650756 3.382720 0.464741
C -2.289135 2.924359 0.200787
C 0.819553 2.467985 0.379648
C 0.943620 2.341045 -1.015194
C 0.058593 1.515075 -1.840520
C -1.341856 1.721177 -1.951815
C -2.194837 2.672956 -1.252533
C -0.989855 -1.565939 1.877011
C -0.021400 -2.083135 0.921440
C 1.362181 -2.087039 1.303956
C 1.946114 -1.170500 2.317721
C 1.548790 0.250861 2.323148
C -3.084196 -0.406768 3.397292
C -4.417072 0.088640 3.626796
C -4.714093 1.414991 3.260789
C -2.249708 -2.253759 1.971714
C -3.182085 -1.777622 2.932827
C -0.579577 -2.487809 -0.358165
C -1.970634 -2.841131 -0.439406
C -2.728989 -3.011476 0.821381
C -4.103530 -3.371990 0.801614
C -5.034387 -2.939018 1.834253
C -4.578655 -2.134447 2.890739
C 0.249170 -2.035341 -1.527739
C 1.616623 -2.307821 -1.699338
C 2.391360 -3.128597 -0.796083
C 2.283383 -2.977057 0.641749
C -2.765247 -2.733611 -1.645228
C -4.176726 -3.079440 -1.638829
C -4.840252 -3.423858 -0.448733
C -0.271252 -0.859116 -2.206050
C -1.617109 -0.645421 -2.597055
C -2.604359 -1.681605 -2.651217
C 0.627139 0.263821 -2.299559
C -2.054724 0.728867 -2.769892
C -3.514118 2.932598 -1.809519
C -4.067072 2.156474 -2.855074
C -3.327578 0.992551 -3.289090
C -3.893965 -1.396752 -3.227305
C -4.255056 -0.076199 -3.576877
C 2.405995 -1.438702 -2.591683
C 1.998627 -0.032784 -2.671840
C -4.405140 3.414899 -0.772808
C -5.784627 3.123392 -0.796188
C -6.335035 2.287264 -1.858839
C -5.476974 1.809632 -2.870786
C -5.754788 2.786582 1.657568
C -6.470894 2.785301 0.444041
C -5.990556 1.732791 2.642317
C -6.913628 0.698478 2.364238
C -7.633460 0.687844 1.097470
C -7.423512 1.718936 0.156520
C -5.360280 -0.981039 3.326082
C -6.586696 -0.685443 2.706069
C -6.322952 -2.664216 1.214713
C -7.081752 -1.547128 1.635120
C -4.880249 -2.271717 -2.628158
C -6.204736 -2.972979 -0.205518
C -6.867020 -2.165728 -1.156626
C -6.190463 -1.813728 -2.398376
C -5.594116 0.416692 -3.322332
C -6.555517 -0.437646 -2.741521
C -7.332495 1.405730 -1.267828
C -7.441389 0.063102 -1.701906
C -7.740621 -0.699883 0.648792
C -7.638403 -1.006401 -0.724058
C 3.773066 -3.454332 -1.086070
C 4.432609 -2.859334 -2.176041
C 3.729693 -1.808590 -2.912634
C 3.569133 -3.290767 1.221244
C 4.503797 -3.623075 0.164511
C 3.206794 -1.518832 2.874461
C 4.013295 -2.615107 2.366773
C 2.053481 3.016613 0.902375
C 2.750849 2.550186 2.042313
C 2.525967 1.235229 2.682415
C 2.254886 2.836269 -1.409338
C 2.855027 3.460187 -0.233333
C 2.999030 0.947408 -2.971433
C 3.116221 2.307615 -2.390048
C 7.094228 1.828515 -1.260323
C 7.749679 0.815614 -0.434238
C 6.553012 1.161010 -2.438102
C 6.909507 -0.254510 -2.359968
C 7.647813 -0.467369 -1.119370
C 7.634464 0.876586 0.970597
C 7.406727 -0.346766 1.733634
C 7.327446 -1.595041 1.072049
C 7.454238 -1.658475 -0.383613
C 6.008425 -1.234075 -2.815963
C 5.833816 -2.484906 -2.082383
C 6.550072 -2.689806 -0.876853
C 6.374233 2.881618 -0.661923
C 6.260653 2.950218 0.797207
C 6.867781 1.954245 1.590729
C 5.290541 1.542551 -2.936479
C 4.331138 0.528515 -3.338689
C 4.698869 -0.830446 -3.314204
C 5.101159 3.311976 -1.229528
C 4.543377 2.628546 -2.335414
C 4.918580 3.425543 1.113475
C 4.230971 3.684975 -0.138270
C 4.182629 2.842886 2.167923
C 6.134463 1.383905 2.713469
C 4.812064 1.803497 2.963202
C 6.474793 -0.033278 2.808444
C 5.484988 -0.969257 3.173168
C 4.117604 -0.526629 3.397106
C 3.785805 0.828168 3.266413
C 6.330819 -2.575922 1.475617

C 5.417641 -2.268432 2.510148
C 5.858911 -3.261705 0.272975

Iso b15
E = -4569.016078
C 2.459251 3.068734 2.261971
C 0.568441 0.420122 -2.242256
C 2.426228 1.030172 -0.709227
C 2.924868 2.310510 -1.166089
C 2.459251 3.068734 -2.261971
C 1.156539 2.834501 -2.922881
C 0.225217 1.777293 -2.640198
C 2.426228 1.030172 0.709227
C 2.924868 2.310510 1.166089
C 3.436918 3.014748 0.000000
C 0.568441 0.420122 2.242256
C 0.225217 1.777293 2.640198
C 1.156539 2.834501 2.922881
C 2.708477 4.507320 2.278964
C 1.620228 5.158126 2.987139
C 0.680470 4.123566 3.376447
C -1.193109 2.152449 2.541809
C -1.628622 3.421894 2.964543
C -0.695446 4.417915 3.422978
C -1.187826 5.725978 3.027779
C -0.278557 6.715387 2.601787
C 1.155174 6.430748 2.595219
C 1.740750 7.087950 1.433294
C 2.794645 6.465118 0.733153
C 3.300047 5.172989 1.176974
C 3.636127 4.402609 0.000000
C -1.998366 1.399590 1.567633
C -1.710172 0.008914 1.471723
C -1.710172 0.008914 -1.471723
C -1.998366 1.399590 -1.567633
C -2.889734 2.161390 -0.734367
C -2.889734 2.161390 0.734367
C -2.724377 4.101301 2.295790
C -3.290376 3.490191 1.159227
C -2.450800 5.525323 2.312221
C -3.593273 4.307475 0.000000
C -3.330312 5.694648 0.000000
C -2.747407 6.320596 1.180478
C -0.584661 7.529503 1.433542
C -1.796395 7.328398 0.729973
C 0.659716 7.748711 0.705724
C -1.796395 7.328398 -0.729973
C -0.584661 7.529503 -1.433542
C 0.659716 7.748711 -0.705724
C 2.794645 6.465118 -0.733153
C 1.740750 7.087950 -1.433294
C 3.300047 5.172989 -1.176974
C 2.708477 4.507320 -2.278964
C 1.155174 6.430748 -2.595219
C 1.620228 5.158126 -2.987139
C 0.680470 4.123566 -3.376447
C -0.695446 4.417915 -3.422978
C -1.187826 5.725978 -3.027779
C -0.278557 6.715387 -2.601787
C -1.628622 3.421894 -2.964543
C -2.724377 4.101301 -2.295790
C -2.450800 5.525323 -2.312221
C -2.747407 6.320596 -1.180478
C -3.290376 3.490191 -1.159227
C -1.193109 2.152449 -2.541809
C -2.694733 -2.256797 1.411886
C -2.608173 -5.018830 -2.293268
C -3.368380 -4.669750 -1.167059
C -3.385142 -5.537951 0.000000
C -2.649114 -6.742759 0.000000
C -1.877856 -7.117529 -1.179618
C -1.863341 -6.273893 -2.312906
C -3.384405 -3.289042 -0.722284
C -3.384405 -3.289042 0.722284
C -3.368380 -4.669750 1.167059
C -2.694733 -2.256797 -1.411886
C -2.457590 -1.014170 -0.712778
C -2.457590 -1.014170 0.712778
C -1.794102 -2.650887 2.500576
C -0.569158 -1.865379 2.635495
C -0.577903 -0.511248 2.166424
C -0.577903 -0.511248 -2.166424
C 1.724369 0.021939 -1.496153
C 1.724369 0.021939 1.496153
C 2.141581 -1.353359 1.271340
C 1.899887 -2.332683 2.282239
C 0.638818 -2.533877 3.004755
C 0.606536 -3.916815 3.429910
C -0.601477 -4.638826 3.431506
C -1.812345 -3.998330 2.953586
C -2.608173 -5.018830 2.293268
C -0.569158 -1.865379 -2.635495
C -1.794102 -2.650887 -2.500576
C -1.812345 -3.998330 -2.953586
C -0.601477 -4.638826 -3.431506
C 0.606536 -3.916815 -3.429910
C 0.638818 -2.533877 -3.004755
C 2.141581 -1.353359 -1.271340
C 1.899887 -2.332683 -2.282239
C 2.734161 -1.835006 0.000000
C 2.599192 -3.599385 -2.279791
C 3.357026 -3.981354 -1.163866
C 3.354010 -3.113470 0.000000
C 2.599192 -3.599385 2.279791
C 3.357026 -3.981354 1.163866
C 1.834241 -4.580306 3.018827
C 3.351822 -5.369518 0.729981
C 2.593006 -6.330774 1.435991
C 1.824396 -5.929886 2.611116
C -0.618873 -6.041307 3.034031
C 0.574337 -6.675496 2.616620
C -1.863341 -6.273893 2.312906
C -1.877856 -7.117529 1.179618
C 0.563849 -7.534617 1.436832
C -0.638396 -7.746172 0.729580
C -0.638396 -7.746172 -0.729580
C 0.563849 -7.534617 -1.436832
C 1.811778 -7.321543 -0.706822
C 1.811778 -7.321543 0.706822
C 0.574337 -6.675496 -2.616620
C 1.824396 -5.929886 -2.611116
C 2.593006 -6.330774 -1.435991
C 3.351822 -5.369518 -0.729981
C 1.834241 -4.580306 -3.018827
C -0.618873 -6.041307 -3.034031

Iso b16
E = -4569.014005
C -1.584469 2.552749 0.971265
C -0.194539 2.117317 1.042185
C -2.436221 2.182469 2.118714
C -2.120836 0.927479 2.794124
C -0.939502 0.212551 2.391092
C 0.168249 1.034644 1.887903
C 0.882511 2.651577 0.156667
C 2.152526 3.198502 0.607281
C 2.848756 2.840860 1.787840
C 2.562269 1.624305 2.577405
C 1.524062 0.657066 2.340971
C -1.060310 -1.184512 2.114949
C -0.094648 -1.825827 1.236958
C 1.277148 -1.754164 1.579281
C 1.887145 -0.763856 2.485126
C -2.276576 3.079061 -0.191342
C -2.124898 2.640348 -1.592720
C -1.223836 1.693003 -2.151809
C 0.169917 1.502834 -1.985016
C 1.033405 2.342626 -1.206551
C -3.170244 0.181770 3.401207
C -3.255745 -1.247900 3.185207
C -2.301388 -1.878124 2.347109
C -3.765726 2.670309 2.239353
C -4.807666 1.933883 2.938845
C -4.511888 0.690755 3.519143
C -3.667921 3.506926 -0.059181
C -4.390257 3.366358 1.136310
C -5.810750 3.020716 1.117637
C -6.069882 2.131933 2.241391
C -4.372178 3.304944 -1.317645
C -5.733193 2.949776 -1.354461
C -6.473683 2.803717 -0.109235
C -3.424445 2.695849 -2.218814
C 2.351852 2.726245 -1.661034
C 2.973935 3.464418 -0.575475
C 0.547509 0.227594 -2.509577
C 1.888622 -0.272896 -2.736036
C 2.955091 0.634181 -3.054665
C 3.148554 2.042620 -2.597442
C -1.732025 0.540612 -2.879814
C -0.684935 -0.489426 -2.788802
C -3.871114 1.670201 -3.090503
C -3.057865 0.489640 -3.293753
C -0.634149 -2.527588 0.089702
C -1.973715 -2.994611 0.156179
C -2.749689 -2.885734 1.390690
C 2.138446 -2.741565 0.989645
C 2.191010 -3.056976 -0.411682
C 1.383653 -2.388393 -1.435922
C -0.011462 -2.297234 -1.241994
C 3.134226 -1.119339 3.065850
C 3.879534 -2.302285 2.663975
C 3.399588 -3.081539 1.603978
C 3.818184 1.218673 3.185125
C 4.097229 -0.126613 3.464867
C -5.443988 -0.425575 3.380874
C -4.650616 -1.628497 3.181324
C -6.974325 1.051051 2.101935
C -6.652936 -0.250981 2.681791
C -5.081342 -2.621467 2.280821
C -4.124214 -3.251730 1.388596
C -2.686736 -3.156523 -1.074837
C -2.317300 -2.341268 -2.216827
C -0.995290 -1.773710 -2.252032
C -4.811895 -3.520184 0.131811
C -4.098384 -3.400831 -1.077558
C -6.342523 -2.458785 1.571573
C -6.166199 -3.010817 0.230897
C -7.107097 -1.286202 1.756854
C 2.205736 -1.679711 -2.465154
C 3.544084 -3.509355 -0.668988
C 4.213924 -3.098325 -1.828431
C 3.538189 -2.119500 -2.688722
C 4.288898 -3.586276 0.586849
C -4.672104 -2.693396 -2.203728
C -3.589979 -1.941145 -2.851108
C -6.753137 -2.365483 -0.880267
C -5.993202 -2.208139 -2.116196
C -3.932572 -0.680239 -3.403237
C -5.255342 1.270695 -3.111280
C -6.193914 1.915421 -2.277811
C -5.300667 -0.178576 -3.314742
C -6.325012 -0.918840 -2.689580
C 4.266628 0.123538 -3.356995
C 4.560145 -1.241685 -3.193571
C -7.218727 1.133444 -1.603387
C -7.394669 1.683277 -0.261258
C -7.286571 -0.261762 -1.813806
C -7.640990 0.819336 0.828548
C -7.720364 -0.624548 0.609787
C -7.544900 -1.155347 -0.686451
C 5.633707 -2.787678 -1.782792
C 6.361421 -2.889706 -0.566724
C 5.661976 -3.288971 0.645406
C 5.855895 -1.641366 -2.653525
C 6.179690 -2.493508 1.760848

C 5.301925 -2.021897 2.764301
C 5.439585 -0.662397 3.278120
C 4.298873 3.070796 1.859508
C 5.051537 3.488383 0.739034
C 4.359371 3.635061 -0.526047
C 4.888485 2.095935 2.758748
C 5.188046 3.089953 -1.576851
C 4.586469 2.303663 -2.589906
C 5.275716 1.129665 -3.076542
C 7.558680 -0.766483 -1.067782
C 7.732015 0.579574 -0.532430
C 6.813217 -0.662002 -2.318539
C 6.526588 0.749929 -2.551078
C 7.113029 1.521893 -1.462177
C 6.448985 2.667296 -0.977986
C 7.640863 0.807877 0.857338
C 6.933147 1.984132 1.359014
C 6.363852 2.913035 0.463457
C 7.320522 -1.854781 -0.200169
C 7.218838 -1.617529 1.239817
C 7.364216 -0.307846 1.754920
C 6.464976 0.174156 2.796876
C 6.188099 1.585019 2.544972

Iso p1
E = -4568.994936
C 7.432201 1.181628 1.436030
C 7.007559 2.327498 0.731600
C 7.007559 2.327498 -0.731600
C 7.432201 1.181628 -1.436030
C 7.880904 -0.000000 -0.704964
C 7.880904 -0.000000 0.704964
C 5.836846 3.073205 -1.180944
C 5.112060 2.625954 -2.311072
C 5.541997 1.433827 -3.024069
C 6.694254 0.728621 -2.610374
C 6.694254 -0.728621 -2.610374
C 5.541997 -1.433827 -3.024069
C 4.340025 -0.698435 -3.405197
C 4.340025 0.698435 -3.405197
C 3.169887 1.420711 -2.921946
C 2.006081 0.742682 -2.435525
C 2.006081 -0.742682 -2.435525
C 3.169887 -1.420711 -2.921946
C 5.112060 -2.625954 -2.311072
C 3.652176 -2.632530 -2.289705
C 3.652176 2.632530 -2.289705
C 2.971289 3.146358 -1.168140
C 1.743332 2.525198 -0.746804
C 1.216033 1.418604 -1.428985
C 0.000000 0.773120 -0.812931
C 1.216033 -1.418604 -1.428985
C 1.743332 -2.525198 -0.746804
C 2.971289 -3.146358 -1.168140
C 7.432201 -1.181628 -1.436030
C 7.007559 -2.327498 -0.731600
C 5.836846 -3.073205 -1.180944
C 5.127101 -3.550504 0.000000
C 3.716968 -3.587750 0.000000
C -0.000000 0.773120 0.812931
C 1.216033 1.418604 1.428985
C 1.743332 2.525198 0.746804
C 2.971289 3.146358 1.168140
C 3.716968 3.587750 0.000000
C 5.127101 3.550504 0.000000
C 5.836846 3.073205 1.180944
C 5.112060 2.625954 2.311072
C 3.652176 2.632530 2.289705
C 2.006081 0.742682 2.435525
C 3.169887 1.420711 2.921946
C 6.694254 0.728621 2.610374
C 5.541997 1.433827 3.024069
C 4.340025 0.698435 3.405197
C 1.216033 -1.418604 1.428985
C 1.743332 -2.525198 0.746804
C 2.971289 -3.146358 1.168140
C 7.432201 -1.181628 1.436030
C 7.007559 -2.327498 0.731600
C 5.836846 -3.073205 1.180944
C 5.112060 -2.625954 2.311072
C 3.652176 -2.632530 2.289705
C 2.006081 -0.742682 2.435525
C 3.169887 -1.420711 2.921946
C 4.340025 -0.698435 3.405197
C 5.541997 -1.433827 3.024069
C 6.694254 -0.728621 2.610374
C -1.216033 -1.418604 -1.428985
C -1.743332 -2.525198 -0.746804
C -1.743332 -2.525198 0.746804
C -1.216033 -1.418604 1.428985
C -0.000000 -0.773120 0.812931
C -0.000000 -0.773120 -0.812931
C -2.006081 -0.742682 2.435525
C -3.169887 -1.420711 2.921946
C -3.652176 -2.632530 2.289705
C -2.971289 -3.146358 1.168140
C -4.340025 -0.698435 3.405197
C -4.340025 0.698435 3.405197
C -3.169887 1.420711 2.921946
C -2.006081 0.742682 2.435525
C -1.216033 1.418604 1.428985
C -1.743332 2.525198 0.746804
C -2.971289 3.146358 1.168140
C -3.652176 2.632530 2.289705
C -1.743332 2.525198 -0.746804
C -2.971289 3.146358 -1.168140
C -3.716968 3.587750 0.000000
C -5.127101 3.550504 0.000000
C -5.836846 3.073205 -1.180944
C -5.112060 2.625954 -2.311072
C -3.652176 2.632530 -2.289705
C -3.169887 1.420711 -2.921946
C -2.006081 0.742682 -2.435525
C -1.216033 1.418604 -1.428985
C -5.836846 3.073205 1.180944
C -5.112060 2.625954 2.311072
C -5.541997 1.433827 3.024069
C -5.112060 -2.625954 2.311072
C -5.541997 -1.433827 3.024069
C -6.694254 -0.728621 2.610374
C -6.694254 0.728621 2.610374
C -5.836846 -3.073205 1.180944
C -5.127101 -3.550504 -0.000000
C -3.716968 -3.587750 -0.000000
C -2.971289 -3.146358 -1.168140
C -7.007559 -2.327498 0.731600
C -7.432201 -1.181628 1.436030
C -7.007559 2.327498 0.731600
C -7.432201 1.181628 1.436030
C -7.880904 -0.000000 0.704964
C -7.880904 0.000000 -0.704964
C -7.432201 -1.181628 -1.436030
C -7.007559 -2.327498 -0.731600
C -5.836846 -3.073205 -1.180944
C -5.112060 -2.625954 -2.311072
C -3.652176 -2.632530 -2.289705
C -7.432201 1.181628 -1.436030
C -7.007559 2.327498 -0.731600
C -6.694254 0.728621 -2.610374
C -6.694254 -0.728621 -2.610374
C -5.541997 -1.433827 -3.024069
C -4.340025 -0.698435 -3.405197
C -3.169887 -1.420711 -2.921946
C -2.006081 -0.742682 -2.435525
C -4.340025 0.698435 -3.405197
C -5.541997 1.433827 -3.024069

Iso p2
E = -4568.993900
C -3.029785 -2.804494 -1.834745
C -2.744848 -1.592613 2.611056
C -2.911842 -2.680783 1.732473
C -4.270931 -3.182884 1.497590
C -5.395753 -2.629565 2.142725
C -5.212541 -1.528484 3.066871
C -3.916061 -1.023248 3.274993
C -2.160779 -2.837745 0.463723
C -3.092373 -3.312717 -0.518962
C -4.380588 -3.562266 0.097457
C -1.004948 -2.132277 0.044203
C -0.928238 -1.640868 -1.298104
C -2.026824 -1.835796 -2.162956
C -4.248304 -2.511435 -2.563043
C -4.020813 -1.278316 -3.308911
C -2.660160 -0.817373 -3.033506
C -0.168167 0.699424 -1.693568
C -1.459887 1.216039 -1.966278
C -2.450147 0.567869 -2.833546
C -3.572929 1.485115 -3.003470
C -4.862713 1.039331 -3.341773
C -5.102736 -0.384646 -3.477368
C -6.405858 -0.674118 -2.896446
C -6.607527 -1.863328 -2.156787
C -5.501220 -2.789705 -1.985625
C -5.567866 -3.331586 -0.622998
C 0.003686 1.677958 1.054457
C -0.454373 -0.602509 1.964296
C -1.715809 -0.532734 2.531049
C -2.393100 0.727543 2.908193
C -2.240710 1.915330 2.169254
C -1.298882 2.131150 1.053334
C -2.120163 2.395825 -1.377213
C -2.063640 2.816291 -0.012981
C -3.385393 2.592791 -2.091003
C -3.281116 3.324134 0.606035
C -4.468629 3.574945 -0.109897
C -4.514132 3.204896 -1.511537
C -6.028446 1.648091 -2.712136
C -5.852859 2.710810 -1.806388
C -6.980382 0.582524 -2.420854
C -6.628660 2.752344 -0.567857
C -7.542884 1.715963 -0.278449
C -7.719742 0.614637 -1.218065
C -7.356805 -1.824392 -0.904044
C -7.906535 -0.610035 -0.443354
C -6.717688 -2.738204 0.039576
C -6.635942 -2.398233 1.408667
C -7.840159 -0.267296 0.972220
C -7.218566 -1.150072 1.887808
C -6.343525 -0.614296 2.923555
C -6.136159 0.774478 3.031019
C -6.787804 1.688564 2.089783
C -7.621365 1.170956 1.075824
C -4.801973 1.289155 3.301049
C -4.632197 2.514329 2.539802
C -5.857043 2.768553 1.794478
C -5.768823 3.285260 0.479900
C -3.377883 2.783586 1.959177
C -3.707476 0.405826 3.431300
C 6.628660 -2.752332 -0.567913
C 7.356805 1.824411 -0.904007
C 7.906534 0.610043 -0.443342
C 7.840159 0.267276 0.972225
C 7.218566 1.150034 1.887831
C 6.635942 2.398204 1.408716
C 6.717687 2.738202 0.039632
C 7.719742 -0.614612 -1.218078
C 7.542884 -1.715957 -0.278484
C 7.621366 -1.170978 1.075801
C 6.980382 -0.582474 -2.420866
C 6.028446 -1.648035 -2.712170
C 5.852859 -2.710772 -1.806443
C 5.768823 -3.285270 0.479833
C 4.468629 -3.574945 -0.109970
C 4.514132 -3.204864 -1.511602

C 4.862713 -1.039263 -3.341795
C 3.572929 -1.485052 -3.003499
C 3.385393 -2.592747 -2.091055
C 2.120163 -2.395796 -1.377261
C 2.063641 -2.816293 -0.013037
C 3.281117 -3.324147 0.605968
C 3.377884 -2.783627 1.959120
C 4.632198 -2.514381 2.539751
C 5.857043 -2.768590 1.794421
C 6.787805 -1.688608 2.089749
C 5.102735 0.384717 -3.477361
C 6.405858 0.674178 -2.896433
C 6.607527 1.863372 -2.156749
C 5.501220 2.789746 -1.985569
C 4.248303 2.511488 -2.562991
C 4.020813 1.278385 -3.308884
C 2.450148 -0.567810 -2.833556
C 2.660160 0.817435 -3.033488
C 1.459887 -1.215999 -1.966300
C 2.026824 1.835842 -2.162919
C 0.928237 1.640897 -1.298071
C 0.168167 -0.699387 -1.693581
C 1.298883 -2.131173 1.053292
C -0.003685 -1.677982 1.054424
C 2.240710 -1.915375 2.169216
C 0.454373 0.602468 1.964308
C 1.715809 0.532681 2.531059
C 2.393100 -0.727603 2.908178
C 4.801973 -1.289223 3.301023
C 3.707476 -0.405897 3.431291
C 6.136159 -0.774540 3.031003
C 6.343525 0.614236 2.923568
C 3.916060 1.023180 3.275014
C 5.212541 1.528421 3.066902
C 5.395753 2.629521 2.142778
C 4.270930 3.182852 1.497655
C 2.911842 2.680746 1.732527
C 2.744848 1.592559 2.611088
C 4.380588 3.562265 0.097529
C 3.092373 3.312729 -0.518895
C 2.160779 2.837736 0.463781
C 1.004948 2.132277 0.044247
C 3.029785 2.804532 -1.834688
C 5.567866 3.331598 -0.622929

Iso c1
E = -4568.973711
C -7.595209 1.437433 1.183285
C -7.156680 0.730052 2.326417
C -7.156680 -0.730052 2.326417
C -7.595209 -1.437433 1.183285
C -8.037937 -0.704479 0.000000
C -8.037937 0.704479 0.000000
C -5.976977 -1.182236 3.050083
C -5.273811 -2.326183 2.618034
C -5.728973 -3.058989 1.435240
C -6.869200 -2.616157 0.732199
C -6.869200 -2.616157 -0.732199
C -5.728973 -3.058989 -1.435240
C -4.553127 -3.517345 -0.707470
C -4.553127 -3.517345 0.707470
C -3.376522 -3.059509 1.436640
C -2.241280 -2.599566 0.731101
C -2.241280 -2.599566 -0.731101
C -3.376522 -3.059509 -1.436640
C -5.273811 -2.326183 -2.618034
C -3.817383 -2.330255 -2.614113
C -3.817383 -2.330255 2.614113
C -3.103368 -1.182184 3.028072
C -1.922687 -0.740709 2.303315
C -1.489079 -1.457357 1.185049
C -0.794726 -0.798939 0.000000
C -1.489079 -1.457357 -1.185049
C -1.922687 -0.740709 -2.303315
C -3.103368 -1.182184 -3.028072
C -7.595209 -1.437433 -1.183285
C -7.156680 -0.730052 -2.326417
C -5.976977 -1.182236 -3.050083
C -5.241359 0.000000 -3.493901
C -3.833764 0.000000 -3.474978
C -0.794726 0.798939 0.000000
C -1.489079 1.457357 1.185049
C -1.922687 0.740709 2.303315
C -3.103368 1.182184 3.028072
C -3.833764 -0.000000 3.474978
C -5.241359 -0.000000 3.493901
C -5.976977 1.182236 3.050083
C -5.273811 2.326183 2.618034
C -3.817383 2.330255 2.614113
C -2.241280 2.599566 0.731101
C -3.376522 3.059509 1.436640
C -6.869200 2.616157 0.732199
C -5.728973 3.058989 1.435240
C -4.553127 3.517345 0.707470
C -1.489079 1.457357 -1.185049
C -1.922687 0.740709 -2.303315
C -3.103368 1.182184 -3.028072
C -7.595209 1.437433 -1.183285
C -7.156680 0.730052 -2.326417
C -5.976977 1.182236 -3.050083
C -5.273811 2.326183 -2.618034
C -3.817383 2.330255 -2.614113
C -2.241280 2.599566 -0.731101
C -3.376522 3.059509 -1.436640
C -4.553127 3.517345 -0.707470
C -5.728973 3.058989 -1.435240
C -6.869200 2.616157 -0.732199
C 1.489079 -1.457357 -1.185049
C 1.922687 -0.740709 -2.303315
C 1.922687 0.740709 -2.303315
C 1.489079 1.457357 -1.185049
C 0.794726 0.798939 0.000000
C 0.794726 -0.798939 0.000000
C 2.241280 2.599566 -0.731101
C 3.376522 3.059509 -1.436640
C 3.817383 2.330255 -2.614113
C 3.103368 1.182184 -3.028072
C 4.553127 3.517345 -0.707470
C 4.553127 3.517345 0.707470
C 3.376522 3.059509 1.436640
C 2.241280 2.599566 0.731101
C 1.489079 1.457357 1.185049
C 1.922687 0.740709 2.303315
C 3.103368 1.182184 3.028072
C 3.817383 2.330255 2.614113
C 1.922687 -0.740709 2.303315
C 3.103368 -1.182184 3.028072
C 3.833764 0.000000 3.474978
C 5.241359 0.000000 3.493901
C 5.976977 -1.182236 3.050083
C 5.273811 -2.326183 2.618034
C 3.817383 -2.330255 2.614113
C 3.376522 -3.059509 1.436640
C 2.241280 -2.599566 0.731101
C 1.489079 -1.457357 1.185049
C 5.976977 1.182236 3.050083
C 5.273811 2.326183 2.618034
C 5.728973 3.058989 1.435240
C 5.273811 2.326183 -2.618034
C 5.728973 3.058989 -1.435240
C 6.869200 2.616157 -0.732199
C 6.869200 2.616157 0.732199
C 5.976977 1.182236 -3.050083
C 5.241359 -0.000000 -3.493901
C 3.833764 -0.000000 -3.474978
C 3.103368 -1.182184 -3.028072
C 7.156680 0.730052 -2.326417
C 7.595209 1.437433 -1.183285
C 7.156680 0.730052 2.326417
C 7.595209 1.437433 1.183285
C 8.037937 0.704479 0.000000
C 8.037937 -0.704479 0.000000
C 7.595209 -1.437433 -1.183285
C 7.156680 -0.730052 -2.326417
C 5.976977 -1.182236 -3.050083
C 5.273811 -2.326183 -2.618034
C 3.817383 -2.330255 -2.614113
C 7.595209 -1.437433 1.183285
C 7.156680 -0.730052 2.326417
C 6.869200 -2.616157 0.732199
C 6.869200 -2.616157 -0.732199
C 5.728973 -3.058989 -1.435240
C 4.553127 -3.517345 -0.707470
C 3.376522 -3.059509 -1.436640
C 2.241280 -2.599566 -0.731101
C 4.553127 -3.517345 0.707470
C 5.728973 -3.058989 1.435240

Iso p3
E = -4568.973062
C 0.556371 -0.179226 -2.188037
C 0.134420 -1.486776 -1.738113
C -0.438432 0.859419 -2.007002
C -1.748350 0.546710 -2.492682
C -2.065944 -0.867398 -2.609112
C -1.272932 -1.781052 -1.756090
C -3.314621 -1.250547 -3.117119
C -3.990188 -2.420176 -2.601553
C -3.398337 -3.072508 -1.497168
C -2.082591 -2.712395 -0.985240
C 1.190956 -2.294417 -1.112718
C 1.351569 -2.496962 0.304832
C 0.918517 -1.545047 1.297523
C -0.403940 -1.116272 1.476737
C -1.572629 -2.004089 1.463686
C -2.169829 -2.851447 0.493446
C -0.030121 1.968212 -1.189971
C 1.335981 2.300339 -1.183726
C 2.162928 1.686582 -2.227480
C 1.872503 0.291314 -2.550910
C -2.783375 1.510260 -2.704549
C -4.041997 1.088485 -3.275271
C -4.307114 -0.274963 -3.500485
C -1.045298 2.505750 -0.265915
C -2.323673 3.032614 -0.701378
C -3.040051 2.703215 -1.878960
C -1.082960 2.283165 1.133864
C -2.411912 2.693583 1.617923
C -3.087811 3.347906 0.497292
C -4.472924 3.550746 0.453247
C -5.194565 3.381306 -0.793520
C -4.485275 2.946214 -1.931422
C -0.125015 1.356283 1.802046
C 1.271409 1.471999 1.855123
C 2.048315 2.571912 1.263308
C 2.069355 2.962667 -0.125923
C -3.251674 2.120197 2.613758
C -4.701030 2.365300 2.585053
C -5.309118 3.080519 1.534997
C -0.787685 0.112563 2.157010
C -2.047423 -0.110213 2.797261
C -3.073602 0.782125 3.191442
C -2.401769 -1.503141 2.548772
C -3.521239 -3.360191 0.808145
C -4.233694 -2.994790 1.973842
C -3.650182 -2.021261 2.865451
C -4.390060 0.237826 3.513732
C -4.679153 -1.139855 3.374506
C 2.102457 0.408140 2.528536
C 1.949281 -1.018674 2.232487
C -4.266186 -3.518009 -0.420257
C -5.662492 -3.301117 -0.465446
C -6.376507 -2.888720 0.736189
C -5.667156 -2.736107 1.941740
C -5.421913 -2.162282 -2.649636

C -6.254523 -2.595901 -1.598279
C -5.620726 -0.828175 -3.221662
C -6.643150 0.013940 -2.736052
C -7.504683 -0.440980 -1.654483
C -7.310052 -1.726797 -1.092545
C -5.099730 1.963706 -2.808489
C -6.379282 1.440016 -2.536180
C -6.506933 2.823793 -0.482178
C -7.092645 1.870228 -1.340861
C -5.395528 1.212616 3.142102
C -6.579402 2.639730 0.967397
C -7.232384 1.509962 1.498403
C -6.638589 0.791192 2.619386
C -5.942207 -1.585283 2.811325
C -6.912670 -0.633134 2.437426
C -7.386647 -1.906099 0.351784
C -7.647670 -0.797302 1.188876
C -7.776427 0.701279 -0.788157
C -7.846195 0.526552 0.608555
C 3.350135 2.905777 1.817623
C 3.958502 2.089360 2.783168
C 3.328377 0.811878 3.125675
C 3.396003 3.499225 -0.420420
C 4.181367 3.526255 0.796429
C 3.413700 2.245665 -2.534657
C 4.040817 3.205872 -1.635925
C 2.461134 -2.670242 -1.728043
C 3.211220 -1.927807 -2.683504
C 2.943554 -0.523370 -3.037756
C 2.576161 -3.232339 0.525402
C 3.237798 -3.434123 -0.737442
C 3.002700 -1.915578 2.562970
C 3.326108 -3.045096 1.689661
C 7.235522 -1.885392 1.060138
C 7.829737 -0.632692 0.601312
C 6.558042 -1.614110 2.325324
C 6.734835 -0.196875 2.642012
C 7.537510 0.405233 1.585261
C 7.829729 -0.318639 -0.773537
C 7.551911 1.047481 -1.207574
C 7.288821 2.058565 -0.256893
C 7.272750 1.729292 1.167585
C 5.673472 0.535280 3.214211
C 5.399977 1.905641 2.793442
C 6.205275 2.499317 1.792001
C 6.669525 -2.789074 0.132887
C 6.648504 -2.452293 -1.290775
C 7.215604 -1.236505 -1.728075
C 5.336989 -2.250946 2.611201
C 4.233497 -1.474569 3.163262
C 4.393577 -0.112941 3.448095
C 5.420882 -3.472497 0.447948
C 4.766969 -3.211903 1.673506
C 5.376668 -2.907695 -1.839656
C 4.635817 -3.559244 -0.777823
C 4.665876 -2.111784 -2.766290
C 6.527273 -0.441376 -2.734463
C 5.271498 -0.866955 -3.216960
C 6.752657 0.971350 -2.425606
C 5.732507 1.911964 -2.651569
C 4.442024 1.457921 -3.150352
C 4.203698 0.092018 -3.399444
C 6.243901 3.047094 -0.501881
C 5.477762 2.982166 -1.685794
C 5.575770 3.319631 0.766693

Iso c2
E = -4568.969095
C 0.000000 0.702404 1.414998
C -0.000000 -0.702404 1.414998
C 1.272247 1.397870 1.563539
C 2.161944 0.734819 2.517879
C 2.161944 -0.734819 2.517879
C 1.272247 -1.397870 1.563539
C 3.052089 -3.055758 1.165666
C 1.807832 -2.438671 0.754338
C 3.800525 -3.491197 0.000000
C 3.052089 -3.055758 -1.165666
C 1.807832 -2.438671 -0.754338
C 2.161944 -0.734819 -2.517879
C 1.272247 -1.397870 -1.563539
C 2.161944 0.734819 -2.517879
C 1.272247 1.397870 -1.563539
C 0.000000 0.702404 -1.414998
C -0.000000 -0.702404 -1.414998
C 3.052089 3.055758 -1.165666
C 1.807832 2.438671 -0.754338
C 3.800525 3.491197 0.000000
C 3.052089 3.055758 1.165666
C 1.807832 2.438671 0.754338
C 5.222522 2.623214 2.317137
C 3.765340 2.612762 2.303948
C 5.675663 1.436788 3.039708
C 4.491092 0.700150 3.455665
C 3.320031 1.411455 2.975908
C 5.675663 -1.436788 3.039708
C 4.491092 -0.700150 3.455665
C 5.222522 -2.623214 2.317137
C 3.765340 -2.612762 2.303948
C 3.320031 -1.411455 2.975908
C 5.675663 -1.436788 -3.039708
C 5.222522 -2.623214 -2.317137
C 4.491092 -0.700150 -3.455665
C 3.320031 -1.411455 -2.975908
C 3.765340 -2.612762 -2.303948
C 5.675663 1.436788 -3.039708
C 4.491092 0.700150 -3.455665
C 5.222522 2.623214 -2.317137
C 3.765340 2.612762 -2.303948
C 3.320031 1.411455 -2.975908
C 7.113783 2.327618 -0.732140
C 5.939165 3.060636 -1.182343
C 7.113783 2.327618 0.732140
C 5.939165 3.060636 1.182343
C 5.207410 3.505435 0.000000
C 7.996618 -0.000000 0.705610
C 7.549786 1.182344 1.434933
C 7.549786 -1.182344 1.434933
C 6.819331 -0.731942 2.614519
C 6.819331 0.731942 2.614519
C 7.113783 -2.327618 -0.732140
C 7.113783 -2.327618 0.732140
C 5.939165 -3.060636 -1.182343
C 5.207410 -3.505435 -0.000000
C 5.939165 -3.060636 1.182343
C 6.819331 0.731942 -2.614519
C 6.819331 -0.731942 -2.614519
C 7.549786 1.182344 -1.434933
C 7.996618 -0.000000 -0.705610
C 7.549786 -1.182344 -1.434933
C -1.272247 -1.397870 -1.563539
C -1.807832 -2.438671 -0.754338
C -1.807832 -2.438671 0.754338
C -1.272247 -1.397870 1.563539
C -1.272247 1.397870 1.563539
C -1.807832 2.438671 0.754338
C -1.807832 2.438671 -0.754338
C -1.272247 1.397870 -1.563539
C -7.549786 -1.182344 -1.434933
C -7.996618 -0.000000 -0.705610
C -6.819331 -0.731942 -2.614519
C -6.819331 0.731942 -2.614519
C -7.549786 1.182344 -1.434933
C -5.939165 3.060636 -1.182343
C -7.113783 2.327618 -0.732140
C -5.207410 3.505435 -0.000000
C -5.939165 3.060636 1.182343
C -7.113783 2.327618 0.732140
C -6.819331 0.731942 2.614519
C -7.549786 1.182344 1.434933
C -6.819331 -0.731942 2.614519
C -7.549786 -1.182344 1.434933
C -7.996618 0.000000 0.705610
C -5.939165 -3.060636 1.182343
C -7.113783 -2.327618 0.732140
C -5.207410 -3.505435 0.000000
C -5.939165 -3.060636 -1.182343
C -7.113783 -2.327618 -0.732140
C -3.765340 -2.612762 -2.303948
C -5.222522 -2.623214 -2.317137
C -3.320031 -1.411455 -2.975908
C -4.491092 -0.700150 -3.455665
C -5.675663 -1.436788 -3.039708
C -3.320031 1.411455 -2.975908
C -4.491092 0.700150 -3.455665
C -3.765340 2.612762 -2.303948
C -5.222522 2.623214 -2.317137
C -5.675663 1.436788 -3.039708
C -3.320031 1.411455 2.975908
C -3.765340 2.612762 2.303948
C -4.491092 0.700150 3.455665
C -5.675663 1.436788 3.039708
C -5.222522 2.623214 2.317137
C -3.320031 -1.411455 2.975908
C -4.491092 -0.700150 3.455665
C -3.765340 -2.612762 2.303948
C -5.222522 -2.623214 2.317137
C -5.675663 -1.436788 3.039708
C -3.052089 -3.055758 1.165666
C -3.052089 -3.055758 -1.165666
C -3.800525 -3.491197 -0.000000
C -2.161944 0.734819 -2.517879
C -2.161944 -0.734819 -2.517879
C -3.052089 3.055758 1.165666
C -3.800525 3.491197 0.000000
C -3.052089 3.055758 -1.165666
C -2.161944 -0.734819 2.517879
C -2.161944 0.734819 2.517879

Iso v1
E = -4568.966867
c -2.392770 0.234153 2.262075
c -2.392770 0.234153 -2.262075
c 2.389424 -0.233807 7.569844
c 2.389424 -0.233807 -7.569844
c 0.215706 -1.228213 -8.258456
c 0.495164 -2.392436 -7.515670
c 1.750400 -2.500868 -6.773714
c 2.678985 -1.438054 -6.800743
c 1.176817 -0.130510 -8.288279
c 1.471211 -3.196386 -5.524040
c 2.127827 -2.810445 -4.337050
c 3.090408 -1.710189 -4.364079
c 3.360059 -1.037667 -5.572871
c 3.490048 0.416524 -5.583602
c 3.345641 1.144869 -4.385987
c 3.068165 0.447558 -3.134231
c 2.942859 -0.955695 -3.124027
c 1.890831 -1.593467 -2.333204
c 0.999770 -0.794433 -1.587231
c 1.130060 0.656293 -1.596535
c 2.148861 1.273477 -2.353494
c 2.594238 2.398450 -4.377002
c 1.858230 2.476981 -3.120026
c 1.391840 -2.739412 -3.080528
c 0.011720 -3.055127 -3.054484
c -0.908337 -2.229642 -2.281479
c -0.425828 -1.118826 -1.563658
c -1.176976 0.130502 -1.549802
c -0.215545 1.227313 -1.581399
c -0.495464 2.394218 -2.316897
c 0.556380 3.031992 -3.101679
c 2.888460 0.912567 -6.818333
c 2.163287 2.122416 -6.808777
c 2.013948 2.876062 -5.570010

c 0.668178 3.449272 -5.550201
c -0.044511 3.520791 -4.335174
c -2.888945 -0.912671 -3.014214
c -2.164924 -2.123518 -3.023544
c -2.014735 -2.875582 -4.262119
c -0.667781 -3.448375 -4.281769
c 0.044874 -3.521576 -5.496640
c -0.556156 -3.030725 -6.729754
c -1.857603 -2.476781 -6.711313
c -2.595865 -2.399617 -5.455937
c -3.488727 -0.417029 -4.250088
c -3.346074 -1.146113 -5.447304
c -1.130010 -0.656413 -8.241049
c -2.146250 -1.271721 -7.478303
c -3.066940 -0.447635 -6.698463
c -2.679315 1.438063 -3.031550
c -1.751757 2.502350 -3.058209
c -1.471883 3.195921 -4.307803
c 0.426135 1.119714 -8.276821
c 0.907750 2.227864 -7.551270
c -0.011808 3.054069 -6.776905
c -1.391142 2.738931 -6.750650
c -2.129050 2.811762 -5.495572
c -3.358486 1.037692 -4.260786
c -3.090311 1.711336 -5.468979
c -2.941437 0.955567 -6.708706
c -1.888825 1.591489 -7.498833
c -0.999821 0.794735 -8.251011
c -0.215545 1.227313 1.581399
c -0.495464 2.394218 2.316897
c -1.751757 2.502350 3.058209
c -2.679315 1.438063 3.031550
c -1.176976 0.130502 1.549802
c -3.358486 1.037692 4.260786
c -3.090311 1.711336 5.468979
c -2.129050 2.811762 5.495572
c -1.471883 3.195921 4.307803
c -2.941437 0.955567 6.708706
c -3.066940 -0.447635 6.698463
c -3.346074 -1.146113 5.447304
c -3.488727 -0.417029 4.250088
c -2.888945 -0.912671 3.014214
c -2.164924 -2.123518 3.023544
c -2.014735 -2.875582 4.262119
c -2.595865 -2.399617 5.455937
c -0.908337 -2.229642 2.281479
c 0.011720 -3.055127 3.054484
c -0.667781 -3.448375 4.281769
c 0.044874 -3.521576 5.496640
c 1.471211 -3.196386 5.524040
c 2.127827 -2.810445 4.337050
c 1.391840 -2.739412 3.080528
c 1.890831 -1.593467 2.333204
c 0.999770 -0.794433 1.587231
c -0.425828 -1.118826 1.563658
c -0.556156 -3.030725 6.729754
c -1.857603 -2.476781 6.711313
c -2.146250 -1.271721 7.478303
c -1.391142 2.738931 6.750650
c -1.888825 1.591489 7.498833
c -0.999821 0.794735 8.251011
c -1.130010 -0.656413 8.241049
c -0.011808 3.054069 6.776905
c 0.668178 3.449272 5.550201
c -0.044511 3.520791 4.335174
c 0.556380 3.031992 3.101679
c 0.907750 2.227864 7.551270
c 0.426135 1.119714 8.276821
c 0.495164 -2.392436 7.515670
c 0.215706 -1.228213 8.258456
c 1.176817 -0.130510 8.288279
c 2.888460 0.912567 6.818333
c 2.163287 2.122416 6.808777
c 2.013948 2.876062 5.570010
c 2.594238 2.398450 4.377002
c 1.858230 2.476981 3.120026
c 2.678985 -1.438054 6.800743
c 1.750400 -2.500868 6.773714
c 3.360059 -1.037667 5.572871
c 3.490048 0.416524 5.583602
c 3.345641 1.144869 4.385987
c 3.068165 0.447558 3.134231
c 2.148861 1.273477 2.353494
c 1.130060 0.656293 1.596535
c 2.942859 -0.955695 3.124027
c 3.090408 -1.710189 4.364079

Iso v2
E = -4568.966311
c -2.271901 -2.385651 0.341191
c 2.275069 0.014938 -2.393368
c -7.569256 2.382458 -0.340470
c 7.566685 -0.015000 2.391406
c 8.268935 -1.205431 0.325493
c 7.528251 -2.344003 0.713528
c 6.784093 -2.338182 1.966503
c 6.802981 -1.193227 2.794747
c 8.286495 -0.021032 1.180644
c 5.532828 -3.057961 1.751971
c 4.345868 -2.612355 2.368716
c 4.366313 -1.426613 3.225562
c 5.576399 -0.732030 3.431502
c 5.586151 0.731179 3.430154
c 4.385194 1.441433 3.222860
c 3.134276 0.723661 3.015330
c 3.124852 -0.692689 3.016692
c 2.340603 -1.415873 2.024167
c 1.595909 -0.707587 1.060876
c 1.605834 0.754411 1.059152
c 2.359666 1.454980 2.020968
c 4.380678 2.625057 2.362888
c 3.126581 2.634534 1.619351
c 3.091872 -2.606623 1.625252
c 3.063481 -3.043065 0.285230
c 2.281877 -2.313798 -0.709548
c 1.561248 -1.161711 -0.323226
c 1.562105 0.020937 -1.179957
c 1.577627 1.205477 -0.325957
c 2.312853 2.347130 -0.714856
c 3.104011 3.068360 0.278434
c 6.818485 1.174336 2.792056
c 6.814981 2.317389 1.961130
c 5.573199 3.053328 1.745028
c 5.550495 3.503867 0.356153
c 4.338847 3.510302 -0.363215
c 3.022409 -1.175041 -2.793575
c 3.026035 -2.317790 -1.961015
c 4.269535 -3.051584 -1.744423
c 4.292419 -3.502949 -0.355251
c 5.504069 -3.511257 0.364120
c 6.737995 -3.067558 -0.278359
c 6.715392 -2.632595 -1.618771
c 5.461205 -2.624378 -2.363620
c 4.255014 -0.731713 -3.429590
c 5.456427 -1.442004 -3.223936
c 8.244436 -0.754894 -1.060266
c 7.482162 -1.453588 -2.019047
c 6.706575 -0.723219 -3.014438
c 3.037987 1.193827 -2.796110
c 3.056877 2.338679 -1.966526
c 4.309872 3.056186 -1.751409
c 8.284927 1.161438 0.322857
c 7.559200 2.310740 0.708336
c 6.778517 3.042299 -0.285144
c 6.750049 2.604742 -1.624665
c 5.495942 2.611687 -2.369465
c 4.264808 0.732504 -3.430919
c 5.475303 1.427184 -3.226662
c 6.716056 0.692393 -3.015896
c 7.501245 1.414673 -2.022318
c 8.254248 0.708146 -1.061852
c -1.648155 -0.599029 -1.274219
c -2.440422 -1.196864 -2.273852
c -3.183101 -2.421502 -1.973866
c -3.100508 -3.000461 -0.690524
c -1.559517 -1.200397 0.050686
c -4.304827 -3.522944 -0.050792
c -5.546069 -3.449326 -0.713781
c -5.630748 -2.848412 -2.042258
c -4.467220 -2.342900 -2.658426
c -6.747735 -3.073706 0.025561
c -6.665135 -2.786058 1.402561
c -5.380351 -2.863165 2.089682
c -4.219680 -3.224777 1.376513
c -2.962734 -2.521519 1.616423
c -2.915266 -1.475831 2.565544
c -4.115557 -1.100205 3.299556
c -5.329953 -1.780856 3.070917
c -2.176372 -0.251293 2.264314
c -2.910867 0.878757 2.824843
c -4.113232 0.355391 3.458487
c -5.326044 1.071874 3.382290
c -5.374262 2.342355 2.659235
c -4.211771 2.846938 2.041576
c -2.956617 2.107233 2.124479
c -2.267843 2.246952 0.849590
c -1.558755 1.152487 0.307965
c -1.517477 -0.117647 1.028075
c -6.580017 0.367806 3.147554
c -6.582450 -1.039455 2.993702
c -7.405690 -1.655868 1.960712
c -6.884152 -2.106503 -2.124342
c -7.573479 -2.244159 -0.848010
c -8.288358 -1.152517 -0.307746
c -8.202495 -0.854896 1.115933
c -6.930262 -0.878326 -2.823592
c -5.728090 -0.354972 -3.458785
c -4.515380 -1.071333 -3.381042
c -3.260747 -0.368027 -3.148455
c -7.666401 0.251166 -2.262753
c -8.333928 0.117614 -1.029238
c -7.401034 1.195879 2.271853
c -8.199873 0.599374 1.274823
c -8.287004 1.200099 -0.050581
c -6.878512 2.521053 -1.616459
c -6.926253 1.474858 -2.564249
c -5.725856 1.099518 -3.299657
c -4.511855 1.779893 -3.069446
c -3.258361 1.039872 -2.994176
c -6.741462 2.999979 0.690443
c -6.658424 2.419650 1.972908
c -5.538211 3.524102 0.050207
c -5.623146 3.226415 -1.376698
c -4.461610 2.863432 -2.088517
c -3.176677 2.787869 -1.402852
c -2.435640 1.657539 -1.962632
c -1.645420 0.854578 -1.115288
c -3.094432 3.074522 -0.025549
c -4.296849 3.448869 0.712910

Iso v3
E = -4568.966159
C -3.537655 0.282685 -3.297076
C -7.715591 -1.125704 -1.848369
C -7.043724 -0.348839 -2.817284
C -6.980522 1.103832 -2.665303
C -7.589403 1.725787 -1.557125
C -8.284648 0.920640 -0.555349
C -8.344296 -0.483194 -0.702780
C -5.774006 -0.802127 -3.367914
C -4.921067 0.367730 -3.552450
C -5.668175 1.548246 -3.123296
C -5.208461 -2.022995 -2.930301
C -3.778736 -2.111618 -2.666623
C -2.952488 -0.980770 -2.846110

C -2.852233 1.370367 -2.608160
C -1.851238 0.777511 -1.726088
C -1.908021 -0.674442 -1.879565
C -3.584729 -2.974008 -1.501875
C -2.575633 -2.676018 -0.564116
C -1.722212 -1.507523 -0.753283
C -1.471860 -0.919168 0.555334
C -1.417076 0.482063 0.703314
C -1.610706 1.344257 -0.460221
C -2.349129 2.526426 -0.031613
C -3.317264 3.101245 -0.885103
C -3.572884 2.512332 -2.194034
C -5.006362 2.598602 -2.455407
C -4.897102 -3.418544 -1.045116
C -5.901065 -2.825123 -1.926158
C -7.130780 -2.386987 -1.394867
C -7.402418 -2.525778 0.031504
C -6.432361 -3.098534 0.883556
C -5.158862 -3.551788 0.334441
C -2.846267 -2.814160 0.865629
C -4.113438 -3.244704 1.306776
C -2.162679 -1.725105 1.557411
C -4.742934 -2.599171 2.455767
C -4.081440 -1.548264 3.123878
C -2.768620 -1.103860 2.667670
C -2.037054 1.125050 1.849017
C -2.705406 0.348850 2.821293
C -2.620592 2.387706 1.395161
C -3.975048 0.802155 3.369416
C -4.540659 2.023895 2.931651
C -3.848868 2.826736 1.927590
C -4.590971 3.552250 -0.334889
C -4.852891 3.418869 1.044922
C -5.636691 3.243312 -1.306228
C -6.905089 2.814860 -0.865771
C -6.164521 2.972586 1.500165
C -7.175058 2.676310 0.563374
C -8.030330 1.508662 0.752714
C -7.840487 0.674453 1.878932
C -6.796722 0.980338 2.846848
C -5.969544 2.109809 2.665468
C -7.898762 -0.777433 1.725865
C -6.896998 -1.369877 2.608596
C -6.212045 -0.282717 3.298156
C -4.828562 -0.367792 3.552293
C -6.175933 -2.510066 2.192911
C -8.146227 -1.345684 0.460938
C 2.753188 1.833735 -2.202961
C 6.980523 0.048712 -2.883151
C 6.166423 1.177706 -3.112403
C 6.307952 2.360903 -2.262673
C 7.256300 2.369707 -1.219006
C 8.098579 1.202538 -0.984194
C 7.961239 0.058730 -1.805690
C 4.753907 1.012155 -3.428336
C 4.019693 2.085627 -2.766079
C 4.980916 2.924341 -2.050843
C 4.192321 -0.284996 -3.502057
C 2.882341 -0.546336 -2.922081
C 2.172449 0.492937 -2.281287
C 2.398075 2.410187 -0.909887
C 1.614280 1.420108 -0.183879
C 1.470619 0.237690 -1.033680
C 2.908851 -1.878977 -2.322774
C 2.226506 -2.125463 -1.115005
C 1.492967 -1.048258 -0.456247
C 1.656121 -1.202012 0.984293
C 1.789228 -0.059176 1.806174
C 1.768981 1.272242 1.207897
C 2.727694 2.110646 1.924114
C 3.493115 3.066842 1.221882
C 3.326457 3.222328 -0.217753
C 4.638713 3.479079 -0.799087
C 4.239062 -2.441744 -2.534566
C 5.033029 -1.453058 -3.261189
C 6.400703 -1.292495 -2.959298
C 7.022159 -2.110414 -1.924828
C 6.255980 -3.064361 -1.221886
C 4.839258 -3.232076 -1.534107
C 2.848070 -2.944066 -0.078089
C 4.130655 -3.489196 -0.283289
C 2.495028 -2.369871 1.219181
C 5.111194 -3.478687 0.799483
C 4.769456 -2.924288 2.051288
C 3.441783 -2.361812 2.264595
C 2.769787 -0.049285 2.882387
C 3.582482 -1.179243 3.114181
C 3.349758 1.292244 2.958176
C 4.995535 -1.012081 3.427580
C 5.556625 0.284617 3.501013
C 4.716880 1.453248 3.261518
C 4.910282 3.231731 1.533874
C 5.510604 2.441628 2.534160
C 5.619749 3.487612 0.283071
C 6.903300 2.944283 0.077570
C 6.840121 1.878080 2.320275
C 7.524570 2.125156 1.113845
C 8.261737 1.048695 0.455676
C 8.285683 -0.238955 1.033397
C 7.578570 -0.494231 2.280864
C 6.866291 0.544854 2.918356
C 8.141922 -1.421535 0.183964
C 7.353022 -2.410151 0.910425
C 6.998132 -1.834188 2.203280
C 5.730590 -2.085272 2.765267
C 6.423115 -3.219161 0.218444
C 7.981975 -1.272544 -1.208028

Iso p4
E = -4568.965684
C -0.279954 -0.083536 -1.940708
C 0.060827 1.238556 -1.635580
C 0.836425 -1.014168 -2.096961
C 1.947388 -0.539222 -2.856963
C 2.243145 0.887558 -2.785147
C 1.463770 1.667025 -1.794300
C 3.536241 1.291518 -3.193336
C 4.181162 2.399779 -2.540764
C 3.535046 2.994836 -1.437905
C 2.203011 2.603895 -0.994161
C -1.030745 2.135798 -1.188527
C -1.327366 2.506131 0.159766
C -1.005308 1.670812 1.293310
C 0.292170 1.207717 1.537502
C 1.515156 2.030520 1.483015
C 2.196107 2.795321 0.504712
C 1.017917 -2.071002 -1.185960
C 0.087833 -2.263121 -0.084395
C -1.274883 -2.455136 -0.402974
C -1.921240 -2.003953 -1.664856
C -1.603547 -0.670039 -2.190103
C 2.998119 -1.461947 -3.097336
C 4.311780 -1.036973 -3.510807
C 4.574981 0.339848 -3.586600
C 2.286036 -2.729023 -1.088325
C 3.157091 -2.591093 -2.202375
C 0.687390 -2.164190 1.253392
C 2.089012 -2.476204 1.416029
C 2.811520 -3.048631 0.235835
C 4.189724 -3.386075 0.296540
C 5.067321 -3.306899 -0.867372
C 4.563514 -2.897142 -2.109693
C -0.134177 -1.216339 2.100282
C -1.513832 -1.254412 2.315635
C -2.290175 -2.418190 1.904585
C -2.184331 -2.972318 0.591562
C 2.961748 -2.025839 2.504908
C 4.387381 -2.355729 2.515045
C 4.993557 -3.045346 1.457181
C 0.596562 0.024271 2.315739
C 1.848091 0.193311 2.923932
C 2.840462 -0.775561 3.232413
C 2.288000 1.549193 2.625074
C 3.535084 3.289057 0.874243
C 4.194597 2.935516 2.080373
C 3.550949 2.002105 2.960898
C 4.175260 -0.301692 3.593178
C 4.528755 1.065752 3.498507
C -2.340300 -0.065125 2.746111
C -2.121220 1.292644 2.212989
C 4.341404 3.433927 -0.313313
C 5.737561 3.220428 -0.287290
C 6.387027 2.783278 0.943211
C 5.619334 2.634976 2.109801
C 5.620099 2.151098 -2.517759
C 6.384347 2.537806 -1.401285
C 5.860053 0.857925 -3.149300
C 6.834083 -0.021671 -2.614048
C 7.607114 0.374570 -1.445607
C 7.399386 1.642586 -0.857646
C 5.298100 -1.943168 -2.928747
C 6.537704 -1.447099 -2.490609
C 6.379374 -2.845465 -0.435176
C 7.098200 -1.921188 -1.227207
C 5.138196 -1.303626 3.189236
C 6.337717 -2.694597 1.013841
C 7.036910 -1.635694 1.629273
C 6.424290 -0.926468 2.743841
C 5.822255 1.467665 2.981277
C 6.773503 0.486206 2.625726
C 7.394565 1.785128 0.594416
C 7.574754 0.651801 1.418109
C 7.782077 -0.799657 -0.591979
C 7.756447 -0.664666 0.811110
C -3.638946 -2.645354 2.366958
C -4.294899 -1.650353 3.101105
C -3.637625 -0.345223 3.278383
C -3.462554 -3.541822 0.232357
C -4.374874 -3.390499 1.351608
C -3.172249 -2.586378 -2.008026
C -3.938888 -3.410478 -1.081605
C -2.211430 2.435662 -1.976378
C -2.875494 1.517786 -2.844894
C -2.604356 0.063669 -2.899023
C -2.542045 3.286852 0.160438
C -3.074690 3.325153 -1.178924
C -3.177703 2.245977 2.303635
C -3.396970 3.255040 1.261172
C -7.254698 2.061417 0.440341
C -7.838668 0.761130 0.120103
C -6.707274 1.980620 1.792371
C -6.955024 0.633544 2.302402
C -7.670270 -0.114744 1.274257
C -7.707454 0.237976 -1.184445
C -7.417710 -1.181132 -1.371371
C -7.284558 -2.037315 -0.255682
C -7.409770 -1.492048 1.097866
C -5.979037 -0.015363 3.085037
C -5.723003 -1.438630 2.926834
C -6.443014 -2.171424 1.947577
C -6.581532 2.807920 -0.551696
C -6.429481 2.256436 -1.899537
C -6.979979 0.991923 -2.199544
C -5.499101 2.633772 2.095177
C -4.470204 1.928783 2.853215
C -4.699152 0.636726 3.335180
C -5.356188 3.524970 -0.221309
C -4.823357 3.438703 1.088241
C -5.103300 2.619521 -2.384477
C -4.460032 3.429875 -1.365289
C -4.313974 1.680744 -3.090147
C -6.204764 0.044571 -2.987011
C -4.896342 0.380866 -3.388258
C -6.488752 -1.300830 -2.489667
C -5.469565 -2.268729 -2.453309

C -4.115737 -1.902989 -2.853672
C -3.836113 -0.596729 -3.280967
C -6.252556 -3.065359 -0.235570
C -5.355280 -3.184279 -1.322757
C -5.741937 -3.156311 1.131817

Iso c3
E = -4568.959473
C 3.650014 3.562115 0.000332
C 2.910662 3.110953 -1.172267
C 1.707845 2.456000 -0.745008
C 1.707842 2.455866 0.745465
C 2.910668 3.110731 1.172843
C 1.200355 1.335989 -1.425502
C -0.013037 0.667111 -0.824061
C -0.013034 0.666954 0.824171
C 1.200362 1.335719 1.425743
C 1.266791 -1.494572 1.438786
C 1.828940 -2.581882 0.747897
C 1.828940 -2.581739 -0.748398
C 1.266789 -1.494299 -1.439082
C 3.067732 -3.176148 1.171444
C 3.738267 -2.651454 2.293409
C 3.227792 -1.447710 2.926949
C 2.052953 -0.798764 2.438799
C 2.012897 0.686538 2.435935
C 3.827803 -3.589796 -0.000347
C 3.067731 -3.175923 -1.172060
C 2.052950 -0.798300 -2.438964
C 3.227789 -1.447154 -2.927238
C 3.738264 -2.651016 -2.293925
C 5.194547 -2.607658 -2.314382
C 5.931824 -3.032153 -1.184525
C 5.230217 -3.522522 -0.000343
C 5.931825 -3.032377 1.183933
C 5.194550 -2.608098 2.313872
C 5.593892 -1.400597 -3.025757
C 4.376574 -0.697419 -3.415748
C 4.340024 0.700163 -3.411592
C 3.155925 1.393697 -2.926282
C 2.012893 0.687000 -2.435816
C 6.724299 -0.669527 -2.608291
C 7.475408 -1.102085 -1.435075
C 7.081212 -2.260745 -0.732880
C 7.081213 -2.260885 0.732433
C 7.475412 -1.102358 1.434849
C 6.724303 -0.670023 2.608146
C 5.593895 -1.401169 3.025472
C 4.376577 -0.698066 3.415598
C 3.155928 1.393143 2.926532
C 4.340027 0.699517 3.411708
C 3.606794 2.623515 -2.297944
C 3.606795 2.623085 2.298432
C 5.053925 3.566063 0.000331
C 5.778928 3.113615 -1.184695
C 5.061559 2.651101 -2.314142
C 5.521925 1.463796 -3.023087
C 6.687235 0.790762 -2.607974
C 6.961946 2.396813 -0.732980
C 7.418363 1.261534 -1.435377
C 7.894954 0.091368 -0.705567
C 7.894957 0.091234 0.705567
C 6.961946 2.396672 0.733419
C 7.418365 1.261262 1.435599
C 6.687236 0.790266 2.608106
C 5.521930 1.463223 3.023349
C 5.061562 2.650661 2.314630
C 5.778929 3.113392 1.185272
C 0.051519 -0.878516 0.810124
C -1.184314 1.376791 1.431939
C -1.720192 2.489936 0.721849
C -1.720195 2.490060 -0.721383
C -1.184325 1.377062 -1.431693
C 0.051518 -0.878366 -0.810302
C -2.110963 1.007900 2.486936
C -2.498261 -0.301947 2.901045
C -2.093754 -1.560420 2.213711
C -1.169770 -1.703638 1.167480
C -1.556961 -2.469297 -0.000232
C -1.169778 -1.703412 -1.167812
C -2.901048 2.972487 -1.421339
C -3.990551 3.539709 -0.732995
C -3.990550 3.539574 0.733678
C -2.901033 2.972222 1.421906
C -3.126333 2.081693 2.544504
C -4.435041 1.863019 3.001079
C -5.569468 2.473539 2.316820
C -5.346857 3.284690 1.181655
C -5.346870 3.284912 -1.181013
C -6.193739 3.133998 0.000311
C -3.869706 -0.508390 3.372285
C -4.821888 0.535501 3.439895
C -6.201677 0.309190 3.031316
C -6.657211 1.502598 2.316165
C -7.483924 1.369048 1.182010
C -7.241488 2.194587 0.000225
C -3.126353 2.082176 -2.544103
C -2.110978 1.008375 -2.486757
C -2.498289 -0.301394 -2.901105
C -2.093770 -1.559987 -2.214002
C -3.244675 -2.463534 2.240136
C -3.534300 -3.343761 1.173996
C -2.695943 -3.289860 -0.000308
C -3.534308 -3.343541 -1.174623
C -3.244695 -2.463103 -2.240588
C -4.435067 1.863589 -3.000719
C -4.821917 0.536157 -3.439774
C -3.869727 -0.507750 -3.372372
C -4.325217 -1.831630 -2.966473
C -5.666344 -2.062939 -2.592315
C -5.960774 -2.906193 -1.432897
C -4.909517 -3.530369 -0.733525
C -4.909514 -3.530508 0.732874
C -5.960763 -2.906462 1.432372
C -5.666322 -2.063428 2.591948
C -4.325198 -1.832190 2.966144
C -5.569485 2.473974 -2.316325
C -7.483932 1.369272 -1.181709
C -6.657234 1.503037 -2.315851
C -6.201697 0.309765 -3.031230
C -6.625215 -0.967484 -2.611357
C -7.895512 0.042576 -0.728516
C -7.484606 -1.105980 -1.438525
C -7.067851 -2.298412 -0.708741
C -7.067845 -2.298546 0.708342
C -7.895505 0.042437 0.728566
C -7.484604 -1.106257 1.438357
C -6.625195 -0.967979 2.611205

Iso c4
E = -4568.954625
C -0.021583 0.840626 0.000000
C 0.021583 -0.840626 0.000000
C -3.234938 4.129929 1.436230
C -3.037590 2.931084 0.726316
C -3.037590 2.931084 -0.726316
C -3.234938 4.129929 -1.436230
C -3.435915 5.377785 -0.704691
C -3.435915 5.377785 0.704691
C -2.016857 1.970612 1.163459
C -1.421971 1.373239 0.000000
C -2.016857 1.970612 -1.163459
C -1.263075 2.236553 2.332243
C 0.180784 1.950994 2.311099
C 0.779689 1.382748 1.185194
C 0.779689 1.382748 -1.185194
C 2.059605 1.868654 -0.730509
C 2.059605 1.868654 0.730509
C 0.863082 3.013019 3.040163
C 2.132146 3.467660 2.616692
C 2.748081 2.880257 1.440276
C 3.432091 3.942042 0.704267
C 3.432091 3.942042 -0.704267
C 2.748081 2.880257 -1.440276
C 2.132146 3.467660 -2.616692
C 0.863082 3.013019 -3.040163
C 0.180784 1.950994 -2.311099
C -1.263075 2.236553 -2.332243
C -0.143875 3.964317 3.488221
C -1.455299 3.483824 3.043112
C -2.433447 4.419420 2.621176
C -2.125678 5.840591 2.616866
C -0.864713 6.303821 3.057290
C 0.142551 5.343960 3.496821
C 2.428446 4.895574 2.617372
C 1.451129 5.817287 3.046761
C 3.234216 5.190396 1.434266
C 1.252338 7.064798 2.321312
C 2.031592 7.350288 1.180137
C 3.040205 6.396144 0.730280
C 3.234216 5.190396 -1.434266
C 3.040205 6.396144 -0.730280
C 2.428446 4.895574 -2.617372
C 2.031592 7.350288 -1.180137
C 1.252338 7.064798 -2.321312
C 1.451129 5.817287 -3.046761
C -0.143875 3.964317 -3.488221
C 0.142551 5.343960 -3.496821
C -1.455299 3.483824 -3.043112
C -2.433447 4.419420 -2.621176
C -0.864713 6.303821 -3.057290
C -2.125678 5.840591 -2.616866
C -2.746989 6.437358 -1.435203
C -2.086499 7.462424 -0.730159
C -0.778635 7.933716 -1.180454
C -0.180782 7.359571 -2.320468
C -2.086499 7.462424 0.730159
C -0.778635 7.933716 1.180454
C 0.028142 8.222957 0.000000
C 1.412382 7.943838 0.000000
C -0.180782 7.359571 2.320468
C -2.746989 6.437358 1.435203
C 2.016857 -1.970612 1.163459
C 3.037590 -2.931084 0.726316
C 3.037590 -2.931084 -0.726316
C 2.016857 -1.970612 -1.163459
C 1.421971 -1.373239 0.000000
C 3.234938 -4.129929 1.436230
C 2.433447 -4.419420 2.621176
C 1.455299 -3.483824 3.043112
C 1.263075 -2.236553 2.332243
C -0.180784 -1.950994 2.311099
C -0.779689 -1.382748 1.185194
C 3.435915 -5.377785 0.704691
C 3.435915 -5.377785 -0.704691
C 3.234938 -4.129929 -1.436230
C 2.433447 -4.419420 -2.621176
C 1.455299 -3.483824 -3.043112
C 1.263075 -2.236553 -2.332243
C -0.779689 -1.382748 -1.185194
C -0.180784 -1.950994 -2.311099
C 0.143875 -3.964317 3.488221
C -0.863082 -3.013019 3.040163
C -2.059605 -1.868654 0.730509
C -2.748081 -2.880257 1.440276
C -2.132146 -3.467660 2.616692
C -2.428446 -4.895574 2.617372
C -1.451129 -5.817287 3.046761
C -0.142551 -5.343960 3.496821
C 2.125678 -5.840591 2.616866
C 0.864713 -6.303821 3.057290
C 2.746989 -6.437358 1.435203
C 0.180782 -7.359571 2.320468
C 0.778635 -7.933716 1.180454
C 2.086499 -7.462424 0.730159

C 2.746989 -6.437358 -1.435203
C 2.086499 -7.462424 -0.730159
C 2.125678 -5.840591 -2.616866
C 0.778635 -7.933716 -1.180454
C 0.180782 -7.359571 -2.320468
C 0.864713 -6.303821 -3.057290
C 0.143875 -3.964317 -3.488221
C -0.142551 -5.343960 -3.496821
C -2.059605 -1.868654 -0.730509
C -0.863082 -3.013019 -3.040163
C -2.132146 -3.467660 -2.616692
C -2.748081 -2.880257 -1.440276
C -3.432091 -3.942042 -0.704267
C -3.432091 -3.942042 0.704267
C -1.451129 -5.817287 -3.046761
C -2.428446 -4.895574 -2.617372
C -3.234216 -5.190396 -1.434266
C -3.040205 -6.396144 -0.730280
C -2.031592 -7.350288 -1.180137
C -1.252338 -7.064798 -2.321312
C -3.040205 -6.396144 0.730280
C -2.031592 -7.350288 1.180137
C -1.412382 -7.943838 0.000000
C -0.028142 -8.222957 0.000000
C -1.252338 -7.064798 2.321312
C -3.234216 -5.190396 1.434266

Iso c5
E = -4568.954381
C 1.390742 -2.239701 -0.953067
C 0.273697 -1.317647 -1.193436
C 2.266563 -2.049863 -2.103088
C 2.035117 -0.703683 -2.611958
C 0.808845 -0.244055 -2.025900
C 2.650745 2.273389 -2.093527
C 1.374325 2.042871 -1.431919
C 3.436213 3.192495 -1.244986
C 2.759742 3.287599 0.025617
C 1.516605 2.552209 -0.062701
C 2.054316 1.526290 2.121341
C 1.100798 1.786013 1.059847
C 2.163575 0.138780 2.589384
C 1.337048 -0.882968 1.918240
C 0.009738 -0.377775 1.554872
C -0.106880 0.990059 1.181893
C 3.244619 -2.447427 2.194767
C 1.969168 -2.119137 1.572817
C 4.046167 -3.270372 1.306512
C 3.296051 -3.420486 0.078181
C 1.983636 -2.761150 0.221207
C 5.462946 -3.286193 -1.170835
C 4.015093 -3.445744 -1.141069
C 5.804974 -2.419464 -2.305363
C 4.563746 -2.032244 -2.954537
C 3.484280 -2.697823 -2.258686
C 5.451110 0.246695 -3.312733
C 4.371161 -0.710113 -3.415067
C 4.860974 1.554507 -3.047386
C 3.403718 1.416668 -2.964598
C 3.110655 -0.001908 -3.194581
C 5.502476 2.604700 2.264858
C 4.963888 3.406544 1.168999
C 4.370280 1.993765 2.949453
C 3.155266 2.407372 2.287575
C 3.513831 3.319891 1.213457
C 5.705127 -0.062972 3.312532
C 4.464182 0.684375 3.445531
C 5.353958 -1.463436 3.088909
C 3.900035 -1.562664 3.073158
C 3.355596 -0.235059 3.255263
C 7.250191 -1.646595 1.496297
C 6.124753 -2.244335 2.204196
C 7.274004 -2.204059 0.147406
C 6.179974 -3.164874 0.035290
C 5.456174 -3.172850 1.300014
C 7.933722 0.027318 -0.735393
C 7.607441 -1.379184 -0.949274
C 7.371881 0.796018 -1.839904
C 6.679292 -0.131083 -2.726058
C 6.849803 -1.481234 -2.192246
C 6.847885 2.646578 -0.261840
C 6.827854 2.076817 -1.609815
C 5.621235 3.411137 -0.078993
C 4.834583 3.289958 -1.303722
C 5.574721 2.466067 -2.240115
C 6.801775 0.516147 2.639962
C 6.691358 1.870474 2.094966
C 7.578616 -0.286371 1.703212
C 7.929062 0.562505 0.570517
C 7.382008 1.895680 0.809523
C -1.359772 1.681407 1.282499
C -1.866636 2.621658 0.327995
C -1.787577 2.412546 -1.115225
C -1.086952 1.323974 -1.781039
C 0.309667 1.091067 -1.817227
C -1.098516 -1.639831 -1.110524
C -1.697783 -2.510289 -0.135376
C -1.733830 -2.265467 1.317851
C -1.229105 -1.062793 1.905814
C -7.570663 1.330662 1.093942
C -7.963127 0.021655 0.581145
C -6.870532 1.123049 2.357352
C -6.840564 -0.314611 2.629410
C -7.520180 -0.993476 1.531544
C -5.881149 -2.857529 1.696929
C -7.045826 -2.241335 1.071657
C -5.101552 -3.506148 0.649045
C -5.779866 -3.285995 -0.623525
C -6.983694 -2.508388 -0.365003
C -6.627453 -1.268678 -2.491101
C -7.406932 -1.521098 -1.284208
C -6.660326 0.169719 -2.762506
C -7.461320 0.806846 -1.723434
C -7.907144 -0.237045 -0.804139
C -5.919565 2.762600 -1.768364
C -7.087457 2.076414 -1.230291
C -5.256171 3.453082 -0.670139
C -6.008829 3.189684 0.551367
C -7.145248 2.343604 0.207205
C -3.858808 2.998741 1.780191
C -5.315741 2.975663 1.761984
C -3.404138 1.933673 2.660090
C -4.564955 1.303707 3.241499
C -5.755911 1.922932 2.674527
C -3.344396 -0.837742 3.210140
C -4.536579 -0.076534 3.507717
C -3.753157 -2.165867 2.787045
C -5.211493 -2.199790 2.751121
C -5.699007 -0.901131 3.211398
C -3.106302 -1.867939 -2.540276
C -3.567380 -2.974149 -1.703382
C -4.259995 -1.313030 -3.204671
C -5.449201 -2.002051 -2.727590
C -5.014595 -3.032419 -1.786360
C -3.160844 0.882467 -3.088353
C -4.289726 0.063937 -3.464102
C -3.684697 2.200174 -2.709426
C -5.135387 2.144896 -2.772309
C -5.514666 0.821165 -3.260104
C -3.042043 2.894556 -1.671475
C -3.130058 3.289862 0.613320
C -3.848461 3.507004 -0.628674
C -2.183393 -0.238553 2.645662
C -2.237111 1.194826 2.334462
C -2.912846 -3.213567 -0.483580
C -3.694480 -3.475013 0.708897
C -2.991545 -2.825583 1.802720
C -1.995874 0.354724 -2.473412
C -1.989570 -1.080693 -2.161713

Iso c6
E = -4568.953141
C 3.468692 -3.833143 -0.000000
C 3.018402 -3.107373 -1.182029
C 2.284520 -1.930765 -0.742734
C 2.284520 -1.930765 0.742734
C 3.018402 -3.107373 1.182029
C 1.166277 -1.502892 -1.453315
C -0.021010 -0.807739 -0.800495
C -0.021010 -0.807739 0.800495
C 1.166277 -1.502892 1.453315
C -1.194714 -1.515406 1.454438
C -2.313180 -1.953118 0.740056
C -2.313180 -1.953118 -0.740056
C -1.194714 -1.515406 -1.454438
C -3.031068 -3.140772 1.180879
C -2.614293 -3.850917 2.329571
C -1.442681 -3.401273 3.061500
C -0.740986 -2.263844 2.600505
C 0.718862 -2.256764 2.601574
C -3.476971 -3.872611 -0.000000
C -3.031068 -3.140772 -1.180879
C -0.740986 -2.263844 -2.600505
C -1.442681 -3.401273 -3.061500
C -2.614293 -3.850917 -2.329571
C -2.612557 -5.308366 -2.325760
C -3.043589 -6.014065 -1.181955
C -3.490203 -5.282614 -0.000000
C -3.043589 -6.014065 1.181955
C -2.612557 -5.308366 2.325760
C -1.429096 -5.758450 -3.056183
C -0.704584 -4.575588 -3.512269
C 0.705713 -4.570929 -3.514255
C 1.431774 -3.386017 -3.059048
C 0.718862 -2.256764 -2.601574
C -0.718910 -6.894274 -2.618043
C -1.167608 -7.622930 -1.435861
C -2.309710 -7.188691 -0.731513
C -2.309710 -7.188691 0.731513
C -1.167608 -7.622930 1.435861
C -0.718910 -6.894274 2.618043
C -1.429096 -5.758450 3.056183
C -0.704584 -4.575588 3.512269
C 1.431774 -3.386017 3.059048
C 0.705713 -4.570929 3.514255
C 2.608767 -3.822302 -2.331294
C 2.608767 -3.822302 2.331294
C 3.494140 -5.242803 -0.000000
C 3.053138 -5.979276 -1.181536
C 2.617824 -5.280281 -2.326026
C 1.440925 -5.743724 -3.060498
C 0.742567 -6.889232 -2.619782
C 2.334297 -7.164865 -0.730025
C 1.195829 -7.609952 -1.437405
C 0.016820 -8.063298 -0.704641
C 0.016820 -8.063298 0.704641
C 2.334297 -7.164865 0.730025
C 1.195829 -7.609952 1.437405
C 0.742567 -6.889232 2.619782
C 1.440925 -5.743724 3.060498
C 2.617824 -5.280281 2.326026
C 3.053138 -5.979276 1.181536
C -0.009679 0.789512 0.813723
C 1.153084 1.478694 1.463671
C 2.273221 1.934169 0.719523
C 2.273221 1.934169 -0.719523
C 1.153084 1.478694 -1.463671
C -0.009679 0.789512 -0.813723
C 0.992315 2.346838 2.613155
C -0.284623 2.727416 3.074627
C -1.464459 2.290320 2.307768
C -1.324364 1.470799 1.184210
C -2.104142 1.747007 0.000000
C -1.324364 1.470799 -1.184210
C 2.883960 3.067094 -1.434032

C 3.457090 4.142822 -0.733612
C 3.457090 4.142822 0.733612
C 2.883960 3.067094 1.434032
C 2.080077 3.321233 2.618216
C 1.859501 4.640945 3.061379
C 2.446651 5.757287 2.331116
C 3.232502 5.508200 1.183417
C 3.232502 5.508200 -1.183417
C 3.086729 6.352263 0.000000
C -0.519890 4.094211 3.508316
C 0.532151 5.041872 3.518871
C 0.290689 6.403385 3.060390
C 1.475145 6.846848 2.323586
C 1.332910 7.657794 1.181431
C 2.155902 7.409212 0.000000
C 2.080077 3.321233 -2.618216
C 0.992315 2.346838 -2.613155
C -0.284623 2.727416 -3.074627
C -1.464459 2.290320 -2.307768
C -2.426588 3.382507 2.307149
C -3.235126 3.620803 1.175793
C -3.079245 2.784301 0.000000
C -3.235126 3.620803 -1.175793
C -2.426588 3.382507 -2.307149
C 1.859501 4.640945 -3.061379
C 0.532151 5.041872 -3.518871
C -0.519890 4.094211 -3.508316
C -1.846552 4.498850 -3.044200
C -2.080685 5.820495 -2.616329
C -2.906366 6.067978 -1.434614
C -3.478050 4.987866 -0.731155
C -3.478050 4.987866 0.731155
C -2.906366 6.067978 1.434614
C -2.080685 5.820495 2.616329
C -1.846552 4.498850 3.044200
C 2.446651 5.757287 -2.331116
C 1.332910 7.657794 -1.181431
C 1.475145 6.846848 -2.323586
C 0.290689 6.403385 -3.060390
C -0.993621 6.794856 -2.624297
C 0.002812 8.059251 -0.728171
C -1.140405 7.633926 -1.438951
C -2.320882 7.186326 -0.707064
C -2.320882 7.186326 0.707064
C 0.002812 8.059251 0.728171
C -1.140405 7.633926 1.438951
C -0.993621 6.794856 2.624297

Benchmarking calculations: B3LYP/def2-SVP

C60
E = -2284.269704
C 0.000000 -1.237586 3.332098
C -0.000000 -2.426853 2.597090
C 1.177015 -2.809289 1.832220
C 2.308075 -0.749939 2.597090
C 1.177015 -0.382435 3.332098
C 0.727435 1.001228 3.332098
C -0.727435 1.001228 3.332098
C -1.177015 -0.382435 3.332098
C -0.000000 1.237586 -3.332098
C 0.000000 2.426853 -2.597090
C -1.177015 2.809289 -1.832220
C -1.177015 0.382435 -3.332098
C -2.308075 1.987525 -1.832220
C -0.727435 -1.001228 -3.332098
C 0.727435 -1.001228 -3.332098
C 1.177015 0.382435 -3.332098
C -1.177015 -2.809289 1.832220
C 3.035510 -1.751167 0.594634
C 2.308075 -1.987525 1.832220
C 2.603483 1.580930 1.832220
C -0.699034 2.964594 1.832220
C -3.035510 0.251290 1.832220
C 1.177015 2.809289 -1.832220
C -3.035510 1.751167 -0.594634
C -2.308075 -1.987525 1.832220
C -2.603483 -2.345801 -0.594634
C 1.426469 -3.200952 -0.594634
C 3.485089 0.367504 -0.594634
C 0.727435 3.428082 -0.594634
C 2.603483 2.345801 0.594634
C 0.699034 -2.964594 -1.832220
C -1.426469 3.200952 0.594634
C -3.485089 -0.367504 0.594634
C -0.727435 -3.428082 0.594634
C 3.035510 -0.251290 -1.832220
C -2.603483 -1.580930 -1.832220
C 0.727435 -3.428082 0.594634
C 2.308075 0.749939 -2.597090
C -2.308075 -0.749939 2.597090
C 1.426469 1.963366 2.597090
C -1.426469 1.963366 2.597090
C -1.426469 -3.200952 -0.594634
C 1.426469 -1.963366 -2.597090
C 1.426469 3.200952 0.594634
C -2.603483 2.345801 0.594634
C -3.035510 -1.751167 0.594634
C 3.485089 -0.367504 0.594634
C 2.603483 -2.345801 -0.594634
C 3.035510 1.751167 -0.594634
C -0.727435 3.428082 -0.594634
C -3.485089 0.367504 -0.594634
C 2.603483 -1.580930 -1.832220
C -1.426469 -1.963366 -2.597090
C 2.308075 1.987525 -1.832220
C -2.308075 0.749939 -2.597090
C 3.035510 0.251290 1.832220
C 0.699034 2.964594 1.832220
C -2.603483 1.580930 1.832220
C -3.035510 -0.251290 -1.832220
C -0.699034 -2.964594 -1.832220

C60-
E = -2284.366687
C -0.275617 -3.355645 1.113567
C -0.130728 -1.614121 -3.153356
C 0.337989 -2.647800 -2.333034
C 1.686070 -2.575969 -1.776356
C 2.502985 -1.471188 -2.063324
C 2.018288 -0.403858 -2.915543
C 0.715901 -0.475768 -3.455694
C -0.534271 -3.254392 -1.345163
C 0.263802 -3.532385 -0.167432
C 1.639871 -3.121866 -0.436040
C -1.862638 -2.795531 -1.203861
C -2.420398 -2.607897 0.120982
C -1.650212 -2.883420 1.260848
C 0.523905 -2.773064 2.173144
C -0.345771 -1.919729 2.960526
C -1.693902 -1.996117 2.404364
C -3.265418 -1.421006 0.095796
C -3.307292 -0.563888 1.199788
C -2.506823 -0.852177 2.382235
C -2.018288 0.403858 2.915543
C -0.715901 0.475768 3.455694
C 0.126723 -0.704705 3.472125
C 1.502849 -0.292490 3.207334
C 2.342198 -1.114917 2.438951
C 1.853699 -2.373510 1.913721
C 2.411390 -2.551758 0.587699
C -3.219083 -0.872054 -1.250378
C -2.347187 -1.723530 -2.049181
C -1.505318 -1.141379 -3.010349
C -1.502849 0.292490 -3.207334
C -2.342198 1.114917 -2.438951
C -3.216600 0.513186 -1.440560
C -3.304705 0.877338 1.001865
C -3.260268 1.404767 -0.292292
C -2.502985 1.471188 2.063324
C -2.411390 2.551758 -0.587699
C -1.639871 3.121866 0.436040
C -1.686070 2.575969 1.776356
C 0.130728 1.614121 3.153356
C -0.337989 2.647800 2.333034
C 1.505318 1.141379 3.010349
C 0.534271 3.254392 1.345163
C 1.862638 2.795531 1.203861
C 2.347187 1.723530 2.049181
C 3.216600 -0.513186 1.440560
C 3.219083 0.872054 1.250378
C 3.260268 -1.404767 0.292292
C 3.304705 -0.877338 -1.001865
C 3.265418 1.421006 -0.095796
C 3.307292 0.563888 -1.199788
C 2.506823 0.852177 -2.382235
C 1.693902 1.996117 -2.404364
C 1.650212 2.883420 -1.260848
C 2.420398 2.607897 -0.120982
C 0.345771 1.919729 -2.960526
C -0.523905 2.773064 -2.173144
C 0.275617 3.355645 -1.113567
C -0.263802 3.532385 0.167432
C -1.853699 2.373510 -1.913721
C -0.126723 0.704705 -3.472125

C120-, B1
E = -4569.162804
C -2.569438 2.312089 4.298986
C -2.581850 1.571707 5.538936
C -3.012540 0.246105 5.538906
C -3.437827 -0.360165 4.298978
C -3.437827 0.360164 -4.298978
C -2.993004 1.729466 -4.298995
C -1.423574 1.959459 6.321970
C -2.303617 -0.748510 6.322093
C -2.993004 -1.729466 4.298995
C -3.012540 -0.246105 -5.538906
C -2.292746 1.969955 -5.539043
C -1.404856 3.158554 4.299098
C -2.581850 -1.571707 -5.538936
C -2.569438 -2.312089 -4.298986
C -1.423574 -1.959459 -6.321970
C -1.404856 -3.158554 -4.299098
C -0.727032 1.000643 7.071518
C -2.292746 -1.969955 5.539043
C -0.696918 -2.941408 -5.539058
C -1.165025 -2.789088 5.538945
C -1.176404 -0.382292 7.071570
C -2.303617 0.748510 -6.322093
C -1.165025 2.789088 -5.538945
C 0.727032 1.000643 7.071518
C -0.719827 3.380742 -4.298993
C -0.696918 2.941408 5.539058
C 0.000000 2.422091 -6.322032
C 0.719827 3.380742 -4.298993
C 0.696918 2.941408 5.539058
C -0.719827 -3.380742 4.298993
C -1.176404 0.382292 -7.071570
C 0.000000 -1.237050 7.071648
C 1.165025 2.789088 -5.538945
C 1.404856 3.158554 4.299098
C 1.423574 1.959459 6.321970
C 2.581850 1.571707 5.538936
C 1.176404 -0.382292 7.071570
C 0.000000 -2.422091 6.322032
C 1.165025 -2.789088 5.538945
C 2.569438 2.312089 4.298986
C 2.993004 1.729466 -4.298995
C 3.437827 0.360165 -4.298978
C 2.292746 1.969955 -5.539043
C -0.727032 -1.000643 -7.071518
C 2.292746 -1.969955 5.539043
C 0.000000 1.237050 -7.071648
C 1.176404 0.382292 -7.071570

C 2.303617 -0.748510 6.322093
C 0.719827 -3.380742 4.298993
C 1.404856 -3.158554 -4.299098
C 3.012540 0.246105 5.538906
C 3.437827 -0.360164 4.298978
C 2.303617 0.748510 -6.322093
C 3.012540 -0.246105 -5.538906
C 2.993004 -1.729466 4.298995
C 0.727032 -1.000643 -7.071518
C 0.696918 -2.941408 -5.539058
C 2.569438 -2.312089 -4.298986
C 1.423574 -1.959459 -6.321970
C 2.581850 -1.571707 -5.538936
C 1.429120 3.202723 -3.091059
C 0.715996 3.389168 -1.852903
C -0.715996 3.389168 -1.852903
C -1.429120 3.202723 -3.091059
C 2.604023 2.348841 -3.091048
C -2.604023 2.348841 -3.091048
C 0.726241 3.431573 3.091175
C -0.726241 3.431573 3.091175
C 3.038001 1.750702 3.091008
C 2.570640 2.320648 1.852881
C 1.412519 3.162790 1.852932
C -1.412519 3.162790 1.852932
C -2.570640 2.320648 1.852881
C -3.038001 1.750702 3.091008
C 3.487052 0.369480 3.091012
C 2.604023 -2.348841 3.091048
C 3.001286 -1.728133 1.852846
C 3.443772 -0.366246 1.852844
C 3.038001 -1.750702 -3.091008
C 3.487052 -0.369480 -3.091012
C 3.443772 0.366246 -1.852844
C 3.001286 1.728133 -1.852846
C 1.429120 -3.202723 3.091059
C 0.726241 -3.431573 -3.091175
C 1.412519 -3.162790 -1.852932
C 2.570640 -2.320648 -1.852881
C -0.726241 -3.431573 -3.091175
C -3.038001 -1.750702 -3.091008
C -2.570640 -2.320648 -1.852881
C -1.412519 -3.162790 -1.852932
C -2.604023 -2.348841 3.091048
C -1.429120 -3.202723 3.091059
C -3.487052 -0.369480 -3.091012
C -3.001286 1.728133 -1.852846
C -3.443772 0.366246 -1.852844
C -3.487052 0.369480 3.091012
C -0.715996 -3.389168 1.852903
C 0.715996 -3.389168 1.852903
C -1.402085 3.152545 -0.614883
C -2.564278 2.307612 -0.614884
C 2.564278 2.307612 -0.614884
C 1.402085 3.152545 -0.614883
C 2.986621 -1.725953 -0.614907
C 3.430732 -0.359414 -0.614895
C -0.718464 -3.374963 -0.614916
C 0.718464 -3.374963 -0.614916
C -3.430732 -0.359414 -0.614895
C -2.986621 -1.725953 -0.614907
C -3.443772 -0.366246 1.852844
C -3.001286 -1.728133 1.852846
C 0.718464 3.374963 0.614916
C -0.718464 3.374963 0.614916
C 3.430732 0.359414 0.614895
C 2.986621 1.725953 0.614907
C 1.402085 -3.152545 0.614883
C 2.564278 -2.307612 0.614884
C -2.564278 -2.307612 0.614884
C -1.402085 -3.152545 0.614883
C -2.986621 1.725953 0.614907
C -3.430732 0.359414 0.614895

C120-, C1
E = -4568.663550
C 1.429380 7.585024 1.176918
C 0.727827 7.146847 2.306393
C -0.727827 7.146847 2.306393
C -1.429380 7.585024 1.176918
C -0.699267 8.034027 0.001084
C 0.699267 8.034027 0.001084
C -1.175187 5.967899 3.030434
C -2.313263 5.274080 2.598000
C -3.043722 5.730508 1.428422
C -2.609891 6.866603 0.726584
C -2.609865 6.866931 -0.725135
C -3.043698 5.731219 -1.427679
C -3.490531 4.554890 -0.698610
C -3.490567 4.554573 0.698624
C -3.045976 3.374880 1.429826
C -2.596357 2.243982 0.725102
C -2.596375 2.244293 -0.726568
C -3.046013 3.375572 -1.430559
C -2.313275 5.275348 -2.597530
C -2.317434 3.818979 -2.592829
C -2.317477 3.817689 2.592405
C -1.174723 3.112879 3.004971
C -0.740610 1.933960 2.279234
C -1.451042 1.500168 1.175787
C -0.797717 0.791160 -0.001129
C -1.451121 1.500596 -1.177745
C -0.740612 1.935011 -2.280870
C -1.174733 3.114351 -3.005826
C -1.429359 7.585596 -1.175024
C -0.727821 7.147935 -2.304752
C -1.175184 5.969389 -3.029553
C 0.000000 5.241730 -3.476086
C 0.000000 3.836517 -3.455670
C 0.797717 0.791160 -0.001129
C 1.451042 1.500168 1.175787
C 0.740610 1.933960 2.279234
C 1.174723 3.112879 3.004971
C 0.000000 3.834837 3.455248
C 0.000000 5.240034 3.476555
C 1.175187 5.967899 3.030434
C 2.313263 5.274080 2.598000
C 2.317477 3.817689 2.592405
C 2.596357 2.243982 0.725102
C 3.045976 3.374880 1.429826
C 2.609891 6.866603 0.726584
C 3.043722 5.730508 1.428422
C 3.490567 4.554573 0.698624
C 1.451121 1.500596 -1.177745
C 0.740612 1.935011 -2.280870
C 1.174733 3.114351 -3.005826
C 1.429359 7.585596 -1.175024
C 0.727821 7.147935 -2.304752
C 1.175184 5.969389 -3.029553
C 2.313275 5.275348 -2.597530
C 2.317434 3.818979 -2.592829
C 2.596375 2.244293 -0.726568
C 3.046013 3.375572 -1.430559
C 3.490531 4.554890 -0.698610
C 3.043698 5.731219 -1.427679
C 2.609865 6.866931 -0.725135
C -1.451121 -1.500596 -1.177745
C -0.740612 -1.935011 -2.280870
C 0.740612 -1.935011 -2.280870
C 1.451121 -1.500596 -1.177745
C 0.797717 -0.791160 -0.001129
C -0.797717 -0.791160 -0.001129
C 2.596375 -2.244293 -0.726568
C 3.046013 -3.375572 -1.430559
C 2.317434 -3.818979 -2.592829
C 1.174733 -3.114351 -3.005826
C 3.490531 -4.554890 -0.698610
C 3.490567 -4.554573 0.698624
C 3.045976 -3.374880 1.429826
C 2.596357 -2.243982 0.725102
C 1.451042 -1.500168 1.175787
C 0.740610 -1.933960 2.279234
C 1.174723 -3.112879 3.004971
C 2.317477 -3.817689 2.592405
C -0.740610 -1.933960 2.279234
C -1.174723 -3.112879 3.004971
C -0.000000 -3.834837 3.455248
C -0.000000 -5.240034 3.476555
C -1.175187 -5.967899 3.030434
C -2.313263 -5.274080 2.598000
C -2.317477 -3.817689 2.592405
C -3.045976 -3.374880 1.429826
C -2.596357 -2.243982 0.725102
C -1.451042 -1.500168 1.175787
C 1.175187 -5.967899 3.030434
C 2.313263 -5.274080 2.598000
C 3.043722 -5.730508 1.428422
C 2.313275 -5.275348 -2.597530
C 3.043698 -5.731219 -1.427679
C 2.609865 -6.866931 -0.725135
C 2.609891 -6.866603 0.726584
C 1.175184 -5.969389 -3.029553
C -0.000000 -5.241730 -3.476086
C -0.000000 -3.836517 -3.455670
C -1.174733 -3.114351 -3.005826
C 0.727821 -7.147935 -2.304752
C 1.429359 -7.585596 -1.175024
C 0.727827 -7.146847 2.306393
C 1.429380 -7.585024 1.176918
C 0.699267 -8.034027 0.001084
C -0.699267 -8.034027 0.001084
C -1.429359 -7.585596 -1.175024
C -0.727821 -7.147935 -2.304752
C -1.175184 -5.969389 -3.029553
C -2.313275 -5.275348 -2.597530
C -2.317434 -3.818979 -2.592829
C -1.429380 -7.585024 1.176918
C -0.727827 -7.146847 2.306393
C -2.609891 -6.866603 0.726584
C -2.609865 -6.866931 -0.725135
C -3.043698 -5.731219 -1.427679
C -3.490531 -4.554890 -0.698610
C -3.046013 -3.375572 -1.430559
C -2.596375 -2.244293 -0.726568
C -3.490567 -4.554573 0.698624
C -3.043722 -5.730508 1.428422

C120-, P1
E = -4568.682625
C 7.405140 1.177294 1.428365
C 6.983000 2.314254 0.728780
C 6.983000 2.314254 -0.728780
C 7.405140 1.177294 -1.428365
C 7.853085 -0.000000 -0.699580
C 7.853085 -0.000000 0.699580
C 5.815145 3.055776 -1.176288
C 5.096555 2.611455 -2.298371
C 5.525174 1.425378 -3.009229
C 6.669942 0.725513 -2.598144
C 6.669942 -0.725513 -2.598144
C 5.525174 -1.425378 -3.009229
C 4.327760 -0.693053 -3.390002
C 4.327760 0.693053 -3.390002
C 3.160439 1.413731 -2.910034
C 2.002414 0.741104 -2.429557
C 2.002414 -0.741104 -2.429557
C 3.160439 -1.413731 -2.910034
C 5.096555 -2.611455 -2.298371
C 3.641189 -2.617994 -2.278575
C 3.641189 2.617994 -2.278575
C 2.965359 3.126169 -1.163505
C 1.742099 2.508967 -0.746031
C 1.216232 1.413155 -1.424482
C 0.000000 0.770734 -0.809461

C 1.216232 -1.413155 -1.424482
C 1.742099 -2.508967 -0.746031
C 2.965359 -3.126169 -1.163505
C 7.405140 -1.177294 -1.428365
C 6.983000 -2.314254 -0.728780
C 5.815145 -3.055776 -1.176288
C 5.107920 -3.531106 0.000000
C 3.707473 -3.566465 0.000000
C -0.000000 0.770734 0.809461
C 1.216232 1.413155 1.424482
C 1.742099 2.508967 0.746031
C 2.965359 3.126169 1.163505
C 3.707473 3.566465 0.000000
C 5.107920 3.531106 0.000000
C 5.815145 3.055776 1.176288
C 5.096555 2.611455 2.298371
C 3.641189 2.617994 2.278575
C 2.002414 0.741104 2.429557
C 3.160439 1.413731 2.910034
C 6.669942 0.725513 2.598144
C 5.525174 1.425378 3.009229
C 4.327760 0.693053 3.390002
C 1.216232 -1.413155 1.424482
C 1.742099 -2.508967 0.746031
C 2.965359 -3.126169 1.163505
C 7.405140 -1.177294 1.428365
C 6.983000 -2.314254 0.728780
C 5.815145 -3.055776 1.176288
C 5.096555 -2.611455 2.298371
C 3.641189 -2.617994 2.278575
C 2.002414 -0.741104 2.429557
C 3.160439 -1.413731 2.910034
C 4.327760 -0.693053 3.390002
C 5.525174 -1.425378 3.009229
C 6.669942 -0.725513 2.598144
C -1.216232 -1.413155 -1.424482
C -1.742099 -2.508967 -0.746031
C -1.742099 -2.508967 0.746031
C -1.216232 -1.413155 1.424482
C -0.000000 -0.770734 0.809461
C -0.000000 -0.770734 -0.809461
C -2.002414 -0.741104 2.429557
C -3.160439 -1.413731 2.910034
C -3.641189 -2.617994 2.278575
C -2.965359 -3.126169 1.163505
C -4.327760 -0.693053 3.390002
C -4.327760 0.693053 3.390002
C -3.160439 1.413731 2.910034
C -2.002414 0.741104 2.429557
C -1.216232 1.413155 1.424482
C -1.742099 2.508967 0.746031
C -2.965359 3.126169 1.163505
C -3.641189 2.617994 2.278575
C -1.742099 2.508967 -0.746031
C -2.965359 3.126169 -1.163505
C -3.707473 3.566465 0.000000
C -5.107920 3.531106 0.000000
C -5.815145 3.055776 -1.176288
C -5.096555 2.611455 -2.298371
C -3.641189 2.617994 -2.278575
C -3.160439 1.413731 -2.910034
C -2.002414 0.741104 -2.429557
C -1.216232 1.413155 -1.424482
C -5.815145 3.055776 1.176288
C -5.096555 2.611455 2.298371
C -5.525174 1.425378 3.009229
C -5.096555 -2.611455 2.298371
C -5.525174 -1.425378 3.009229
C -6.669942 -0.725513 2.598144
C -6.669942 0.725513 2.598144
C -5.815145 -3.055776 1.176288
C -5.107920 -3.531106 -0.000000
C -3.707473 -3.566465 -0.000000
C -2.965359 -3.126169 -1.163505
C -6.983000 -2.314254 0.728780
C -7.405140 -1.177294 1.428365
C -6.983000 2.314254 0.728780
C -7.405140 1.177294 1.428365
C -7.853085 -0.000000 0.699580
C -7.853085 0.000000 -0.699580
C -7.405140 -1.177294 -1.428365
C -6.983000 -2.314254 -0.728780
C -5.815145 -3.055776 -1.176288
C -5.096555 -2.611455 -2.298371
C -3.641189 -2.617994 -2.278575
C -7.405140 1.177294 -1.428365
C -6.983000 2.314254 -0.728780
C -6.669942 0.725513 -2.598144
C -6.669942 -0.725513 -2.598144
C -5.525174 -1.425378 -3.009229
C -4.327760 -0.693053 -3.390002
C -3.160439 -1.413731 -2.910034
C -2.002414 -0.741104 -2.429557
C -4.327760 0.693053 -3.390002
C -5.525174 1.425378 -3.009229

C120-, V1
E = -4568.661958
C 0.000000 2.393134 2.459738
C -0.000000 -2.393134 2.459738
C 0.000000 7.511277 -2.458718
C -0.000000 -7.511277 -2.458718
C 1.179030 -8.279625 -0.426064
C 2.314469 -7.539934 -0.787272
C 2.315335 -6.760428 -2.012087
C 1.179417 -6.745776 -2.835618
C 0.000000 -8.262920 -1.279425
C 3.040719 -5.525943 -1.759464
C 2.606945 -4.326396 -2.335280
C 1.426132 -4.311964 -3.189969
C 0.729089 -5.501914 -3.432584
C -0.729089 -5.501914 -3.432584
C -1.426132 -4.311964 -3.189969
C -0.703604 -3.076803 -2.944676
C 0.703604 -3.076803 -2.944676
C 1.427200 -2.332129 -1.931415
C 0.728702 -1.615875 -0.953267
C -0.728702 -1.615875 -0.953267
C -1.427200 -2.332129 -1.931415
C -2.606945 -4.326396 -2.335280
C -2.608411 -3.100533 -1.556099
C 2.608411 -3.100533 -1.556099
C 3.040434 -3.116296 -0.225637
C 2.317808 -2.363772 0.788605
C 1.179411 -1.628257 0.425955
C 0.000000 -1.647518 1.278116
C -1.179411 -1.628257 0.425955
C -2.317808 -2.363772 0.788605
C -3.040434 -3.116296 -0.225637
C -1.179417 -6.745776 -2.835618
C -2.315335 -6.760428 -2.012087
C -3.040719 -5.525943 -1.759464
C -3.491214 -5.543055 -0.375933
C -3.490136 -4.363093 0.374801
C 1.180190 -3.157395 2.837128
C 2.315679 -3.143197 2.011572
C 3.038632 -4.379893 1.758582
C 3.490136 -4.363093 0.374801
C 3.491214 -5.543055 -0.375933
C 3.039929 -6.788850 0.225592
C 2.606846 -6.804492 1.555742
C 2.606285 -5.578313 2.336232
C 0.729484 -4.401426 3.431637
C 1.426910 -5.592159 3.191655
C 0.728850 -8.294740 0.954246
C 1.426387 -7.572403 1.929724
C 0.702909 -6.825715 2.944042
C -1.180190 -3.157395 2.837128
C -2.315679 -3.143197 2.011572
C -3.038632 -4.379893 1.758582
C -1.179030 -8.279625 -0.426064
C -2.314469 -7.539934 -0.787272
C -3.039929 -6.788850 0.225592
C -2.606846 -6.804492 1.555742
C -2.606285 -5.578313 2.336232
C -0.729484 -4.401426 3.431637
C -1.426910 -5.592159 3.191655
C -0.702909 -6.825715 2.944042
C -1.426387 -7.572403 1.929724
C -0.728850 -8.294740 0.954246
C -1.179411 1.628257 0.425955
C -2.317808 2.363772 0.788605
C -2.315679 3.143197 2.011572
C -1.180190 3.157395 2.837128
C 0.000000 1.647518 1.278116
C -0.729484 4.401426 3.431637
C -1.426910 5.592159 3.191655
C -2.606285 5.578313 2.336232
C -3.038632 4.379893 1.758582
C -0.702909 6.825715 2.944042
C 0.702909 6.825715 2.944042
C 1.426910 5.592159 3.191655
C 0.729484 4.401426 3.431637
C 1.180190 3.157395 2.837128
C 2.315679 3.143197 2.011572
C 3.038632 4.379893 1.758582
C 2.606285 5.578313 2.336232
C 2.317808 2.363772 0.788605
C 3.040434 3.116296 -0.225637
C 3.490136 4.363093 0.374801
C 3.491214 5.543055 -0.375933
C 3.040719 5.525943 -1.759464
C 2.606945 4.326396 -2.335280
C 2.608411 3.100533 -1.556099
C 1.427200 2.332129 -1.931415
C 0.728702 1.615875 -0.953267
C 1.179411 1.628257 0.425955
C 3.039929 6.788850 0.225592
C 2.606846 6.804492 1.555742
C 1.426387 7.572403 1.929724
C -2.606846 6.804492 1.555742
C -1.426387 7.572403 1.929724
C -0.728850 8.294740 0.954246
C 0.728850 8.294740 0.954246
C -3.039929 6.788850 0.225592
C -3.491214 5.543055 -0.375933
C -3.490136 4.363093 0.374801
C -3.040434 3.116296 -0.225637
C -2.314469 7.539934 -0.787272
C -1.179030 8.279625 -0.426064
C 2.314469 7.539934 -0.787272
C 1.179030 8.279625 -0.426064
C 0.000000 8.262920 -1.279425
C -1.179417 6.745776 -2.835618
C -2.315335 6.760428 -2.012087
C -3.040719 5.525943 -1.759464
C -2.606945 4.326396 -2.335280
C -2.608411 3.100533 -1.556099
C 1.179417 6.745776 -2.835618
C 2.315335 6.760428 -2.012087
C 0.729089 5.501914 -3.432584
C -0.729089 5.501914 -3.432584
C -1.426132 4.311964 -3.189969
C -0.703604 3.076803 -2.944676
C -1.427200 2.332129 -1.931415
C -0.728702 1.615875 -0.953267
C 0.703604 3.076803 -2.944676
C 1.426132 4.311964 -3.189969

Benchmarking calculations: BHandHLYP/def2-SVP

C60
E = -2282.805220
C 0.000000 -1.230128 3.306745

C -0.000000 -2.407512 2.579081
C 1.169922 -2.787643 1.818820
C 2.289680 -0.743962 2.579081
C 1.169922 -0.380131 3.306745
C 0.723051 0.995195 3.306745
C -0.723051 0.995195 3.306745
C -1.169922 -0.380131 3.306745
C -0.000000 1.230128 -3.306745
C 0.000000 2.407512 -2.579081
C -1.169922 2.787643 -1.818820
C -1.169922 0.380131 -3.306745
C -2.289680 1.974091 -1.818820
C -0.723051 -0.995195 -3.306745
C 0.723051 -0.995195 -3.306745
C 1.169922 0.380131 -3.306745
C -1.169922 -2.787643 1.818820
C 3.012732 -1.739157 0.588692
C 2.289680 -1.974091 1.818820
C 2.585022 1.567588 1.818820
C -0.692049 2.942913 1.818820
C -3.012732 0.251232 1.818820
C 1.169922 2.787643 -1.818820
C -3.012732 1.739157 -0.588692
C -2.289680 -1.974091 1.818820
C -2.585022 -2.327849 -0.588692
C 1.415100 -3.177847 -0.588692
C 3.459602 0.363832 -0.588692
C 0.723051 3.402707 -0.588692
C 2.585022 2.327849 0.588692
C 0.692049 -2.942913 -1.818820
C -1.415100 3.177847 0.588692
C -3.459602 -0.363832 0.588692
C -0.723051 -3.402707 0.588692
C 3.012732 -0.251232 -1.818820
C -2.585022 -1.567588 -1.818820
C 0.723051 -3.402707 0.588692
C 2.289680 0.743962 -2.579081
C -2.289680 -0.743962 2.579081
C 1.415100 1.947718 2.579081
C -1.415100 1.947718 2.579081
C -1.415100 -3.177847 -0.588692
C 1.415100 -1.947718 -2.579081
C 1.415100 3.177847 0.588692
C -2.585022 2.327849 0.588692
C -3.012732 -1.739157 0.588692
C 3.459602 -0.363832 0.588692
C 2.585022 -2.327849 -0.588692
C 3.012732 1.739157 -0.588692
C -0.723051 3.402707 -0.588692
C -3.459602 0.363832 -0.588692
C 2.585022 -1.567588 -1.818820
C -1.415100 -1.947718 -2.579081
C 2.289680 1.974091 -1.818820
C -2.289680 0.743962 -2.579081
C 3.012732 0.251232 1.818820
C 0.692049 2.942913 1.818820
C -2.585022 1.567588 1.818820
C -3.012732 -0.251232 -1.818820
C -0.692049 -2.942913 -1.818820

C60-
E = -2282.891847
C -0.273624 -3.341959 1.110597
C -0.127940 -1.605939 -3.132003
C 0.341284 -2.626528 -2.321609
C 1.676397 -2.555974 -1.772409
C 2.488884 -1.465269 -2.052366
C 1.999741 -0.401303 -2.897314
C 0.718207 -0.471916 -3.427296
C -0.526074 -3.229761 -1.330155
C 0.261954 -3.518449 -0.168272
C 1.622414 -3.095996 -0.432795
C -1.838729 -2.768441 -1.196920
C -2.394136 -2.580365 0.127985
C -1.632599 -2.859419 1.250817
C 0.515886 -2.753310 2.151979
C -0.348961 -1.902234 2.943712
C -1.684151 -1.977987 2.395199
C -3.239226 -1.408019 0.102350
C -3.286722 -0.558524 1.199647
C -2.492749 -0.849462 2.370220
C -1.999741 0.401303 2.897314
C -0.718207 0.471916 3.427296
C 0.124004 -0.702548 3.449218
C 1.488000 -0.287662 3.190719
C 2.319383 -1.107751 2.422794
C 1.829969 -2.349231 1.899791
C 2.385362 -2.527108 0.573541
C -3.183475 -0.863368 -1.235401
C -2.324232 -1.712280 -2.035493
C -1.490416 -1.132310 -2.995678
C -1.488000 0.287662 -3.190719
C -2.319383 1.107751 -2.422794
C -3.181101 0.508823 -1.423822
C -3.284234 0.872121 1.003163
C -3.234328 1.394067 -0.282520
C -2.488884 1.465269 2.052366
C -2.385362 2.527108 -0.573541
C -1.622414 3.095996 0.432795
C -1.676397 2.555974 1.772409
C 0.127940 1.605939 3.132003
C -0.341284 2.626528 2.321609
C 1.490416 1.132310 2.995678
C 0.526074 3.229761 1.330155
C 1.838729 2.768441 1.196920
C 2.324232 1.712280 2.035493
C 3.181101 -0.508823 1.423822
C 3.183475 0.863368 1.235401
C 3.234328 -1.394067 0.282520
C 3.284234 -0.872121 -1.003163
C 3.239226 1.408019 -0.102350
C 3.286722 0.558524 -1.199647
C 2.492749 0.849462 -2.370220
C 1.684151 1.977987 -2.395199
C 1.632599 2.859419 -1.250817
C 2.394136 2.580365 -0.127985
C 0.348961 1.902234 -2.943712
C -0.515886 2.753310 -2.151979
C 0.273624 3.341959 -1.110597
C -0.261954 3.518449 0.168272
C -1.829969 2.349231 -1.899791
C -0.124004 0.702548 -3.449218

C120-, B1
E = -4566.235059
C -2.551234 2.295606 4.268110
C -2.561680 1.557737 5.499506
C -2.988030 0.245523 5.499489
C -3.413366 -0.357566 4.268105
C -3.413366 0.357566 -4.268105
C -2.971683 1.717138 -4.268118
C -1.411523 1.942841 6.278074
C -2.284029 -0.742135 6.278149
C -2.971683 -1.717138 4.268118
C -2.988030 -0.245523 -5.499489
C -2.273180 1.955024 -5.499570
C -1.394837 3.135966 4.268170
C -2.561680 -1.557737 -5.499506
C -2.551234 -2.295606 -4.268110
C -1.411523 -1.942841 -6.278074
C -1.394837 -3.135966 -4.268170
C -0.722503 0.994419 7.021500
C -2.273180 -1.955024 5.499570
C -0.689877 -2.917804 -5.499573
C -1.156875 -2.765913 5.499495
C -1.169063 -0.379884 7.021540
C -2.284029 0.742135 -6.278149
C -1.156875 2.765913 -5.499495
C 0.722503 0.994419 7.021500
C -0.714733 3.356707 -4.268103
C -0.689877 2.917804 5.499573
C 0.000000 2.401503 -6.278095
C 0.714733 3.356707 -4.268103
C 0.689877 2.917804 5.499573
C -0.714733 -3.356707 4.268103
C -1.169063 0.379884 -7.021540
C 0.000000 -1.229288 7.021598
C 1.156875 2.765913 -5.499495
C 1.394837 3.135966 4.268170
C 1.411523 1.942841 6.278074
C 2.561680 1.557737 5.499506
C 1.169063 -0.379884 7.021540
C 0.000000 -2.401503 6.278095
C 1.156875 -2.765913 5.499495
C 2.551234 2.295606 4.268110
C 2.971683 1.717138 -4.268118
C 3.413366 0.357566 -4.268105
C 2.273180 1.955024 -5.499570
C -0.722503 -0.994419 -7.021500
C 2.273180 -1.955024 5.499570
C 0.000000 1.229288 -7.021598
C 1.169063 0.379884 -7.021540
C 2.284029 -0.742135 6.278149
C 0.714733 -3.356707 4.268103
C 1.394837 -3.135966 -4.268170
C 2.988030 0.245523 5.499489
C 3.413366 -0.357566 4.268105
C 2.284029 0.742135 -6.278149
C 2.988030 -0.245523 -5.499489
C 2.971683 -1.717138 4.268118
C 0.722503 -0.994419 -7.021500
C 0.689877 -2.917804 -5.499573
C 2.551234 -2.295606 -4.268110
C 1.411523 -1.942841 -6.278074
C 2.561680 -1.557737 -5.499506
C 1.419197 3.182163 -3.071964
C 0.709414 3.365579 -1.840636
C -0.709414 3.365579 -1.840636
C -1.419197 3.182163 -3.071964
C 2.587696 2.333065 -3.071963
C -2.587696 2.333065 -3.071963
C 0.722229 3.408877 3.072033
C -0.722229 3.408877 3.072033
C 3.018179 1.740046 3.071939
C 2.551781 2.305623 1.840626
C 1.404135 3.139821 1.840654
C -1.404135 3.139821 1.840654
C -2.551781 2.305623 1.840626
C -3.018179 1.740046 3.071939
C 3.464666 0.366384 3.071942
C 2.587696 -2.333065 3.071963
C 2.981241 -1.714652 1.840607
C 3.419661 -0.365273 1.840605
C 3.018179 -1.740046 -3.071939
C 3.464666 -0.366384 -3.071942
C 3.419661 0.365273 -1.840605
C 2.981241 1.714652 -1.840607
C 1.419197 -3.182163 3.071964
C 0.722229 -3.408877 -3.072033
C 1.404135 -3.139821 -1.840654
C 2.551781 -2.305623 -1.840626
C -0.722229 -3.408877 -3.072033
C -3.018179 -1.740046 -3.071939
C -2.551781 -2.305623 -1.840626
C -1.404135 -3.139821 -1.840654
C -2.587696 -2.333065 3.071963
C -1.419197 -3.182163 3.071964
C -3.464666 -0.366384 -3.071942
C -2.981241 1.714652 -1.840607
C -3.419661 0.365273 -1.840605
C -3.464666 0.366384 3.071942
C -0.709414 -3.365579 1.840636
C 0.709414 -3.365579 1.840636
C -1.393883 3.133695 -0.609049
C -2.549210 2.294009 -0.609051

C 2.549210 2.294009 -0.609051
C 1.393883 3.133695 -0.609049
C 2.969220 -1.715697 -0.609064
C 3.410618 -0.357370 -0.609058
C -0.714132 -3.354717 -0.609069
C 0.714132 -3.354717 -0.609069
C -3.410618 -0.357370 -0.609058
C -2.969220 -1.715697 -0.609064
C -3.419661 -0.365273 1.840605
C -2.981241 -1.714652 1.840607
C 0.714132 3.354717 0.609069
C -0.714132 3.354717 0.609069
C 3.410618 0.357370 0.609058
C 2.969220 1.715697 0.609064
C 1.393883 -3.133695 0.609049
C 2.549210 -2.294009 0.609051
C -2.549210 -2.294009 0.609051
C -1.393883 -3.133695 0.609049
C -2.969220 1.715697 0.609064
C -3.410618 0.357370 0.609058

C120-, C1
E = -4565.706677
6 -1.418019 7.547239 -1.168726
6 -0.723304 7.113556 -2.286875
6 0.723304 7.113556 -2.286875
6 1.418019 7.547239 -1.168726
6 0.692133 7.993992 0.000035
6 -0.692133 7.993992 0.000035
6 1.167578 5.941135 -3.006138
6 2.294848 5.254541 -2.578010
6 3.020532 5.707812 -1.416197
6 2.591163 6.833329 -0.720932
6 2.591158 6.833310 0.720973
6 3.020528 5.707787 1.416220
6 3.464208 4.538986 0.691678
6 3.464212 4.539003 -0.691679
6 3.022938 3.365998 -1.418636
6 2.579136 2.244424 -0.721185
6 2.579134 2.244408 0.721142
6 3.022946 3.365973 1.418616
6 2.294848 5.254498 2.578028
6 2.299004 3.806764 2.571814
6 2.299007 3.806807 -2.571834
6 1.166849 3.109183 -2.979645
6 0.737322 1.937057 -2.260061
6 1.440757 1.503432 -1.168412
6 0.792850 0.786892 -0.000036
6 1.440758 1.503417 1.168348
6 0.737317 1.937020 2.260009
6 1.166848 3.109132 2.979614
6 1.418015 7.547219 1.168786
6 0.723304 7.113514 2.286926
6 1.167581 5.941085 3.006173
6 0.000000 5.218281 3.450082
6 0.000000 3.826178 3.427153
6 -0.792850 0.786892 -0.000036
6 -1.440757 1.503432 -1.168412
6 -0.737322 1.937057 -2.260061
6 -1.166849 3.109183 -2.979645
6 0.000000 3.826233 -3.427166
6 0.000000 5.218340 -3.450061
6 -1.167578 5.941135 -3.006138
6 -2.294848 5.254541 -2.578010
6 -2.299007 3.806807 -2.571834
6 -2.579136 2.244424 -0.721185
6 -3.022938 3.365998 -1.418636
6 -2.591163 6.833329 -0.720932
6 -3.020532 5.707812 -1.416197
6 -3.464212 4.539003 -0.691679
6 -1.440758 1.503417 1.168348
6 -0.737317 1.937020 2.260009
6 -1.166848 3.109132 2.979614
6 -1.418015 7.547219 1.168786
6 -0.723304 7.113514 2.286926
6 -1.167581 5.941085 3.006173
6 -2.294848 5.254498 2.578028
6 -2.299004 3.806764 2.571814
6 -2.579134 2.244408 0.721142
6 -3.022946 3.365973 1.418616
6 -3.464208 4.538986 0.691678
6 -3.020528 5.707787 1.416220
6 -2.591158 6.833310 0.720973
6 1.440758 -1.503417 1.168348
6 0.737317 -1.937020 2.260009
6 -0.737317 -1.937020 2.260009
6 -1.440758 -1.503417 1.168348
6 -0.792850 -0.786892 -0.000036
6 0.792850 -0.786892 -0.000036
6 -2.579134 -2.244408 0.721142
6 -3.022946 -3.365973 1.418616
6 -2.299004 -3.806764 2.571814
6 -1.166848 -3.109132 2.979614
6 -3.464208 -4.538986 0.691678
6 -3.464212 -4.539003 -0.691679
6 -3.022938 -3.365998 -1.418636
6 -2.579136 -2.244424 -0.721185
6 -1.440757 -1.503432 -1.168412
6 -0.737322 -1.937057 -2.260061
6 -1.166849 -3.109183 -2.979645
6 -2.299007 -3.806807 -2.571834
6 0.737322 -1.937057 -2.260061
6 1.166849 -3.109183 -2.979645
6 -0.000000 -3.826233 -3.427166
6 -0.000000 -5.218340 -3.450061
6 1.167578 -5.941135 -3.006138
6 2.294848 -5.254541 -2.578010
6 2.299007 -3.806807 -2.571834
6 3.022938 -3.365998 -1.418636
6 2.579136 -2.244424 -0.721185
6 1.440757 -1.503432 -1.168412
6 -1.167578 -5.941135 -3.006138
6 -2.294848 -5.254541 -2.578010
6 -3.020532 -5.707812 -1.416197
6 -2.294848 -5.254498 2.578028
6 -3.020528 -5.707787 1.416220
6 -2.591158 -6.833310 0.720973
6 -2.591163 -6.833329 -0.720932
6 -1.167581 -5.941085 3.006173
6 -0.000000 -5.218281 3.450082
6 -0.000000 -3.826178 3.427153
6 1.166848 -3.109132 2.979614
6 -0.723304 -7.113514 2.286926
6 -1.418015 -7.547219 1.168786
6 -0.723304 -7.113556 -2.286875
6 -1.418019 -7.547239 -1.168726
6 -0.692133 -7.993992 0.000035
6 0.692133 -7.993992 0.000035
6 1.418015 -7.547219 1.168786
6 0.723304 -7.113514 2.286926
6 1.167581 -5.941085 3.006173
6 2.294848 -5.254498 2.578028
6 2.299004 -3.806764 2.571814
6 1.418019 -7.547239 -1.168726
6 0.723304 -7.113556 -2.286875
6 2.591163 -6.833329 -0.720932
6 2.591158 -6.833310 0.720973
6 3.020528 -5.707787 1.416220
6 3.464208 -4.538986 0.691678
6 3.022946 -3.365973 1.418616
6 2.579134 -2.244408 0.721142
6 3.464212 -4.539003 -0.691679
6 3.020532 -5.707812 -1.416197

C120-, P1
E = -4565.720628
6 1.416792 1.170537 -7.359881
6 0.724888 2.295964 -6.940728
6 -0.724888 2.295964 -6.940728
6 -1.416792 1.170537 -7.359881
6 -0.692523 0.000000 -7.805010
6 0.692523 -0.000000 -7.805010
6 -1.171156 3.030778 -5.781075
6 -2.280617 2.592112 -5.069238
6 -2.986483 1.412035 -5.495607
6 -2.578463 0.721905 -6.627547
6 -2.578463 -0.721905 -6.627547
6 -2.986483 -1.412035 -5.495607
6 -3.369899 -0.685996 -4.306275
6 -3.369899 0.685996 -4.306275
6 -2.891589 1.402716 -3.145732
6 -2.417806 0.738672 -1.998963
6 -2.417806 -0.738672 -1.998963
6 -2.891589 -1.402716 -3.145732
6 -2.280617 -2.592112 -5.069238
6 -2.264125 -2.599273 -3.623840
6 -2.264125 2.599273 -3.623840
6 -1.157881 3.096118 -2.954526
6 -0.743653 2.485549 -1.740818
6 -1.415053 1.403424 -1.214454
6 -0.802337 0.766674 -0.000457
6 -1.415053 -1.403424 -1.214454
6 -0.743653 -2.485549 -1.740818
6 -1.157881 -3.096118 -2.954526
6 -1.416792 -1.170537 -7.359881
6 -0.724888 -2.295964 -6.940728
6 -1.171156 -3.030778 -5.781075
6 0.000000 -3.500595 -5.075881
6 0.000000 -3.530622 -3.693827
6 0.802337 0.766674 -0.000457
6 1.415053 1.403424 -1.214454
6 0.743653 2.485549 -1.740818
6 1.157881 3.096118 -2.954526
6 0.000000 3.530622 -3.693827
6 0.000000 3.500595 -5.075881
6 1.171156 3.030778 -5.781075
6 2.280617 2.592112 -5.069238
6 2.264125 2.599273 -3.623840
6 2.417806 0.738672 -1.998963
6 2.891589 1.402716 -3.145732
6 2.578463 0.721905 -6.627547
6 2.986483 1.412035 -5.495607
6 3.369899 0.685996 -4.306275
6 1.415053 -1.403424 -1.214454
6 0.743653 -2.485549 -1.740818
6 1.157881 -3.096118 -2.954526
6 1.416792 -1.170537 -7.359881
6 0.724888 -2.295964 -6.940728
6 1.171156 -3.030778 -5.781075
6 2.280617 -2.592112 -5.069238
6 2.264125 -2.599273 -3.623840
6 2.417806 -0.738672 -1.998963
6 2.891589 -1.402716 -3.145732
6 3.369899 -0.685996 -4.306275
6 2.986483 -1.412035 -5.495607
6 2.578463 -0.721905 -6.627547
6 -1.418208 -1.404766 1.212993
6 -0.742305 -2.487032 1.735760
6 0.742305 -2.487032 1.735760
6 1.418208 -1.404766 1.212993
6 0.802337 -0.766674 -0.000457
6 -0.802337 -0.766674 -0.000457
6 2.421402 -0.736508 1.990555
6 2.890799 -1.403620 3.143317
6 2.259285 -2.593435 3.622176
6 1.152887 -3.098255 2.954432
6 3.362905 -0.686297 4.307238
6 3.362905 0.686297 4.307238
6 2.890799 1.403620 3.143317
6 2.421402 0.736508 1.990555
6 1.418208 1.404766 1.212993
6 0.742305 2.487032 1.735760
6 1.152887 3.098255 2.954432
6 2.259285 2.593435 3.622176
6 -0.742305 2.487032 1.735760

6 -1.152887 3.098255 2.954432
6 0.000000 3.540818 3.688062
6 0.000000 3.504947 5.080552
6 -1.166218 3.030084 5.780429
6 -2.279049 2.587979 5.070800
6 -2.259285 2.593435 3.622176
6 -2.890799 1.403620 3.143317
6 -2.421402 0.736508 1.990555
6 -1.418208 1.404766 1.212993
6 1.166218 3.030084 5.780429
6 2.279049 2.587979 5.070800
6 2.987019 1.414267 5.497284
6 2.279049 -2.587979 5.070800
6 2.987019 -1.414267 5.497284
6 2.580389 -0.719099 6.634253
6 2.580389 0.719099 6.634253
6 1.166218 -3.030084 5.780429
6 -0.000000 -3.504947 5.080552
6 -0.000000 -3.540818 3.688062
6 -1.152887 -3.098255 2.954432
6 0.723589 -2.294250 6.944834
6 1.417602 -1.169519 7.363046
6 0.723589 2.294250 6.944834
6 1.417602 1.169519 7.363046
6 0.692303 -0.000000 7.810155
6 -0.692303 0.000000 7.810155
6 -1.417602 -1.169519 7.363046
6 -0.723589 -2.294250 6.944834
6 -1.166218 -3.030084 5.780429
6 -2.279049 -2.587979 5.070800
6 -2.259285 -2.593435 3.622176
6 -1.417602 1.169519 7.363046
6 -0.723589 2.294250 6.944834
6 -2.580389 0.719099 6.634253
6 -2.580389 -0.719099 6.634253
6 -2.987019 -1.414267 5.497284
6 -3.362905 -0.686297 4.307238
6 -2.890799 -1.403620 3.143317
6 -2.421402 -0.736508 1.990555
6 -3.362905 0.686297 4.307238
6 -2.987019 1.414267 5.497284

C120-, V1
E = -4565.705556
6 1.398962 -1.910271 2.512820
6 1.398962 -1.910271 -2.512820
6 -1.398912 1.909441 7.740730
6 -1.398912 1.909441 -7.740730
6 0.739860 0.933890 -8.444806
6 1.431124 1.900010 -7.730330
6 0.712873 2.913056 -6.994323
6 -0.679460 2.917485 -6.999637
6 -0.708875 0.938541 -8.450272
6 1.428441 3.161501 -5.767250
6 0.734109 3.405047 -4.591210
6 -0.715311 3.409462 -4.596671
6 -1.402443 3.170297 -5.777402
6 -2.578096 2.324619 -5.764819
6 -3.011928 1.758160 -4.577948
6 -2.296871 2.010694 -3.346986
6 -1.169628 2.824401 -3.359643
6 -0.001500 2.447914 -2.594763
6 -0.007946 1.281658 -1.848262
6 -1.183404 0.439032 -1.830052
6 -2.305519 0.799107 -2.568095
6 -3.465541 0.384600 -4.558168
6 -3.024110 -0.208801 -3.314956
6 1.175000 2.816849 -3.351247
6 2.296337 1.995245 -3.330016
6 2.291695 0.784056 -2.550896
6 1.161569 0.431275 -1.820666
6 0.709025 -0.937442 -1.804677
6 -0.740008 -0.932817 -1.810283
6 -1.431196 -1.900826 -2.523352
6 -2.600885 -1.526980 -3.292729
6 -2.576311 1.544318 -6.980732
6 -3.009444 0.228898 -6.962178
6 -3.464855 -0.362733 -5.723224
6 -3.021338 -1.739373 -5.699576
6 -2.598072 -2.306252 -4.508446
6 2.576774 -1.544077 -3.273851
6 3.008610 -0.228554 -3.292813
6 3.463462 0.361653 -4.532761
6 3.018993 1.738078 -4.555958
6 2.597880 2.307532 -5.745759
6 2.600430 1.527226 -6.961886
6 3.024819 0.209097 -6.940044
6 3.466879 -0.385733 -5.697845
6 2.578271 -2.323288 -4.489331
6 3.014328 -1.759398 -5.677616
6 1.182747 -0.439339 -8.423140
6 2.302833 -0.797940 -7.684999
6 2.297172 -2.012330 -6.905935
6 0.680252 -2.916821 -3.251588
6 -0.713738 -2.912599 -3.256856
6 -1.427486 -3.158927 -4.486148
6 -1.160859 -0.431568 -8.432061
6 -2.288943 -0.782877 -7.701928
6 -2.296786 -1.997013 -6.922933
6 -1.174726 -2.816214 -6.900865
6 -0.734037 -3.406629 -5.661244
6 1.401623 -3.167715 -4.475959
6 0.715308 -3.410971 -5.655793
6 1.169427 -2.823918 -6.892500
6 0.001484 -2.448383 -7.658838
6 0.007942 -1.283353 -8.407643
6 -0.740008 -0.932817 1.810283
6 -1.431196 -1.900826 2.523352
6 -0.713738 -2.912599 3.256856
6 0.680252 -2.916821 3.251588
6 0.709025 -0.937442 1.804677
6 1.401623 -3.167715 4.475959
6 0.715308 -3.410971 5.655793
6 -0.734037 -3.406629 5.661244
6 -1.427486 -3.158927 4.486148
6 1.169427 -2.823918 6.892500
6 2.297172 -2.012330 6.905935
6 3.014328 -1.759398 5.677616
6 2.578271 -2.323288 4.489331
6 2.576774 -1.544077 3.273851
6 3.008610 -0.228554 3.292813
6 3.463462 0.361653 4.532761
6 3.466879 -0.385733 5.697845
6 2.291695 0.784056 2.550896
6 2.296337 1.995245 3.330016
6 3.018993 1.738078 4.555958
6 2.597880 2.307532 5.745759
6 1.428441 3.161501 5.767250
6 0.734109 3.405047 4.591210
6 1.175000 2.816849 3.351247
6 -0.001500 2.447914 2.594763
6 -0.007946 1.281658 1.848262
6 1.161569 0.431275 1.820666
6 2.600430 1.527226 6.961886
6 3.024819 0.209097 6.940044
6 2.302833 -0.797940 7.684999
6 -1.174726 -2.816214 6.900865
6 0.001484 -2.448383 7.658838
6 0.007942 -1.283353 8.407643
6 1.182747 -0.439339 8.423140
6 -2.296786 -1.997013 6.922933
6 -3.021338 -1.739373 5.699576
6 -2.598072 -2.306252 4.508446
6 -2.600885 -1.526980 3.292729
6 -2.288943 -0.782877 7.701928
6 -1.160859 -0.431568 8.432061
6 1.431124 1.900010 7.730330
6 0.739860 0.933890 8.444806
6 -0.708875 0.938541 8.450272
6 -2.576311 1.544318 6.980732
6 -3.009444 0.228898 6.962178
6 -3.464855 -0.362733 5.723224
6 -3.465541 0.384600 4.558168
6 -3.024110 -0.208801 3.314956
6 -0.679460 2.917485 6.999637
6 0.712873 2.913056 6.994323
6 -1.402443 3.170297 5.777402
6 -2.578096 2.324619 5.764819
6 -3.011928 1.758160 4.577948
6 -2.296871 2.010694 3.346986
6 -2.305519 0.799107 2.568095
6 -1.183404 0.439032 1.830052
6 -1.169628 2.824401 3.359643
6 -0.715311 3.409462 4.596671

Benchmarking calculations: BLYP/def2-SVP

C60
E = -2283.552693
C 0.000000 -1.245734 3.357497
C -0.000000 -2.445927 2.615736
C 1.184763 -2.830880 1.845831
C 2.326215 -0.755833 2.615736
C 1.184763 -0.384953 3.357497
C 0.732224 1.007820 3.357497
C -0.732224 1.007820 3.357497
C -1.184763 -0.384953 3.357497
C -0.000000 1.245734 -3.357497
C 0.000000 2.445927 -2.615736
C -1.184763 2.830880 -1.845831
C -1.184763 0.384953 -3.357497
C -2.326215 2.001567 -1.845831
C -0.732224 -1.007820 -3.357497
C 0.732224 -1.007820 -3.357497
C 1.184763 0.384953 -3.357497
C -1.184763 -2.830880 1.845831
C 3.058439 -1.763653 0.600097
C 2.326215 -2.001567 1.845831
C 2.622443 1.593844 1.845831
C -0.705456 2.986617 1.845831
C -3.058439 0.251987 1.845831
C 1.184763 2.830880 -1.845831
C -3.058439 1.763653 -0.600097
C -2.326215 -2.001567 1.845831
C -2.622443 -2.363750 -0.600097
C 1.437680 -3.224531 -0.600097
C 3.510978 0.370880 -0.600097
C 0.732224 3.453747 -0.600097
C 2.622443 2.363750 0.600097
C 0.705456 -2.986617 -1.845831
C -1.437680 3.224531 0.600097
C -3.510978 -0.370880 0.600097
C -0.732224 -3.453747 0.600097
C 3.058439 -0.251987 -1.845831
C -2.622443 -1.593844 -1.845831
C 0.732224 -3.453747 0.600097
C 2.326215 0.755833 -2.615736
C -2.326215 -0.755833 2.615736
C 1.437680 1.978797 2.615736
C -1.437680 1.978797 2.615736
C -1.437680 -3.224531 -0.600097
C 1.437680 -1.978797 -2.615736
C 1.437680 3.224531 0.600097
C -2.622443 2.363750 0.600097
C -3.058439 -1.763653 0.600097
C 3.510978 -0.370880 0.600097
C 2.622443 -2.363750 -0.600097
C 3.058439 1.763653 -0.600097
C -0.732224 3.453747 -0.600097
C -3.510978 0.370880 -0.600097
C 2.622443 -1.593844 -1.845831
C -1.437680 -1.978797 -2.615736
C 2.326215 2.001567 -1.845831
C -2.326215 0.755833 -2.615736

C 3.058439 0.251987 1.845831
C 0.705456 2.986617 1.845831
C -2.622443 1.593844 1.845831
C -3.058439 -0.251987 -1.845831
C -0.705456 -2.986617 -1.845831

C60-
E = -2283.644874
C -0.278161 -3.381272 1.122699
C -0.132120 -1.625976 -3.177917
C 0.340907 -2.668609 -2.350510
C 1.697800 -2.596150 -1.789600
C 2.522222 -1.481730 -2.078952
C 2.033722 -0.406849 -2.937193
C 0.720371 -0.479373 -3.481853
C -0.537471 -3.278970 -1.355333
C 0.266242 -3.559518 -0.169304
C 1.651316 -3.145846 -0.439989
C -1.876923 -2.816314 -1.212794
C -2.439084 -2.627445 0.121331
C -1.661758 -2.905438 1.271137
C 0.527022 -2.793989 2.189556
C -0.348766 -1.935038 2.982983
C -1.705714 -2.011964 2.422570
C -3.289175 -1.432407 0.095407
C -3.331412 -0.567249 1.209723
C -2.526102 -0.858051 2.400113
C -2.033722 0.406849 2.937193
C -0.720371 0.479373 3.481853
C 0.128090 -0.709515 3.498995
C 1.513285 -0.294232 3.232133
C 2.360326 -1.123640 2.457016
C 1.867877 -2.391105 1.927895
C 2.429975 -2.570698 0.592619
C -3.242587 -0.879854 -1.259502
C -2.365340 -1.736736 -2.064233
C -1.515761 -1.149683 -3.033739
C -1.513285 0.294232 -3.232133
C -2.360326 1.123640 -2.457016
C -3.240076 0.518311 -1.451458
C -3.328782 0.883313 1.010508
C -3.283925 1.415672 -0.295734
C -2.522222 1.481730 2.078952
C -2.429975 2.570698 -0.592619
C -1.651316 3.145846 0.439989
C -1.697800 2.596150 1.789600
C 0.132120 1.625976 3.177917
C -0.340907 2.668609 2.350510
C 1.515761 1.149683 3.033739
C 0.537471 3.278970 1.355333
C 1.876923 2.816314 1.212794
C 2.365340 1.736736 2.064233
C 3.240076 -0.518311 1.451458
C 3.242587 0.879854 1.259502
C 3.283925 -1.415672 0.295734
C 3.328782 -0.883313 -1.010508
C 3.289175 1.432407 -0.095407
C 3.331412 0.567249 -1.209723
C 2.526102 0.858051 -2.400113
C 1.705714 2.011964 -2.422570
C 1.661758 2.905438 -1.271137
C 2.439084 2.627445 -0.121331
C 0.348766 1.935038 -2.982983
C -0.527022 2.793989 -2.189556
C 0.278161 3.381272 -1.122699
C -0.266242 3.559518 0.169304
C -1.867877 2.391105 -1.927895
C -0.128090 0.709515 -3.498995

C120-, B1
E = -4567.701106
C -2.587995 2.328709 4.330184
C -2.601724 1.584920 5.578944
C -3.036386 0.247117 5.578909
C -3.462643 -0.362669 4.330171
C -3.462643 0.362669 -4.330171
C -3.014540 1.742057 -4.330188
C -1.435182 1.975461 6.366889
C -2.322453 -0.754641 6.367032
C -3.014540 -1.742056 4.330188
C -3.036386 -0.247117 -5.578909
C -2.311475 1.984825 -5.579074
C -1.414908 3.181498 4.330341
C -2.601724 -1.584920 -5.578944
C -2.587994 -2.328708 -4.330184
C -1.435182 -1.975461 -6.366889
C -1.414908 -3.181498 -4.330340
C -0.731893 1.007331 7.122498
C -2.311475 -1.984824 5.579074
C -0.703336 -2.964476 -5.579112
C -1.173372 -2.811511 5.578987
C -1.184275 -0.384862 7.122542
C -2.322453 0.754641 -6.367032
C -1.173372 2.811511 -5.578987
C 0.731893 1.007331 7.122498
C -0.725122 3.405152 -4.330221
C -0.703336 2.964476 5.579112
C -0.000000 2.441914 -6.366993
C 0.725121 3.405151 -4.330221
C 0.703336 2.964476 5.579112
C -0.725121 -3.405151 4.330221
C -1.184275 0.384862 -7.122542
C 0.000000 -1.245344 7.122617
C 1.173372 2.811511 -5.578987
C 1.414908 3.181498 4.330340
C 1.435182 1.975461 6.366889
C 2.601724 1.584920 5.578944
C 1.184275 -0.384862 7.122542
C 0.000000 -2.441914 6.366993
C 1.173372 -2.811511 5.578987
C 2.587994 2.328708 4.330184
C 3.014540 1.742056 -4.330188
C 3.462643 0.362669 -4.330171
C 2.311475 1.984824 -5.579074
C -0.731893 -1.007331 -7.122498
C 2.311475 -1.984825 5.579074
C -0.000000 1.245344 -7.122617
C 1.184275 0.384862 -7.122542
C 2.322453 -0.754641 6.367032
C 0.725122 -3.405152 4.330221
C 1.414908 -3.181498 -4.330341
C 3.036386 0.247117 5.578909
C 3.462643 -0.362669 4.330171
C 2.322453 0.754641 -6.367032
C 3.036386 -0.247117 -5.578909
C 3.014540 -1.742057 4.330188
C 0.731893 -1.007331 -7.122498
C 0.703336 -2.964476 -5.579112
C 2.587995 -2.328709 -4.330184
C 1.435182 -1.975461 -6.366889
C 2.601724 -1.584920 -5.578944
C 1.439535 3.225306 -3.111544
C 0.722365 3.414624 -1.865739
C -0.722365 3.414625 -1.865739
C -1.439535 3.225306 -3.111545
C 2.622117 2.365705 -3.111513
C -2.622118 2.365705 -3.111513
C 0.731039 3.456133 3.111687
C -0.731040 3.456133 3.111687
C 3.059490 1.762737 3.111461
C 2.590488 2.337339 1.865703
C 1.422199 3.187133 1.865771
C -1.422200 3.187135 1.865771
C -2.590489 2.337340 1.865703
C -3.059490 1.762737 3.111461
C 3.511563 0.372437 3.111467
C 2.622118 -2.365705 3.111513
C 3.023215 -1.741986 1.865656
C 3.469652 -0.368004 1.865653
C 3.059490 -1.762737 -3.111461
C 3.511563 -0.372437 -3.111467
C 3.469652 0.368003 -1.865652
C 3.023214 1.741986 -1.865656
C 1.439535 -3.225306 3.111545
C 0.731040 -3.456133 -3.111687
C 1.422200 -3.187135 -1.865771
C 2.590489 -2.337340 -1.865703
C -0.731039 -3.456133 -3.111687
C -3.059490 -1.762737 -3.111461
C -2.590488 -2.337339 -1.865703
C -1.422199 -3.187133 -1.865771
C -2.622117 -2.365705 3.111513
C -1.439535 -3.225306 3.111544
C -3.511563 -0.372437 -3.111467
C -3.023215 1.741986 -1.865656
C -3.469652 0.368004 -1.865653
C -3.511563 0.372437 3.111467
C -0.722365 -3.414624 1.865739
C 0.722365 -3.414625 1.865739
C -1.411393 3.174068 -0.620233
C -2.581596 2.323091 -0.620230
C 2.581595 2.323091 -0.620230
C 1.411392 3.174067 -0.620233
C 3.006553 -1.737750 -0.620256
C 3.453803 -0.361678 -0.620240
C -0.723488 -3.397977 -0.620270
C 0.723489 -3.397978 -0.620270
C -3.453803 -0.361677 -0.620240
C -3.006552 -1.737750 -0.620256
C -3.469652 -0.368003 1.865652
C -3.023214 -1.741986 1.865656
C 0.723488 3.397977 0.620270
C -0.723489 3.397978 0.620270
C 3.453803 0.361677 0.620240
C 3.006552 1.737750 0.620256
C 1.411393 -3.174068 0.620233
C 2.581596 -2.323091 0.620230
C -2.581595 -2.323091 0.620230
C -1.411392 -3.174067 0.620233
C -3.006553 1.737750 0.620256
C -3.453803 0.361678 0.620240

C120-, C1
E = -4567.222119
C -7.624474 1.440911 1.186834
C -7.185095 0.732180 2.332133
C -7.185095 -0.732180 2.332133
C -7.624474 -1.440911 1.186834
C -8.068648 -0.705670 0.000000
C -8.068648 0.705670 0.000000
C -6.001771 -1.185761 3.057835
C -5.297494 -2.331693 2.624975
C -5.754108 -3.066775 1.438620
C -6.896461 -2.623135 0.734490
C -6.896461 -2.623135 -0.734490
C -5.754108 -3.066775 -1.438620
C -4.574589 -3.526033 -0.708737
C -4.574589 -3.526033 0.708737
C -3.394212 -3.066667 1.439497
C -2.255970 -2.606996 0.733267
C -2.255970 -2.606996 -0.733267
C -3.394212 -3.066667 -1.439497
C -5.297494 -2.331693 -2.624975
C -3.836664 -2.335421 -2.620205
C -3.836664 -2.335421 2.620205
C -3.121450 -1.185317 3.034600
C -1.937593 -0.743341 2.306829
C -1.500787 -1.462791 1.189219
C -0.799063 -0.803683 0.000000
C -1.500787 -1.462791 -1.189219
C -1.937593 -0.743341 -2.306829
C -3.121450 -1.185317 -3.034600
C -7.624474 -1.440911 -1.186834
C -7.185095 -0.732180 -2.332133
C -6.001771 -1.185761 -3.057835

C -5.263785 0.000000 -3.503018
C -3.853827 0.000000 -3.483517
C -0.799063 0.803683 0.000000
C -1.500787 1.462791 1.189219
C -1.937593 0.743341 2.306829
C -3.121450 1.185317 3.034600
C -3.853827 -0.000000 3.483517
C -5.263785 -0.000000 3.503018
C -6.001771 1.185761 3.057835
C -5.297494 2.331693 2.624975
C -3.836664 2.335421 2.620205
C -2.255970 2.606996 0.733267
C -3.394212 3.066667 1.439497
C -6.896461 2.623135 0.734490
C -5.754108 3.066775 1.438620
C -4.574589 3.526033 0.708737
C -1.500787 1.462791 -1.189219
C -1.937593 0.743341 -2.306829
C -3.121450 1.185317 -3.034600
C -7.624474 1.440911 -1.186834
C -7.185095 0.732180 -2.332133
C -6.001771 1.185761 -3.057835
C -5.297494 2.331693 -2.624975
C -3.836664 2.335421 -2.620205
C -2.255970 2.606996 -0.733267
C -3.394212 3.066667 -1.439497
C -4.574589 3.526033 -0.708737
C -5.754108 3.066775 -1.438620
C -6.896461 2.623135 -0.734490
C 1.500787 -1.462791 -1.189219
C 1.937593 -0.743341 -2.306829
C 1.937593 0.743341 -2.306829
C 1.500787 1.462791 -1.189219
C 0.799063 0.803683 0.000000
C 0.799063 -0.803683 0.000000
C 2.255970 2.606996 -0.733267
C 3.394212 3.066667 -1.439497
C 3.836664 2.335421 -2.620205
C 3.121450 1.185317 -3.034600
C 4.574589 3.526033 -0.708737
C 4.574589 3.526033 0.708737
C 3.394212 3.066667 1.439497
C 2.255970 2.606996 0.733267
C 1.500787 1.462791 1.189219
C 1.937593 0.743341 2.306829
C 3.121450 1.185317 3.034600
C 3.836664 2.335421 2.620205
C 1.937593 -0.743341 2.306829
C 3.121450 -1.185317 3.034600
C 3.853827 0.000000 3.483517
C 5.263785 0.000000 3.503018
C 6.001771 -1.185761 3.057835
C 5.297494 -2.331693 2.624975
C 3.836664 -2.335421 2.620205
C 3.394212 -3.066667 1.439497
C 2.255970 -2.606996 0.733267
C 1.500787 -1.462791 1.189219
C 6.001771 1.185761 3.057835
C 5.297494 2.331693 2.624975
C 5.754108 3.066775 1.438620
C 5.297494 2.331693 -2.624975
C 5.754108 3.066775 -1.438620
C 6.896461 2.623135 -0.734490
C 6.896461 2.623135 0.734490
C 6.001771 1.185761 -3.057835
C 5.263785 -0.000000 -3.503018
C 3.853827 -0.000000 -3.483517
C 3.121450 -1.185317 -3.034600
C 7.185095 0.732180 -2.332133
C 7.624474 1.440911 -1.186834
C 7.185095 0.732180 2.332133
C 7.624474 1.440911 1.186834
C 8.068648 0.705670 0.000000
C 8.068648 -0.705670 0.000000
C 7.624474 -1.440911 -1.186834
C 7.185095 -0.732180 -2.332133
C 6.001771 -1.185761 -3.057835
C 5.297494 -2.331693 -2.624975
C 3.836664 -2.335421 -2.620205
C 7.624474 -1.440911 1.186834
C 7.185095 -0.732180 2.332133
C 6.896461 -2.623135 0.734490
C 6.896461 -2.623135 -0.734490
C 5.754108 -3.066775 -1.438620
C 4.574589 -3.526033 -0.708737
C 3.394212 -3.066667 -1.439497
C 2.255970 -2.606996 -0.733267
C 4.574589 -3.526033 0.708737
C 5.754108 -3.066775 1.438620

C120-, P1
E = -4567.241200
C 7.458201 1.185166 1.439462
C 7.032890 2.333065 0.733817
C 7.032890 2.333065 -0.733817
C 7.458201 1.185166 -1.439462
C 7.908472 -0.000000 -0.706166
C 7.908472 -0.000000 0.706166
C 5.858361 3.080597 -1.184460
C 5.132530 2.632502 -2.316777
C 5.563479 1.437205 -3.031912
C 6.717861 0.730738 -2.617226
C 6.717861 -0.730738 -2.617226
C 5.563479 -1.437205 -3.031912
C 4.357720 -0.699412 -3.414345
C 4.357720 0.699412 -3.414345
C 3.184389 1.423989 -2.929602
C 2.017216 0.745259 -2.442650
C 2.017216 -0.745259 -2.442650
C 3.184389 -1.423989 -2.929602
C 5.132530 -2.632502 -2.316777
C 3.668113 -2.638694 -2.295250
C 3.668113 2.638694 -2.295250
C 2.985844 3.152278 -1.171519
C 1.754983 2.529143 -0.749661
C 1.223584 1.423482 -1.434471
C 0.000000 0.776616 -0.819238
C 1.223584 -1.423482 -1.434471
C 1.754983 -2.529143 -0.749661
C 2.985844 -3.152278 -1.171519
C 7.458201 -1.185166 -1.439462
C 7.032890 -2.333065 -0.733817
C 5.858361 -3.080597 -1.184460
C 5.146129 -3.558888 0.000000
C 3.733438 -3.595409 0.000000
C -0.000000 0.776616 0.819238
C 1.223584 1.423482 1.434471
C 1.754983 2.529143 0.749661
C 2.985844 3.152278 1.171519
C 3.733438 3.595409 0.000000
C 5.146129 3.558888 0.000000
C 5.858361 3.080597 1.184460
C 5.132530 2.632502 2.316777
C 3.668113 2.638694 2.295250
C 2.017216 0.745259 2.442650
C 3.184389 1.423989 2.929602
C 6.717861 0.730738 2.617226
C 5.563479 1.437205 3.031912
C 4.357720 0.699412 3.414345
C 1.223584 -1.423482 1.434471
C 1.754983 -2.529143 0.749661
C 2.985844 -3.152278 1.171519
C 7.458201 -1.185166 1.439462
C 7.032890 -2.333065 0.733817
C 5.858361 -3.080597 1.184460
C 5.132530 -2.632502 2.316777
C 3.668113 -2.638694 2.295250
C 2.017216 -0.745259 2.442650
C 3.184389 -1.423989 2.929602
C 4.357720 -0.699412 3.414345
C 5.563479 -1.437205 3.031912
C 6.717861 -0.730738 2.617226
C -1.223584 -1.423482 -1.434471
C -1.754983 -2.529143 -0.749661
C -1.754983 -2.529143 0.749661
C -1.223584 -1.423482 1.434471
C -0.000000 -0.776616 0.819238
C -0.000000 -0.776616 -0.819238
C -2.017216 -0.745259 2.442650
C -3.184389 -1.423989 2.929602
C -3.668113 -2.638694 2.295250
C -2.985844 -3.152278 1.171519
C -4.357720 -0.699412 3.414345
C -4.357720 0.699412 3.414345
C -3.184389 1.423989 2.929602
C -2.017216 0.745259 2.442650
C -1.223584 1.423482 1.434471
C -1.754983 2.529143 0.749661
C -2.985844 3.152278 1.171519
C -3.668113 2.638694 2.295250
C -1.754983 2.529143 -0.749661
C -2.985844 3.152278 -1.171519
C -3.733438 3.595409 0.000000
C -5.146129 3.558888 0.000000
C -5.858361 3.080597 -1.184460
C -5.132530 2.632502 -2.316777
C -3.668113 2.638694 -2.295250
C -3.184389 1.423989 -2.929602
C -2.017216 0.745259 -2.442650
C -1.223584 1.423482 -1.434471
C -5.858361 3.080597 1.184460
C -5.132530 2.632502 2.316777
C -5.563479 1.437205 3.031912
C -5.132530 -2.632502 2.316777
C -5.563479 -1.437205 3.031912
C -6.717861 -0.730738 2.617226
C -6.717861 0.730738 2.617226
C -5.858361 -3.080597 1.184460
C -5.146129 -3.558888 -0.000000
C -3.733438 -3.595409 -0.000000
C -2.985844 -3.152278 -1.171519
C -7.032890 -2.333065 0.733817
C -7.458201 -1.185166 1.439462
C -7.032890 2.333065 0.733817
C -7.458201 1.185166 1.439462
C -7.908472 -0.000000 0.706166
C -7.908472 0.000000 -0.706166
C -7.458201 -1.185166 -1.439462
C -7.032890 -2.333065 -0.733817
C -5.858361 -3.080597 -1.184460
C -5.132530 -2.632502 -2.316777
C -3.668113 -2.638694 -2.295250
C -7.458201 1.185166 -1.439462
C -7.032890 2.333065 -0.733817
C -6.717861 0.730738 -2.617226
C -6.717861 -0.730738 -2.617226
C -5.563479 -1.437205 -3.031912
C -4.357720 -0.699412 -3.414345
C -3.184389 -1.423989 -2.929602
C -2.017216 -0.745259 -2.442650
C -4.357720 0.699412 -3.414345
C -5.563479 1.437205 -3.031912

C120-, V1
E = -4567.227736
C 0.000000 2.408314 2.487520
C -0.000000 -2.408314 2.487520
C 0.000000 7.548492 -2.486235
C -0.000000 -7.548492 -2.486235
C 1.186964 -8.329915 -0.439522
C 2.332924 -7.582426 -0.801750
C 2.333503 -6.793814 -2.032258
C 1.187343 -6.776620 -2.863271
C 0.000000 -8.310297 -1.298549
C 3.063366 -5.551464 -1.773790

C 2.625762 -4.339420 -2.351354
C 1.437442 -4.322538 -3.212163
C 0.733931 -5.522431 -3.460231
C -0.733931 -5.522431 -3.460231
C -1.437442 -4.322538 -3.212163
C -0.709586 -3.080236 -2.960737
C 0.709586 -3.080236 -2.960737
C 1.438436 -2.333178 -1.937993
C 0.733409 -1.614150 -0.949124
C -0.733409 -1.614150 -0.949124
C -1.438436 -2.333178 -1.937993
C -2.625762 -4.339420 -2.351354
C -2.627121 -3.107836 -1.562987
C 2.627121 -3.107836 -1.562987
C 3.063493 -3.127890 -0.220423
C 2.335189 -2.374018 0.802627
C 1.186884 -1.631423 0.439482
C 0.000000 -1.652397 1.297676
C -1.186884 -1.631423 0.439482
C -2.335189 -2.374018 0.802627
C -3.063493 -3.127890 -0.220423
C -1.187343 -6.776620 -2.863271
C -2.333503 -6.793814 -2.032258
C -3.063366 -5.551464 -1.773790
C -3.516849 -5.573145 -0.381165
C -3.515930 -4.384657 0.380276
C 1.187921 -3.179533 2.864464
C 2.333728 -3.162533 2.032167
C 3.061743 -4.406206 1.773217
C 3.515930 -4.384657 0.380276
C 3.516849 -5.573145 -0.381165
C 3.062861 -6.829179 0.220254
C 2.625819 -6.849302 1.562591
C 2.625238 -5.617623 2.352061
C 0.734348 -4.433825 3.459586
C 1.438068 -5.634197 3.213162
C 0.733788 -8.349428 0.949853
C 1.437670 -7.623931 1.936642
C 0.709218 -6.875693 2.960181
C -1.187921 -3.179533 2.864464
C -2.333728 -3.162533 2.032167
C -3.061743 -4.406206 1.773217
C -1.186964 -8.329915 -0.439522
C -2.332924 -7.582426 -0.801750
C -3.062861 -6.829179 0.220254
C -2.625819 -6.849302 1.562591
C -2.625238 -5.617623 2.352061
C -0.734348 -4.433825 3.459586
C -1.438068 -5.634197 3.213162
C -0.709218 -6.875693 2.960181
C -1.437670 -7.623931 1.936642
C -0.733788 -8.349428 0.949853
C -1.186884 1.631423 0.439482
C -2.335189 2.374018 0.802627
C -2.333728 3.162533 2.032167
C -1.187921 3.179533 2.864464
C 0.000000 1.652397 1.297676
C -0.734348 4.433825 3.459586
C -1.438068 5.634197 3.213162
C -2.625238 5.617623 2.352061
C -3.061743 4.406206 1.773217
C -0.709218 6.875693 2.960181
C 0.709218 6.875693 2.960181
C 1.438068 5.634197 3.213162
C 0.734348 4.433825 3.459586
C 1.187921 3.179533 2.864464
C 2.333728 3.162533 2.032167
C 3.061743 4.406206 1.773217
C 2.625238 5.617623 2.352061
C 2.335189 2.374018 0.802627
C 3.063493 3.127890 -0.220423
C 3.515930 4.384657 0.380276
C 3.516849 5.573145 -0.381165
C 3.063366 5.551464 -1.773790
C 2.625762 4.339420 -2.351354
C 2.627121 3.107836 -1.562987
C 1.438436 2.333178 -1.937993
C 0.733409 1.614150 -0.949124
C 1.186884 1.631423 0.439482
C 3.062861 6.829179 0.220254
C 2.625819 6.849302 1.562591
C 1.437670 7.623931 1.936642
C -2.625819 6.849302 1.562591
C -1.437670 7.623931 1.936642
C -0.733788 8.349428 0.949853
C 0.733788 8.349428 0.949853
C -3.062861 6.829179 0.220254
C -3.516849 5.573145 -0.381165
C -3.515930 4.384657 0.380276
C -3.063493 3.127890 -0.220423
C -2.332924 7.582426 -0.801750
C -1.186964 8.329915 -0.439522
C 2.332924 7.582426 -0.801750
C 1.186964 8.329915 -0.439522
C 0.000000 8.310297 -1.298549
C -1.187343 6.776620 -2.863271
C -2.333503 6.793814 -2.032258
C -3.063366 5.551464 -1.773790
C -2.625762 4.339420 -2.351354
C -2.627121 3.107836 -1.562987
C 1.187343 6.776620 -2.863271
C 2.333503 6.793814 -2.032258
C 0.733931 5.522431 -3.460231
C -0.733931 5.522431 -3.460231
C -1.437442 4.322538 -3.212163
C -0.709586 3.080236 -2.960737
C -1.438436 2.333178 -1.937993
C -0.733409 1.614150 -0.949124
C 0.709586 3.080236 -2.960737
C 1.437442 4.322538 -3.212163

Benchmarking calculations: BMK/def2-SVP

C60
E = -2282.805737
C 0.000000 -1.239539 3.334373
C -0.000000 -2.428015 2.599854
C 1.178871 -2.811054 1.833777
C 2.309180 -0.750298 2.599854
C 1.178871 -0.383039 3.334373
C 0.728583 1.002808 3.334373
C -0.728583 1.002808 3.334373
C -1.178871 -0.383039 3.334373
C -0.000000 1.239539 -3.334373
C 0.000000 2.428015 -2.599854
C -1.178871 2.811054 -1.833777
C -1.178871 0.383039 -3.334373
C -2.309180 1.989837 -1.833777
C -0.728583 -1.002808 -3.334373
C 0.728583 -1.002808 -3.334373
C 1.178871 0.383039 -3.334373
C -1.178871 -2.811054 1.833777
C 3.037762 -1.753106 0.594238
C 2.309180 -1.989837 1.833777
C 2.606023 1.581267 1.833777
C -0.698569 2.967113 1.833777
C -3.037762 0.252510 1.833777
C 1.178871 2.811054 -1.833777
C -3.037762 1.753106 -0.594238
C -2.309180 -1.989837 1.833777
C -2.606023 -2.347344 -0.594238
C 1.427152 -3.203844 -0.594238
C 3.488051 0.367259 -0.594238
C 0.728583 3.430823 -0.594238
C 2.606023 2.347344 0.594238
C 0.698569 -2.967113 -1.833777
C -1.427152 3.203844 0.594238
C -3.488051 -0.367259 0.594238
C -0.728583 -3.430823 0.594238
C 3.037762 -0.252510 -1.833777
C -2.606023 -1.581267 -1.833777
C 0.728583 -3.430823 0.594238
C 2.309180 0.750298 -2.599854
C -2.309180 -0.750298 2.599854
C 1.427152 1.964306 2.599854
C -1.427152 1.964306 2.599854
C -1.427152 -3.203844 -0.594238
C 1.427152 -1.964306 -2.599854
C 1.427152 3.203844 0.594238
C -2.606023 2.347344 0.594238
C -3.037762 -1.753106 0.594238
C 3.488051 -0.367259 0.594238
C 2.606023 -2.347344 -0.594238
C 3.037762 1.753106 -0.594238
C -0.728583 3.430823 -0.594238
C -3.488051 0.367259 -0.594238
C 2.606023 -1.581267 -1.833777
C -1.427152 -1.964306 -2.599854
C 2.309180 1.989837 -1.833777
C -2.309180 0.750298 -2.599854
C 3.037762 0.252510 1.833777
C 0.698569 2.967113 1.833777
C -2.606023 1.581267 1.833777
C -3.037762 -0.252510 -1.833777
C -0.698569 -2.967113 -1.833777

C60-
E = -2282.903222
C -0.274260 -3.360164 1.113162
C -0.130665 -1.619404 -3.168975
C 0.337269 -2.660619 -2.341923
C 1.680534 -2.575157 -1.774929
C 2.494732 -1.473362 -2.053335
C 2.010478 -0.399492 -2.914685
C 0.710926 -0.481048 -3.457781
C -0.530223 -3.253363 -1.350067
C 0.270346 -3.536147 -0.162463
C 1.644023 -3.130771 -0.429504
C -1.855784 -2.795528 -1.198376
C -2.418618 -2.613045 0.120027
C -1.648418 -2.891433 1.268295
C 0.532382 -2.771892 2.173620
C -0.343876 -1.920466 2.965927
C -1.691229 -2.004866 2.408618
C -3.255228 -1.417442 0.092684
C -3.296840 -0.560087 1.196002
C -2.502828 -0.852336 2.386406
C -2.010478 0.399492 2.914685
C -0.710926 0.481048 3.457781
C 0.133103 -0.709657 3.476578
C 1.507056 -0.301203 3.214282
C 2.349748 -1.118004 2.448079
C 1.853405 -2.378036 1.916849
C 2.420586 -2.561296 0.589375
C -3.219720 -0.865828 -1.255101
C -2.340575 -1.713240 -2.049834
C -1.501299 -1.140350 -3.009868
C -1.507056 0.301203 -3.214282
C -2.349748 1.118004 -2.448079
C -3.223945 0.521742 -1.448786
C -3.304729 0.882156 0.996146
C -3.267478 1.414052 -0.299732
C -2.494732 1.473362 2.053335
C -2.420586 2.561296 -0.589375
C -1.644023 3.130771 0.429504
C -1.680534 2.575157 1.774929
C 0.130665 1.619404 3.168975
C -0.337269 2.660619 2.341923
C 1.501299 1.140350 3.009868
C 0.530223 3.253363 1.350067
C 1.855784 2.795528 1.198376
C 2.340575 1.713240 2.049834
C 3.223945 -0.521742 1.448786

C 3.219720 0.865828 1.255101
C 3.267478 -1.414052 0.299732
C 3.304729 -0.882156 -0.996146
C 3.255228 1.417442 -0.092684
C 3.296840 0.560087 -1.196002
C 2.502828 0.852336 -2.386406
C 1.691229 2.004866 -2.408618
C 1.648418 2.891433 -1.268295
C 2.418618 2.613045 -0.120027
C 0.343876 1.920466 -2.965927
C -0.532382 2.771892 -2.173620
C 0.274260 3.360164 -1.113162
C -0.270346 3.536147 0.162463
C -1.853405 2.378036 -1.916849
C -0.133103 0.709657 -3.476578

C120-, B1
E = -4566.238972
C -2.571827 2.314445 4.302640
C -2.583133 1.572079 5.544168
C -3.013459 0.247212 5.544333
C -3.440893 -0.360214 4.302754
C -3.440893 0.360214 -4.302754
C -2.995500 1.730893 -4.302568
C -1.423958 1.959826 6.329363
C -2.303729 -0.748529 6.329162
C -2.995500 -1.730893 4.302568
C -3.013459 -0.247212 -5.544333
C -2.292934 1.970786 -5.543884
C -1.405878 3.161401 4.302957
C -2.583133 -1.572079 -5.544168
C -2.571827 -2.314445 -4.302640
C -1.423958 -1.959826 -6.329363
C -1.405878 -3.161401 -4.302957
C -0.728044 1.002321 7.079624
C -2.292934 -1.970786 5.543884
C -0.696490 -2.942166 -5.544687
C -1.165898 -2.789698 5.543995
C -1.177906 -0.382985 7.079393
C -2.303729 0.748529 -6.329162
C -1.165898 2.789698 -5.543995
C 0.728044 1.002321 7.079624
C -0.720770 3.384283 -4.302592
C -0.696490 2.942166 5.544687
C 0.000000 2.422597 -6.328570
C 0.720770 3.384283 -4.302592
C 0.696490 2.942166 5.544687
C -0.720770 -3.384283 4.302592
C -1.177906 0.382985 -7.079393
C 0.000000 -1.239055 7.079105
C 1.165898 2.789698 -5.543995
C 1.405878 3.161401 4.302957
C 1.423958 1.959826 6.329363
C 2.583133 1.572079 5.544168
C 1.177906 -0.382985 7.079393
C 0.000000 -2.422597 6.328570
C 1.165898 -2.789698 5.543995
C 2.571827 2.314445 4.302640
C 2.995500 1.730893 -4.302568
C 3.440893 0.360214 -4.302754
C 2.292934 1.970786 -5.543884
C -0.728044 -1.002321 -7.079624
C 2.292934 -1.970786 5.543884
C 0.000000 1.239055 -7.079105
C 1.177906 0.382985 -7.079393
C 2.303729 -0.748529 6.329162
C 0.720770 -3.384283 4.302592
C 1.405878 -3.161401 -4.302957
C 3.013459 0.247212 5.544333
C 3.440893 -0.360214 4.302754
C 2.303729 0.748529 -6.329162
C 3.013459 -0.247212 -5.544333
C 2.995500 -1.730893 4.302568
C 0.728044 -1.002321 -7.079624
C 0.696490 -2.942166 -5.544687
C 2.571827 -2.314445 -4.302640
C 1.423958 -1.959826 -6.329363
C 2.583133 -1.572079 -5.544168
C 1.430351 3.206813 -3.095397
C 0.715780 3.391991 -1.854946
C -0.715780 3.391991 -1.854946
C -1.430351 3.206813 -3.095397
C 2.607358 2.351093 -3.095330
C -2.607358 2.351093 -3.095330
C 0.727521 3.435271 3.095484
C -0.727521 3.435271 3.095484
C 3.041321 1.753356 3.095235
C 2.572240 2.323132 1.854968
C 1.414211 3.164907 1.855119
C -1.414211 3.164907 1.855119
C -2.572240 2.323132 1.854968
C -3.041321 1.753356 3.095235
C 3.491059 0.369416 3.095388
C 2.607358 -2.351093 3.095330
C 3.003937 -1.728747 1.854930
C 3.446118 -0.367053 1.854993
C 3.041321 -1.753356 -3.095235
C 3.491059 -0.369416 -3.095388
C 3.446118 0.367053 -1.854993
C 3.003937 1.728747 -1.854930
C 1.430351 -3.206813 3.095397
C 0.727521 -3.435271 -3.095484
C 1.414211 -3.164907 -1.855119
C 2.572240 -2.323132 -1.854968
C -0.727521 -3.435271 -3.095484
C -3.041321 -1.753356 -3.095235
C -2.572240 -2.323132 -1.854968
C -1.414211 -3.164907 -1.855119
C -2.607358 -2.351093 3.095330
C -1.430351 -3.206813 3.095397
C -3.491059 -0.369416 -3.095388
C -3.003937 1.728747 -1.854930
C -3.446118 0.367053 -1.854993
C -3.491059 0.369416 3.095388
C -0.715780 -3.391991 1.854946
C 0.715780 -3.391991 1.854946
C -1.404018 3.157614 -0.614430
C -2.568493 2.311164 -0.614463
C 2.568493 2.311164 -0.614463
C 1.404018 3.157614 -0.614430
C 2.991246 -1.728775 -0.614487
C 3.435938 -0.359713 -0.614514
C -0.719618 -3.380121 -0.614598
C 0.719618 -3.380121 -0.614598
C -3.435938 -0.359713 -0.614514
C -2.991246 -1.728775 -0.614487
C -3.446118 -0.367053 1.854993
C -3.003937 -1.728747 1.854930
C 0.719618 3.380121 0.614598
C -0.719618 3.380121 0.614598
C 3.435938 0.359713 0.614514
C 2.991246 1.728775 0.614487
C 1.404018 -3.157614 0.614430
C 2.568493 -2.311164 0.614463
C -2.568493 -2.311164 0.614463
C -1.404018 -3.157614 0.614430
C -2.991246 1.728775 0.614487
C -3.435938 0.359713 0.614514

C120-, C1
E = -4565.758457
6 -1.430216 7.581430 -1.177680
6 -0.728952 7.143109 -2.306241
6 0.728952 7.143109 -2.306241
6 1.430216 7.581430 -1.177680
6 0.698713 8.031380 -0.000014
6 -0.698713 8.031380 -0.000014
6 1.176641 5.962392 -3.031492
6 2.314404 5.269338 -2.599270
6 3.045860 5.726270 -1.428633
6 2.612230 6.862144 -0.726818
6 2.612232 6.862152 0.726801
6 3.045865 5.726281 1.428625
6 3.493481 4.548726 0.698327
6 3.493480 4.548719 -0.698327
6 3.049405 3.366780 -1.431943
6 2.599849 2.236501 -0.727053
6 2.599847 2.236509 0.727070
6 3.049400 3.366791 1.431951
6 2.314403 5.269356 2.599262
6 2.319263 3.810296 2.594756
6 2.319262 3.810279 -2.594748
6 1.176590 3.105566 -3.007721
6 0.742578 1.924403 -2.282329
6 1.450105 1.494983 -1.176262
6 0.794490 0.786599 0.000015
6 1.450104 1.494991 1.176288
6 0.742580 1.924419 2.282351
6 1.176591 3.105585 3.007736
6 1.430218 7.581438 1.177655
6 0.728951 7.143125 2.306219
6 1.176641 5.962412 3.031477
6 0.000000 5.233727 3.479059
6 0.000000 3.828745 3.458401
6 -0.794490 0.786599 0.000015
6 -1.450105 1.494983 -1.176262
6 -0.742578 1.924403 -2.282329
6 -1.176590 3.105566 -3.007721
6 0.000000 3.828723 -3.458393
6 0.000000 5.233705 -3.479068
6 -1.176641 5.962392 -3.031492
6 -2.314404 5.269338 -2.599270
6 -2.319262 3.810279 -2.594748
6 -2.599849 2.236501 -0.727053
6 -3.049405 3.366780 -1.431943
6 -2.612230 6.862144 -0.726818
6 -3.045860 5.726270 -1.428633
6 -3.493480 4.548719 -0.698327
6 -1.450104 1.494991 1.176288
6 -0.742580 1.924419 2.282351
6 -1.176591 3.105585 3.007736
6 -1.430218 7.581438 1.177655
6 -0.728951 7.143125 2.306219
6 -1.176641 5.962412 3.031477
6 -2.314403 5.269356 2.599262
6 -2.319263 3.810296 2.594756
6 -2.599847 2.236509 0.727070
6 -3.049400 3.366791 1.431951
6 -3.493481 4.548726 0.698327
6 -3.045865 5.726281 1.428625
6 -2.612232 6.862152 0.726801
6 1.450104 -1.494991 1.176288
6 0.742580 -1.924419 2.282351
6 -0.742580 -1.924419 2.282351
6 -1.450104 -1.494991 1.176288
6 -0.794490 -0.786599 0.000015
6 0.794490 -0.786599 0.000015
6 -2.599847 -2.236509 0.727070
6 -3.049400 -3.366791 1.431951
6 -2.319263 -3.810296 2.594756
6 -1.176591 -3.105585 3.007736
6 -3.493481 -4.548726 0.698327
6 -3.493480 -4.548719 -0.698327
6 -3.049405 -3.366780 -1.431943
6 -2.599849 -2.236501 -0.727053
6 -1.450105 -1.494983 -1.176262
6 -0.742578 -1.924403 -2.282329
6 -1.176590 -3.105566 -3.007721
6 -2.319262 -3.810279 -2.594748
6 0.742578 -1.924403 -2.282329
6 1.176590 -3.105566 -3.007721
6 0.000000 -3.828723 -3.458393
6 0.000000 -5.233705 -3.479068
6 1.176641 -5.962392 -3.031492
6 2.314404 -5.269338 -2.599270

6 2.319262 -3.810279 -2.594748
6 3.049405 -3.366780 -1.431943
6 2.599849 -2.236501 -0.727053
6 1.450105 -1.494983 -1.176262
6 -1.176641 -5.962392 -3.031492
6 -2.314404 -5.269338 -2.599270
6 -3.045860 -5.726270 -1.428633
6 -2.314403 -5.269356 2.599262
6 -3.045865 -5.726281 1.428625
6 -2.612232 -6.862152 0.726801
6 -2.612230 -6.862144 -0.726818
6 -1.176641 -5.962412 3.031477
6 0.000000 -5.233727 3.479059
6 0.000000 -3.828745 3.458401
6 1.176591 -3.105585 3.007736
6 -0.728951 -7.143125 2.306219
6 -1.430218 -7.581438 1.177655
6 -0.728952 -7.143109 -2.306241
6 -1.430216 -7.581430 -1.177680
6 -0.698713 -8.031380 -0.000014
6 0.698713 -8.031380 -0.000014
6 1.430218 -7.581438 1.177655
6 0.728951 -7.143125 2.306219
6 1.176641 -5.962412 3.031477
6 2.314403 -5.269356 2.599262
6 2.319263 -3.810296 2.594756
6 1.430216 -7.581430 -1.177680
6 0.728952 -7.143109 -2.306241
6 2.612230 -6.862144 -0.726818
6 2.612232 -6.862152 0.726801
6 3.045865 -5.726281 1.428625
6 3.493481 -4.548726 0.698327
6 3.049400 -3.366791 1.431951
6 2.599847 -2.236509 0.727070
6 3.493480 -4.548719 -0.698327
6 3.045860 -5.726270 -1.428633

C120-, P1
E = -4565.779161
C 7.408293 1.179151 1.429209
C 6.985596 2.314967 0.729861
C 6.985596 2.314967 -0.729861
C 7.408293 1.179151 -1.429209
C 7.857263 -0.000000 -0.699006
C 7.857263 -0.000000 0.699006
C 5.815579 3.056990 -1.177786
C 5.097879 2.612533 -2.299249
C 5.527729 1.425728 -3.011420
C 6.672455 0.726416 -2.600451
C 6.672455 -0.726416 -2.600451
C 5.527729 -1.425728 -3.011420
C 4.328566 -0.692725 -3.392803
C 4.328566 0.692725 -3.392803
C 3.158010 1.414864 -2.913496
C 1.999627 0.742625 -2.434600
C 1.999627 -0.742625 -2.434600
C 3.158010 -1.414864 -2.913496
C 5.097879 -2.612533 -2.299249
C 3.639653 -2.619031 -2.280394
C 3.639653 2.619031 -2.280397
C 2.964507 3.126806 -1.164492
C 1.738210 2.511340 -0.748300
C 1.215789 1.413405 -1.423842
C 0.000000 0.769465 -0.803870
C 1.215789 -1.413405 -1.423842
C 1.738210 -2.511340 -0.748300
C 2.964507 -3.126806 -1.164492
C 7.408293 -1.179151 -1.429209
C 6.985596 -2.314967 -0.729861
C 5.815579 -3.056990 -1.177786
C 5.107617 -3.533672 0.000000
C 3.707619 -3.568919 0.000000
C -0.000000 0.769465 0.803870
C 1.215789 1.413405 1.423842
C 1.738210 2.511340 0.748300
C 2.964507 3.126806 1.164492
C 3.707619 3.568919 0.000000
C 5.107617 3.533672 0.000000
C 5.815579 3.056990 1.177786
C 5.097879 2.612533 2.299249
C 3.639653 2.619031 2.280394
C 1.999627 0.742625 2.434600
C 3.158010 1.414864 2.913496
C 6.672455 0.726416 2.600451
C 5.527729 1.425728 3.011420
C 4.328566 0.692725 3.392803
C 1.215789 -1.413405 1.423842
C 1.738210 -2.511340 0.748300
C 2.964507 -3.126806 1.164492
C 7.408293 -1.179151 1.429209
C 6.985596 -2.314967 0.729861
C 5.815579 -3.056990 1.177786
C 5.097879 -2.612533 2.299249
C 3.639653 -2.619031 2.280394
C 1.999627 -0.742625 2.434600
C 3.158010 -1.414864 2.913496
C 4.328566 -0.692725 3.392803
C 5.527729 -1.425728 3.011420
C 6.672455 -0.726416 2.600451
C -1.215789 -1.413405 -1.423842
C -1.738210 -2.511340 -0.748300
C -1.738210 -2.511340 0.748300
C -1.215789 -1.413405 1.423842
C -0.000000 -0.769465 0.803870
C -0.000000 -0.769465 -0.803870
C -1.999627 -0.742625 2.434600
C -3.158010 -1.414864 2.913496
C -3.639653 -2.619031 2.280394
C -2.964507 -3.126806 1.164492
C -4.328566 -0.692725 3.392803
C -4.328566 0.692725 3.392803
C -3.158010 1.414864 2.913496
C -1.999627 0.742625 2.434600
C -1.215789 1.413405 1.423842
C -1.738210 2.511340 0.748300
C -2.964507 3.126806 1.164492
C -3.639653 2.619031 2.280394
C -1.738210 2.511340 -0.748300
C -2.964507 3.126806 -1.164492
C -3.707619 3.568919 0.000000
C -5.107617 3.533672 0.000000
C -5.815579 3.056990 -1.177786
C -5.097879 2.612533 -2.299249
C -3.639653 2.619031 -2.280394
C -3.158010 1.414864 -2.913496
C -1.999627 0.742625 -2.434600
C -1.215789 1.413405 -1.423842
C -5.815579 3.056990 1.177786
C -5.097879 2.612533 2.299249
C -5.527729 1.425728 3.011420
C -5.097879 -2.612533 2.299249
C -5.527729 -1.425728 3.011420
C -6.672455 -0.726416 2.600451
C -6.672455 0.726416 2.600451
C -5.815579 -3.056990 1.177786
C -5.107617 -3.533672 -0.000000
C -3.707619 -3.568919 -0.000000
C -2.964507 -3.126806 -1.164492
C -6.985596 -2.314967 0.729861
C -7.408293 -1.179151 1.429209
C -6.985596 2.314967 0.729861
C -7.408293 1.179151 1.429209
C -7.857263 -0.000000 0.699006
C -7.857263 0.000000 -0.699006
C -7.408293 -1.179151 -1.429209
C -6.985596 -2.314967 -0.729861
C -5.815579 -3.056990 -1.177786
C -5.097879 -2.612533 -2.299249
C -3.639653 -2.619031 -2.280394
C -7.408293 1.179151 -1.429209
C -6.985596 2.314967 -0.729861
C -6.672455 0.726416 -2.600451
C -6.672455 -0.726416 -2.600451
C -5.527729 -1.425728 -3.011420
C -4.328566 -0.692725 -3.392803
C -3.158010 -1.414864 -2.913496
C -1.999627 -0.742625 -2.434600
C -4.328566 0.692725 -3.392803
C -5.527729 1.425728 -3.011420

C120-, V1
E = -4565.733535
C 1.502946 -1.913756 2.339698
C 1.502946 -1.913756 -2.339698
C -1.501906 1.911510 7.530807
C -1.501906 1.911510 -7.530807
C 0.699441 1.046779 -8.259285
C 1.349338 2.038321 -7.516632
C 0.576836 3.007623 -6.754163
C -0.826733 2.945140 -6.761565
C -0.758947 0.981496 -8.266732
C 1.285644 3.265622 -5.511819
C 0.573847 3.452234 -4.320452
C -0.886038 3.387731 -4.328169
C -1.567129 3.138917 -5.525899
C -2.708357 2.230162 -5.532117
C -3.118511 1.612519 -4.347167
C -2.411551 1.875168 -3.100754
C -1.315560 2.750862 -3.095211
C -0.119846 2.411698 -2.332177
C -0.070992 1.218596 -1.607971
C -1.213409 0.314094 -1.605906
C -2.364130 0.638590 -2.340564
C -3.508507 0.208004 -4.357068
C -3.037087 -0.394658 -3.116583
C 1.046398 2.856963 -3.083089
C 2.217662 2.082191 -3.078419
C 2.271023 0.846309 -2.320430
C 1.147458 0.419098 -1.597218
C 0.758947 -0.980218 -1.609169
C -0.699539 -1.045401 -1.616212
C -1.349932 -2.040162 -2.353740
C -2.546702 -1.704327 -3.122082
C -2.668711 1.470702 -6.774258
C -3.041725 0.123672 -6.783976
C -3.471739 -0.520415 -5.549150
C -2.959059 -1.884876 -5.554369
C -2.504581 -2.460990 -4.362908
C 2.670468 -1.470892 -3.097717
C 3.042072 -0.123667 -3.088548
C 3.470845 0.518869 -4.324225
C 2.956514 1.883370 -4.318288
C 2.503811 2.462336 -5.507771
C 2.544764 1.703866 -6.749238
C 3.037134 0.394946 -6.755428
C 3.510235 -0.209368 -5.516515
C 2.709133 -2.228772 -4.339219
C 3.121388 -1.613608 -5.525898
C 1.212434 -0.314280 -8.266705
C 2.359289 -0.636950 -7.528887
C 2.411233 -1.876348 -6.769198
C 0.827831 -2.945477 -3.106014
C -0.577881 -3.008496 -3.113190
C -1.284760 -3.262873 -4.357894
C -1.146917 -0.419530 -8.276959
C -2.266819 -0.844286 -7.549525
C -2.217078 -2.083315 -6.790966
C -1.045577 -2.855025 -6.785427
C -0.573605 -3.453586 -5.548375
C 1.565987 -3.136157 -4.344457
C 0.885546 -3.388788 -5.540856
C 1.314296 -2.749024 -6.773948
C 0.119887 -2.411300 -7.538997
C 0.070919 -1.220842 -8.269933
C -0.699539 -1.045401 1.616212

C -1.349932 -2.040162 2.353740
C -0.577881 -3.008496 3.113190
C 0.827831 -2.945477 3.106014
C 0.758947 -0.980218 1.609169
C 1.565987 -3.136157 4.344457
C 0.885546 -3.388788 5.540856
C -0.573605 -3.453586 5.548375
C -1.284760 -3.262873 4.357894
C 1.314296 -2.749024 6.773948
C 2.411233 -1.876348 6.769198
C 3.121388 -1.613608 5.525898
C 2.709133 -2.228772 4.339219
C 2.670468 -1.470892 3.097717
C 3.042072 -0.123667 3.088548
C 3.470845 0.518869 4.324225
C 3.510235 -0.209368 5.516515
C 2.271023 0.846309 2.320430
C 2.217662 2.082191 3.078419
C 2.956514 1.883370 4.318288
C 2.503811 2.462336 5.507771
C 1.285644 3.265622 5.511819
C 0.573847 3.452234 4.320452
C 1.046398 2.856963 3.083089
C -0.119846 2.411698 2.332177
C -0.070992 1.218596 1.607971
C 1.147458 0.419098 1.597218
C 2.544764 1.703866 6.749238
C 3.037134 0.394946 6.755428
C 2.359289 -0.636950 7.528887
C -1.045577 -2.855025 6.785427
C 0.119887 -2.411300 7.538997
C 0.070919 -1.220842 8.269933
C 1.212434 -0.314280 8.266705
C -2.217078 -2.083315 6.790966
C -2.959059 -1.884876 5.554369
C -2.504581 -2.460990 4.362908
C -2.546702 -1.704327 3.122082
C -2.266819 -0.844286 7.549525
C -1.146917 -0.419530 8.276959
C 1.349338 2.038321 7.516632
C 0.699441 1.046779 8.259285
C -0.758947 0.981496 8.266732
C -2.668711 1.470702 6.774258
C -3.041725 0.123672 6.783976
C -3.471739 -0.520415 5.549150
C -3.508507 0.208004 4.357068
C -3.037087 -0.394658 3.116583
C -0.826733 2.945140 6.761565
C 0.576836 3.007623 6.754163
C -1.567129 3.138917 5.525899
C -2.708357 2.230162 5.532117
C -3.118511 1.612519 4.347167
C -2.411551 1.875168 3.100754
C -2.364130 0.638590 2.340564
C -1.213409 0.314094 1.605906
C -1.315560 2.750862 3.095211
C -0.886038 3.387731 4.328169

Benchmarking calculations: BP86/def2-TZVP

C60
E = -2286.744758
C 0.000000 -1.237384 3.333284
C -0.000000 -2.428004 2.597440
C 1.176822 -2.810377 1.832694
C 2.309169 -0.750295 2.597440
C 1.176822 -0.382373 3.333284
C 0.727316 1.001065 3.333284
C -0.727316 1.001065 3.333284
C -1.176822 -0.382373 3.333284
C -0.000000 1.237384 -3.333284
C 0.000000 2.428004 -2.597440
C -1.176822 2.810377 -1.832694
C -1.176822 0.382373 -3.333284
C -2.309169 1.987679 -1.832694
C -0.727316 -1.001065 -3.333284
C 0.727316 -1.001065 -3.333284
C 1.176822 0.382373 -3.333284
C -1.176822 -2.810377 1.832694
C 3.036486 -1.751360 0.595310
C 2.309169 -1.987679 1.832694
C 2.603968 1.581924 1.832694
C -0.699829 2.965362 1.832694
C -3.036486 0.250770 1.832694
C 1.176822 2.810377 -1.832694
C -3.036486 1.751360 -0.595310
C -2.309169 -1.987679 1.832694
C -2.603968 -2.346670 -0.595310
C 1.427145 -3.201681 -0.595310
C 3.485992 0.367922 -0.595310
C 0.727316 3.429069 -0.595310
C 2.603968 2.346670 0.595310
C 0.699829 -2.965362 -1.832694
C -1.427145 3.201681 0.595310
C -3.485992 -0.367922 0.595310
C -0.727316 -3.429069 0.595310
C 3.036486 -0.250770 -1.832694
C -2.603968 -1.581924 -1.832694
C 0.727316 -3.429069 0.595310
C 2.309169 0.750295 -2.597440
C -2.309169 -0.750295 2.597440
C 1.427145 1.964297 2.597440
C -1.427145 1.964297 2.597440
C -1.427145 -3.201681 -0.595310
C 1.427145 -1.964297 -2.597440
C 1.427145 3.201681 0.595310
C -2.603968 2.346670 0.595310
C -3.036486 -1.751360 0.595310
C 3.485992 -0.367922 0.595310
C 2.603968 -2.346670 -0.595310
C 3.036486 1.751360 -0.595310
C -0.727316 3.429069 -0.595310
C -3.485992 0.367922 -0.595310
C 2.603968 -1.581924 -1.832694
C -1.427145 -1.964297 -2.597440
C 2.309169 1.987679 -1.832694
C -2.309169 0.750295 -2.597440
C 3.036486 0.250770 1.832694
C 0.699829 2.965362 1.832694
C -2.603968 1.581924 1.832694
C -3.036486 -0.250770 -1.832694
C -0.699829 -2.965362 -1.832694

C60-
E = -2286.853818
C -0.276663 -3.358408 1.115023
C -0.131601 -1.614700 -3.153948
C 0.338824 -2.648079 -2.333788
C 1.686406 -2.577778 -1.777872
C 2.505260 -1.471501 -2.065359
C 2.018689 -0.404265 -2.916063
C 0.716329 -0.474755 -3.456053
C -0.534810 -3.254810 -1.344319
C 0.263079 -3.534799 -0.167537
C 1.638812 -3.122079 -0.437041
C -1.863024 -2.795863 -1.204407
C -2.421708 -2.608803 0.121892
C -1.650659 -2.884949 1.261303
C 0.522310 -2.774675 2.174412
C -0.346717 -1.921879 2.962935
C -1.694306 -1.996921 2.405690
C -3.266646 -1.422694 0.095854
C -3.308484 -0.564411 1.201230
C -2.507933 -0.853324 2.382782
C -2.018689 0.404265 2.916063
C -0.716329 0.474755 3.456053
C 0.125920 -0.705237 3.474621
C 1.501872 -0.291581 3.207909
C 2.341144 -1.114617 2.438640
C 1.852856 -2.373891 1.914020
C 2.410414 -2.551353 0.587589
C -3.219378 -0.874301 -1.250459
C -2.349488 -1.725526 -2.050266
C -1.505667 -1.142520 -3.012722
C -1.501872 0.291581 -3.207909
C -2.341144 1.114617 -2.438640
C -3.216067 0.512962 -1.439832
C -3.304998 0.876716 1.004004
C -3.259679 1.404006 -0.292340
C -2.505260 1.471501 2.065359
C -2.410414 2.551353 -0.587589
C -1.638812 3.122079 0.437041
C -1.686406 2.577778 1.777872
C 0.131601 1.614700 3.153948
C -0.338824 2.648079 2.333788
C 1.505667 1.142520 3.012722
C 0.534810 3.254810 1.344319
C 1.863024 2.795863 1.204407
C 2.349488 1.725526 2.050266
C 3.216067 -0.512962 1.439832
C 3.219378 0.874301 1.250459
C 3.259679 -1.404006 0.292340
C 3.304998 -0.876716 -1.004004
C 3.266646 1.422694 -0.095854
C 3.308484 0.564411 -1.201230
C 2.507933 0.853324 -2.382782
C 1.694306 1.996921 -2.405690
C 1.650659 2.884949 -1.261303
C 2.421708 2.608803 -0.121892
C 0.346717 1.921879 -2.962935
C -0.522310 2.774675 -2.174412
C 0.276663 3.358408 -1.115023
C -0.263079 3.534799 0.167537
C -1.852856 2.373891 -1.914020
C -0.125920 0.705237 -3.474621

C120-, B1
E = -4574.083099
C -2.569832 2.311873 4.298417
C -2.583158 1.573079 5.538442
C -3.014432 0.245784 5.538436
C -3.438102 -0.359800 4.298451
C -3.438101 0.359800 -4.298451
C -2.992998 1.729865 -4.298460
C -1.424512 1.960743 6.321034
C -2.305164 -0.748978 6.321179
C -2.992998 -1.729865 4.298460
C -3.014432 -0.245784 -5.538436
C -2.294497 1.970732 -5.538567
C -1.404554 3.158776 4.298522
C -2.583158 -1.573079 -5.538442
C -2.569832 -2.311873 -4.298416
C -1.424512 -1.960743 -6.321034
C -1.404554 -3.158776 -4.298522
C -0.726927 1.000477 7.071192
C -2.294497 -1.970732 5.538567
C -0.697825 -2.943038 -5.538546
C -1.165281 -2.790877 5.538418
C -1.176240 -0.382245 7.071246
C -2.305164 0.748978 -6.321179
C -1.165281 2.790877 -5.538418
C 0.726927 1.000476 7.071192
C -0.720229 3.380709 -4.298410
C -0.697825 2.943038 5.538546
C 0.000000 2.423637 -6.321039
C 0.720229 3.380709 -4.298410
C 0.697825 2.943038 5.538546
C -0.720229 -3.380709 4.298410
C -1.176240 0.382245 -7.071246
C 0.000000 -1.236904 7.071301
C 1.165281 2.790877 -5.538418
C 1.404554 3.158776 4.298522
C 1.424512 1.960743 6.321034
C 2.583158 1.573079 5.538442

C 1.176240 -0.382245 7.071246
C 0.000000 -2.423637 6.321039
C 1.165281 -2.790877 5.538418
C 2.569832 2.311873 4.298416
C 2.992998 1.729865 -4.298460
C 3.438102 0.359800 -4.298451
C 2.294497 1.970732 -5.538567
C -0.726927 -1.000476 -7.071192
C 2.294497 -1.970732 5.538567
C 0.000000 1.236904 -7.071301
C 1.176240 0.382245 -7.071246
C 2.305164 -0.748978 6.321179
C 0.720229 -3.380709 4.298410
C 1.404554 -3.158776 -4.298522
C 3.014432 0.245784 5.538436
C 3.438101 -0.359800 4.298451
C 2.305164 0.748978 -6.321179
C 3.014432 -0.245784 -5.538436
C 2.992998 -1.729865 4.298460
C 0.726927 -1.000477 -7.071192
C 0.697825 -2.943038 -5.538546
C 2.569832 -2.311873 -4.298417
C 1.424512 -1.960743 -6.321034
C 2.583158 -1.573079 -5.538442
C 1.429386 3.202037 -3.089054
C 0.716833 3.388515 -1.852374
C -0.716834 3.388515 -1.852374
C -1.429386 3.202037 -3.089054
C 2.603438 2.348922 -3.089071
C -2.603439 2.348923 -3.089071
C 0.725647 3.431004 3.089150
C -0.725647 3.431004 3.089150
C 3.037992 1.750038 3.089032
C 2.571222 2.319807 1.852375
C 1.411598 3.162778 1.852414
C -1.411599 3.162779 1.852414
C -2.571223 2.319808 1.852375
C -3.037992 1.750039 3.089032
C 3.486706 0.369890 3.089049
C 2.603439 -2.348923 3.089071
C 3.000775 -1.728811 1.852360
C 3.443811 -0.365296 1.852355
C 3.037992 -1.750039 -3.089032
C 3.486706 -0.369891 -3.089049
C 3.443811 0.365296 -1.852355
C 3.000775 1.728811 -1.852360
C 1.429386 -3.202037 3.089054
C 0.725647 -3.431004 -3.089150
C 1.411599 -3.162779 -1.852414
C 2.571223 -2.319808 -1.852375
C -0.725647 -3.431004 -3.089150
C -3.037992 -1.750038 -3.089032
C -2.571222 -2.319807 -1.852375
C -1.411598 -3.162778 -1.852414
C -2.603438 -2.348922 3.089071
C -1.429386 -3.202037 3.089054
C -3.486706 -0.369890 -3.089049
C -3.000775 1.728811 -1.852360
C -3.443811 0.365296 -1.852355
C -3.486706 0.369891 3.089049
C -0.716833 -3.388515 1.852374
C 0.716834 -3.388515 1.852374
C -1.400332 3.148968 -0.615859
C -2.561742 2.304884 -0.615868
C 2.561741 2.304883 -0.615868
C 1.400332 3.148967 -0.615859
C 2.983381 -1.724240 -0.615883
C 3.427142 -0.358764 -0.615878
C -0.717876 -3.370830 -0.615891
C 0.717876 -3.370831 -0.615891
C -3.427142 -0.358763 -0.615878
C -2.983380 -1.724239 -0.615883
C -3.443811 -0.365296 1.852355
C -3.000775 -1.728811 1.852360
C 0.717876 3.370830 0.615891
C -0.717876 3.370831 0.615891
C 3.427142 0.358763 0.615878
C 2.983380 1.724239 0.615883
C 1.400332 -3.148968 0.615859
C 2.561742 -2.304884 0.615868
C -2.561741 -2.304883 0.615868
C -1.400332 -3.148967 0.615859
C -2.983381 1.724240 0.615883
C -3.427142 0.358764 0.615878

C120-, C1
E = -4573.626721
C -7.569340 1.430464 1.178748
C -7.133477 0.727387 2.314950
C -7.133477 -0.727387 2.314950
C -7.569340 -1.430464 1.178748
C -8.010659 -0.700135 0.000000
C -8.010659 0.700135 0.000000
C -5.958426 -1.177739 3.035752
C -5.259613 -2.314674 2.606543
C -5.713204 -3.044938 1.428142
C -6.846465 -2.604866 0.729596
C -6.846465 -2.604866 -0.729596
C -5.713204 -3.044938 -1.428142
C -4.541668 -3.500983 -0.703094
C -4.541668 -3.500983 0.703094
C -3.369449 -3.044904 1.429007
C -2.240173 -2.588661 0.728368
C -2.240173 -2.588661 -0.728368
C -3.369449 -3.044904 -1.429007
C -5.259613 -2.314674 -2.606543
C -3.808864 -2.318322 -2.601571
C -3.808864 -2.318322 2.601571
C -3.099013 -1.177369 3.012433
C -1.922619 -0.738472 2.290281
C -1.489379 -1.452424 1.181354
C -0.794153 -0.798038 0.000000
C -1.489379 -1.452424 -1.181354
C -1.922619 -0.738472 -2.290281
C -3.099013 -1.177369 -3.012433
C -7.569340 -1.430464 -1.178748
C -7.133477 -0.727387 -2.314950
C -5.958426 -1.177739 -3.035752
C -5.225369 0.000000 -3.478095
C -3.826595 0.000000 -3.458290
C -0.794153 0.798038 0.000000
C -1.489379 1.452424 1.181354
C -1.922619 0.738472 2.290281
C -3.099013 1.177369 3.012433
C -3.826595 -0.000000 3.458290
C -5.225369 -0.000000 3.478095
C -5.958426 1.177739 3.035752
C -5.259613 2.314674 2.606543
C -3.808864 2.318322 2.601571
C -2.240173 2.588661 0.728368
C -3.369449 3.044904 1.429007
C -6.846465 2.604866 0.729596
C -5.713204 3.044938 1.428142
C -4.541668 3.500983 0.703094
C -1.489379 1.452424 -1.181354
C -1.922619 0.738472 -2.290281
C -3.099013 1.177369 -3.012433
C -7.569340 1.430464 -1.178748
C -7.133477 0.727387 -2.314950
C -5.958426 1.177739 -3.035752
C -5.259613 2.314674 -2.606543
C -3.808864 2.318322 -2.601571
C -2.240173 2.588661 -0.728368
C -3.369449 3.044904 -1.429007
C -4.541668 3.500983 -0.703094
C -5.713204 3.044938 -1.428142
C -6.846465 2.604866 -0.729596
C 1.489379 -1.452424 -1.181354
C 1.922619 -0.738472 -2.290281
C 1.922619 0.738472 -2.290281
C 1.489379 1.452424 -1.181354
C 0.794153 0.798038 0.000000
C 0.794153 -0.798038 0.000000
C 2.240173 2.588661 -0.728368
C 3.369449 3.044904 -1.429007
C 3.808864 2.318322 -2.601571
C 3.099013 1.177369 -3.012433
C 4.541668 3.500983 -0.703094
C 4.541668 3.500983 0.703094
C 3.369449 3.044904 1.429007
C 2.240173 2.588661 0.728368
C 1.489379 1.452424 1.181354
C 1.922619 0.738472 2.290281
C 3.099013 1.177369 3.012433
C 3.808864 2.318322 2.601571
C 1.922619 -0.738472 2.290281
C 3.099013 -1.177369 3.012433
C 3.826595 0.000000 3.458290
C 5.225369 0.000000 3.478095
C 5.958426 -1.177739 3.035752
C 5.259613 -2.314674 2.606543
C 3.808864 -2.318322 2.601571
C 3.369449 -3.044904 1.429007
C 2.240173 -2.588661 0.728368
C 1.489379 -1.452424 1.181354
C 5.958426 1.177739 3.035752
C 5.259613 2.314674 2.606543
C 5.713204 3.044938 1.428142
C 5.259613 2.314674 -2.606543
C 5.713204 3.044938 -1.428142
C 6.846465 2.604866 -0.729596
C 6.846465 2.604866 0.729596
C 5.958426 1.177739 -3.035752
C 5.225369 -0.000000 -3.478095
C 3.826595 -0.000000 -3.458290
C 3.099013 -1.177369 -3.012433
C 7.133477 0.727387 -2.314950
C 7.569340 1.430464 -1.178748
C 7.133477 0.727387 2.314950
C 7.569340 1.430464 1.178748
C 8.010659 0.700135 0.000000
C 8.010659 -0.700135 0.000000
C 7.569340 -1.430464 -1.178748
C 7.133477 -0.727387 -2.314950
C 5.958426 -1.177739 -3.035752
C 5.259613 -2.314674 -2.606543
C 3.808864 -2.318322 -2.601571
C 7.569340 -1.430464 1.178748
C 7.133477 -0.727387 2.314950
C 6.846465 -2.604866 0.729596
C 6.846465 -2.604866 -0.729596
C 5.713204 -3.044938 -1.428142
C 4.541668 -3.500983 -0.703094
C 3.369449 -3.044904 -1.429007
C 2.240173 -2.588661 -0.728368
C 4.541668 -3.500983 0.703094
C 5.713204 -3.044938 1.428142

C120-, P1
E = -4573.644293
C 7.403092 1.177220 1.428960
C 6.981216 2.316162 0.729077
C 6.981216 2.316162 -0.729077
C 7.403092 1.177220 -1.428960
C 7.850215 -0.000000 -0.700612
C 7.850215 -0.000000 0.700612
C 5.814824 3.058587 -1.176547
C 5.094360 2.613815 -2.299872
C 5.522550 1.426492 -3.010029
C 6.667715 0.725814 -2.598630
C 6.667715 -0.725814 -2.598630
C 5.522550 -1.426492 -3.010029
C 4.325109 -0.693854 -3.390656
C 4.325109 0.693854 -3.390656
C 3.159605 1.413566 -2.909453

C 2.001107 0.740017 -2.426353
C 2.001107 -0.740017 -2.426353
C 3.159605 -1.413566 -2.909453
C 5.094360 -2.613815 -2.299872
C 3.640029 -2.619852 -2.278733
C 3.640029 2.619852 -2.278733
C 2.963507 3.128927 -1.163321
C 1.740745 2.510337 -0.744931
C 1.214167 1.412824 -1.424194
C 0.000000 0.771213 -0.813098
C 1.214167 -1.412824 -1.424194
C 1.740745 -2.510337 -0.744931
C 2.963507 -3.128927 -1.163321
C 7.403092 -1.177220 -1.428960
C 6.981216 -2.316162 -0.729077
C 5.814824 -3.058587 -1.176547
C 5.107504 -3.533422 0.000000
C 3.706181 -3.569131 0.000000
C -0.000000 0.771213 0.813098
C 1.214167 1.412824 1.424194
C 1.740745 2.510337 0.744931
C 2.963507 3.128927 1.163321
C 3.706181 3.569131 0.000000
C 5.107504 3.533422 0.000000
C 5.814824 3.058587 1.176547
C 5.094360 2.613815 2.299872
C 3.640029 2.619852 2.278733
C 2.001107 0.740017 2.426353
C 3.159605 1.413566 2.909453
C 6.667715 0.725814 2.598630
C 5.522550 1.426492 3.010029
C 4.325109 0.693854 3.390656
C 1.214167 -1.412824 1.424194
C 1.740745 -2.510337 0.744931
C 2.963507 -3.128927 1.163321
C 7.403092 -1.177220 1.428960
C 6.981216 -2.316162 0.729077
C 5.814824 -3.058587 1.176547
C 5.094360 -2.613815 2.299872
C 3.640029 -2.619852 2.278733
C 2.001107 -0.740017 2.426353
C 3.159605 -1.413566 2.909453
C 4.325109 -0.693854 3.390656
C 5.522550 -1.426492 3.010029
C 6.667715 -0.725814 2.598630
C -1.214167 -1.412824 -1.424194
C -1.740745 -2.510337 -0.744931
C -1.740745 -2.510337 0.744931
C -1.214167 -1.412824 1.424194
C -0.000000 -0.771213 0.813098
C -0.000000 -0.771213 -0.813098
C -2.001107 -0.740017 2.426353
C -3.159605 -1.413566 2.909453
C -3.640029 -2.619852 2.278733
C -2.963507 -3.128927 1.163321
C -4.325109 -0.693854 3.390656
C -4.325109 0.693854 3.390656
C -3.159605 1.413566 2.909453
C -2.001107 0.740017 2.426353
C -1.214167 1.412824 1.424194
C -1.740745 2.510337 0.744931
C -2.963507 3.128927 1.163321
C -3.640029 2.619852 2.278733
C -1.740745 2.510337 -0.744931
C -2.963507 3.128927 -1.163321
C -3.706181 3.569131 0.000000
C -5.107504 3.533422 0.000000
C -5.814824 3.058587 -1.176547
C -5.094360 2.613815 -2.299872
C -3.640029 2.619852 -2.278733
C -3.159605 1.413566 -2.909453
C -2.001107 0.740017 -2.426353
C -1.214167 1.412824 -1.424194
C -5.814824 3.058587 1.176547
C -5.094360 2.613815 2.299872
C -5.522550 1.426492 3.010029
C -5.094360 -2.613815 2.299872
C -5.522550 -1.426492 3.010029
C -6.667715 -0.725814 2.598630
C -6.667715 0.725814 2.598630
C -5.814824 -3.058587 1.176547
C -5.107504 -3.533422 -0.000000
C -3.706181 -3.569131 -0.000000
C -2.963507 -3.128927 -1.163321
C -6.981216 -2.316162 0.729077
C -7.403092 -1.177220 1.428960
C -6.981216 2.316162 0.729077
C -7.403092 1.177220 1.428960
C -7.850215 -0.000000 0.700612
C -7.850215 0.000000 -0.700612
C -7.403092 -1.177220 -1.428960
C -6.981216 -2.316162 -0.729077
C -5.814824 -3.058587 -1.176547
C -5.094360 -2.613815 -2.299872
C -3.640029 -2.619852 -2.278733
C -7.403092 1.177220 -1.428960
C -6.981216 2.316162 -0.729077
C -6.667715 0.725814 -2.598630
C -6.667715 -0.725814 -2.598630
C -5.522550 -1.426492 -3.010029
C -4.325109 -0.693854 -3.390656
C -3.159605 -1.413566 -2.909453
C -2.001107 -0.740017 -2.426353
C -4.325109 0.693854 -3.390656
C -5.522550 1.426492 -3.010029

C120-, V1
E = -4573.627357
C 0.000000 2.362520 2.446068
C -0.000000 -2.362520 2.446068
C 0.000000 7.511181 -2.444645
C -0.000000 -7.511181 -2.444645
C 1.178988 -8.267890 -0.406203
C 2.315794 -7.529638 -0.772247
C 2.316375 -6.757483 -2.001491
C 1.179325 -6.747882 -2.825988
C 0.000000 -8.256309 -1.259683
C 3.041376 -5.521286 -1.756156
C 2.607355 -4.324091 -2.340123
C 1.426933 -4.315083 -3.195304
C 0.729026 -5.507678 -3.430350
C -0.729026 -5.507678 -3.430350
C -1.426933 -4.315083 -3.195304
C -0.703984 -3.078956 -2.956623
C 0.703984 -3.078956 -2.956623
C 1.428045 -2.327491 -1.947915
C 0.728691 -1.604560 -0.973533
C -0.728691 -1.604560 -0.973533
C -1.428045 -2.327491 -1.947915
C -2.607355 -4.324091 -2.340123
C -2.608744 -3.093659 -1.568284
C 2.608744 -3.093659 -1.568284
C 3.041513 -3.101550 -0.236190
C 2.318220 -2.343624 0.773172
C 1.179132 -1.609831 0.406206
C 0.000000 -1.623273 1.258938
C -1.179132 -1.609831 0.406206
C -2.318220 -2.343624 0.773172
C -3.041513 -3.101550 -0.236190
C -1.179325 -6.747882 -2.825988
C -2.316375 -6.757483 -2.001491
C -3.041376 -5.521286 -1.756156
C -3.491774 -5.530241 -0.372721
C -3.490943 -4.344414 0.371839
C 1.179928 -3.125089 2.827260
C 2.316644 -3.115638 2.001408
C 3.039838 -4.353211 1.755629
C 3.490943 -4.344414 0.371839
C 3.491774 -5.530241 -0.372721
C 3.040862 -6.772332 0.236020
C 2.607358 -6.780199 1.567820
C 2.606801 -5.549718 2.340839
C 0.729469 -4.365368 3.429896
C 1.427537 -5.558427 3.196298
C 0.728882 -8.274850 0.973989
C 1.427160 -7.546235 1.946368
C 0.703580 -6.793664 2.956046
C -1.179928 -3.125089 2.827260
C -2.316644 -3.115638 2.001408
C -3.039838 -4.353211 1.755629
C -1.178988 -8.267890 -0.406203
C -2.315794 -7.529638 -0.772247
C -3.040862 -6.772332 0.236020
C -2.607358 -6.780199 1.567820
C -2.606801 -5.549718 2.340839
C -0.729469 -4.365368 3.429896
C -1.427537 -5.558427 3.196298
C -0.703580 -6.793664 2.956046
C -1.427160 -7.546235 1.946368
C -0.728882 -8.274850 0.973989
C -1.179132 1.609831 0.406206
C -2.318220 2.343624 0.773172
C -2.316644 3.115638 2.001408
C -1.179928 3.125089 2.827260
C 0.000000 1.623273 1.258938
C -0.729469 4.365368 3.429896
C -1.427537 5.558427 3.196298
C -2.606801 5.549718 2.340839
C -3.039838 4.353211 1.755629
C -0.703580 6.793664 2.956046
C 0.703580 6.793664 2.956046
C 1.427537 5.558427 3.196298
C 0.729469 4.365368 3.429896
C 1.179928 3.125089 2.827260
C 2.316644 3.115638 2.001408
C 3.039838 4.353211 1.755629
C 2.606801 5.549718 2.340839
C 2.318220 2.343624 0.773172
C 3.041513 3.101550 -0.236190
C 3.490943 4.344414 0.371839
C 3.491774 5.530241 -0.372721
C 3.041376 5.521286 -1.756156
C 2.607355 4.324091 -2.340123
C 2.608744 3.093659 -1.568284
C 1.428045 2.327491 -1.947915
C 0.728691 1.604560 -0.973533
C 1.179132 1.609831 0.406206
C 3.040862 6.772332 0.236020
C 2.607358 6.780199 1.567820
C 1.427160 7.546235 1.946368
C -2.607358 6.780199 1.567820
C -1.427160 7.546235 1.946368
C -0.728882 8.274850 0.973989
C 0.728882 8.274850 0.973989
C -3.040862 6.772332 0.236020
C -3.491774 5.530241 -0.372721
C -3.490943 4.344414 0.371839
C -3.041513 3.101550 -0.236190
C -2.315794 7.529638 -0.772247
C -1.178988 8.267890 -0.406203
C 2.315794 7.529638 -0.772247
C 1.178988 8.267890 -0.406203
C 0.000000 8.256309 -1.259683
C -1.179325 6.747882 -2.825988
C -2.316375 6.757483 -2.001491
C -3.041376 5.521286 -1.756156
C -2.607355 4.324091 -2.340123
C -2.608744 3.093659 -1.568284
C 1.179325 6.747882 -2.825988
C 2.316375 6.757483 -2.001491
C 0.729026 5.507678 -3.430350
C -0.729026 5.507678 -3.430350
C -1.426933 4.315083 -3.195304
C -0.703984 3.078956 -2.956623
C -1.428045 2.327491 -1.947915

C -0.728691 1.604560 -0.973533
C 0.703984 3.078956 -2.956623
C 1.426933 4.315083 -3.195304

Benchmarking calculations: M06L/def2-SVP

C60
E = -2284.054996
C 0.000000 -1.230808 3.320598
C -0.000000 -2.419599 2.585885
C 1.170568 -2.799940 1.825204
C 2.301175 -0.747697 2.585885
C 1.170568 -0.380341 3.320598
C 0.723451 0.995745 3.320598
C -0.723451 0.995745 3.320598
C -1.170568 -0.380341 3.320598
C -0.000000 1.230808 -3.320598
C 0.000000 2.419599 -2.585885
C -1.170568 2.799940 -1.825204
C -1.170568 0.380341 -3.320598
C -2.301175 1.978506 -1.825204
C -0.723451 -0.995745 -3.320598
C 0.723451 -0.995745 -3.320598
C 1.170568 0.380341 -3.320598
C -1.170568 -2.799940 1.825204
C 3.024626 -1.743442 0.594395
C 2.301175 -1.978506 1.825204
C 2.592773 1.577156 1.825204
C -0.698754 2.953242 1.825204
C -3.024626 0.248048 1.825204
C 1.170568 2.799940 -1.825204
C -3.024626 1.743442 -0.594395
C -2.301175 -1.978506 1.825204
C -2.592773 -2.337837 -0.594395
C 1.422205 -3.188305 -0.594395
C 3.471744 0.367357 -0.594395
C 0.723451 3.415344 -0.594395
C 2.592773 2.337837 0.594395
C 0.698754 -2.953242 -1.825204
C -1.422205 3.188305 0.594395
C -3.471744 -0.367357 0.594395
C -0.723451 -3.415344 0.594395
C 3.024626 -0.248048 -1.825204
C -2.592773 -1.577156 -1.825204
C 0.723451 -3.415344 0.594395
C 2.301175 0.747697 -2.585885
C -2.301175 -0.747697 2.585885
C 1.422205 1.957497 2.585885
C -1.422205 1.957497 2.585885
C -1.422205 -3.188305 -0.594395
C 1.422205 -1.957497 -2.585885
C 1.422205 3.188305 0.594395
C -2.592773 2.337837 0.594395
C -3.024626 -1.743442 0.594395
C 3.471744 -0.367357 0.594395
C 2.592773 -2.337837 -0.594395
C 3.024626 1.743442 -0.594395
C -0.723451 3.415344 -0.594395
C -3.471744 0.367357 -0.594395
C 2.592773 -1.577156 -1.825204
C -1.422205 -1.957497 -2.585885
C 2.301175 1.978506 -1.825204
C -2.301175 0.747697 -2.585885
C 3.024626 0.248048 1.825204
C 0.698754 2.953242 1.825204
C -2.592773 1.577156 1.825204
C -3.024626 -0.248048 -1.825204
C -0.698754 -2.953242 -1.825204

C60-
E = -2284.155485
C -0.275588 -3.343297 1.111246
C -0.131187 -1.606644 -3.142830
C 0.337475 -2.639558 -2.323348
C 1.678049 -2.567868 -1.769524
C 2.494608 -1.464150 -2.055979
C 2.012416 -0.402334 -2.904549
C 0.711637 -0.474152 -3.443874
C -0.530757 -3.243432 -1.340588
C 0.263884 -3.519744 -0.168571
C 1.632095 -3.111051 -0.435729
C -1.857368 -2.785173 -1.199295
C -2.412666 -2.597958 0.118923
C -1.642452 -2.873296 1.257644
C 0.520395 -2.763760 2.165853
C -0.345276 -1.914371 2.948985
C -1.685860 -1.990404 2.395624
C -3.252378 -1.417149 0.093385
C -3.293989 -0.560211 1.197072
C -2.498342 -0.847428 2.373160
C -2.012416 0.402334 2.904549
C -0.711637 0.474152 3.443874
C 0.127220 -0.700425 3.460178
C 1.495753 -0.290490 3.196727
C 2.334519 -1.111899 2.428945
C 1.848249 -2.364624 1.906456
C 2.403420 -2.541450 0.587000
C -3.206226 -0.871211 -1.245371
C -2.339617 -1.718063 -2.040208
C -1.498124 -1.136625 -3.000453
C -1.495753 0.290490 -3.196727
C -2.334519 1.111899 -2.428945
C -3.203624 0.513703 -1.435512
C -3.291392 0.872983 1.000268
C -3.247074 1.400353 -0.293585
C -2.494608 1.464150 2.055979
C -2.403420 2.541450 -0.587000
C -1.632095 3.111051 0.435729
C -1.678049 2.567868 1.769524
C 0.131187 1.606644 3.142830
C -0.337475 2.639558 2.323348
C 1.498124 1.136625 3.000453
C 0.530757 3.243432 1.340588
C 1.857368 2.785173 1.199295
C 2.339617 1.718063 2.040208
C 3.203624 -0.513703 1.435512
C 3.206226 0.871211 1.245371
C 3.247074 -1.400353 0.293585
C 3.291392 -0.872983 -1.000268
C 3.252378 1.417149 -0.093385
C 3.293989 0.560211 -1.197072
C 2.498342 0.847428 -2.373160
C 1.685860 1.990404 -2.395624
C 1.642452 2.873296 -1.257644
C 2.412666 2.597958 -0.118923
C 0.345276 1.914371 -2.948985
C -0.520395 2.763760 -2.165853
C 0.275588 3.343297 -1.111246
C -0.263884 3.519744 0.168571
C -1.848249 2.364624 -1.906456
C -0.127220 0.700425 -3.460178

C120-, B1
E = -4568.723088
C -2.560050 2.304527 4.288170
C -2.571873 1.568349 5.521670
C -3.002243 0.243140 5.521566
C -3.425576 -0.359373 4.288126
C -3.425576 0.359373 -4.288126
C -2.982738 1.723007 -4.288193
C -1.419418 1.953611 6.299900
C -2.296895 -0.746239 6.300126
C -2.982738 -1.723007 4.288193
C -3.002243 -0.243140 -5.521566
C -2.286427 1.961360 -5.521907
C -1.400256 3.147417 4.288492
C -2.571873 -1.568349 -5.521670
C -2.560050 -2.304527 -4.288170
C -1.419418 -1.953611 -6.299900
C -1.400256 -3.147417 -4.288492
C -0.723061 0.995020 7.048622
C -2.286428 -1.961360 5.521907
C -0.696704 -2.930598 -5.521872
C -1.159002 -2.780158 5.521616
C -1.169830 -0.380255 7.048653
C -2.296895 0.746239 -6.300126
C -1.159002 2.780158 -5.521616
C 0.723061 0.995020 7.048622
C -0.716785 3.368734 -4.288136
C -0.696704 2.930598 5.521872
C 0.000000 2.415113 -6.299881
C 0.716785 3.368734 -4.288136
C 0.696704 2.930598 5.521872
C -0.716785 -3.368734 4.288136
C -1.169830 0.380255 -7.048653
C 0.000000 -1.230502 7.048697
C 1.159002 2.780158 -5.521616
C 1.400256 3.147417 4.288492
C 1.419418 1.953611 6.299900
C 2.571873 1.568349 5.521670
C 1.169830 -0.380255 7.048653
C 0.000000 -2.415113 6.299881
C 1.159002 -2.780158 5.521616
C 2.560050 2.304527 4.288170
C 2.982738 1.723007 -4.288193
C 3.425576 0.359373 -4.288126
C 2.286428 1.961360 -5.521907
C -0.723061 -0.995020 -7.048622
C 2.286427 -1.961360 5.521907
C 0.000000 1.230502 -7.048697
C 1.169830 0.380255 -7.048653
C 2.296895 -0.746239 6.300126
C 0.716785 -3.368734 4.288136
C 1.400256 -3.147417 -4.288492
C 3.002243 0.243140 5.521566
C 3.425576 -0.359373 4.288126
C 2.296895 0.746239 -6.300126
C 3.002243 -0.243140 -5.521566
C 2.982738 -1.723007 4.288193
C 0.723061 -0.995020 -7.048622
C 0.696704 -2.930598 -5.521872
C 2.560050 -2.304527 -4.288170
C 1.419418 -1.953611 -6.299900
C 2.571873 -1.568349 -5.521670
C 1.425598 3.192780 -3.081360
C 0.714986 3.381540 -1.848048
C -0.714986 3.381540 -1.848048
C -1.425598 3.192780 -3.081360
C 2.595376 2.342417 -3.081386
C -2.595376 2.342417 -3.081386
C 0.723113 3.421793 3.081628
C -0.723113 3.421793 3.081628
C 3.028693 1.744499 3.081250
C 2.564698 2.314797 1.848018
C 1.408515 3.156102 1.848150
C -1.408515 3.156102 1.848150
C -2.564698 2.314797 1.848018
C -3.028693 1.744499 3.081250
C 3.475948 0.369111 3.081261
C 2.595376 -2.342417 3.081386
C 2.993650 -1.724796 1.847956
C 3.435250 -0.364648 1.847938
C 3.028693 -1.744499 -3.081250
C 3.475948 -0.369111 -3.081261
C 3.435250 0.364648 -1.847938
C 2.993650 1.724796 -1.847956
C 1.425598 -3.192780 3.081360
C 0.723113 -3.421793 -3.081628
C 1.408515 -3.156102 -1.848150
C 2.564698 -2.314797 -1.848018
C -0.723113 -3.421793 -3.081628
C -3.028693 -1.744499 -3.081250
C -2.564698 -2.314797 -1.848018

C -1.408515 -3.156102 -1.848150
C -2.595376 -2.342417 3.081386
C -1.425598 -3.192780 3.081360
C -3.475948 -0.369111 -3.081261
C -2.993650 1.724796 -1.847956
C -3.435250 0.364648 -1.847938
C -3.475948 0.369111 3.081261
C -0.714986 -3.381540 1.848048
C 0.714986 -3.381540 1.848048
C -1.397787 3.143191 -0.614291
C -2.556003 2.300740 -0.614320
C 2.556003 2.300740 -0.614320
C 1.397787 3.143192 -0.614291
C 2.976962 -1.720595 -0.614348
C 3.419393 -0.358322 -0.614329
C -0.716129 -3.365170 -0.614416
C 0.716129 -3.365170 -0.614416
C -3.419393 -0.358322 -0.614329
C -2.976962 -1.720595 -0.614348
C -3.435250 -0.364648 1.847938
C -2.993650 -1.724796 1.847956
C 0.716129 3.365170 0.614416
C -0.716129 3.365170 0.614416
C 3.419393 0.358322 0.614329
C 2.976962 1.720595 0.614348
C 1.397787 -3.143191 0.614291
C 2.556003 -2.300740 0.614320
C -2.556003 -2.300740 0.614320
C -1.397787 -3.143192 0.614291
C -2.976962 1.720595 0.614348
C -3.419393 0.358322 0.614329

C120-, C1
E = -4568.233987
C -7.538603 1.425649 1.172564
C -7.103575 0.723472 2.306830
C -7.103575 -0.723472 2.306830
C -7.538603 -1.425649 1.172564
C -7.977061 -0.699094 0.000000
C -7.977061 0.699094 0.000000
C -5.934545 -1.171560 3.023971
C -5.236690 -2.306610 2.595315
C -5.687244 -3.032794 1.423308
C -6.818711 -2.593562 0.725623
C -6.818711 -2.593562 -0.725623
C -5.687244 -3.032794 -1.423308
C -4.521937 -3.486693 -0.702026
C -4.521937 -3.486693 0.702026
C -3.356206 -3.033100 1.424873
C -2.228701 -2.577887 0.724760
C -2.228701 -2.577887 -0.724760
C -3.356206 -3.033100 -1.424873
C -5.236690 -2.306610 -2.595315
C -3.793302 -2.310703 -2.591394
C -3.793302 -2.310703 2.591394
C -3.085214 -1.171554 3.002113
C -1.915203 -0.734424 2.284406
C -1.483596 -1.444413 1.173952
C -0.788084 -0.790813 0.000000
C -1.483596 -1.444413 -1.173952
C -1.915203 -0.734424 -2.284406
C -3.085214 -1.171554 -3.002113
C -7.538603 -1.425649 -1.172564
C -7.103575 -0.723472 -2.306830
C -5.934545 -1.171560 -3.023971
C -5.205587 0.000000 -3.463794
C -3.808956 0.000000 -3.444816
C -0.788084 0.790813 0.000000
C -1.483596 1.444413 1.173952
C -1.915203 0.734424 2.284406
C -3.085214 1.171554 3.002113
C -3.808956 -0.000000 3.444816
C -5.205587 -0.000000 3.463794
C -5.934545 1.171560 3.023971
C -5.236690 2.306610 2.595315
C -3.793302 2.310703 2.591394
C -2.228701 2.577887 0.724760
C -3.356206 3.033100 1.424873
C -6.818711 2.593562 0.725623
C -5.687244 3.032794 1.423308
C -4.521937 3.486693 0.702026
C -1.483596 1.444413 -1.173952
C -1.915203 0.734424 -2.284406
C -3.085214 1.171554 -3.002113
C -7.538603 1.425649 -1.172564
C -7.103575 0.723472 -2.306830
C -5.934545 1.171560 -3.023971
C -5.236690 2.306610 -2.595315
C -3.793302 2.310703 -2.591394
C -2.228701 2.577887 -0.724760
C -3.356206 3.033100 -1.424873
C -4.521937 3.486693 -0.702026
C -5.687244 3.032794 -1.423308
C -6.818711 2.593562 -0.725623
C 1.483596 -1.444413 -1.173952
C 1.915203 -0.734424 -2.284406
C 1.915203 0.734424 -2.284406
C 1.483596 1.444413 -1.173952
C 0.788084 0.790813 0.000000
C 0.788084 -0.790813 0.000000
C 2.228701 2.577887 -0.724760
C 3.356206 3.033100 -1.424873
C 3.793302 2.310703 -2.591394
C 3.085214 1.171554 -3.002113
C 4.521937 3.486693 -0.702026
C 4.521937 3.486693 0.702026
C 3.356206 3.033100 1.424873
C 2.228701 2.577887 0.724760
C 1.483596 1.444413 1.173952
C 1.915203 0.734424 2.284406
C 3.085214 1.171554 3.002113
C 3.793302 2.310703 2.591394
C 1.915203 -0.734424 2.284406
C 3.085214 -1.171554 3.002113
C 3.808956 0.000000 3.444816
C 5.205587 0.000000 3.463794
C 5.934545 -1.171560 3.023971
C 5.236690 -2.306610 2.595315
C 3.793302 -2.310703 2.591394
C 3.356206 -3.033100 1.424873
C 2.228701 -2.577887 0.724760
C 1.483596 -1.444413 1.173952
C 5.934545 1.171560 3.023971
C 5.236690 2.306610 2.595315
C 5.687244 3.032794 1.423308
C 5.236690 2.306610 -2.595315
C 5.687244 3.032794 -1.423308
C 6.818711 2.593562 -0.725623
C 6.818711 2.593562 0.725623
C 5.934545 1.171560 -3.023971
C 5.205587 -0.000000 -3.463794
C 3.808956 -0.000000 -3.444816
C 3.085214 -1.171554 -3.002113
C 7.103575 0.723472 -2.306830
C 7.538603 1.425649 -1.172564
C 7.103575 0.723472 2.306830
C 7.538603 1.425649 1.172564
C 7.977061 0.699094 0.000000
C 7.977061 -0.699094 0.000000
C 7.538603 -1.425649 -1.172564
C 7.103575 -0.723472 -2.306830
C 5.934545 -1.171560 -3.023971
C 5.236690 -2.306610 -2.595315
C 3.793302 -2.310703 -2.591394
C 7.538603 -1.425649 1.172564
C 7.103575 -0.723472 2.306830
C 6.818711 -2.593562 0.725623
C 6.818711 -2.593562 -0.725623
C 5.687244 -3.032794 -1.423308
C 4.521937 -3.486693 -0.702026
C 3.356206 -3.033100 -1.424873
C 2.228701 -2.577887 -0.724760
C 4.521937 -3.486693 0.702026
C 5.687244 -3.032794 1.423308

C120-, P1
E = -4568.250296
C 7.374383 1.170926 1.424089
C 6.953057 2.307855 0.725025
C 6.953057 2.307855 -0.725025
C 7.374383 1.170926 -1.424089
C 7.819137 -0.000000 -0.699506
C 7.819137 -0.000000 0.699506
C 5.792670 3.046606 -1.170196
C 5.073169 2.602480 -2.291005
C 5.499374 1.421606 -2.998006
C 6.642858 0.722030 -2.587580
C 6.642858 -0.722030 -2.587580
C 5.499374 -1.421606 -2.998006
C 4.308356 -0.693030 -3.375670
C 4.308356 0.693030 -3.375670
C 3.148236 1.409546 -2.898200
C 1.991263 0.737447 -2.418280
C 1.991263 -0.737447 -2.418280
C 3.148236 -1.409546 -2.898200
C 5.073169 -2.602480 -2.291005
C 3.626374 -2.609511 -2.270266
C 3.626374 2.609511 -2.270266
C 2.951312 3.118991 -1.156929
C 1.732040 2.506090 -0.740569
C 1.208719 1.407518 -1.416804
C 0.000000 0.766307 -0.802447
C 1.208719 -1.407518 -1.416804
C 1.732040 -2.506090 -0.740569
C 2.951312 -3.118991 -1.156929
C 7.374383 -1.170926 -1.424089
C 6.953057 -2.307855 -0.725025
C 5.792670 -3.046606 -1.170196
C 5.089539 -3.519651 0.000000
C 3.690218 -3.556317 0.000000
C -0.000000 0.766307 0.802447
C 1.208719 1.407518 1.416804
C 1.732040 2.506090 0.740569
C 2.951312 3.118991 1.156929
C 3.690218 3.556317 0.000000
C 5.089539 3.519651 0.000000
C 5.792670 3.046606 1.170196
C 5.073169 2.602480 2.291005
C 3.626374 2.609511 2.270266
C 1.991263 0.737447 2.418280
C 3.148236 1.409546 2.898200
C 6.642858 0.722030 2.587580
C 5.499374 1.421606 2.998006
C 4.308356 0.693030 3.375670
C 1.208719 -1.407518 1.416804
C 1.732040 -2.506090 0.740569
C 2.951312 -3.118991 1.156929
C 7.374383 -1.170926 1.424089
C 6.953057 -2.307855 0.725025
C 5.792670 -3.046606 1.170196
C 5.073169 -2.602480 2.291005
C 3.626374 -2.609511 2.270266
C 1.991263 -0.737447 2.418280
C 3.148236 -1.409546 2.898200
C 4.308356 -0.693030 3.375670
C 5.499374 -1.421606 2.998006
C 6.642858 -0.722030 2.587580
C -1.208719 -1.407518 -1.416804
C -1.732040 -2.506090 -0.740569
C -1.732040 -2.506090 0.740569
C -1.208719 -1.407518 1.416804
C -0.000000 -0.766307 0.802447
C -0.000000 -0.766307 -0.802447
C -1.991263 -0.737447 2.418280
C -3.148236 -1.409546 2.898200

C -3.626374 -2.609511 2.270266
C -2.951312 -3.118991 1.156929
C -4.308356 -0.693030 3.375670
C -4.308356 0.693030 3.375670
C -3.148236 1.409546 2.898200
C -1.991263 0.737447 2.418280
C -1.208719 1.407518 1.416804
C -1.732040 2.506090 0.740569
C -2.951312 3.118991 1.156929
C -3.626374 2.609511 2.270266
C -1.732040 2.506090 -0.740569
C -2.951312 3.118991 -1.156929
C -3.690218 3.556317 0.000000
C -5.089539 3.519651 0.000000
C -5.792670 3.046606 -1.170196
C -5.073169 2.602480 -2.291005
C -3.626374 2.609511 -2.270266
C -3.148236 1.409546 -2.898200
C -1.991263 0.737447 -2.418280
C -1.208719 1.407518 -1.416804
C -5.792670 3.046606 1.170196
C -5.073169 2.602480 2.291005
C -5.499374 1.421606 2.998006
C -5.073169 -2.602480 2.291005
C -5.499374 -1.421606 2.998006
C -6.642858 -0.722030 2.587580
C -6.642858 0.722030 2.587580
C -5.792670 -3.046606 1.170196
C -5.089539 -3.519651 -0.000000
C -3.690218 -3.556317 -0.000000
C -2.951312 -3.118991 -1.156929
C -6.953057 -2.307855 0.725025
C -7.374383 -1.170926 1.424089
C -6.953057 2.307855 0.725025
C -7.374383 1.170926 1.424089
C -7.819137 -0.000000 0.699506
C -7.819137 0.000000 -0.699506
C -7.374383 -1.170926 -1.424089
C -6.953057 -2.307855 -0.725025
C -5.792670 -3.046606 -1.170196
C -5.073169 -2.602480 -2.291005
C -3.626374 -2.609511 -2.270266
C -7.374383 1.170926 -1.424089
C -6.953057 2.307855 -0.725025
C -6.642858 0.722030 -2.587580
C -6.642858 -0.722030 -2.587580
C -5.499374 -1.421606 -2.998006
C -4.308356 -0.693030 -3.375670
C -3.148236 -1.409546 -2.898200
C -1.991263 -0.737447 -2.418280
C -4.308356 0.693030 -3.375670
C -5.499374 1.421606 -2.998006

C120-, V1
E = -4568.239255
C 0.000000 2.406121 2.470410
C -0.000000 -2.406121 2.470410
C 0.000000 7.469189 -2.469006
C -0.000000 -7.469189 -2.469006
C 1.172798 -8.250745 -0.446669
C 2.308001 -7.509220 -0.802560
C 2.308595 -6.725706 -2.015422
C 1.173337 -6.705516 -2.838482
C 0.000000 -8.228059 -1.295287
C 3.029508 -5.499066 -1.755536
C 2.595904 -4.296444 -2.323111
C 1.421769 -4.276392 -3.173243
C 0.725071 -5.464006 -3.423538
C -0.725071 -5.464006 -3.423538
C -1.421769 -4.276392 -3.173243
C -0.702763 -3.049782 -2.920703
C 0.702763 -3.049782 -2.920703
C 1.423152 -2.315562 -1.907456
C 0.724818 -1.605753 -0.925619
C -0.724818 -1.605753 -0.925619
C -1.423152 -2.315562 -1.907456
C -2.595904 -4.296444 -2.323111
C -2.597381 -3.082274 -1.539715
C 2.597381 -3.082274 -1.539715
C 3.029632 -3.107103 -0.210085
C 2.310316 -2.365662 0.803439
C 1.173075 -1.627794 0.446766
C 0.000000 -1.651833 1.294795
C -1.173075 -1.627794 0.446766
C -2.310316 -2.365662 0.803439
C -3.029632 -3.107103 -0.210085
C -1.173337 -6.705516 -2.838482
C -2.308595 -6.725706 -2.015422
C -3.029508 -5.499066 -1.755536
C -3.477742 -5.525452 -0.379650
C -3.476657 -4.351028 0.378732
C 1.173804 -3.169372 2.839467
C 2.308577 -3.149418 2.015165
C 3.027751 -4.377290 1.754868
C 3.476657 -4.351028 0.378732
C 3.477742 -5.525452 -0.379650
C 3.029287 -6.768688 0.209923
C 2.596333 -6.793587 1.539447
C 2.595490 -5.579334 2.323889
C 0.725413 -4.411022 3.422752
C 1.422418 -5.599103 3.174413
C 0.724981 -8.275000 0.926046
C 1.422339 -7.560044 1.906048
C 0.702476 -6.824972 2.920377
C -1.173804 -3.169372 2.839467
C -2.308577 -3.149418 2.015165
C -3.027751 -4.377290 1.754868
C -1.172798 -8.250745 -0.446669
C -2.308001 -7.509220 -0.802560
C -3.029287 -6.768688 0.209923
C -2.596333 -6.793587 1.539447
C -2.595490 -5.579334 2.323889
C -0.725413 -4.411022 3.422752
C -1.422418 -5.599103 3.174413
C -0.702476 -6.824972 2.920377
C -1.422339 -7.560044 1.906048
C -0.724981 -8.275000 0.926046
C -1.173075 1.627794 0.446766
C -2.310316 2.365662 0.803439
C -2.308577 3.149418 2.015165
C -1.173804 3.169372 2.839467
C 0.000000 1.651833 1.294795
C -0.725413 4.411022 3.422752
C -1.422418 5.599103 3.174413
C -2.595490 5.579334 2.323889
C -3.027751 4.377290 1.754868
C -0.702476 6.824972 2.920377
C 0.702476 6.824972 2.920377
C 1.422418 5.599103 3.174413
C 0.725413 4.411022 3.422752
C 1.173804 3.169372 2.839467
C 2.308577 3.149418 2.015165
C 3.027751 4.377290 1.754868
C 2.595490 5.579334 2.323889
C 2.310316 2.365662 0.803439
C 3.029632 3.107103 -0.210085
C 3.476657 4.351028 0.378732
C 3.477742 5.525452 -0.379650
C 3.029508 5.499066 -1.755536
C 2.595904 4.296444 -2.323111
C 2.597381 3.082274 -1.539715
C 1.423152 2.315562 -1.907456
C 0.724818 1.605753 -0.925619
C 1.173075 1.627794 0.446766
C 3.029287 6.768688 0.209923
C 2.596333 6.793587 1.539447
C 1.422339 7.560044 1.906048
C -2.596333 6.793587 1.539447
C -1.422339 7.560044 1.906048
C -0.724981 8.275000 0.926046
C 0.724981 8.275000 0.926046
C -3.029287 6.768688 0.209923
C -3.477742 5.525452 -0.379650
C -3.476657 4.351028 0.378732
C -3.029632 3.107103 -0.210085
C -2.308001 7.509220 -0.802560
C -1.172798 8.250745 -0.446669
C 2.308001 7.509220 -0.802560
C 1.172798 8.250745 -0.446669
C 0.000000 8.228059 -1.295287
C -1.173337 6.705516 -2.838482
C -2.308595 6.725706 -2.015422
C -3.029508 5.499066 -1.755536
C -2.595904 4.296444 -2.323111
C -2.597381 3.082274 -1.539715
C 1.173337 6.705516 -2.838482
C 2.308595 6.725706 -2.015422
C 0.725071 5.464006 -3.423538
C -0.725071 5.464006 -3.423538
C -1.421769 4.276392 -3.173243
C -0.702763 3.049782 -2.920703
C -1.423152 2.315562 -1.907456
C -0.724818 1.605753 -0.925619
C 0.702763 3.049782 -2.920703
C 1.421769 4.276392 -3.173243

Benchmarking calculations: PBE/def2-SVP

C60
E = -2281.678052
C 0.000000 -1.240013 3.344502
C -0.000000 -2.436863 2.604808
C 1.179322 -2.820048 1.838438
C 2.317595 -0.753032 2.604808
C 1.179322 -0.383185 3.344502
C 0.728861 1.003192 3.344502
C -0.728861 1.003192 3.344502
C -1.179322 -0.383185 3.344502
C -0.000000 1.240013 -3.344502
C 0.000000 2.436863 -2.604808
C -1.179322 2.820048 -1.838438
C -1.179322 0.383185 -3.344502
C -2.317595 1.993045 -1.838438
C -0.728861 -1.003192 -3.344502
C 0.728861 -1.003192 -3.344502
C 1.179322 0.383185 -3.344502
C -1.179322 -2.820048 1.838438
C 3.046456 -1.756224 0.598425
C 2.317595 -1.993045 1.838438
C 2.611675 1.588279 1.838438
C -0.703491 2.974655 1.838438
C -3.046456 0.250159 1.838438
C 1.179322 2.820048 -1.838438
C -3.046456 1.756224 -0.598425
C -2.317595 -1.993045 1.838438
C -2.611675 -2.354649 -0.598425
C 1.432352 -3.211477 -0.598425
C 3.496917 0.369847 -0.598425
C 0.728861 3.440055 -0.598425
C 2.611675 2.354649 0.598425
C 0.703491 -2.974655 -1.838438
C -1.432352 3.211477 0.598425
C -3.496917 -0.369847 0.598425
C -0.728861 -3.440055 0.598425
C 3.046456 -0.250159 -1.838438
C -2.611675 -1.588279 -1.838438
C 0.728861 -3.440055 0.598425
C 2.317595 0.753032 -2.604808
C -2.317595 -0.753032 2.604808
C 1.432352 1.971464 2.604808
C -1.432352 1.971464 2.604808
C -1.432352 -3.211477 -0.598425
C 1.432352 -1.971464 -2.604808
C 1.432352 3.211477 0.598425

C -2.611675 2.354649 0.598425
C -3.046456 -1.756224 0.598425
C 3.496917 -0.369847 0.598425
C 2.611675 -2.354649 -0.598425
C 3.046456 1.756224 -0.598425
C -0.728861 3.440055 -0.598425
C -3.496917 0.369847 -0.598425
C 2.611675 -1.588279 -1.838438
C -1.432352 -1.971464 -2.604808
C 2.317595 1.993045 -1.838438
C -2.317595 0.753032 -2.604808
C 3.046456 0.250159 1.838438
C 0.703491 2.974655 1.838438
C -2.611675 1.588279 1.838438
C -3.046456 -0.250159 -1.838438
C -0.703491 -2.974655 -1.838438

C60-
E = -2281.781516
C -0.277392 -3.367374 1.118914
C -0.131899 -1.618696 -3.165530
C 0.339848 -2.658400 -2.340424
C 1.690444 -2.586192 -1.782331
C 2.512599 -1.475150 -2.070929
C 2.026382 -0.405246 -2.925653
C 0.717144 -0.477516 -3.468425
C -0.534933 -3.266221 -1.350076
C 0.265545 -3.545106 -0.169425
C 1.644139 -3.133494 -0.438638
C -1.870161 -2.805260 -1.208046
C -2.429756 -2.616844 0.120105
C -1.654513 -2.893892 1.266500
C 0.524550 -2.783212 2.181110
C -0.347634 -1.927953 2.970522
C -1.698253 -2.004339 2.412867
C -3.275721 -1.427207 0.094354
C -3.317884 -0.564461 1.205630
C -2.516464 -0.853930 2.390652
C -2.026382 0.405246 2.925653
C -0.717144 0.477516 3.468425
C 0.127908 -0.705837 3.485076
C 1.506614 -0.292726 3.219409
C 2.351344 -1.119737 2.446831
C 1.861190 -2.381816 1.920416
C 2.420701 -2.560192 0.590980
C -3.229340 -0.877196 -1.254367
C -2.356358 -1.730245 -2.055424
C -1.509091 -1.144884 -3.021911
C -1.506614 0.292726 -3.219409
C -2.351344 1.119737 -2.446831
C -3.226830 0.517135 -1.445805
C -3.315234 0.879462 1.007315
C -3.270496 1.410389 -0.295354
C -2.512599 1.475150 2.070929
C -2.420701 2.560192 -0.590980
C -1.644139 3.133494 0.438638
C -1.690444 2.586192 1.782331
C 0.131899 1.618696 3.165530
C -0.339848 2.658400 2.340424
C 1.509091 1.144884 3.021911
C 0.534933 3.266221 1.350076
C 1.870161 2.805260 1.208046
C 2.356358 1.730245 2.055424
C 3.226830 -0.517135 1.445805
C 3.229340 0.877196 1.254367
C 3.270496 -1.410389 0.295354
C 3.315234 -0.879462 -1.007315
C 3.275721 1.427207 -0.094354
C 3.317884 0.564461 -1.205630
C 2.516464 0.853930 -2.390652
C 1.698253 2.004339 -2.412867
C 1.654513 2.893892 -1.266500
C 2.429756 2.616844 -0.120105
C 0.347634 1.927953 -2.970522
C -0.524550 2.783212 -2.181110
C 0.277392 3.367374 -1.118914
C -0.265545 3.545106 0.169425
C -1.861190 2.381816 -1.920416
C -0.127908 0.705837 -3.485076

C120-, B1
E = -4563.959640
C -2.578169 2.320209 4.314712
C -2.591022 1.579453 5.557935
C -3.024540 0.245241 5.557933
C -3.449765 -0.361607 4.314734
C -3.449765 0.361607 -4.314734
C -3.003491 1.735257 -4.314741
C -1.429700 1.967871 6.342113
C -2.313587 -0.751724 6.342294
C -3.003491 -1.735257 4.314741
C -3.024540 -0.245241 -5.557933
C -2.302960 1.976220 -5.558042
C -1.409871 3.169354 4.314831
C -2.591022 -1.579453 -5.557935
C -2.578168 -2.320209 -4.314712
C -1.429700 -1.967871 -6.342113
C -1.409871 -3.169354 -4.314831
C -0.728515 1.002655 7.095781
C -2.302960 -1.976220 5.558042
C -0.701452 -2.952480 -5.558053
C -1.167895 -2.800684 5.557927
C -1.178810 -0.383091 7.095866
C -2.313587 0.751724 -6.342295
C -1.167895 2.800684 -5.557927
C 0.728515 1.002655 7.095781
C -0.722110 3.392396 -4.314710
C -0.701452 2.952480 5.558053
C 0.000000 2.432520 -6.342154
C 0.722110 3.392396 -4.314710
C 0.701452 2.952480 5.558053
C -0.722110 -3.392396 4.314710
C -1.178810 0.383091 -7.095866
C -0.000000 -1.239614 7.095897
C 1.167895 2.800684 -5.557927
C 1.409871 3.169354 4.314831
C 1.429700 1.967871 6.342113
C 2.591022 1.579453 5.557935
C 1.178810 -0.383091 7.095866
C -0.000000 -2.432520 6.342154
C 1.167895 -2.800684 5.557927
C 2.578168 2.320209 4.314712
C 3.003491 1.735257 -4.314741
C 3.449765 0.361607 -4.314734
C 2.302960 1.976220 -5.558042
C -0.728515 -1.002655 -7.095781
C 2.302960 -1.976220 5.558042
C 0.000000 1.239614 -7.095897
C 1.178810 0.383091 -7.095866
C 2.313587 -0.751724 6.342295
C 0.722110 -3.392396 4.314710
C 1.409871 -3.169354 -4.314831
C 3.024540 0.245241 5.557933
C 3.449765 -0.361607 4.314734
C 2.313587 0.751724 -6.342294
C 3.024540 -0.245241 -5.557933
C 3.003491 -1.735257 4.314741
C 0.728515 -1.002655 -7.095781
C 0.701452 -2.952480 -5.558053
C 2.578169 -2.320209 -4.314712
C 1.429700 -1.967871 -6.342113
C 2.591022 -1.579453 -5.557935
C 1.434607 3.212965 -3.099936
C 0.719706 3.401983 -1.859086
C -0.719706 3.401983 -1.859086
C -1.434607 3.212965 -3.099936
C 2.612146 2.357249 -3.099941
C -2.612146 2.357249 -3.099941
C 0.727841 3.443119 3.100063
C -0.727841 3.443119 3.100063
C 3.048496 1.755788 3.099895
C 2.581208 2.328869 1.859075
C 1.417068 3.175369 1.859128
C -1.417068 3.175369 1.859128
C -2.581208 2.328869 1.859075
C -3.048496 1.755788 3.099895
C 3.498516 0.371500 3.099908
C 2.612146 -2.357249 3.099941
C 3.012441 -1.735657 1.859040
C 3.457183 -0.366681 1.859040
C 3.048496 -1.755788 -3.099895
C 3.498516 -0.371500 -3.099908
C 3.457182 0.366681 -1.859040
C 3.012441 1.735657 -1.859040
C 1.434607 -3.212965 3.099936
C 0.727841 -3.443119 -3.100063
C 1.417068 -3.175369 -1.859128
C 2.581208 -2.328869 -1.859075
C -0.727841 -3.443119 -3.100063
C -3.048496 -1.755788 -3.099895
C -2.581208 -2.328869 -1.859075
C -1.417068 -3.175369 -1.859128
C -2.612146 -2.357249 3.099941
C -1.434607 -3.212965 3.099936
C -3.498516 -0.371500 -3.099908
C -3.012441 1.735657 -1.859040
C -3.457183 0.366681 -1.859040
C -3.498516 0.371500 3.099908
C -0.719706 -3.401983 1.859086
C 0.719706 -3.401983 1.859086
C -1.406468 3.162344 -0.618123
C -2.572318 2.314836 -0.618127
C 2.572317 2.314836 -0.618127
C 1.406468 3.162345 -0.618123
C 2.996007 -1.731348 -0.618152
C 3.441475 -0.360535 -0.618140
C -0.720694 -3.385471 -0.618161
C 0.720694 -3.385471 -0.618161
C -3.441475 -0.360535 -0.618140
C -2.996006 -1.731348 -0.618152
C -3.457182 -0.366681 1.859040
C -3.012441 -1.735657 1.859040
C 0.720694 3.385471 0.618161
C -0.720694 3.385471 0.618161
C 3.441475 0.360535 0.618140
C 2.996006 1.731348 0.618152
C 1.406468 -3.162344 0.618123
C 2.572318 -2.314836 0.618127
C -2.572317 -2.314836 0.618127
C -1.406468 -3.162345 0.618123
C -2.996007 1.731348 0.618152
C -3.441475 0.360535 0.618140

C120-, C1
E = -4563.494731
C -7.590880 1.435553 1.181424
C -7.152933 0.728906 2.323361
C -7.152933 -0.728906 2.323361
C -7.590880 -1.435553 1.181424
C -8.032822 -0.703757 0.000000
C -8.032822 0.703757 0.000000
C -5.975085 -1.180389 3.045835
C -5.272627 -2.323124 2.614217
C -5.726914 -3.054692 1.433326
C -6.865970 -2.612391 0.731025
C -6.865970 -2.612391 -0.731025
C -5.726914 -3.054692 -1.433326
C -4.552872 -3.512177 -0.706657
C -4.552872 -3.512177 0.706657
C -3.378225 -3.054780 1.434552
C -2.244070 -2.595552 0.729859
C -2.244070 -2.595552 -0.729859
C -3.378225 -3.054780 -1.434552
C -5.272627 -2.323124 -2.614217
C -3.818514 -2.327077 -2.610196

C -3.818514 -2.327077 2.610196
C -3.105451 -1.180234 3.023293
C -1.926796 -0.739457 2.299905
C -1.491989 -1.454987 1.182686
C -0.795026 -0.797896 0.000000
C -1.491989 -1.454987 -1.182686
C -1.926796 -0.739457 -2.299905
C -3.105451 -1.180234 -3.023293
C -7.590880 -1.435553 -1.181424
C -7.152933 -0.728906 -2.323361
C -5.975085 -1.180389 -3.045835
C -5.240675 0.000000 -3.488882
C -3.834578 0.000000 -3.469592
C -0.795026 0.797896 0.000000
C -1.491989 1.454987 1.182686
C -1.926796 0.739457 2.299905
C -3.105451 1.180234 3.023293
C -3.834578 -0.000000 3.469592
C -5.240675 -0.000000 3.488882
C -5.975085 1.180389 3.045835
C -5.272627 2.323124 2.614217
C -3.818514 2.327077 2.610196
C -2.244070 2.595552 0.729859
C -3.378225 3.054780 1.434552
C -6.865970 2.612391 0.731025
C -5.726914 3.054692 1.433326
C -4.552872 3.512177 0.706657
C -1.491989 1.454987 -1.182686
C -1.926796 0.739457 -2.299905
C -3.105451 1.180234 -3.023293
C -7.590880 1.435553 -1.181424
C -7.152933 0.728906 -2.323361
C -5.975085 1.180389 -3.045835
C -5.272627 2.323124 -2.614217
C -3.818514 2.327077 -2.610196
C -2.244070 2.595552 -0.729859
C -3.378225 3.054780 -1.434552
C -4.552872 3.512177 -0.706657
C -5.726914 3.054692 -1.433326
C -6.865970 2.612391 -0.731025
C 1.491989 -1.454987 -1.182686
C 1.926796 -0.739457 -2.299905
C 1.926796 0.739457 -2.299905
C 1.491989 1.454987 -1.182686
C 0.795026 0.797896 0.000000
C 0.795026 -0.797896 0.000000
C 2.244070 2.595552 -0.729859
C 3.378225 3.054780 -1.434552
C 3.818514 2.327077 -2.610196
C 3.105451 1.180234 -3.023293
C 4.552872 3.512177 -0.706657
C 4.552872 3.512177 0.706657
C 3.378225 3.054780 1.434552
C 2.244070 2.595552 0.729859
C 1.491989 1.454987 1.182686
C 1.926796 0.739457 2.299905
C 3.105451 1.180234 3.023293
C 3.818514 2.327077 2.610196
C 1.926796 -0.739457 2.299905
C 3.105451 -1.180234 3.023293
C 3.834578 0.000000 3.469592
C 5.240675 0.000000 3.488882
C 5.975085 -1.180389 3.045835
C 5.272627 -2.323124 2.614217
C 3.818514 -2.327077 2.610196
C 3.378225 -3.054780 1.434552
C 2.244070 -2.595552 0.729859
C 1.491989 -1.454987 1.182686
C 5.975085 1.180389 3.045835
C 5.272627 2.323124 2.614217
C 5.726914 3.054692 1.433326
C 5.272627 2.323124 -2.614217
C 5.726914 3.054692 -1.433326
C 6.865970 2.612391 -0.731025
C 6.865970 2.612391 0.731025
C 5.975085 1.180389 -3.045835
C 5.240675 -0.000000 -3.488882
C 3.834578 -0.000000 -3.469592
C 3.105451 -1.180234 -3.023293
C 7.152933 0.728906 -2.323361
C 7.590880 1.435553 -1.181424
C 7.152933 0.728906 2.323361
C 7.590880 1.435553 1.181424
C 8.032822 0.703757 0.000000
C 8.032822 -0.703757 0.000000
C 7.590880 -1.435553 -1.181424
C 7.152933 -0.728906 -2.323361
C 5.975085 -1.180389 -3.045835
C 5.272627 -2.323124 -2.614217
C 3.818514 -2.327077 -2.610196
C 7.590880 -1.435553 1.181424
C 7.152933 -0.728906 2.323361
C 6.865970 -2.612391 0.731025
C 6.865970 -2.612391 -0.731025
C 5.726914 -3.054692 -1.433326
C 4.552872 -3.512177 -0.706657
C 3.378225 -3.054780 -1.434552
C 2.244070 -2.595552 -0.729859
C 4.552872 -3.512177 0.706657
C 5.726914 -3.054692 1.433326

C120-, P1
E = -4563.513892
C 7.423913 1.179731 1.434139
C 6.999756 2.324388 0.730422
C 6.999756 2.324388 -0.730422
C 7.423913 1.179731 -1.434139
C 7.871929 -0.000000 -0.704241
C 7.871929 -0.000000 0.704241
C 5.830872 3.068823 -1.179071
C 5.106782 2.622106 -2.307915
C 5.536019 1.431829 -3.019769
C 6.687075 0.727463 -2.606564
C 6.687075 -0.727463 -2.606564
C 5.536019 -1.431829 -3.019769
C 4.335901 -0.697757 -3.400157
C 4.335901 0.697757 -3.400157
C 3.167491 1.418827 -2.917887
C 2.004673 0.741700 -2.432818
C 2.004673 -0.741700 -2.432818
C 3.167491 -1.418827 -2.917887
C 5.106782 -2.622106 -2.307915
C 3.649242 -2.628628 -2.286567
C 3.649242 2.628628 -2.286567
C 2.969299 3.141589 -1.166090
C 1.742877 2.521910 -0.745397
C 1.214940 1.417017 -1.427446
C 0.000000 0.772345 -0.811353
C 1.214940 -1.417017 -1.427446
C 1.742877 -2.521910 -0.745397
C 2.969299 -3.141589 -1.166090
C 7.423913 -1.179731 -1.434139
C 6.999756 -2.324388 -0.730422
C 5.830872 -3.068823 -1.179071
C 5.122333 -3.545341 0.000000
C 3.713724 -3.582325 0.000000
C -0.000000 0.772345 0.811353
C 1.214940 1.417017 1.427446
C 1.742877 2.521910 0.745397
C 2.969299 3.141589 1.166090
C 3.713724 3.582325 0.000000
C 5.122333 3.545341 0.000000
C 5.830872 3.068823 1.179071
C 5.106782 2.622106 2.307915
C 3.649242 2.628628 2.286567
C 2.004673 0.741700 2.432818
C 3.167491 1.418827 2.917887
C 6.687075 0.727463 2.606564
C 5.536019 1.431829 3.019769
C 4.335901 0.697757 3.400157
C 1.214940 -1.417017 1.427446
C 1.742877 -2.521910 0.745397
C 2.969299 -3.141589 1.166090
C 7.423913 -1.179731 1.434139
C 6.999756 -2.324388 0.730422
C 5.830872 -3.068823 1.179071
C 5.106782 -2.622106 2.307915
C 3.649242 -2.628628 2.286567
C 2.004673 -0.741700 2.432818
C 3.167491 -1.418827 2.917887
C 4.335901 -0.697757 3.400157
C 5.536019 -1.431829 3.019769
C 6.687075 -0.727463 2.606564
C -1.214940 -1.417017 -1.427446
C -1.742877 -2.521910 -0.745397
C -1.742877 -2.521910 0.745397
C -1.214940 -1.417017 1.427446
C -0.000000 -0.772345 0.811353
C -0.000000 -0.772345 -0.811353
C -2.004673 -0.741700 2.432818
C -3.167491 -1.418827 2.917887
C -3.649242 -2.628628 2.286567
C -2.969299 -3.141589 1.166090
C -4.335901 -0.697757 3.400157
C -4.335901 0.697757 3.400157
C -3.167491 1.418827 2.917887
C -2.004673 0.741700 2.432818
C -1.214940 1.417017 1.427446
C -1.742877 2.521910 0.745397
C -2.969299 3.141589 1.166090
C -3.649242 2.628628 2.286567
C -1.742877 2.521910 -0.745397
C -2.969299 3.141589 -1.166090
C -3.713724 3.582325 0.000000
C -5.122333 3.545341 0.000000
C -5.830872 3.068823 -1.179071
C -5.106782 2.622106 -2.307915
C -3.649242 2.628628 -2.286567
C -3.167491 1.418827 -2.917887
C -2.004673 0.741700 -2.432818
C -1.214940 1.417017 -1.427446
C -5.830872 3.068823 1.179071
C -5.106782 2.622106 2.307915
C -5.536019 1.431829 3.019769
C -5.106782 -2.622106 2.307915
C -5.536019 -1.431829 3.019769
C -6.687075 -0.727463 2.606564
C -6.687075 0.727463 2.606564
C -5.830872 -3.068823 1.179071
C -5.122333 -3.545341 -0.000000
C -3.713724 -3.582325 -0.000000
C -2.969299 -3.141589 -1.166090
C -6.999756 -2.324388 0.730422
C -7.423913 -1.179731 1.434139
C -6.999756 2.324388 0.730422
C -7.423913 1.179731 1.434139
C -7.871929 -0.000000 0.704241
C -7.871929 0.000000 -0.704241
C -7.423913 -1.179731 -1.434139
C -6.999756 -2.324388 -0.730422
C -5.830872 -3.068823 -1.179071
C -5.106782 -2.622106 -2.307915
C -3.649242 -2.628628 -2.286567
C -7.423913 1.179731 -1.434139
C -6.999756 2.324388 -0.730422
C -6.687075 0.727463 -2.606564
C -6.687075 -0.727463 -2.606564
C -5.536019 -1.431829 -3.019769
C -4.335901 -0.697757 -3.400157
C -3.167491 -1.418827 -2.917887
C -2.004673 -0.741700 -2.432818
C -4.335901 0.697757 -3.400157
C -5.536019 1.431829 -3.019769

C120-, V1

E = -4563.488814
C 0.000000 2.402476 2.479703
C -0.000000 -2.402476 2.479703
C 0.000000 7.518118 -2.478250
C -0.000000 -7.518118 -2.478250
C 1.181633 -8.298614 -0.439397
C 2.324357 -7.553224 -0.800145
C 2.324903 -6.767500 -2.024660
C 1.182016 -6.749877 -2.853144
C 0.000000 -8.278534 -1.294304
C 3.051340 -5.530961 -1.766654
C 2.614779 -4.322001 -2.341872
C 1.432022 -4.304670 -3.198655
C 0.730508 -5.501035 -3.446708
C -0.730508 -5.501035 -3.446708
C -1.432022 -4.304670 -3.198655
C -0.707416 -3.068076 -2.947907
C 0.707416 -3.068076 -2.947907
C 1.433220 -2.325014 -1.929593
C 0.730159 -1.607919 -0.943186
C -0.730159 -1.607919 -0.943186
C -1.433220 -2.325014 -1.929593
C -2.614779 -4.322001 -2.341872
C -2.616075 -3.096371 -1.556454
C 2.616075 -3.096371 -1.556454
C 3.051315 -3.117034 -0.217673
C 2.326812 -2.366901 0.801074
C 1.181826 -1.625861 0.439447
C 0.000000 -1.647496 1.293622
C -1.181826 -1.625861 0.439447
C -2.326812 -2.366901 0.801074
C -3.051315 -3.117034 -0.217673
C -1.182016 -6.749877 -2.853144
C -2.324903 -6.767500 -2.024660
C -3.051340 -5.530961 -1.766654
C -3.502694 -5.553226 -0.380416
C -3.501682 -4.368418 0.379510
C 1.182546 -3.170086 2.854262
C 2.325032 -3.152657 2.024486
C 3.049626 -4.390544 1.766010
C 3.501682 -4.368418 0.379510
C 3.502694 -5.553226 -0.380416
C 3.050820 -6.803817 0.217537
C 2.614911 -6.824542 1.556107
C 2.614324 -5.598892 2.342636
C 0.730892 -4.419078 3.446026
C 1.432676 -5.615909 3.199783
C 0.730364 -8.318665 0.943681
C 1.432316 -7.595758 1.928072
C 0.707073 -6.851647 2.947449
C -1.182546 -3.170086 2.854262
C -2.325032 -3.152657 2.024486
C -3.049626 -4.390544 1.766010
C -1.181633 -8.298614 -0.439397
C -2.324357 -7.553224 -0.800145
C -3.050820 -6.803817 0.217537
C -2.614911 -6.824542 1.556107
C -2.614324 -5.598892 2.342636
C -0.730892 -4.419078 3.446026
C -1.432676 -5.615909 3.199783
C -0.707073 -6.851647 2.947449
C -1.432316 -7.595758 1.928072
C -0.730364 -8.318665 0.943681
C -1.181826 1.625861 0.439447
C -2.326812 2.366901 0.801074
C -2.325032 3.152657 2.024486
C -1.182546 3.170086 2.854262
C 0.000000 1.647496 1.293622
C -0.730892 4.419078 3.446026
C -1.432676 5.615909 3.199783
C -2.614324 5.598892 2.342636
C -3.049626 4.390544 1.766010
C -0.707073 6.851647 2.947449
C 0.707073 6.851647 2.947449
C 1.432676 5.615909 3.199783
C 0.730892 4.419078 3.446026
C 1.182546 3.170086 2.854262
C 2.325032 3.152657 2.024486
C 3.049626 4.390544 1.766010
C 2.614324 5.598892 2.342636
C 2.326812 2.366901 0.801074
C 3.051315 3.117034 -0.217673
C 3.501682 4.368418 0.379510
C 3.502694 5.553226 -0.380416
C 3.051340 5.530961 -1.766654
C 2.614779 4.322001 -2.341872
C 2.616075 3.096371 -1.556454
C 1.433220 2.325014 -1.929593
C 0.730159 1.607919 -0.943186
C 1.181826 1.625861 0.439447
C 3.050820 6.803817 0.217537
C 2.614911 6.824542 1.556107
C 1.432316 7.595758 1.928072
C -2.614911 6.824542 1.556107
C -1.432316 7.595758 1.928072
C -0.730364 8.318665 0.943681
C 0.730364 8.318665 0.943681
C -3.050820 6.803817 0.217537
C -3.502694 5.553226 -0.380416
C -3.501682 4.368418 0.379510
C -3.051315 3.117034 -0.217673
C -2.324357 7.553224 -0.800145
C -1.181633 8.298614 -0.439397
C 2.324357 7.553224 -0.800145
C 1.181633 8.298614 -0.439397
C 0.000000 8.278534 -1.294304
C -1.182016 6.749877 -2.853144
C -2.324903 6.767500 -2.024660
C -3.051340 5.530961 -1.766654
C -2.614779 4.322001 -2.341872
C -2.616075 3.096371 -1.556454
C 1.182016 6.749877 -2.853144
C 2.324903 6.767500 -2.024660
C 0.730508 5.501035 -3.446708
C -0.730508 5.501035 -3.446708
C -1.432022 4.304670 -3.198655
C -0.707416 3.068076 -2.947907
C -1.433220 2.325014 -1.929593
C -0.730159 1.607919 -0.943186
C 0.707416 3.068076 -2.947907
C 1.432022 4.304670 -3.198655

Benchmarking calculations: *ω*B97XD/def2-SVP

C60
E = -2283.435135
C 0.000000 -1.234118 3.317751
C -0.000000 -2.415572 2.587572
C 1.173716 -2.796936 1.824845
C 2.297346 -0.746453 2.587572
C 1.173716 -0.381363 3.317751
C 0.725396 0.998423 3.317751
C -0.725396 0.998423 3.317751
C -1.173716 -0.381363 3.317751
C -0.000000 1.234118 -3.317751
C 0.000000 2.415572 -2.587572
C -1.173716 2.796936 -1.824845
C -1.173716 0.381363 -3.317751
C -2.297346 1.980571 -1.824845
C -0.725396 -0.998423 -3.317751
C 0.725396 -0.998423 -3.317751
C 1.173716 0.381363 -3.317751
C -1.173716 -2.796936 1.824845
C 3.022742 -1.744875 0.590727
C 2.297346 -1.980571 1.824845
C 2.593554 1.572876 1.824845
C -0.694441 2.952662 1.824845
C -3.022742 0.251970 1.824845
C 1.173716 2.796936 -1.824845
C -3.022742 1.744875 -0.590727
C -2.297346 -1.980571 1.824845
C -2.593554 -2.335603 -0.590727
C 1.419838 -3.188357 -0.590727
C 3.471062 0.365089 -0.590727
C 0.725396 3.413995 -0.590727
C 2.593554 2.335603 0.590727
C 0.694441 -2.952662 -1.824845
C -1.419838 3.188357 0.590727
C -3.471062 -0.365089 0.590727
C -0.725396 -3.413995 0.590727
C 3.022742 -0.251970 -1.824845
C -2.593554 -1.572876 -1.824845
C 0.725396 -3.413995 0.590727
C 2.297346 0.746453 -2.587572
C -2.297346 -0.746453 2.587572
C 1.419838 1.954239 2.587572
C -1.419838 1.954239 2.587572
C -1.419838 -3.188357 -0.590727
C 1.419838 -1.954239 -2.587572
C 1.419838 3.188357 0.590727
C -2.593554 2.335603 0.590727
C -3.022742 -1.744875 0.590727
C 3.471062 -0.365089 0.590727
C 2.593554 -2.335603 -0.590727
C 3.022742 1.744875 -0.590727
C -0.725396 3.413995 -0.590727
C -3.471062 0.365089 -0.590727
C 2.593554 -1.572876 -1.824845
C -1.419838 -1.954239 -2.587572
C 2.297346 1.980571 -1.824845
C -2.297346 0.746453 -2.587572
C 3.022742 0.251970 1.824845
C 0.694441 2.952662 1.824845
C -2.593554 1.572876 1.824845
C -3.022742 -0.251970 -1.824845
C -0.694441 -2.952662 -1.824845

C60-
E = -2283.526199
C -0.274681 -3.352922 1.114537
C -0.128332 -1.610885 -3.142207
C 0.342634 -2.634907 -2.328952
C 1.682153 -2.564473 -1.778404
C 2.497701 -1.470149 -2.059413
C 2.006876 -0.402618 -2.907121
C 0.720954 -0.473368 -3.438805
C -0.527663 -3.240123 -1.334336
C 0.262770 -3.529926 -0.168879
C 1.627515 -3.105967 -0.434336
C -1.844879 -2.777298 -1.200769
C -2.402174 -2.588667 0.128387
C -1.637995 -2.868835 1.255010
C 0.517216 -2.762470 2.159229
C -0.350363 -1.908629 2.953282
C -1.690056 -1.984632 2.403173
C -3.250278 -1.412696 0.102787
C -3.298143 -0.560380 1.203730
C -2.501499 -0.852514 2.378326
C -2.006876 0.402618 2.907121
C -0.720954 0.473368 3.438805
C 0.124120 -0.704646 3.460380
C 1.492530 -0.288427 3.200943
C 2.326759 -1.111427 2.430525
C 1.835992 -2.356955 1.906160
C 2.393053 -2.535128 0.575578
C -3.193716 -0.866383 -1.239380
C -2.332044 -1.718069 -2.042058
C -1.495130 -1.135983 -3.005641
C -1.492530 0.288427 -3.200943
C -2.326759 1.111427 -2.430525
C -3.191388 0.510597 -1.428270
C -3.295630 0.875092 1.006760
C -3.245309 1.398717 -0.283465

C -2.497701 1.470149 2.059413
C -2.393053 2.535128 -0.575578
C -1.627515 3.105967 0.434336
C -1.682153 2.564473 1.778404
C 0.128332 1.610885 3.142207
C -0.342634 2.634907 2.328952
C 1.495130 1.135983 3.005641
C 0.527663 3.240123 1.334336
C 1.844879 2.777298 1.200769
C 2.332044 1.718069 2.042058
C 3.191388 -0.510597 1.428270
C 3.193716 0.866383 1.239380
C 3.245309 -1.398717 0.283465
C 3.295630 -0.875092 -1.006760
C 3.250278 1.412696 -0.102787
C 3.298143 0.560380 -1.203730
C 2.501499 0.852514 -2.378326
C 1.690056 1.984632 -2.403173
C 1.637995 2.868835 -1.255010
C 2.402174 2.588667 -0.128387
C 0.350363 1.908629 -2.953282
C -0.517216 2.762470 -2.159229
C 0.274681 3.352922 -1.114537
C -0.262770 3.529926 0.168879
C -1.835992 2.356955 -1.906160
C -0.124120 0.704646 -3.460380

C120-, B1
E = -4567.480492
C -2.559931 2.303461 4.281942
C -2.570384 1.563003 5.518217
C -2.998175 0.246372 5.518252
C -3.425089 -0.358783 4.281971
C -3.425089 0.358783 -4.281971
C -2.981871 1.722845 -4.281959
C -1.416183 1.949181 6.299332
C -2.291426 -0.744564 6.299397
C -2.981871 -1.722845 4.281959
C -2.998175 -0.246372 -5.518252
C -2.280782 1.961486 -5.518153
C -1.399609 3.146487 4.281959
C -2.570384 -1.563003 -5.518217
C -2.559931 -2.303461 -4.281942
C -1.416183 -1.949181 -6.299332
C -1.399609 -3.146487 -4.281959
C -0.725053 0.997955 7.045951
C -2.280782 -1.961486 5.518153
C -0.692185 -2.927553 -5.518226
C -1.160794 -2.775257 5.518132
C -1.173131 -0.381240 7.046023
C -2.291426 0.744564 -6.299397
C -1.160794 2.775257 -5.518132
C 0.725053 0.997955 7.045951
C -0.717130 3.368192 -4.281895
C -0.692185 2.927553 5.518226
C 0.000000 2.409411 -6.299287
C 0.717130 3.368192 -4.281895
C 0.692185 2.927553 5.518226
C -0.717130 -3.368192 4.281895
C -1.173131 0.381240 -7.046023
C 0.000000 -1.233657 7.045962
C 1.160794 2.775257 -5.518132
C 1.399609 3.146487 4.281959
C 1.416183 1.949181 6.299332
C 2.570384 1.563003 5.518217
C 1.173131 -0.381240 7.046023
C 0.000000 -2.409411 6.299287
C 1.160794 -2.775257 5.518132
C 2.559931 2.303461 4.281942
C 2.981871 1.722845 -4.281959
C 3.425089 0.358783 -4.281971
C 2.280782 1.961486 -5.518153
C -0.725053 -0.997955 -7.045951
C 2.280782 -1.961486 5.518153
C 0.000000 1.233657 -7.045962
C 1.173131 0.381240 -7.046023
C 2.291426 -0.744564 6.299397
C 0.717130 -3.368192 4.281895
C 1.399609 -3.146487 -4.281959
C 2.998175 0.246372 5.518252
C 3.425089 -0.358783 4.281971
C 2.291426 0.744564 -6.299397
C 2.998175 -0.246372 -5.518252
C 2.981871 -1.722845 4.281959
C 0.725053 -0.997955 -7.045951
C 0.692185 -2.927553 -5.518226
C 2.559931 -2.303461 -4.281942
C 1.416183 -1.949181 -6.299332
C 2.570384 -1.563003 -5.518217
C 1.422929 3.190689 -3.082455
C 0.710466 3.372293 -1.846760
C -0.710466 3.372293 -1.846760
C -1.422929 3.190689 -3.082455
C 2.594923 2.339237 -3.082462
C -2.594923 2.339237 -3.082462
C 0.724303 3.417763 3.082447
C -0.724303 3.417763 3.082447
C 3.026533 1.744959 3.082426
C 2.556961 2.310660 1.846750
C 1.407404 3.145886 1.846768
C -1.407404 3.145886 1.846768
C -2.556961 2.310660 1.846750
C -3.026533 1.744959 3.082426
C 3.474206 0.367239 3.082457
C 2.594923 -2.339237 3.082462
C 2.987809 -1.717816 1.846755
C 3.426834 -0.366376 1.846764
C 3.026533 -1.744959 -3.082426
C 3.474206 -0.367239 -3.082457
C 3.426834 0.366376 -1.846764
C 2.987809 1.717816 -1.846755
C 1.422929 -3.190689 3.082455
C 0.724303 -3.417763 -3.082447
C 1.407404 -3.145886 -1.846768
C 2.556961 -2.310660 -1.846750
C -0.724303 -3.417763 -3.082447
C -3.026533 -1.744959 -3.082426
C -2.556961 -2.310660 -1.846750
C -1.407404 -3.145886 -1.846768
C -2.594923 -2.339237 3.082462
C -1.422929 -3.190689 3.082455
C -3.474206 -0.367239 -3.082457
C -2.987809 1.717816 -1.846755
C -3.426834 0.366376 -1.846764
C -3.474206 0.367239 3.082457
C -0.710466 -3.372293 1.846760
C 0.710466 -3.372293 1.846760
C -1.397809 3.141952 -0.610298
C -2.556270 2.300359 -0.610301
C 2.556270 2.300359 -0.610301
C 1.397809 3.141952 -0.610298
C 2.977684 -1.720274 -0.610293
C 3.420126 -0.358469 -0.610303
C -0.715920 -3.363567 -0.610292
C 0.715920 -3.363567 -0.610292
C -3.420126 -0.358469 -0.610303
C -2.977684 -1.720274 -0.610293
C -3.426834 -0.366376 1.846764
C -2.987809 -1.717816 1.846755
C 0.715920 3.363567 0.610292
C -0.715920 3.363567 0.610292
C 3.420126 0.358469 0.610303
C 2.977684 1.720274 0.610293
C 1.397809 -3.141952 0.610298
C 2.556270 -2.300359 0.610301
C -2.556270 -2.300359 0.610301
C -1.397809 -3.141952 0.610298
C -2.977684 1.720274 0.610293
C -3.420126 0.358469 0.610303

C120-, C1
E = -4567.005183
C -1.173359 -7.548456 -1.421716
C -2.295599 -7.112606 -0.726482
C -2.295599 -7.112606 0.726482
C -1.173359 -7.548456 1.421716
C -0.000015 -7.995374 0.694661
C -0.000015 -7.995374 -0.694661
C -3.017312 -5.940394 1.175396
C -2.590851 -5.249500 2.303452
C -1.419711 -5.704250 3.030362
C -0.725634 -6.828775 2.597831
C 0.725606 -6.828776 2.597831
C 1.419684 -5.704252 3.030362
C 0.693931 -4.533147 3.481844
C -0.693956 -4.533147 3.481844
C -1.422512 -3.359521 3.031749
C -0.725318 -2.240414 2.583230
C 0.725296 -2.240415 2.583230
C 1.422489 -3.359523 3.031749
C 2.590825 -5.249503 2.303452
C 2.587246 -3.799480 2.309910
C -2.587269 -3.799477 2.309910
C -2.990695 -3.097938 1.174788
C -2.272360 -1.925528 0.740952
C -1.170470 -1.497192 1.439689
C -0.000010 -0.785680 0.792013
C 1.170449 -1.497194 1.439689
C 2.272339 -1.925532 0.740952
C 2.990672 -3.097942 1.174788
C 1.173330 -7.548458 1.421716
C 2.295570 -7.112609 0.726482
C 3.017286 -5.940398 1.175396
C 3.456774 -5.210153 0.000000
C 3.435654 -3.822114 0.000000
C -0.000010 -0.785680 -0.792013
C -1.170470 -1.497192 -1.439689
C -2.272360 -1.925528 -0.740952
C -2.990695 -3.097938 -1.174788
C -3.435678 -3.822109 -0.000000
C -3.456799 -5.210148 -0.000000
C -3.017312 -5.940394 -1.175396
C -2.590851 -5.249500 -2.303452
C -2.587269 -3.799477 -2.309910
C -0.725318 -2.240414 -2.583230
C -1.422512 -3.359521 -3.031749
C -0.725634 -6.828775 -2.597831
C -1.419711 -5.704250 -3.030362
C -0.693956 -4.533147 -3.481844
C 1.170449 -1.497194 -1.439689
C 2.272339 -1.925532 -0.740952
C 2.990672 -3.097942 -1.174788
C 1.173330 -7.548458 -1.421716
C 2.295570 -7.112609 -0.726482
C 3.017286 -5.940398 -1.175396
C 2.590825 -5.249503 -2.303452
C 2.587246 -3.799480 -2.309910
C 0.725296 -2.240415 -2.583230
C 1.422489 -3.359523 -3.031749
C 0.693931 -4.533147 -3.481844
C 1.419684 -5.704252 -3.030362
C 0.725606 -6.828776 -2.597831
C 1.172743 1.489657 1.447182
C 2.271047 1.918154 0.737922
C 2.271047 1.918154 -0.737922
C 1.172743 1.489657 -1.447182
C -0.000005 0.785325 -0.793198
C -0.000005 0.785325 0.793198
C 0.722131 2.225827 -2.590063
C 1.425945 3.354302 -3.033697
C 2.576818 3.796959 -2.303985
C 2.993270 3.098028 -1.166430
C 0.694506 4.533947 -3.469344
C -0.694479 4.533955 -3.469345
C -1.425928 3.354312 -3.033697

C -0.722124 2.225840 -2.590060
C -1.172748 1.489665 -1.447178
C -2.271046 1.918172 -0.737926
C -2.993261 3.098048 -1.166434
C -2.576801 3.796979 -2.303983
C -2.271046 1.918172 0.737926
C -2.993261 3.098048 1.166434
C -3.448820 3.812505 -0.000000
C -3.466323 5.219259 -0.000000
C -3.015546 5.939557 1.166847
C -2.582485 5.252258 2.300589
C -2.576801 3.796979 2.303983
C -1.425928 3.354312 3.033697
C -0.722124 2.225840 2.590060
C -1.172748 1.489665 1.447178
C -3.015546 5.939557 -1.166847
C -2.582485 5.252258 -2.300589
C -1.422518 5.707185 -3.029906
C 2.582518 5.252242 -2.300591
C 1.422553 5.707173 -3.029899
C 0.720823 6.841418 -2.601177
C -0.720778 6.841418 -2.601175
C 3.015582 5.939535 -1.166844
C 3.466360 5.219241 0.000000
C 3.448841 3.812476 0.000000
C 2.993270 3.098028 1.166430
C 2.292933 7.120222 -0.724504
C 1.171774 7.555072 -1.423398
C -2.292887 7.120236 -0.724505
C -1.171724 7.555080 -1.423395
C 0.000027 8.005437 -0.694301
C 0.000027 8.005437 0.694301
C 1.171774 7.555072 1.423398
C 2.292933 7.120222 0.724504
C 3.015582 5.939535 1.166844
C 2.582518 5.252242 2.300591
C 2.576818 3.796959 2.303985
C -1.171724 7.555080 1.423395
C -2.292887 7.120236 0.724505
C -0.720778 6.841418 2.601175
C 0.720823 6.841418 2.601177
C 1.422553 5.707173 3.029899
C 0.694506 4.533947 3.469344
C 1.425945 3.354302 3.033697
C 0.722131 2.225827 2.590063
C -0.694479 4.533955 3.469345
C -1.422518 5.707185 3.029906

C120-, P1
E = -4567.023133
C -1.421162 1.174657 7.373503
C -0.727553 2.303826 6.952107
C 0.727553 2.303826 6.952107
C 1.421162 1.174657 7.373503
C 0.694894 0.000000 7.819636
C -0.694894 -0.000000 7.819636
C 1.176889 3.041327 5.790558
C 2.288938 2.602872 5.075770
C 2.996153 1.416378 5.503415
C 2.586275 0.725249 6.637485
C 2.586275 -0.725249 6.637485
C 2.996153 -1.416378 5.503415
C 3.382418 -0.688446 4.310381
C 3.382418 0.688446 4.310381
C 2.898755 1.407128 3.147806
C 2.420711 0.741027 2.001308
C 2.420711 -0.741027 2.001308
C 2.898755 -1.407128 3.147806
C 2.288938 -2.602872 5.075770
C 2.273938 -2.611785 3.626067
C 2.273938 2.611785 3.626067
C 1.163429 3.107711 2.954205
C 0.746697 2.496809 1.738142
C 1.415659 1.406841 1.213661
C 0.802066 0.767577 0.001046
C 1.415659 -1.406841 1.213661
C 0.746697 -2.496809 1.738142
C 1.163429 -3.107711 2.954205
C 1.421162 -1.174657 7.373503
C 0.727553 -2.303826 6.952107
C 1.176889 -3.041327 5.790558
C 0.000000 -3.511106 5.081196
C 0.000000 -3.541842 3.697949
C -0.802066 0.767577 0.001046
C -1.415659 1.406841 1.213661
C -0.746697 2.496809 1.738142
C -1.163429 3.107711 2.954205
C -0.000000 3.541842 3.697949
C -0.000000 3.511106 5.081196
C -1.176889 3.041327 5.790558
C -2.288938 2.602872 5.075770
C -2.273938 2.611785 3.626067
C -2.420711 0.741027 2.001308
C -2.898755 1.407128 3.147806
C -2.586275 0.725249 6.637485
C -2.996153 1.416378 5.503415
C -3.382418 0.688446 4.310381
C -1.415659 -1.406841 1.213661
C -0.746697 -2.496809 1.738142
C -1.163429 -3.107711 2.954205
C -1.421162 -1.174657 7.373503
C -0.727553 -2.303826 6.952107
C -1.176889 -3.041327 5.790558
C -2.288938 -2.602872 5.075770
C -2.273938 -2.611785 3.626067
C -2.420711 -0.741027 2.001308
C -2.898755 -1.407128 3.147806
C -3.382418 -0.688446 4.310381
C -2.996153 -1.416378 5.503415
C -2.586275 -0.725249 6.637485
C 1.421441 -1.409229 -1.211097
C 0.744304 -2.499094 -1.729343
C -0.744304 -2.499094 -1.729343
C -1.421441 -1.409229 -1.211097
C -0.802066 -0.767577 0.001046
C 0.802066 -0.767577 0.001046
C -2.427665 -0.737078 -1.986449
C -2.897533 -1.408536 -3.143708
C -2.265401 -2.601068 -3.623297
C -1.154560 -3.111219 -2.954042
C -3.369978 -0.689018 -4.312193
C -3.369978 0.689018 -4.312193
C -2.897533 1.408536 -3.143708
C -2.427665 0.737078 -1.986449
C -1.421441 1.409229 -1.211097
C -0.744304 2.499094 -1.729343
C -1.154560 3.111219 -2.954042
C -2.265401 2.601068 -3.623297
C 0.744304 2.499094 -1.729343
C 1.154560 3.111219 -2.954042
C 0.000000 3.559122 -3.687859
C 0.000000 3.518409 -5.089214
C 1.168093 3.039698 -5.789184
C 2.286474 2.595515 -5.078531
C 2.265401 2.601068 -3.623297
C 2.897533 1.408536 -3.143708
C 2.427665 0.737078 -1.986449
C 1.421441 1.409229 -1.211097
C -1.168093 3.039698 -5.789184
C -2.286474 2.595515 -5.078531
C -2.997391 1.420448 -5.506505
C -2.286474 -2.595515 -5.078531
C -2.997391 -1.420448 -5.506505
C -2.589711 -0.720281 -6.649139
C -2.589711 0.720281 -6.649139
C -1.168093 -3.039698 -5.789184
C 0.000000 -3.518409 -5.089214
C 0.000000 -3.559122 -3.687859
C 1.154560 -3.111219 -2.954042
C -0.725381 -2.300778 -6.959281
C -1.422614 -1.172879 -7.379024
C -0.725381 2.300778 -6.959281
C -1.422614 1.172879 -7.379024
C -0.694440 -0.000000 -7.829031
C 0.694440 0.000000 -7.829031
C 1.422614 -1.172879 -7.379024
C 0.725381 -2.300778 -6.959281
C 1.168093 -3.039698 -5.789184
C 2.286474 -2.595515 -5.078531
C 2.265401 -2.601068 -3.623297
C 1.422614 1.172879 -7.379024
C 0.725381 2.300778 -6.959281
C 2.589711 0.720281 -6.649139
C 2.589711 -0.720281 -6.649139
C 2.997391 -1.420448 -5.506505
C 3.369978 -0.689018 -4.312193
C 2.897533 -1.408536 -3.143708
C 2.427665 -0.737078 -1.986449
C 3.369978 0.689018 -4.312193
C 2.997391 1.420448 -5.506505

C120-, V1
E = -4566.980466
6 2.413193 -2.331811 0.000000
6 2.405927 2.308461 -0.000000
6 -2.413760 -7.478998 0.000000
6 -2.404603 7.500320 -0.000000
6 -0.368041 8.224736 1.173981
6 -0.736438 7.495549 2.298186
6 -1.973425 6.737817 2.299117
6 -2.790157 6.740053 1.174266
6 -1.220699 8.227438 -0.000000
6 -1.742955 5.503946 3.025727
6 -2.335739 4.322881 2.594101
6 -3.188143 4.325253 1.418810
6 -3.411418 5.508932 0.725655
6 -3.411418 5.508932 -0.725655
6 -3.188143 4.325253 -1.418810
6 -2.961325 3.088921 -0.695966
6 -2.961325 3.088921 0.695966
6 -1.966832 2.324724 1.420659
6 -1.010033 1.599063 0.724890
6 -1.010033 1.599063 -0.724890
6 -1.966832 2.324724 -1.420659
6 -2.335739 4.322881 -2.594101
6 -1.580622 3.085866 -2.596289
6 -1.580622 3.085866 2.596289
6 -0.259214 3.080380 3.024277
6 0.738161 2.311866 2.302129
6 0.367867 1.591037 1.173761
6 1.218406 1.591602 -0.000000
6 0.367867 1.591037 -1.173761
6 0.738161 2.311866 -2.302129
6 -0.259214 3.080380 -3.024277
6 -2.790157 6.740053 -1.174266
6 -1.973425 6.737817 -2.299117
6 -1.742955 5.503946 -3.025727
6 -0.363621 5.498127 -3.473715
6 0.361597 4.312706 -3.470913
6 2.791889 3.066950 1.175085
6 1.972662 3.069936 2.299073
6 1.741680 4.306528 3.021615
6 0.361597 4.312706 3.470913
6 -0.363621 5.498127 3.473715
6 0.259143 6.729211 3.024200
6 1.580289 6.723217 2.594577
6 2.338442 5.486255 2.594294
6 3.408874 4.298864 0.726113
6 3.191586 5.483326 1.420754
6 1.011584 8.218710 0.725560
6 1.964609 7.483502 1.419691
6 2.961013 6.717258 0.695282
6 2.791889 3.066950 -1.175085
6 1.972662 3.069936 -2.299073
6 1.741680 4.306528 -3.021615

6 -0.368041 8.224736 -1.173981
6 -0.736438 7.495549 -2.298186
6 0.259143 6.729211 -3.024200
6 1.580289 6.723217 -2.594577
6 2.338442 5.486255 -2.594294
6 3.408874 4.298864 -0.726113
6 3.191586 5.483326 -1.420754
6 2.961013 6.717258 -0.695282
6 1.964609 7.483502 -1.419691
6 1.011584 8.218710 -0.725560
6 0.381804 -1.588979 -1.177795
6 0.756863 -2.316284 -2.316159
6 1.979501 -3.075788 -2.311205
6 2.804320 -3.081871 -1.178850
6 1.227589 -1.606528 0.000000
6 3.407087 -4.312802 -0.728306
6 3.185368 -5.502662 -1.420270
6 2.332248 -5.497639 -2.598631
6 1.744402 -4.314708 -3.029572
6 2.956690 -6.733127 -0.701116
6 2.956690 -6.733127 0.701116
6 3.185368 -5.502662 1.420270
6 3.407087 -4.312802 0.728306
6 2.804320 -3.081871 1.178850
6 1.979501 -3.075788 2.311205
6 1.744402 -4.314708 3.029572
6 2.332248 -5.497639 2.598631
6 0.756863 -2.316284 2.316159
6 -0.247306 -3.080385 3.030638
6 0.364954 -4.317032 3.482110
6 -0.366387 -5.497612 3.482167
6 -1.746450 -5.498624 3.031825
6 -2.331608 -4.312058 2.600453
6 -1.569928 -3.082096 2.601780
6 -1.956541 -2.322218 1.420653
6 -0.987256 -1.597645 0.727903
6 0.381804 -1.588979 1.177795
6 0.248212 -6.732524 3.030463
6 1.569650 -6.729542 2.598991
6 1.953183 -7.487938 1.419658
6 1.569650 -6.729542 -2.598991
6 1.953183 -7.487938 -1.419658
6 0.991038 -8.218163 -0.727535
6 0.991038 -8.218163 0.727535
6 0.248212 -6.732524 -3.030463
6 -0.366387 -5.497612 -3.482167
6 0.364954 -4.317032 -3.482110
6 -0.247306 -3.080385 -3.030638
6 -0.753209 -7.492195 -2.307232
6 -0.383169 -8.222248 -1.176682
6 -0.753209 -7.492195 2.307232
6 -0.383169 -8.222248 1.176682
6 -1.232875 -8.212240 0.000000
6 -2.801189 -6.723180 -1.177169
6 -1.982197 -6.731342 -2.309564
6 -1.746450 -5.498624 -3.031825
6 -2.331608 -4.312058 -2.600453
6 -1.569928 -3.082096 -2.601780
6 -2.801189 -6.723180 1.177169
6 -1.982197 -6.731342 2.309564
6 -3.408188 -5.492403 0.728441
6 -3.408188 -5.492403 -0.728441
6 -3.181532 -4.304744 -1.419989
6 -2.958388 -3.068928 -0.703908
6 -1.956541 -2.322218 -1.420653
6 -0.987256 -1.597645 -0.727903
6 -2.958388 -3.068928 0.703908
6 -3.181532 -4.304744 1.419989
